# Direct Activation of Sulfides by C–H Oxidation with Photoexcited Nitroarenes: Formal Manipulations of the C─S Bond

**DOI:** 10.1002/anie.202509244

**Published:** 2025-06-18

**Authors:** Valentina D. Cuomo, Ciro Romano, David J. Procter

**Affiliations:** ^1^ Department of Chemistry University of Manchester Oxford Road Manchester M139PL U K

**Keywords:** Anaerobic oxidation, C(sp^3^)−S manipulation, Nitrobenzene, Photochemistry, Sulfides

## Abstract

The sulfide motif is distributed widely across chemical and biological space. In synthesis, its installation often marks the end point of a sequence, due to its relative inertness; sulfides typically require direct oxidation of sulfur before they are receptive toward transformation. Unfortunately, selective S‐oxidation is not always straightforward, with the need for oxidants lacking chemoselectivity in the presence of functionality and delivering mixtures of oxidation products. This multistep manipulation of the sulfide motif, initiated by direct S‐oxidation, limits the use of sulfides as synthetic handles for downstream manipulation. Herein, we describe a direct activation of sulfides by C–H oxidation alpha to sulfur—rather than traditional oxidation at sulfur—that facilitates efficient formal C─S bond manipulation. The mild nature of the photo‐induced anaerobic oxidation protocol enables its merger with high‐value transformations in telescoped or one‐pot protocols that deliver branched amines, secondary alcohols, and alkenes from aldehyde and ketone intermediates. The method expands the chemistry of sulfides by diverting reactivity away from sulfur (oxidation, alkylation) and instead targeting directly the alpha position, resulting in formal manipulation of the C─S bond, and redefining sulfides as latent synthetic handles to be “switched on” at will.

## Graphical abstract

Photochemical anaerobic oxidation of sulfides, using nitroarenes as bifunctional reagents, enables the formal manipulation of the C─S bond. The method allows direct and selective access to versatile carbonyl compounds from largely inert sulfides by C–H oxidation, thus avoiding multistep activation by sulfur oxidation. The mild photo‐induced protocol has been engaged in telescoped procedures to convert sulfides into amines, alcohols, and alkenes.

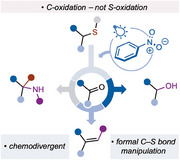

The sulfide motif is a fundamental functional group that plays key roles in many areas of science^[^ ^1^, ^2^, ^3^ ^]^ – from functional materials (e.g., MOFs),^[^ ^4^, ^5^, ^6^ ^]^ through ligand design for metal catalysis (e.g., for Pd‐catalysed Tsuji‐Trost allylation),^[^ ^7^, ^8^ ^]^ to natural product chemistry and drug design (e.g., Montelukast and Diltiazem) (Scheme 1a).^[^ ^9^, ^10^ ^]^ Due to their relative inertness, however, sulfides do not feature among the most used building blocks for synthesis; the motif can often be installed early in a synthesis and carried through intact.^[^ ^11^, ^12^ ^]^ In fact, sulfide modification is typically challenging and step intensive; activation of the S‐atom by oxidation to a S(IV) or S(VI) species is required as a prelude to further manipulation (Scheme 1b).^[^ ^13^, ^14^ ^]^ The oxidative activation of sulfur can be difficult to achieve as it relies on the use of oxidants lacking chemoselectivity in the presence of functionality and delivering mixtures of oxidation products. Crucially, only when S(IV) or S(VI) species are obtained can classical approaches for functionalization, for example, at the carbon alpha to sulfur, be engaged (e.g., ylide formation, sulfone alkylation, Pummerer reaction, desulfonylation).^[^ ^15^, ^16^, ^17^, ^18^, ^19^, ^20^, ^21^, ^22^, ^23^, ^24^, ^25^, ^26^ ^]^ Building on our interest in Pummerer chemistry^[^ ^27^ ^]^ and light‐induced methods for the α‐functionalization of sulfides,^[^ ^28^, ^29^, ^30^, ^31^ ^]^ we envisioned a method for the direct activation of sulfides using light to promote C–H oxidation alpha to sulfur – rather than traditional oxidation at sulfur. Such activation would facilitate expedient formal C(sp^3^)─S bond manipulation and reduce the step‐count involved in the transformation of the sulfide unit. Crucially, if the activating C–H oxidation alpha to sulfur could be carried out under anaerobic conditions, then functional group tolerance might be improved (Scheme 1c).

**Scheme 1:**
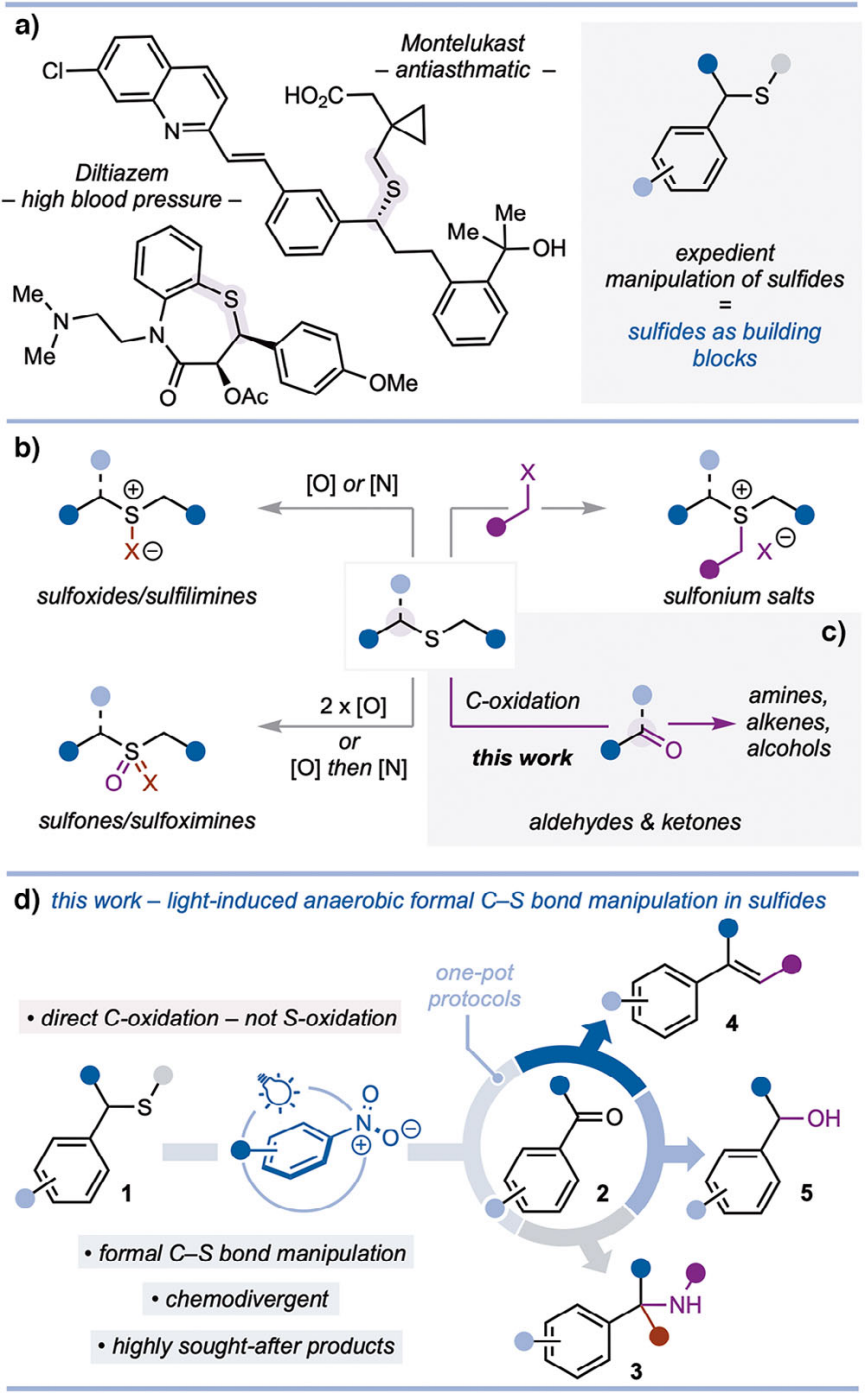
a) Sulfides are important motifs in bioactive molecules but are under‐exploited as building blocks for synthesis. b) Established multistep methods for sulfide manipulation focus on the oxidation of sulfur, rather than, c) direct oxidation at the alpha carbon. d) Formal C─S bond manipulation in benzylic sulfides by direct oxidation at the alpha carbon using photoexcited nitroarenes; application in chemodivergent, telescoped processes.

We reasoned that such a selective protocol would complement existing methods and help redefine sulfide units as useful, latent synthetic handles in valuable building blocks, that can be “switched on”—under specific conditions—for the introduction of molecular complexity.

We envisaged that photoexcited nitroarenes **B** could serve as bifunctional reagents in activating sulfides by mediating HAT alpha to sulfur and subsequent coupling of the nucleophilic **1‐rad** with the electrophilic oxyl radical **C**.^[^ ^32^, ^33^, ^34^, ^35^, ^36^, ^37^, ^38^, ^39^, ^40^, ^41^, ^42^ ^]^ Intermediate **D** would then undergo fragmentation, to expel thiol and deliver aldehyde or ketone products **2** (Scheme 2).^[^ ^36^ ^]^ Challenges include, i) avoiding competing oxidation of sulfur in the sulfide or thiol byproduct, by the triplet nitroarene,^[^ ^41^ ^]^ ii) achieving efficient radical–radical coupling between the carbon‐centred radical and the oxyl radical,^[^ ^43^, ^44^ ^]^ and iii) avoiding “over‐oxidation” of aldehyde products—some photoexcited nitroarenes are known to oxidize aldehydes to carboxylic acids.^[^ ^36^ ^]^ Regarding the latter point, as aldehydes cannot be obtained by oxidation of alcohols using photoexcited nitroarenes due to their facile over‐oxidation, accessing them by oxidation of sulfides under similar conditions is noteworthy and highlights the complementary reactivity of these two classes of compounds. We reasoned that the greater difference in bond‐dissociation energy (BDE) between the acyl C─H bond of aldehydes and α‐heteroatom C─H bonds in sulfides, compared to alcohols, would ensure the desired chemoselective oxidation.^[^ ^45^ ^]^ This mechanism differs substantially from other reported methods for the manipulation of C─S bonds, which proceed via the formation of carbocation intermediates.^[^ ^46^, ^47^, ^48^ ^]^

**Scheme 2:**
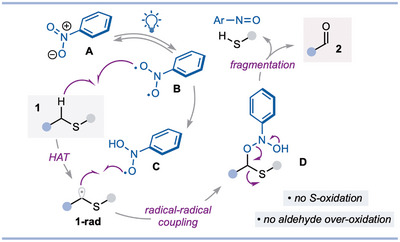
Proposed mechanism for the direct photochemical activation of sulfides.

Herein, we describe the development of a light‐induced anaerobic oxidation of sulfides, which directly targets the alpha carbon, rather than the S‐atom, and results in formal manipulation of the C(sp^3^)─S bond. Crucially, the use of photoexcited nitroarenes allows sulfides **1** to be efficiently converted to aldehydes and ketones (**2**), which can be engaged in situ in transformations that deliver important products such as α‐tertiary amines **3**, alkenes **4**, and secondary alcohols **5** (Scheme 1d). Importantly, over‐oxidation of aldehyde intermediates is not seen. The approach allows sulfides to be considered as latent carbonyl groups—without recourse to the use of multistep sequences for their activation—and streamlines their use as building blocks for synthesis.^[^ ^49^, ^50^, ^51^ ^]^

We first studied the conversion of benzylic sulfide **1a** to benzaldehyde **2a** using a range of nitroarenes under purple light irradiation (Table 1). The nature of the nitroarene had a profound impact on the outcome, with most of the derivatives screened (see Supporting Information) delivering **2a** in <20% yield. Notable exceptions were nitroarenes **A1** and **A2**, which proved to be optimal photo‐oxidants, furnishing **2a** in 70% and 74% yield, respectively (entries 1–3); due to its greater generality, **A1** was selected to explore the process scope (vide infra). Given the fast decay of photoexcited nitroarenes,^[^ ^32^, ^33^, ^34^, ^35^, ^36^, ^37^, ^38^, ^39^, ^40^ ^]^ two equivalents of nitroarene were used to drive conversion. MeCN proved to be the optimal solvent with alternative solvents giving unsatisfactory results (see Supporting Information). Neither adjusting the reaction temperature (entry 5), nor drying and degassing the solvent had a significant effect (see Supporting Information). In contrast, increasing the reaction concentration had a significant negative impact on the yield (entries 6–7). Control experiments confirmed the essential role of the nitroarene and light (entry 8) and lend support to our proposed mechanism. In addition, the radical (**1‐rad**) could be trapped by a radical scavenger (TEMPO), and the adduct detected by HRMS. Also, an analogue of **1a** lacking α C─H bonds did not deliver product and starting material was fully recovered (see Supporting Information). Crucially, over‐oxidation of **2a** to benzoic acid was not observed under our optimized reaction conditions, in stark contrast to the oxidation of alcohols using nitroarenes and light.^[^ ^36^ ^]^

**Table 1:** Optimization of the direct oxidation of sulfide **1a** to benzaldehyde **2a**.

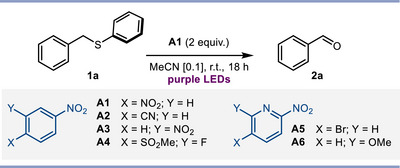			
Entry^a)^	Deviations	Conversion (%)	Yield (%)^b)^
1	None	89	70
2	**A1** (1 equiv.)	88	65
3	**A2** (2 equiv.)	>99	74
4	**A3‐A6**	86–99	16–66
5	0 °C (or 40 °C)	75 (or 95)	54 (or 68)
6	MeCN [0.05]	94	66
7	MeCN [0.2]	>99	44
8	No light at 45 °C (or no **A1)**	<5	n.r.

^a)^ Reaction run on 0.1 mmol scale.

^b)^ Calculated by ^1^H NMR analysis of the crude against CH_2_Br_2_ as internal standard. n.r.: no reaction. Me: methyl.

With optimal conditions in hand, we moved on to the development of sequential protocols for the conversion of sulfides into important product classes. We anticipated that the mild reaction conditions used for the direct oxidation of sulfides to aldehydes would allow the successful implementation of telescoped or one‐pot procedures.

Targeting sequential processes that convert otherwise inert sulfide building blocks into decorated derivatives of relevance to medicinal chemistry,^[^ ^52^, ^53^, ^54^, ^55^, ^56^, ^57^ ^]^ we studied the conversion of benzyl sulfides to α‐tertiary amines **3** (Scheme 3). The telescoped process is compatible with structural and electronic variation of the aromatic ring in the benzyl sulfide, with electron‐neutral (**3a**), electron‐withdrawing (**3b–f**, **3h**), and electron‐donating groups (**3g**, **3j**) tolerated. Sulfides bearing substitution at the *ortho*‐ (**3i**) and *meta*‐position (**3j**), as well as polysubstituted sulfides (**3k–l**), and those containing extended aromaticity (**3m**), also gave products in good yield. The mild reaction conditions proved compatible with the presence of a range of functional groups, primed for further manipulation, such as esters, nitriles, halides, boronic esters, protected alcohols and amines, and even some heteroaromatics (**3n**), which could potentially undergo competing light‐absorption.^[^ ^58^, ^59^ ^]^ Noteworthy, also nonbenzylic primary sulfides can be engaged in light‐mediated anaerobic oxidation and ensuing amino‐allylation. Despite the lower yields of **3o–q**, activation of a nonbenzylic C─H bond, in a sterically demanding environment, using a mild HAT abstractor is striking.

**Scheme 3:**
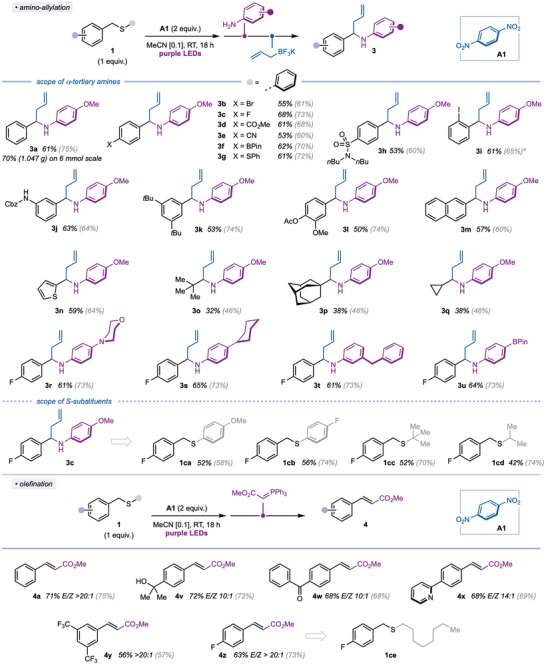
Scope of the light‐induced anaerobic oxidation of benzylic sulfides in sequential amino‐allylation (*top*) and olefination (*bottom*) reactions. Reactions were performed under standard condition on 0.3 mmol scale. Yields are overall isolated yields for the three/two stage processes. ^1^H NMR yield of intermediate aldehydes **2** in parentheses. *^a)^* **A2** (3 equiv.), two Tuna blue Kessil lamps, 5 days without fan. Me: methyl; PinB: boron(pinacolato); Ph: phenyl; *n*Bu: *normal*‐butyl; Cbz: benzyloxycarbonyl; *t*Bu: *tert*‐butyl; Ac: acetyl; Et: ethyl.

Screening the aniline partner, we found that the presence of hetero‐ and carbocyclic motifs (**3r‐s**), alternative substitution patterns (**3t**), and synthetic handles (**3u**) was tolerated. The light‐induced C–H oxidation of sulfides is not limited to phenylsulfide derivatives; electron‐rich (**1ca**) and electron‐poor (**1cb**) aryl S‐substituents deliver **3c** in uniformly high yields. In addition, sulfides bearing S‐alkyl substituents (**1cc‐cd**) underwent sequential transformations, further highlighting the generality of the method. In the case of **1** **cd**, products arising from HAT at the alternative alpha position were not observed, likely due to the difference in BDE for the two alpha C─H bonds.^[^ ^60^ ^]^

To underline the potential of our sulfide oxidation protocol to be used more widely in telescoped processes, we examined a sulfide olefination process (Scheme 3, *bottom*). A subset of sulfides—chosen to further showcase the functional group compatibility of the light‐induced anaerobic sulfide C–H oxidation (i.e., trifluoromethyl, free hydroxyl, aryl ketone, and pyridyl)—was converted to cinnamyl derivatives **4a**, **4v–y** in good overall yield. Regioselective sulfide activation is seen again in the preparation of **4z**; **1ce** undergoes selective HAT activation of a benzylic alpha C─H bond.

Secondary benzylic sulfides can also be engaged in this light‐induced activation. Due to the importance of secondary alcohols and the lack of general methods for the conversion of C─S bonds in otherwise inert sulfides to the corresponding C─OH motifs,^[^ ^61^, ^62^, ^63^, ^64^ ^]^ we developed a one‐pot sequence combining light‐induced anaerobic oxidation with a simple NaBH_4_ reduction (Scheme 4). The method provides the corresponding alcohols in moderate to good overall yields for benzyl sulfides containing electron‐neutral (**5aa**), electron‐poor (**5ab**), and electron‐rich (**5ac**) aromatic rings. More complex molecules derived from amino acids (**5ad**) or blockbuster drugs—for example, fenofibrate (**5ac**) and naproxen (**5ae**)—were also good substrates for the transformation. A sulfide bearing both an aromatic and aliphatic substituent α to sulfur delivered the corresponding alcohol **5af** in good yield, as well as aliphatic secondary sulfides (to give **5ag** and **5ah**), highlighting the opportunity to expand the scope of this transformation beyond α‐bis‐aryl sulfides. As seen with primary and secondary alkyl sulfides (preparation of **3o–q**, **5ag**, and **5ah**), the protocol is currently effective when alkyl substituents without β‐hydrogens or small‐ring cycloalkanes are present. This is likely due to a competing process wherein the intermediate **D** (Scheme 2) undergoes β‐elimination *en route* to a vinyl sulfide species that is prone to decomposition.^[^ ^65^, ^66^ ^]^

**Scheme 4:**
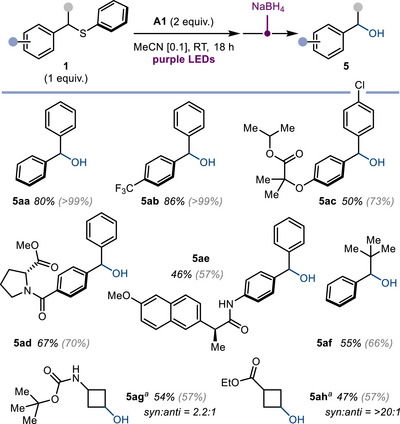
Reaction scope for the formal conversion of secondary sulfides to the corresponding alcohols, using a one‐pot light‐induced anaerobic oxidation / reduction protocol. Reactions were performed under standard conditions on 0.3 mmol scale. Yields are overall isolated yields for the two‐stage process. ^1^H NMR yield of intermediate ketones in parentheses. ^*a)*^ photochemical reaction run for 3 days. Me: methyl; Et: ethyl.

In conclusion, a light‐induced anaerobic C–H oxidation of sulfides, using photoexcited nitrobenzenes, allows their direct and selective conversion to aldehyde and ketone intermediates, thus avoiding multistep sequences proceeding via S‐oxidation and facilitating the use of sulfides as building blocks for synthesis. In the case of primary sulfide activation, no over‐oxidation of aldehyde products is observed; this is in marked contrast to the oxidation of alcohols using photoexcited nitrobenzenes, which delivers carboxylic acids rather than aldehydes. The mild nature of the photo‐induced protocol enables its merger with high‐value transformations in telescoped or one‐pot processes that correspond to the formal manipulation of the C─S bond in sulfides; branched amines, secondary alcohols, and alkenes are accessed by engaging aldehyde and ketone intermediates. The method expands the chemistry of sulfides by diverting reactivity away from sulfur and instead directly targeting the position alpha to it, and thus redefining sulfides as latent synthetic handles to be “switched on” at will.

## Conflict of Interests

The authors declare no conflict of interest.

## Supporting information



Supporting Information

## Data Availability

The data that support the findings of this study are available in the Supporting Information of this article.
