# Supplementary material for: Direct Activation of Sulfides by C–H Oxidation with Photoexcited Nitroarenes: Formal Manipulations of the C─S Bond
Source: Angew Chem Int Ed Engl. 2025 Jun 18;64(33):e202509244. doi: 10.1002/anie.202509244 (PMC12338387; doi:10.1002/anie.202509244)
Supplement: Supplementary file 1 — Supporting Information [file ANIE-64-e202509244-s001.pdf]

Supporting Information

Direct activation of sulfides by C–H oxidation with photoexcited nitroarenes: Formal manipulations of the C–S bond

Valentina D. Cuomo, Ciro Romano, David J. Procter**

Department of Chemistry, University of Manchester, Oxford Road, Manchester, M13 9PL, UK.

E-mail: ciro.romano@manchester.ac.uk; david.j.procter@manchester.ac.uk

Table of contents

1 *General Information* 4

2 *Experimental Procedures* 5

2.1 General Procedures for the Synthesis of Starting Materials 5

2.2 Optimization studies 7

2.3 Investigations into the Reaction Mechanism 12

2.4 General Procedure for Telescoped Aminoallylation – GP6 14

2.5 General Procedure for Telescoped Olefination – GP7 15

2.6 General Procedure for Telescoped Alcohol Formation from Secondary Sulfides – GP8 16

2.7 Gram-Scale Telescoped Aminoallylation 17

2.8 Photochemical Setup 18

2.9 Failed substrates 18

3 *Starting Material Synthesis and Characterization* 19

4 *Substrate Scope of Aminoallylation* 39

5 *Substrate Scope of Olefination* 55

6 *Substrate Scope of Alcohol Formation* 58

7 *NMR Spectra* 63

7.1 NMR Spectra of Starting Materials 63

7.2 NMR Spectra of Aminoallylation Products 101

7.3 NMR Spectra of Olefination Products 127

7.4 NMR Spectra of Alcohol Products 133

8 References 142

1 General Information

All solvents and reagents were used directly without purification unless stated otherwise. All air and moisture sensitive reactions were carried out under nitrogen atmosphere using standard Schlenk manifold techniques. All solvents were purchased as 99.8% purity. ^{1}H NMR spectra were recorded on NMR spectrometers at 400 MHz and 500 MHz and ^{13}C NMR at 101 MHz and 126 MHz. ^{1}H NMR chemical shifts (δ_H) and ^{13}C NMR chemical shifts (δ_C) are quoted in parts per million (ppm) downfield from trimethylsilane (TMS) and coupling constants (J) are quoted in Hertz (Hz). Splitting patterns are assigned s = singlet, d = doublet, t = triplet, q = quartet, p = pentet, h= heptet, dd = doublet of doublets, td = triplet of doublets, dt = doublet of triplets, ddt = doublet of doublet of triplets, m = multiplet, and br. = broad. ^{1}H and ^{13}C Nuclear Magnetic Resonance (NMR) spectra were referenced to $CHCl_3$ (7.26 and 77.2 ppm for ^{1}H and ^{13}C, respectively) and assignments (determined by 2D NMR experiments: NOESY, HSQC and HMBC) were reported when possible and diagnostic. High-resolution mass spectra were obtained using a JEOL JMS-700 spectrometer or a Fissions VG Trio 2000 quadrupole mass spectrometer. Spectra were obtained using chemical ionization (CI) techniques, or positive electrospray (ES). Analytical thin layer chromatography (TLC) was carried out using aluminum backed plates pre-coated (0.25 mm) with Merck Silica Gel 60 F254. Compounds were visualized by exposure to UV-light (254 nm) or by dipping the plates in permanganate ($KMnO_4$) stain or ninhydrin followed by heating. Column chromatography was performed using Merck Silica Gel 60 (40–63 μm). All mixed solvent eluents are reported as v/v solutions. The light sources employed are light-emitting diodes (LEDs) Kessil PR 160 390 nm and A160WE tuna blue lamp. All the reactions were conducted in CEM 10 mL glass microwave tubes.

2 Experimental Procedures

2.1 General Procedures for the Synthesis of Starting Materials

*General Procedure for the Preparation of Sulfides from Bromides – **GP1***

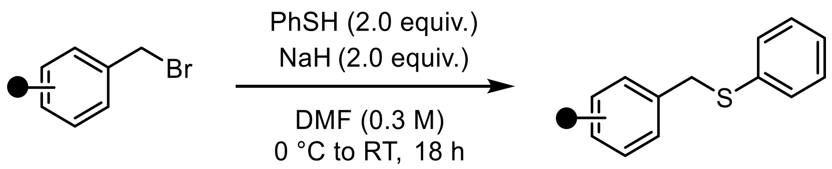

1

In a two-necked flask dried under vacuum and equipped with a stirring bar, sodium hydride (2.0 equiv.) was slowly added in portions to a solution of thiophenol (2.0 equiv.) in DMF (0.3 M) at 0 °C, under nitrogen. The reaction was stirred for 1 h and allowed to reach room temperature. Then, bromide (1.0 equiv.) was added and the reaction was monitored by TLC (18 h). The reaction was quenched by the addition of water (30 mL) and extracted with CH_2Cl_2 (3 x 30 mL) and Na_2CO_3 (30 mL) to remove excess of thiophenol. The combined organic phases were washed with brine (30 mL) and dried over $MgSO_4$. The solvents were removed under reduced pressure and the crude product was purified by column chromatography.

*General Procedure for the Preparation of Sulfides from Bromides – **GP2***

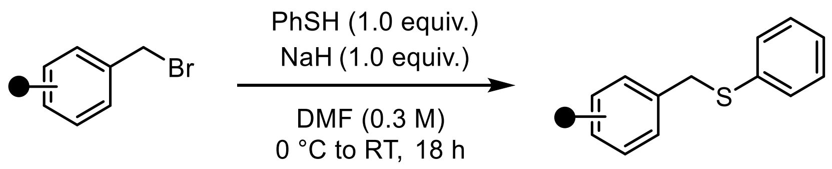

1

In a two-necked flask dried under vacuum and equipped with a stirring bar, sodium hydride (1.0 equiv.) was slowly added in portions to a solution of thiophenol (1.0 equiv.) in DMF (0.3 M) at 0 °C, under nitrogen. The reaction was stirred for 1 h and allowed to reach room temperature. Then, bromide (2.0 equiv.) was added and the reaction was monitored by TLC (18 h). The reaction was quenched by the addition of water (30 mL) and extracted with CH_2Cl_2 (3 x 30 mL) and Na_2CO_3 (30 mL) to remove excess of thiophenol. The combined organic phases were washed with brine (30 mL)

and dried over $MgSO_4$. The solvents were removed under reduced pressure and the crude product was purified by column chromatography.

*General Procedure for the Preparation of Sulfides from Primary Alcohols – **GP3***

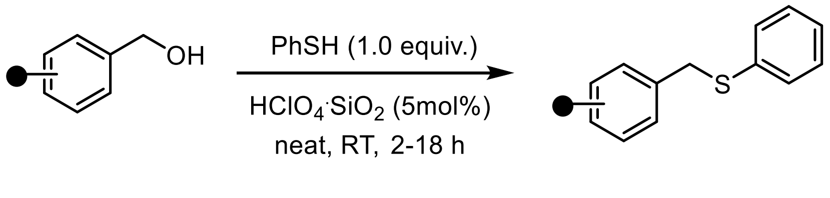

1

In a one-necked round-bottomed flask, equipped with a stirring bar, thiophenol (1.0 equiv.), alcohol (1.0 equiv.), and $HClO_4$-SiO_2 catalyst (5 mol% - prepared according to a literature procedure[1]) were added at room temperature. The reaction mixture was stirred at room temperature until completion (monitored by TLC). The reaction was diluted with EtOAc (30 mL), the silica was filtered off, and the solvent was removed under reduced pressure. The crude product was purified by column chromatography.

*General Procedure for the Preparation of Sulfides from Primary and Secondary Alcohols – **GP4***

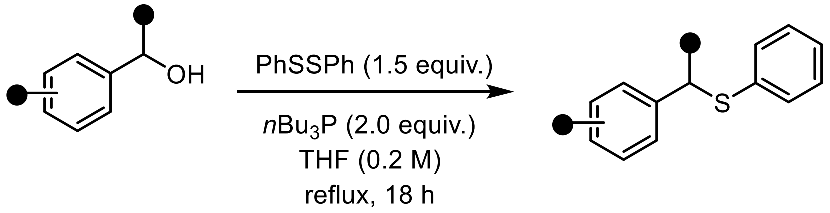

1

In a schlenk flask dried under vacuum and equipped with a stirring bar, tributylphosphine (2.0 equiv.) was added dropwise to a solution of diphenyl sulfide (1.5 equiv.) and alcohol (1.0 equiv.) in THF (0.2 M) at room temperature. The reaction mixture was stirred at reflux for 18 h. The reaction was quenched by the addition of 2N aq. NaOH (30 mL) and extracted with Et_2O (3 x 30 mL). The combined organic phases were washed with brine (30 mL) and dried over $MgSO_4$. The solvents were removed under reduced pressure and the crude product was purified by column chromatography.

*General Procedure for the Preparation of Sulfides from Secondary Alcohols – **GP5***

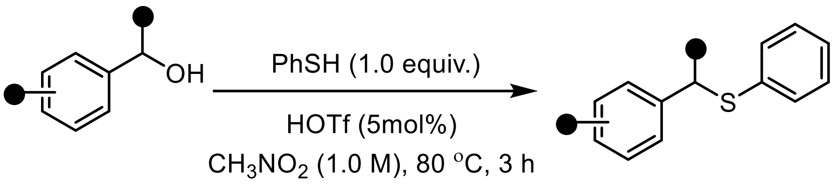

1

In a one-necked round-bottomed flask, equipped with a stirring bar, thiophenol (1.0 equiv.), alcohol (1.0 equiv.), CH_3NO_2 (1.0 M) and HOTf (5 mol%) were added at room temperature. The reaction mixture was stirred at 80 °C until completion (monitored by TLC). The reaction was diluted with EtOAc (30 mL), washed with brine, dried over $MgSO_4$ and the solvent was removed under reduced pressure. The crude product was purified by column chromatography.

2.2 Optimization studies

Solvent screening

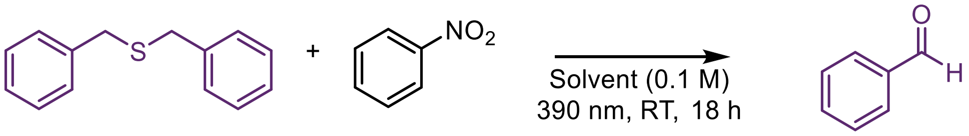

S0 1.0 equiv. **A5** 2.0 equiv. **2a**

Entry	***Solvent***	***Conversion (%)[a]***	***Product (%)[a]***
1	CD_2Cl_2	77	28
2	EtOAc	80	30
3	DCE	n.d.	traces
4	THF	n.d.	traces
5	Benzene	n.d.	traces
6	EtOH	n.d.	n.r.
7	$(CH_3)_3CCN$	>99	36
8	DMSO	n.d.	19
9	PhF	89	26
10	CD_3CN	>99	**40**

Table S1. Reactions run on 0.1 mmol scale. [a] Calculated by ^{1}H-NMR analysis of the crude without evaporating the solvent, using CH_2Br_2 or 1,1',2,2'-tetrachloroethane as internal standard. n.d. not detected. n.r., no reaction.

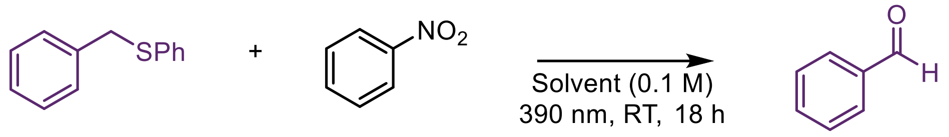

S0 1.0 equiv. **A5** 2.0 equiv. **2a**

Entry	***Solvent***	***Conversion (%)***[a]	***Product (%)***[a]
1	CH_3CN	>99	32
2	dioxane	n.d.	traces
3	HFIP	n.d.	traces
4	DME	n.d.	16
5	DMA	n.d.	8
6	PhCN	n.d.	26
7	$CH_3(CH_2)_2CN$	n.d.	32
8	*i*Pr acetate	n.d.	traces
9	*o*-dichlorobenzene	>99	44
10	dimethyl carbonate	n.d.	18
11	acetone	>99	26
12	$CHCl_3$	>99	22
13	cyclohexane	>99	10
14	DMF	15	22
15	Pyridine	>99	traces
16	PhCl	>99	20
17	$PhCF_3$	>99	42
18	4-chlorobenzo trifluoride	>99	16

Table S2. Reactions run on 0.1 mmol scale. [a] Calculated by ^{1}H-NMR analysis of the crude without evaporating the solvent, using CH_2Br_2 or 1,1',2,2'-tetrachloroethane as internal standard. n.d. not detected.

CH_3CN handling

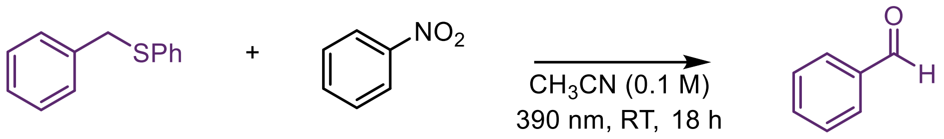

1a 1.0 equiv. **A5** 2.0 equiv. **2a**

Entry	***Solvent***	***Conversion (%)[a]***	***Product (%)[a]***
1	dry CH_3CN	>99	34
2	not dry CH_3CN	>99	30
3	dry CH_3CN sparged with N_2 for 5 min	>99	29

Table S3. Reactions run on 0.1 mmol scale. [a] Calculated by ^{1}H-NMR analysis of the crude without evaporating the solvent, using CH_2Br_2 or 1,1',2,2'-tetrachloroethane as internal standard.

Nitroarene screening

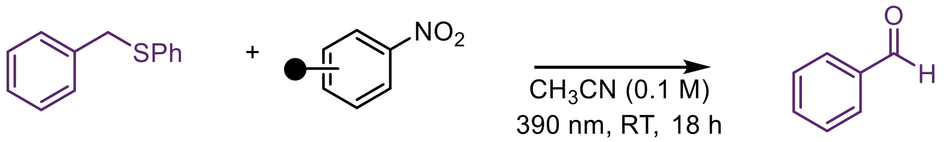

1a 1.0 equiv. **A** 2.0 equiv. **2a**

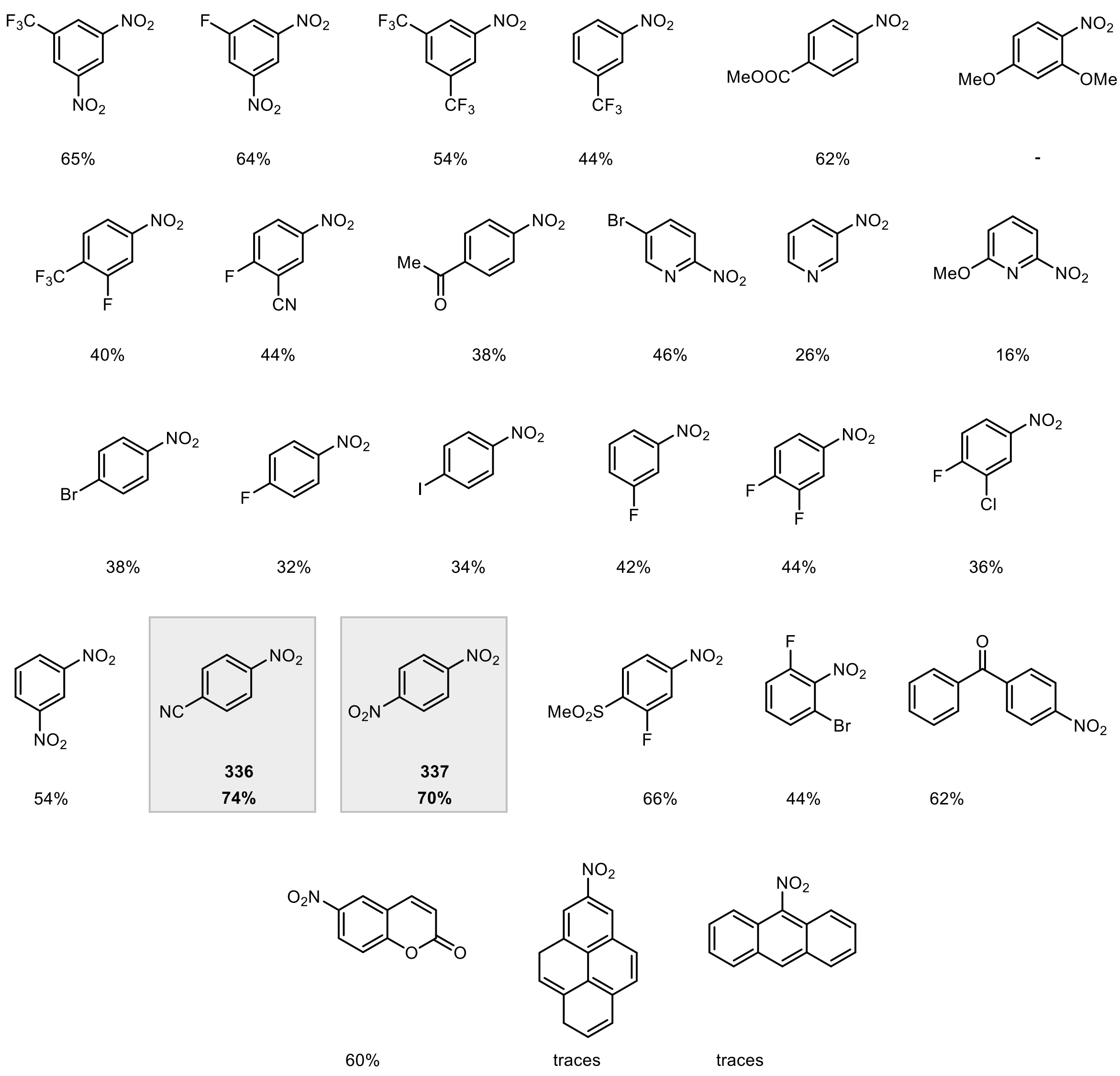

Figure S1. Reactions run on 0.1 mmol scale. Calculated by ^{1}H-NMR analysis of the crude without evaporating the solvent, using CH_2Br_2 or 1,1',2,2'-tetrachloroethane as internal standard.

Temperature screening

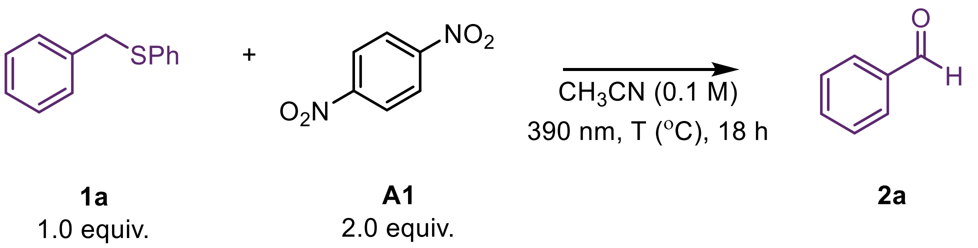

Entry	T (°C)	$ArNO_2$[a] recovered (%)	Conversion (%)[a]	Product (%)[a]
**1**	0	35	75	54
**2**	40	38	95	68

Table S4. Reactions run on 0.1 mmol scale. [a] Calculated by ^{1}H-NMR analysis of the crude without evaporating the solvent, using CH_2Br_2 or 1,1',2,2'-tetrachloroethane as internal standard.

Concentration screening

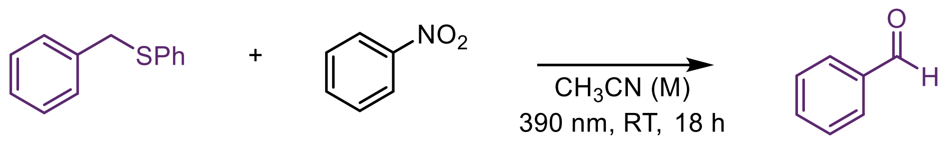

1a
1.0 equiv.

A5
2.0 equiv.

2a

Entry	SM (equiv.)	XX (equiv.)	Molarity (M)	Conversion (%)[a]	Product (%)[a]
**1**	1	2	0.1	>99	32
**2**	1	2	0.2	90	46
**3**	1	2	0.05	>99	20
**4**	1 (0.2 mmol)	2	0.2	>99	50
**5**	1	1	0.1	92	**52**
**6**	1	1	0.2	>99	26
**7**	1 (0.2 mmol)	1	0.2	>99	44
**8**	2	1	0.1	40	16

Table S5. Reactions run on 0.1 mmol scale. [a] Calculated by ^{1}H-NMR analysis of the crude without evaporating the solvent, using CH_2Br_2 or 1,1',2,2'-tetrachloroethane as internal standard.

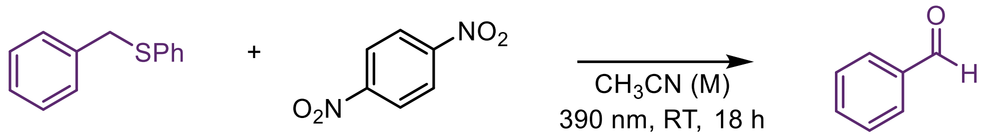

1a
1.0 equiv.

A1
2.0 equiv.

2a

Entry	SM (equiv.)	XX (equiv.)	Molarity (M)	$ArNO_2$ (%)	Conversion (%)[a]	Product (%)[a]
**1**	1	1	0.1	90	90	65
**2**	1	2	0.05	35	94	66
**3**	1	2	0.2	>99	>99	44
**4**	1[b]	2	0.1	37	>99	70

Table S6. Reactions run on 0.1 mmol scale. [a] Calculated by ^{1}H-NMR analysis of the crude without evaporating the solvent, using CH_2Br_2 or 1,1',2,2'-tetrachloroethane as internal standard. [b] Reaction run on 0.25 mmol scale.

Control experiments

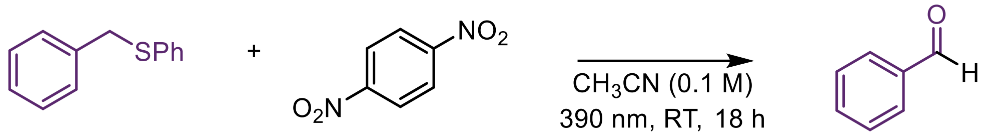

1a 1.0 equiv. **A1** 2.0 equiv. **2a**

Entry	*Deviations*	*ArNO₂ (%)*	*Conversion (%)[a]*	*Product (%)[a]*
1	no light	>99	<5	n.r.
2	no A1	–	<5	n.r.

Table S7. Reactions run on 0.1 mmol scale. [a] Calculated by ^{1}H-NMR analysis of the crude without evaporating the solvent, using CH_2Br_2 or 1,1',2,2'-tetrachloroethane as internal standard. n.r., no reaction.

2.3 Investigations into the Reaction Mechanism

TEMPO Trapping Experiment

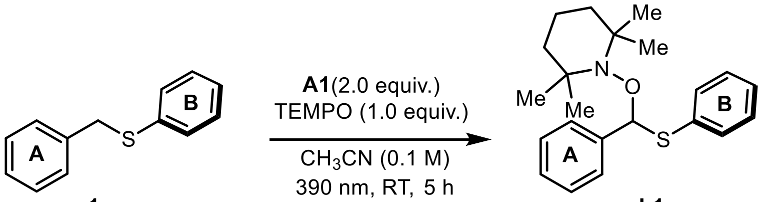

1 (1 equiv.) **I-1**

detected by HRMS
Mass exp. 356.2043
Mass obs. 356.2054

The reaction was run according to the General Procedure for the photochemical step. An oven-dried microwave vial, equipped with a stirring bar, was charged with sulfide **1** (20 mg, 0.1 mmol, 1.0 equiv.), 1,4-dinitrobenzene **A1** (34 mg, 0.2 mmol, 2.0 equiv.) and TEMPO (16 mg, 0.1 mmol, 1.0 equiv.). The tube was then sealed with an aluminum crimp cap with a septum, evacuated under high vacuum and backfilled with nitrogen (x 3 times). The reagents were dissolved in anhydrous CH_3CN (1.0 mL, 0.1 M). The reaction mixture was degassed by bubbling nitrogen through the solution for 2 minutes. The reaction mixture was irradiated with purple LEDs and stirred at room temperature for 5 hours whilst being cooled with a fan. The TEMPO adduct product **I-1** was detected by HRMS. HRMS (APCI): Calculated for $C_{22}H_{30}NOS$ $(M-H)^+$, 356.2043; found 356.2054.

This experiment suggests the formation of a radical α to sulfur, through a HAT process.

HAT vs Oxidation at Sulfur

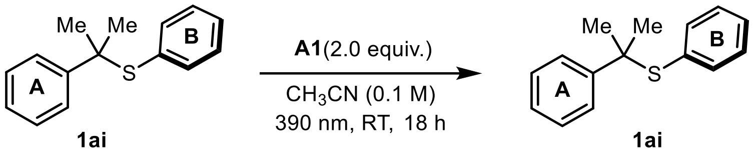

1ai (1 equiv.) → **1ai** 93%

Phenyl(2-phenylpropan-2-yl)sulfide **1ai** was prepared according to a literature procedure[2] (1.06 g, 93%) as a colorless liquid, after purification by column chromatography on silica gel (hexane 100%). ^{1}H NMR (400 MHz, $CDCl_3$) δ 7.45 – 7.38 (m, 2H, Ar_AC*H*), 7.31 – 7.09 (m, 8H, Ar_AC*H* and Ar_BC*H*), 1.69 (s, 6H, 2 x C*H*$_3$); ^{13}C NMR (101 MHz, $CDCl_3$) δ 146.5 (Ar_B*C*), 136.6 (Ar_BCH), 132.9 (Ar_A*C*), 128.6 (Ar_B*C*H), 128.4 (Ar_B*C*H), 128.0 (Ar_A*C*H), 126.7 (Ar_A*C*H), 126.6 (Ar_A*C*H), 51.1 (S*C*(CH_3)$_2$), 29.8 (2 x *C*H$_3$). Data in accordance with the literature.[2]

The reaction was run according to the General Procedure for the photochemical step. An oven-dried microwave vial, equipped with a stirring bar, was charged with sulfide **1ai** (23 mg, 0.1 mmol, 1.0 equiv.) and 1,4-dinitrobenzene **A1** (34 mg, 0.2 mmol, 2.0 equiv.). The tube was then sealed with an aluminum crimp cap with a septum, evacuated under high vacuum and backfilled with nitrogen (x 3 times). The reagents were dissolved in anhydrous CH_3CN (1.0 mL, 0.1 M). The reaction mixture was degassed by bubbling nitrogen through the solution for 2 minutes. The reaction mixture was irradiated with purple LEDs and stirred at room temperature for 18 hours whilst being cooled with a fan. Internal standard – dibromo-methane (0.1 mmol, 7 μL) – was added to the mixture and an aliquot was diluted with $CDCl_3$ for ^{1}H-NMR analysis. Only recovery of starting material **1ai** in 93% yield was observed.

This experiment rules out the intermediacy of a benzylic carbocation intermediate and lends support to our reaction proceeding via HAT from the α C–H bond.

2.4 General Procedure for Telescoped Aminoallylation – **GP6**

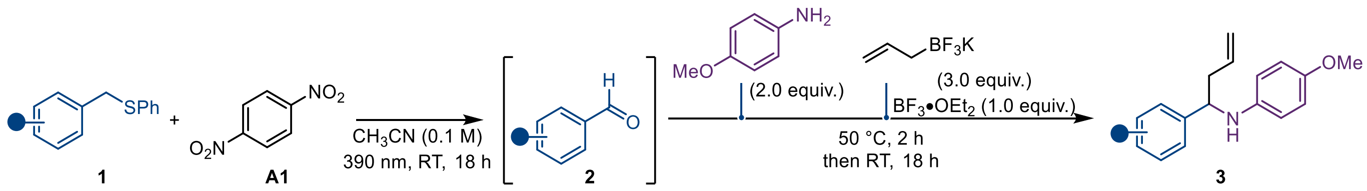

Photochemical step: An oven-dried microwave vial, equipped with a stirring bar, was charged with sulfide **1** (0.3 mmol, 1.0 equiv.) and 1,4-dinitrobenzene **A1** (101 mg, 0.6 mmol, 2.0 equiv.). The tube was then sealed with an aluminum crimp cap with a septum, evacuated under high vacuum and backfilled with nitrogen (x 3 times). The reagents were dissolved in anhydrous CH_3CN (3.0 mL, 0.1 M). The reaction mixture was degassed by bubbling nitrogen through the solution for 2 minutes. The reaction mixture was irradiated with purple LEDs and stirred at room temperature for 16 hours whilst being cooled with a fan. When the reaction is carried out on 0.1 mmol scale, internal standard – dibromo-methane (0.3 mmol, 21 μL) or 1,1,2,2-tetrachloroethane (0.3 mmol, 30 μL) – was added to the mixture and an aliquot was diluted with $CDCl_3$ for ^{1}H-NMR analysis to calculate the yield of **2**.

Aminoallylation step: After completion of the photochemical reaction, the LEDs were switched off and the mixture was transferred into another oven-dried vial, equipped with a stirring bar, and charged with *para*-methoxy aniline (74 mg, 0.6 mmol, 2.0 equiv.) and molecular sieves. The initial vial was rinsed with 0.3 mL of anhydrous CH_3CN and the washings were added to the new vial. The second vial was sealed with an aluminum crimp cap with a septum, evacuated under high vacuum and backfilled with nitrogen (x 3 times), and then stirred at 50 °C for 2-16 h. After this time, the mixture was transferred into a third oven-dried microwave vial, equipped with a stirring bar, which was charged with potassium allyl trifluoroborate (133 mg, 0.9 mmol, 3.0 equiv.) and sealed with an aluminum crimp cap with a septum. After evacuation under high vacuum and backfilling with nitrogen (x 3 times), boron trifluoride diethyl etherate (ca 48-50% BF_3) (55 μL, 0.3 mmol, 1.0 equiv.) was added to the mixture while stirring at room temperature, and the mixture was stirred at this temperature for 16 h. The tube was opened, and the mixture was treated with aqueous NaOH 25 M until pH 8-9 and extracted with CH_2Cl_2 (5 mL). The layers were separated, andthe aqueous layer was extracted with CH_2Cl_2 (2

× 5 mL). The combined organic layers were washed with brine (3 mL), dried over $MgSO_4$, filtered and evaporated under reduced pressure. Internal standard – dibromo-methane (0.3 mmol, 21 μL) or 1,1,2,2-tetrachloroethane (0.3 mmol, 30 μL) – was added to the mixture and an aliquot was diluted with $CDCl_3$ for ^{1}H-NMR analysis. The crude was then purified by column chromatography on deactivated silica gel (1% Et_3N added to the first volume of the solvent system used).

2.5 General Procedure for Telescoped Olefination – **GP7**

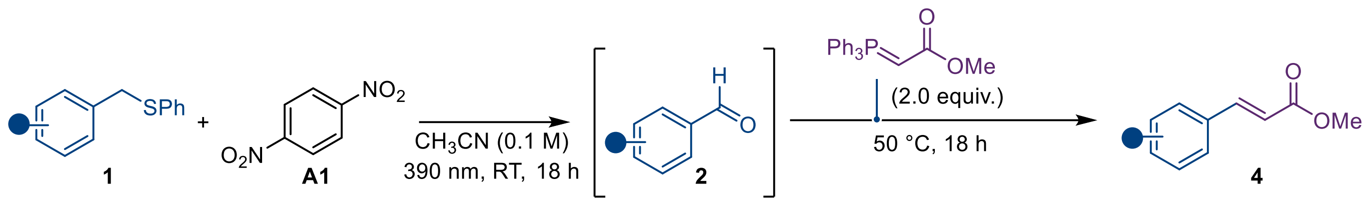

Photochemical step: An oven-dried vial, equipped with a stirring bar, was charged with sulfide **1** (0.3 mmol, 1.0 equiv.) and 1,4-dinitrobenzene **A1** (101 mg, 0.6 mmol, 2.0 equiv.). The tube was then sealed with an aluminum crimp cap with a septum, evacuated under high vacuum and backfilled with nitrogen (x 3 times). The reagentswere dissolved in anhydrous CH_3CN (3.0 mL, 0.1 M). The reaction mixture was degassed by bubbling nitrogen through the solution for 2 minutes. The reaction mixture was irradiated with purple LEDs and stirred at room temperature for 16 hours whilst being cooled with a fan. When the reaction is carried out on 0.1 mmol scale, internal standard – dibromo-methane (0.3 mmol, 21 μL) or 1,1,2,2-tetrachloroethane (0.3 mmol, 30 μL) – was added to the mixture and an aliquot was diluted with $CDCl_3$ for ^{1}H-NMR analysis to calculate the yield of **2**.

Olefination step: After completion of the photochemical reaction, the LEDs were switched off and the mixture was transferred to another oven-dried vial, equipped with a stirring bar, and charged with methyl (triphenylphosphoranylidene)acetate (201 mg, 0.6 mmol, 2.0 equiv.). The initial vial was rinsed with 0.3 mL of anhydrous CH_3CN and the washings were added to the new vial after the second vial was sealed with an aluminum crimp cap with septum, evacuated under high vacuum and backfilled with nitrogen atmosphere (x 3 times). The resulting mixture was stirred at 50 °C for 18 h, then

evaporated under reduced pressure. 1,1,2,2-Tetrachloroethane (0.3 mmol, 30 μL) was added as an internal standard and an aliquot was dissolved in $CDCl_3$ for ^1H NMR analysis. The crude was then purified by column chromatography on silica gel.

2.6 General Procedure for Telescoped Alcohol Formation from Secondary Sulfides – **GP8**

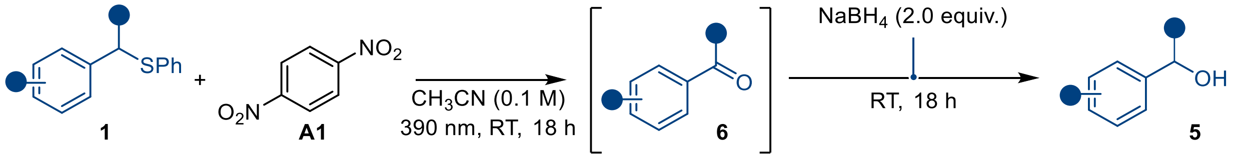

Photochemical step: An oven-dried vial, equipped with a stirring bar, was charged with secondary sulfide **1** (0.3 mmol, 1.0 equiv.) and 1,4-dinitrobenzene **A1** (101 mg, 0.6 mmol, 2.0 equiv.). The tube was then sealed with an aluminum crimp cap with a septum, evacuated under high vacuum and backfilled with nitrogen (x 3 times). The reagents were dissolved in anhydrous CH_3CN (3.0 mL, 0.1 M). The reaction mixture was degassed by bubbling nitrogen through the solution for 2 minutes. The reaction mixture was irradiated with purple LEDs and stirred at room temperature for 16 hours whilst being cooled with a fan. When the reaction is carried out on 0.1 mmol scale, internal standard – dibromo-methane (0.3 mmol, 21 μL) or 1,1,2,2-tetrachloroethane (0.3 mmol, 30 μL) – was added to the mixture and an aliquot was diluted with $CDCl_3$ for ^1H-NMR analysis to calculate the yield of **6**.

Reduction step: After completion of the photochemical reaction, the LEDs were switched off and $NaBH_4$ (23 mg, 0.6 mmol, 2.0 equiv.) was added to the mixture which was then stirred at RT for 18 h. The tube was opened, and the mixture was treated with aqueous saturated $NaHCO_3$ until pH 7 and extracted with EtOAc (5 mL). The layers were separated, and the aqueous layer was extracted with EtOAc (2 × 5 mL). The combined organic layers were washed with brine (3 mL), dried over $MgSO_4$, filtered and evaporated under reduced pressure. Internal standard – dibromo-methane (0.3 mmol, 21 μL) or 1,1,2,2-tetrachloroethane (0.3 mmol, 30 μL) – was added to the mixture and an aliquot was diluted with $CDCl_3$ for ^1H-NMR analysis. The crude was then purified by column chromatography.

2.7 Gram-Scale Telescoped Aminoallylation

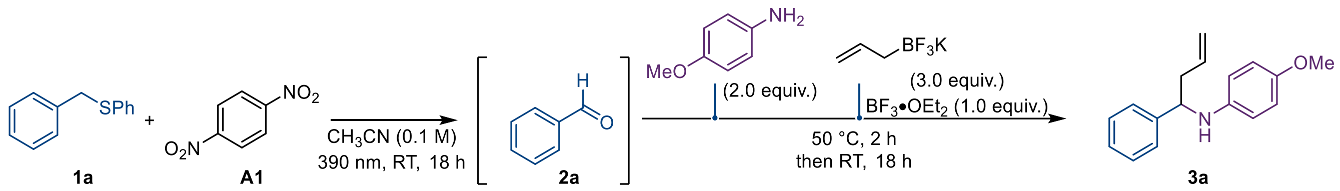

Photochemical step: An oven-dried round-bottom flask, equipped with a stirring bar, was charged with benzylphenyl sulfide **1a** (6.0 mmol, 1.0 equiv.) and 1,4-dinitrobenzene **A1** (2.01 g, 12.0 mmol, 2.0 equiv.). The flask was then evacuated under high vacuum and backfilled with nitrogen (x 3 times). The reagents were dissolved in anhydrous CH_3CN (60 mL, 0.1 M). The reaction mixture was irradiated with purple LEDs and stirred at room temperature for 16 hours whilst being cooled with a fan.

Aminoallylation step: After completion of the photochemical reaction, the LEDs were switched off and *para*-methoxy aniline (1.48 g, 12.0 mmol, 2.0 equiv.) and molecular sieves were added to the mixture which was then stirred at 50 °C for 16 h. After this time, the mixture was charged with potassium allyl trifluoroborate (2.66 g, 18.0 mmol, 3.0 equiv.) and boron trifluoride diethyl etherate (ca 48-50% BF_3) (1.1 mL, 6.0 mmol, 1.0 equiv.) was added to the mixture while stirring at room temperature, and the mixture was stirred at this temperature for 16 h. The flask was opened, and the mixture was treated with aqueous NaOH 25 M until pH 8-9 and extracted with CH_2Cl_2 (100 mL). The layers were separated, andthe aqueous layer was extracted with CH_2Cl_2 (2 × 100 mL). The combined organic layers were washed with brine (60 mL), dried over $MgSO_4$, filtered and evaporated under reduced pressure. The crude was purified by column chromatography on deactivated silica gel (1% Et_3N added to the first volume of the solvent system used and then toluene/hexane 9:1) affording **3a** (1.05 g, 70%) as a liquid.

2.8 Photochemical Setup

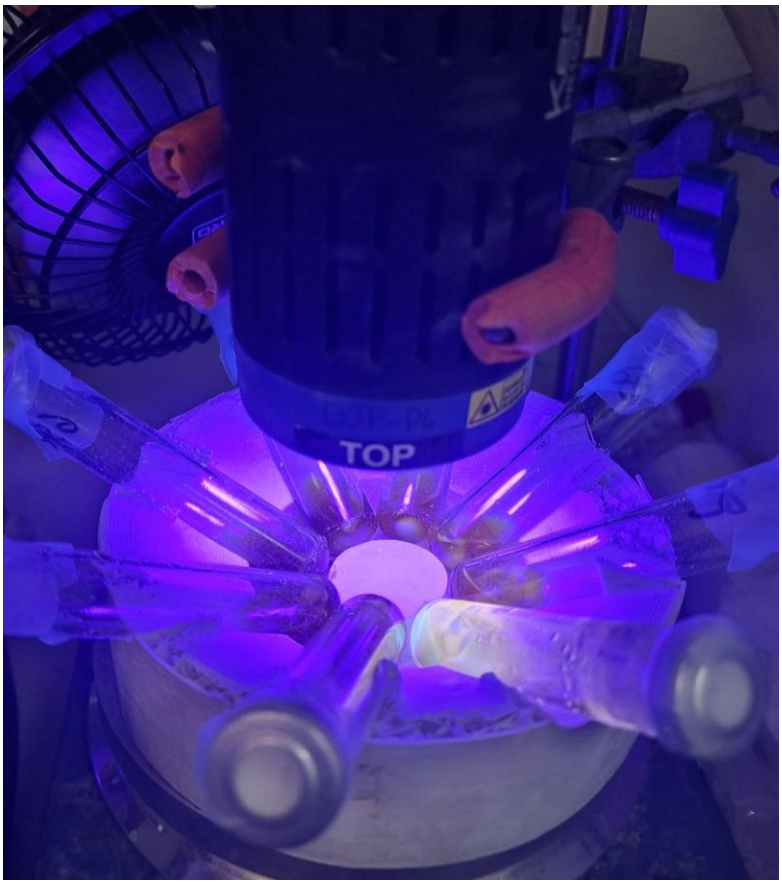

Figure S2. Photochemical setup for the anaerobic oxidation of sulfides to aldehydes/ketones. The Kessil lamp is kept at 5 cm distance from the carousel with the reaction vials.

2.9 Failed substrates

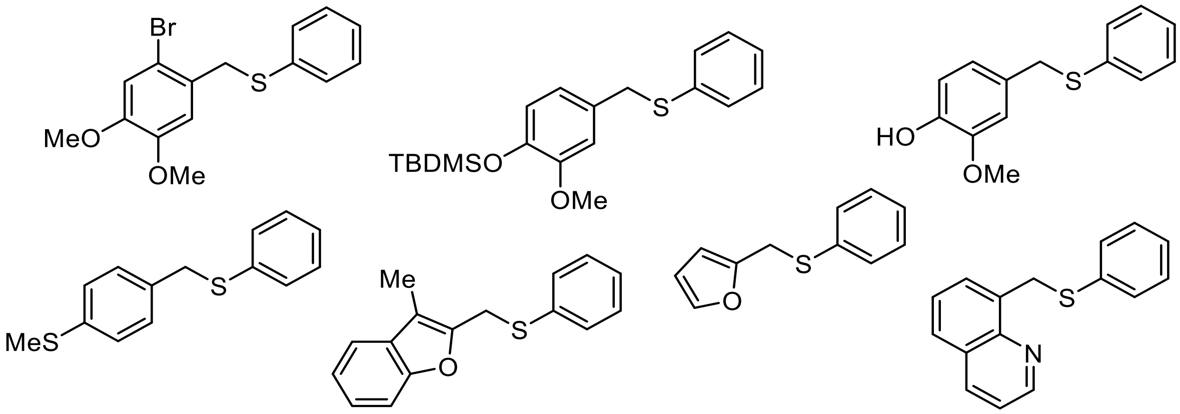

Figure S3. Substrates which displayed no reaction or afforded low yields of aldehyde product.

3 Starting Material Synthesis and Characterization

Phenyl *p*-bromobenzyl sulfide 1b

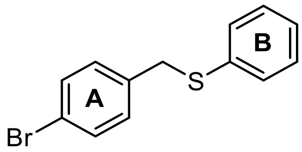

Following **GP2**, *1-bromo-4-(bromomethyl)benzene* (1.25 g, 5.00 mmol) afforded **1b** (500 mg, 72%) as a white solid, after purification by column chromatography on silica gel (hexane/EtOAc 100:0 to 100:2). ^{1}H NMR (500 MHz, $CDCl_3$) δ 7.41 – 7.34 (m, 2H, Ar_AC*H*), 7.31 – 7.20 (m, 5H, Ar_BC*H*), 7.20 – 7.09 (m, 2H, Ar_AC*H*), 4.03 (s, 2H, C*H*$_2$); ^{13}C NMR (126 MHz, $CDCl_3$) δ 136.8 (Ar_A*C*), 135.7 (Ar_B*C*), 131.7 (Ar_A*C*H), 130.6 (Ar_A*C*H), 130.3 (Ar_B*C*H), 129.0 (Ar_B*C*H), 126.8 (Ar_B*C*H), 121.1 (Ar_A*C*), 38.7 (*C*H_2). Data in accordance with the literature.[3]

(4-Fluorobenzyl)(phenyl)sulfide 1c

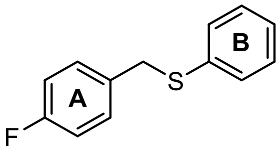

Following **GP1**, *4-(bromomethyl)-4-fluorobenzene* (623 μL, 5.00 mmol) afforded **1c** (1.01 g, 93%) as a white solid, after purification by column chromatography on silica gel (hexane 100%). ^{1}H NMR (400 MHz, $CDCl_3$) δ 7.35 – 7.15 (m, 7H, Ar_AC*H* and Ar_BC*H*), 7.01 – 6.91 (m, 2H, Ar_AC*H*), 4.08 (s, 2H, C*H*$_2$); ^{13}C NMR (101 MHz, $CDCl_3$) δ 162.1 (d, J = 245 Hz, Ar*C*F), 136.0 (Ar_B*C*), 133.4 (d, *J* = 3.0 Hz, Ar_A*C*), 130.49 (d, *J* = 8.0 Hz, Ar_A*C*H), 130.34 (Ar_B*C*H), 129.03 (Ar_B*C*H), 126.73 (Ar_B*C*H), 115.46 (d, *J* = 21.5 Hz, Ar_ACH), 38.6 (*C*H_2). Data in accordance with the literature.[4]

Methyl 4-((phenylthio)methyl)benzoate 1d

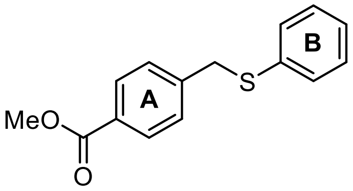

Following **GP1**, *methyl 4-(bromomethyl)benzoate* (573 mg, 2.50 mmol) afforded **1d** (561 mg, 87%) as a white solid, after purification by column chromatography on silica gel (hexane/EtOAc 100:0 to 9:1). ^{1}H NMR (500 MHz, $CDCl_3$) δ 7.93 (dd, *J* = 8.3, 2.0 Hz, 2H, $Ar_AC\textit{H}$), 7.33 – 7.14 (m, 7H, $Ar_BC\textit{H}$ and $Ar_AC\textit{H}$), 4.11 (s, 2H, $C\textit{H}_2$), 3.89 (s, 3H, $C\textit{H}_3$); ^{13}C NMR (126 MHz, $CDCl_3$) δ 167.0 (*C*O), 143.2 ($Ar_A\textit{C}$), 135.5 ($Ar_A\textit{C}$), 130.7 ($Ar_B\textit{C}H$), 129.9 ($Ar_A\textit{C}H$), 129.1 ($Ar_B\textit{C}$), 129.1 ($Ar_A\textit{C}H$), 129.0 ($Ar_B\textit{C}H$), 127.0 ($Ar_B\textit{C}H$), 52.2 ($\textit{C}H_3$), 39.2 ($\textit{C}H_2$). Data in accordance with the literature.[5]

4-((Phenylthio)methyl)benzonitrile 1e

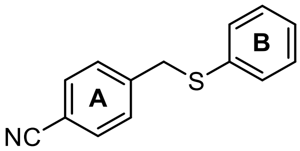

Following **GP1**, *4-(bromomethyl)benzonitrile* (980 mg, 5 mmol) afforded **1e** (998 mg, 89%) as a white solid, after purification by column chromatography on silica gel (hexane/EtOAc 100:0 to 9:1). ^{1}H NMR (400 MHz, $CDCl_3$) δ 7.56 – 7.53 (d, J = 7.9, 2H, $Ar_AC\textit{H}$), 7.35 – 7.31 (d, J = 7.9, 2H, $Ar_AC\textit{H}$), 7.29 – 7.18 (m, 5H, $Ar_BC\textit{H}$), 4.10 (s, 2H, $C\textit{H}_2$); ^{13}C NMR (101 MHz, $CDCl_3$) δ 143.5 ($Ar_A\textit{C}CH_2$), 134.8 ($Ar_B\textit{C}$), 132.4 ($Ar_A\textit{C}H$), 131.0 ($Ar_B\textit{C}H$), 129.6 ($Ar_A\textit{C}H$), 129.2 ($Ar_B\textit{C}H$), 127.3 ($Ar_B\textit{C}H$), 118.9 ($Ar_A\textit{C}$), 111.1 (*C*N), 39.3 ($\textit{C}H_2$). Data in accordance with the literature.[5]

4,4,5,5-Tetramethyl-2-(4-((phenylthio)methyl)phenyl)-1,3,2-dioxaborolane 1f

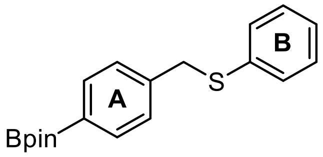

Following **GP1**, *2-(4-(bromomethyl)phenyl)-4,4,5,5-tetramethyl-1,3,2-dioxaborolane* (743 mg, 2.50 mmol) afforded **1f** (631 mg, 77%) as a white solid, after purification by column chromatography on silica gel (hexane/EtOAc 100:0 to 9:1). ^{1}H NMR (400 MHz, $CDCl_3$) δ 7.76 – 7.68 (m, 2H, $Ar_AC\textit{H}$), 7.33 – 7.13 (m, 7H, $Ar_AC\textit{H}$ and $Ar_BC\textit{H}$), 4.11 (s, 2H, $C\textit{H}_2$), 1.34 (s, 12H, $C\textit{H}_3$); ^{13}C NMR (101 MHz, $CDCl_3$) δ 140.9 ($Ar_A\textit{C}CH_2$), 136.3 ($Ar_A\textit{C}B$), 135.1 ($Ar_A\textit{C}H$), 130.1 ($Ar_B\textit{C}H$), 129.0 ($Ar_B\textit{C}H$ and $Ar_B\textit{C}$), 128.3 ($Ar_A\textit{C}H$), 126.5 ($Ar_B\textit{C}H$), 83.9 (2 x $\textit{C}CH_3$), 39.3 ($\textit{C}H_2$), 25.0 (4 x $\textit{C}H_3$). Data in accordance with the literature.[6]

Phenyl(4-(phenylthio)benzyl)sulfide 1g

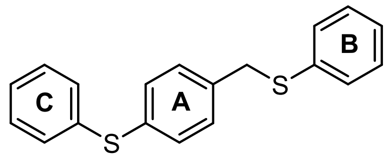

Following **GP3**, *(4-(phenylthio)phenyl)methanol* (707 mg, 3.24 mmol) afforded **1g** (260 mg, 26%) as a liquid, after purification by column chromatography on silica gel (hexane/EtOAc 100:0 to 200:3). ^{1}H NMR (400 MHz, $CDCl_3$) δ 7.37 – 7.19 (m, 14H, $Ar_AC\textit{H}$, $Ar_BC\textit{H}$ and $Ar_CC\textit{H}$), 4.12 (s, 2H, $C\textit{H}_2$); ^{13}C NMR (101 MHz, $CDCl_3$) δ 136.7 (Ar*C*), 136.0 (Ar*C*), 135.9 (Ar*C*), 134.6 (Ar*C*), 131.3 (Ar*C*H), 131.0 (Ar*C*H), 130.3 (Ar*C*H), 129.8 (Ar*C*H), 129.3 (Ar*C*H), 129.0 (Ar*C*H), 127.1 (Ar*C*H), 126.7 (Ar*C*H), 38.9 ($\textit{C}H_2$). HRMS (APCI): Calculated for $C_{19}H_{15}S_2$ $(M-H)^-$, 307.0621; found 307.0621.

4-((Phenylthio)methyl)-*N,N*-dipropylbenzenesulfonamide 1h

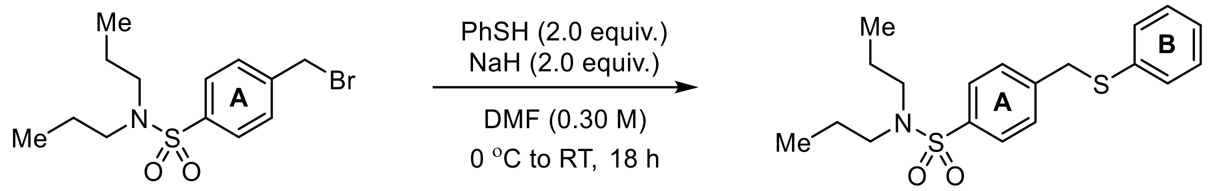

Following **GP1**, *4-(bromomethyl)-N,N-dipropylbenzenesulfonamide* (669 mg, 2.00 mmol) – synthesized following a literature procedure[7]– afforded **1h** (399 mg, 55%) as a white solid (m.p. 80 – 82 °C), after purification by column chromatography on silica gel (hexane/EtOAc 100:0 to 1:1). ^{1}H NMR (400 MHz, $CDCl_3$) δ 7.73 – 7.65 (m, 2H, Ar_AC*H*), 7.39 – 7.31 (m, 2H, Ar_AC*H*), 7.31 – 7.16 (m, 5H, Ar_BC*H*), 4.11 (s, 2H, SCH_2), 3.10 – 3.01 (m, 4H, NCH_2), 1.60 – 1.46 (m, 4H, NCH_2CH_2), 0.86 (t, *J* = 7.4 Hz, 6H, CH_3); ^{13}C NMR (101 MHz, $CDCl_3$) δ 142.6 (Ar_A*C*CH_2), 139.0 (Ar_A*C*SO_2), 135.1 (Ar_B*C*), 131.0 (Ar_B*C*H), 129.5 (Ar_A*C*H), 129.1 (Ar_B*C*H), 127.3 (Ar_A*C*H), 127.2 (Ar_B*C*H), 50.1 (2 x N*C*H_2), 39.1 (S*C*H_2), 22.1 (2 x NCH_2*C*H_2), 11.3 (2 x *C*H_3). HRMS (APCI): Calculated for $C_{19}H_{26}O_2NS_2$ $(M-H)^+$, 364.1396; found 364.1399.

(2-Iodobenzyl)(phenyl)sulfide 1i

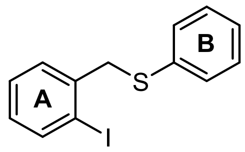

Following **GP2**, *1-(bromomethyl)-2-iodobenzene* (1.48 g, 5.00 mmol) afforded **1i** (1.24 g, 50%) as a colorless liquid, after purification by column chromatography on silica gel (hexane/EtOAc 100:0 to 100:2). ^{1}H NMR (400 MHz, $CDCl_3$) δ 7.90 – 7.81 (m, 1H, Ar_AC*H*), 7.39 – 7.34 (m, 2H, Ar_AC*H*), 7.32 – 7.21 (m, 5H, Ar_BC*H*), 6.97 – 6.89 (m, 1H, Ar_AC*H*), 4.23 (s, 2H, CH_2); ^{13}C NMR (101 MHz, $CDCl_3$) δ 140.0 (Ar_A*C*), 139.8 (Ar_A*C*H), 135.7 (Ar_B*C*), 130.9 (Ar_A*C*H), 130.2 (Ar_B*C*H), 129.0 (2 x Ar_A*C*H), 128.4 (Ar_B*C*H), 126.9 (Ar_B*C*H), 100.7 (Ar_A*C*I), 44.8 (*C*H_2). Data in accordance with the literature.[8]

Benzyl (3-((phenylthio)methyl)phenyl)carbamate 1j

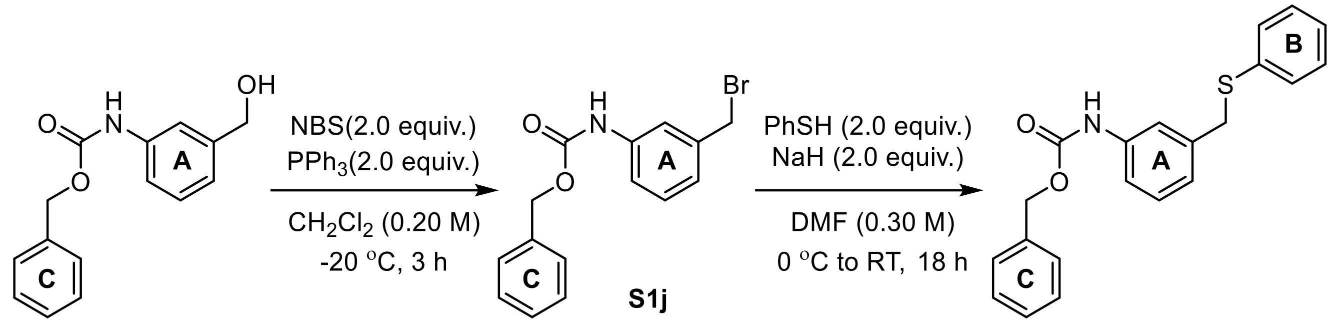

In a two-necked round-bottomed flask, dried under vacuum and equipped with a stirring bar, triphenylphosphine (1.02 g, 3.88 mmol) was added to a solution of benzyl (3-(hydroxymethyl)phenyl)carbamate (500 mg, 1.94 mmol) in CH_2Cl_2 (1.95 mL, 0.2 M). The solution was cooled to -20 °C and *N*-bromosuccinimide (691 mg, 3.88 mmol) was added portion-wise. The solution was stirred for 3 h at this temperature and then concentrated under reduced pressure. The crude was purified by column chromatography (hexane/ethyl acetate 9/1) to give *benzyl (3-(bromomethyl)phenyl)carbamate* **S1j** (452 mg, 73%) as a white solid (m.p. 100 – 102 °C).

^{1}H NMR (400 MHz, $CDCl_3$) δ 7.51 (s, 1H, Ar_AH), 7.42 – 7.33 (m, 5H, Ar_CH), 7.29 – 7.25 (m, 2H, Ar_AH), 7.10 (d, *J* = 4.6 Hz, 1H, Ar_AH), 6.72 (s, 1H, N*H*), 5.21 (s, 2H, CH_2O), 4.45 (s, 2H, CH_2); ^{13}C NMR (101 MHz, $CDCl_3$) δ 153.3 (*C*O), 138.9 (Ar_A*C*CH_2), 138.3 (Ar_A*C*), 136.0 (Ar_C*C*), 129.6 (Ar_CCH), 128.8 (Ar_C*C*H), 128.6 (Ar_A*C*H), 128.5 (Ar_CCH), 124.2 (Ar_A*C*H), 119.2 (Ar_A*C*H), 118.7 (Ar_A*C*H), 67.3 (*C*H_2O), 33.3 (*C*H_2). HRMS (APCI): Calculated for $C_{15}H_{13}O_2NBr$ $(M-H)^-$, 318.0124; found 318.0134.

Following **GP1**, *benzyl (3-(bromomethyl)phenyl)carbamate* **S1j** (452 mg, 1.41 mmol) afforded **1j** (300 mg, 61%) as a white solid (m.p. 66 – 68 °C), after purification by column chromatography on silica gel (hexane/EtOAc 100:0 to 1:1). ^{1}H NMR (400 MHz, $CDCl_3$) δ 7.44 – 7.13 (m, 13H, Ar_AC*H* and Ar_BC*H* and Ar_CC*H*), 6.99 (m, 1H, Ar_AC*H*), 6.61 (brs, 1H, N*H*), 5.20 (s, 2H, OCH_2), 4.09 (s, 2H, SCH_2); ^{13}C NMR (101 MHz, $CDCl_3$) δ 153.3 (*C*O), 138.7 (Ar_B*C*), 138.1 (Ar_A*C*), 136.3 (Ar_A*C*CH_2), 136.1 (Ar_C*C*), 129.9 (Ar_CCH)*, 129.3 (Ar_C*C*H)*, 129.2 (Ar_B*C*H), 129.0 (Ar_BCH), 128.8 (Ar_BCH), 128.5 (Ar_C*C*H)*, 128.4 (Ar_C*C*H)*, 127.6 (Ar_CCH)*, 127.3 (Ar_C*C*H)*, 126.5 (Ar_ACH), 124.1 (Ar_A*C*H), 119.0 (Ar_A*C*H), 117.6 (Ar_A*C*H), 67.2 (O*C*H_2), 39.0 (S*C*H_2). Some carbon atoms (highlighted with the

asterisk) appear as two signals rather than one due to the presence of rotamers around the carbamate bond. HRMS (APCI): Calculated for $C_{21}H_{18}O_2NS$ $(M-H)^-$, 348.1064; found 348.1064.

(3,5-Di-*tert*-butylbenzyl)(phenyl)sulfide 1k

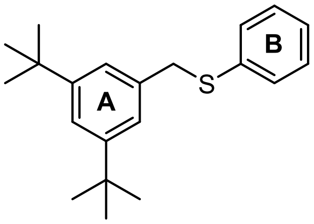

Following **GP1**, *1-(bromomethyl)-3,5-di-tert-butylbenzene* (708 mg, 2.50 mmol) afforded **1k** (503 mg, 64%) as a colorless liquid, after purification by column chromatography on silica gel (hexane/EtOAc 100:0 to 10:1). ^1H NMR (500 MHz, $CDCl_3$) δ 7.35 – 7.21 (m, 5H, Ar_BCH), 7.20 – 7.16 (m, 1H, Ar_ACH), 7.09 (m, 2H, Ar_ACH), 4.11 (s, 2H, CH_2), 1.28 (s, 18H, CH_3); ^{13}C NMR (126 MHz, $CDCl_3$) δ 151.0 (Ar_AC), 136.8 (Ar_ACCH_2), 136.4 (Ar_BC), 130.3 (Ar_BCH), 128.9 (Ar_BCH), 126.5 (Ar_ACH), 123.3 (Ar_ACH), 121.3 (Ar_BCH), 40.0 (CH_2), 34.9 (CCH_3), 31.6 (CH_3). HRMS (APCI): Calculated for $C_{21}H_{28}S$ $(M)^+$, 312.1906; found 312.1891.

2-Methoxy-4-((phenylthio)methyl)phenyl acetate 1l

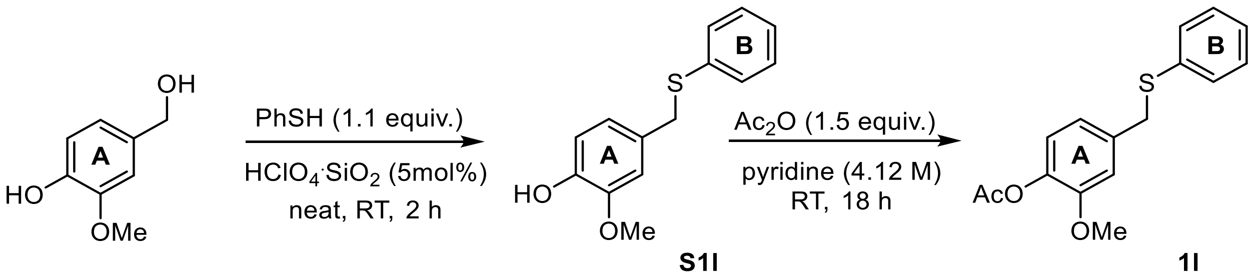

Following **GP3**, *4-(hydroxymethyl)-2-methoxyphenol* (500 mg, 3.24 mmol) afforded *2-methoxy-4-((phenylthio)methyl)phenol* **S1l**, which was used in the next step without any further purification.

A solution of *2-methoxy-4-((phenylthio)methyl)phenol* **S1l** (250 mg, 1.01 mmol), gave **1l** (200 mg, 69% over 2 steps) – synthesized following a literature procedure[9] – as a white solid (m.p. 70 – 72 °C), after purification by column chromatography on silica gel (hexane/EtOAc 100:0 to 9:1). ^1H NMR

(400 MHz, $CDCl_3$) δ 7.37 – 7.15 (m, 5H, Ar_BCH), 6.93 (d, J = 7.7 Hz, 1H, Ar_ACH), 6.88 – 6.81 (m, 2H, Ar_ACH), 4.08 (s, 2H, CH_2), 3.76 (s, 3H, OCH_3), 2.30 (s, 3H, $COCH_3$); ^{13}C NMR (101 MHz, $CDCl_3$) δ 169.2 (*C*O), 151.1 (Ar_ACOCH_3), 139.0 (Ar_ACOAc), 136.6 (Ar_ACCH_2), 136.1 (Ar_BCS), 130.4 (Ar_ACH), 129.0 (Ar_ACH), 126.7 (Ar_ACH), 122.8 (Ar_BCH), 121.1 (Ar_BCH), 113.0 (Ar_BCH), 56.0 (OCH_3), 39.3 (CH_2), 20.8 ($COCH_3$). HRMS (APCI): Calculated for $C_{16}H_{16}O_3SNa$ $(M\text{-}Na)^+$, 311.0710; found 311.0712.

(Naphthalen-2-yl-methyl)(phenyl)sulfide 1m

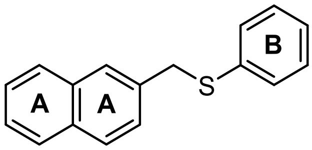

Following **GP2**, *2-(bromomethyl)naphthalene* (1.32 g, 6.00 mmol) afforded **1m** (1.08 g, 90%) as a white solid, after purification by column chromatography on silica gel (hexane/EtOAc 100:0 to 100:2). ^1H NMR (500 MHz, $CDCl_3$) δ 7.78 – 7.65 (m, 3H, Ar_ACH), 7.60 (d, J = 1.8 Hz, 1H, Ar_ACH), 7.45 – 7.35 (m, 3H, Ar_ACH), 7.27 – 7.24 (m, 2H, Ar_BCH), 7.19 – 7.14 (m, 2H, Ar_BCH), 7.12 – 7.08 (m, 1H, Ar_BCH), 4.20 (s, 2H, CH_2); ^{13}C NMR (126 MHz, $CDCl_3$) δ 136.3 (Ar_BC), 135.0 (Ar_ACCH_2), 133.4 (Ar_AC), 132.7 (Ar_AC), 130.2 (Ar_BCH), 129.0 (Ar_BCH), 128.4 (Ar_ACH), 127.8 (Ar_ACH), 127.7 (Ar_ACH), 127.5 (Ar_ACH), 127.1 (Ar_ACH), 126.6 (Ar_ACH), 126.3 (Ar_BCH), 126.0 (Ar_ACH), 39.6 (CH_2). Data in accordance with the literature.[10]

2-((Phenylthio)methyl)thiophene 1n

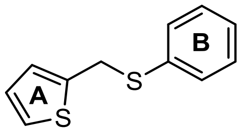

Following **GP3**, *thiophen-2-ylmethanol* (307 µL, 3.24 mmol) afforded **1n** (667 mg, >99%) as a white solid, after purification by column chromatography on silica gel (hexane/EtOAc 100:0 to 96:4). ^1H NMR (400 MHz, $CDCl_3$) δ 7.38 – 7.30 (m, 2H, Ar_BCH), 7.28 – 7.23 (m, 2H, Ar_BCH), 7.22 – 7.13 (m,

2H, Ar_AC*H* and Ar_BC*H*), 6.90 – 6.83 (m, 2H, Ar_AC*H*), 4.29 (s, 2H, CH_2); ^{13}C NMR (101 MHz, $CDCl_3$) δ 140.9 (Ar_A*C*CH_2S), 135.7 (Ar_B*C*S), 130.3 (Ar_BCH), 129.0 (Ar_B*C*H), 127.0 (Ar_B*C*H), 126.9 (Ar_A*C*H), 126.3 (Ar_A*C*H), 125.0 (Ar_A*C*H), 33.7 (*C*H_2). Data in accordance with the literature.[11]

Neopentyl(phenyl)sulfide 1o

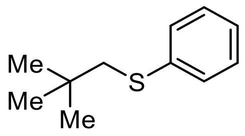

1o (200 mg, 85%) was synthesized following a literature procedure[12] as a colorless liquid, after purification by column chromatography on silica gel (hexane/EtOAc 100:0 to 96:4). ^{1}H NMR (500 MHz, $CDCl_3$) δ 7.26 – 7.23 (m, 2H, ArC*H*), 7.16 (dd, *J* = 8.6, 7.1 Hz, 2H, ArC*H*), 7.07 – 7.02 (m, 1H, ArC*H*), 2.80 (s, 2H, CH_2), 0.95 (s, 9H, 3 x CH_3); ^{13}C NMR (126 MHz, $CDCl_3$) δ 138.5 (Ar*C*), 129.0 (Ar*C*H), 128.9 (Ar*C*H), 125.6 (Ar*C*H), 48.7 (*C*H_2), 32.6 (*C*CH_3), 29.2 (3 x *C*H_3). Data in accordance with the literature.[13]

Adamantan-1-yl(methyl)(phenyl)sulfide 1p

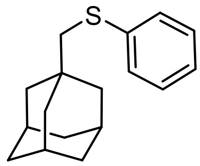

Following **GP4**, *adamantan-1-ylmethanol* (333 mg, 2.00 mmol) afforded **1p** (414 mg, 80%) as a white solid, after purification by column chromatography on silica gel (hexane 100%). ^{1}H NMR (500 MHz, $CDCl_3$) δ 7.37 – 7.33 (m, 2H, ArC*H*), 7.27 – 7.23 (m, 2H, ArC*H*), 7.15 – 7.11 (m, 1H, ArC*H*), 2.78 (s, 2H, SCH_2), 1.99 (p, *J* = 3.2 Hz, 3H, CH_2 and C*H*), 1.74 – 1.60 (m, 12H, CH_2 and C*H*); ^{13}C NMR (126 MHz, $CDCl_3$) δ 138.8 (Ar*C*), 128.9 (Ar*C*H), 128.9 (Ar*C*H), 125.5 (Ar*C*H), 48.7 (S*C*H_2), 42.1 (*C*H_2 and *C*H), 37.0 (*C*H_2 and *C*H), 34.3 (Alk*C*), 28.7 (*C*H_2 and *C*H). HRMS (APCI): Calculated for $C_{17}H_{23}S$ $(M-H)^+$, 259.1515; found 259.1510.

Cyclopropylmethyl(phenyl)sulfide 1q

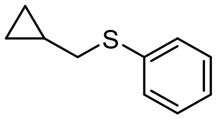

Following **GP4**, *cyclopropylmethanol* (227 mL, 2.80 mmol) afforded **1q** (400 mg, 87%) as a colorless liquid, after purification by column chromatography (hexane 100%). ^{1}H NMR (400 MHz, $CDCl_3$) δ 7.40 – 7.36 (m, 2H, ArC*H*), 7.30 – 7.26 (m, 2H, ArC*H*), 7.21 – 7.15 (m, 1H, ArC*H*), 2.88 (d, *J* = 7.0 Hz, 2H, SCH_2), 1.14 – 1.01 (m, 1H, C*H*), 0.65 – 0.55 (m, 2H, CH_2), 0.30 – 0.23 (m, 2H, CH_2); ^{13}C NMR (101 MHz, $CDCl_3$) δ 137.2 (Ar*C*), 129.4 (Ar*C*H), 128.9 (Ar*C*H), 125.9 (Ar*C*H), 39.7 (S*C*H_2), 10.8 (*C*H), 5.7 (2 x *C*H_2). Data in accordance with the literature.[18]

2-(4-((Phenylthio)methyl)phenyl)propan-2-ol 1v

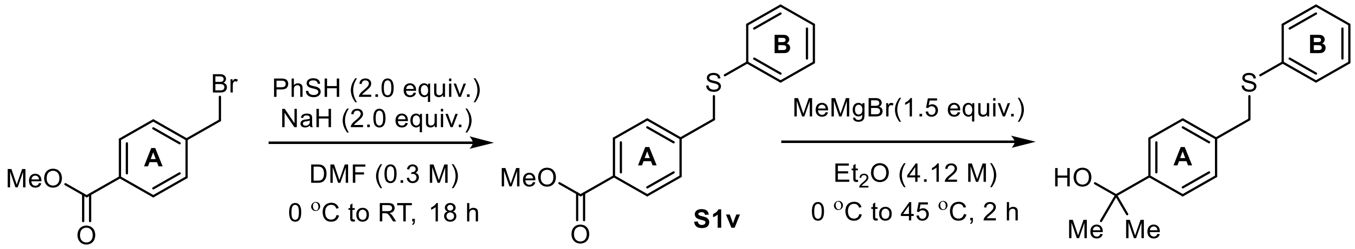

Following **GP1**, *methyl 4-(bromomethyl)benzoate* (573 mg, 2.50 mmol) afforded **S1v** as a white solid, which was used in the next step without any further purification.

2-(4-((Phenylthio)methyl)phenyl)propan-2-ol **1v** was prepared according to literature procedure, starting from *methyl 4-((phenylthio)methyl)benzoate* **S1v** (304 mg, 1.24 mmol) and affording **1v** (290 mg, 91% over 2 steps) as a yellow solid (m.p. 50 – 52 °C), after purification by column chromatography on silica gel (hexane/EtOAc 9:1 to 1:1). ^{1}H NMR (400 MHz, $CDCl_3$) δ 7.45 – 7.38 (m, 2H, Ar_AC*H*), 7.36 – 7.14 (m, 7H, Ar_AC*H* and Ar_BC*H*), 4.12 (s, 2H, CH_2), 1.70 (brs, 1H, O*H*) 1.57 (s, 6H, 2 x CH_3); ^{13}C NMR (101 MHz, $CDCl_3$) δ 148.2 (Ar_A*C*), 136.7 (Ar_B*C*), 135.9 (Ar_A*C*CH_2), 129.7 (Ar_B*C*H), 129.0 (Ar_B*C*H), 128.8 (Ar_ACH), 126.4 (Ar_B*C*H), 124.7 (Ar_ACH), 72.6 (*C*CH_3), 38.7 (*C*H_2), 31.9 (2 x *C*H_3). HRMS (ESI): Calculated for $C_{16}H_{18}OSNa$ $(M\text{-}Na)^+$, 281.0970; found 281.0971.

Phenyl(4-((phenylthio)methyl)phenyl)methanone 1w

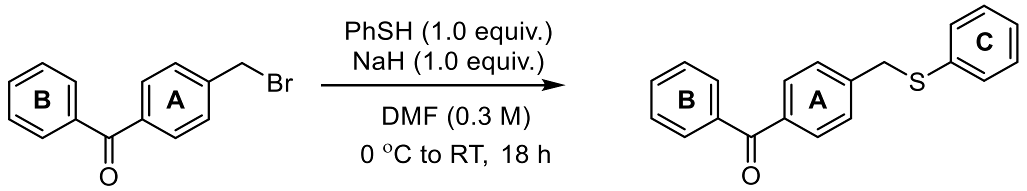

Following **GP2**, *(4-(bromomethyl)phenyl)(phenyl)methanone* (1.38 g, 5.00 mmol) afforded **1w** (947 mg, 73%) as a white solid (m.p. 73 – 75 °C), after purification by column chromatography on silica gel (hexane/EtOAc 100:0 to 9:1). ^{1}H NMR (500 MHz, $CDCl_3$) δ 7.78 (dd, *J* = 8.3, 1.3 Hz, 2H, Ar_BCH), 7.73 (d, *J* = 8.3 Hz, 2H, Ar_ACH), 7.60 – 7.56 (m, 1H, Ar_BCH), 7.48 (dd, *J* = 8.4, 7.1 Hz, 2H, Ar_BCH), 7.38 (d, *J* = 8.2 Hz, 2H, Ar_ACH), 7.33 – 7.31 (m, 2H, Ar_CCH), 7.28 – 7.25 (m, 2H, Ar_CCH), 7.23 – 7.19 (m, 1H, Ar_CCH), 4.16 (s, 2H, CH_2); ^{13}C NMR (126 MHz, $CDCl_3$) δ 196.4 (*C*O), 142.7 (Ar_ACCH_2), 137.8 (Ar_BC), 136.5 (Ar_ACCO), 135.6 (Ar_CC), 132.5 (Ar_BCH), 130.5 (Ar_ACH and Ar_CCH), 130.1 (Ar_BCH), 129.1 (Ar_CCH), 128.8 (Ar_ACH), 128.4 (Ar_BCH), 127.0 (Ar_CCH), 39.2 (CH_2). HRMS (APCI): Calculated for $C_{20}H_{17}OS$ $(M-H)^+$, 305.0995; found 305.0997.

2-(4-((Phenylthio)methyl)phenyl)pyridine 1x

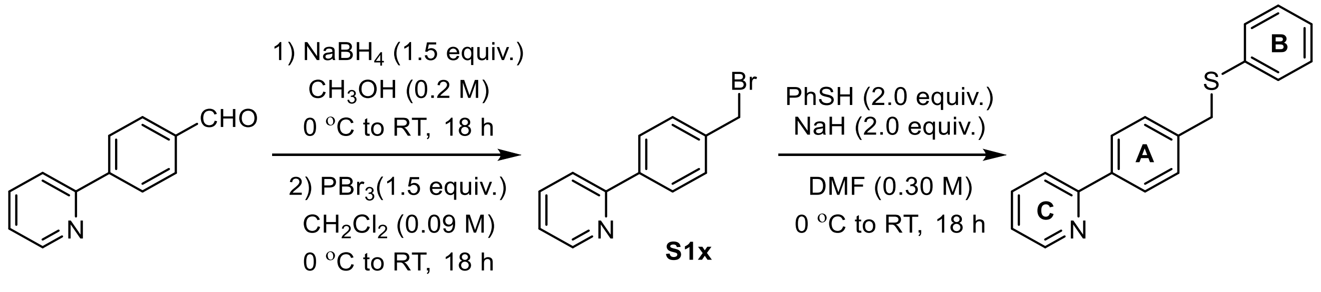

In a one-necked round-bottomed flask, equipped with a stirring bar, $NaBH_4$ (284 mg, 7.50 mmol) was added portion-wise to a solution of *4-(pyridin-2-yl)benzaldehyde* (916 mg, 5.00 mmol) in methanol (25 mL, 0.2 M), at 0 °C. The reaction mixture was allowed to reach RT and monitored by TLC. When all starting material was converted, the mixture was quenched with water (30 mL) and extracted with ethyl acetate (3 x 30 mL). The organic phases were collected and dried over $MgSO_4$. The solvent was removed in vacuo and the crude was used in the next step without any further purification.

In a two-necked round-bottomed flask, dried under vacuum and equipped with a stirring bar, PBr_3 (1.5 mL, 1.5 equiv.) was added dropwise to a solution of the crude *(4-(pyridin-2-yl)phenyl)methanol* **S1x** (500 mg, 2.7 mmol) in CH_2Cl_2 (30 mL, 0.09 M), at 0 °C. The reaction mixture was allowed to reach RT and monitored by TLC. When all starting material was converted, the mixture was quenched with water (30 mL) and extracted with ethyl acetate (3 x 30 mL). The organic phases were collected and dried over $MgSO_4$. The solvent was removed in vacuo and the crude was used in the next step without any further purification.

Following **GP1**, *2-(4-(bromomethyl)phenyl)pyridine* **S1x** (236 mg, 0.95 mmol) afforded **1x** (190 mg, 25% over 3 steps) as a colorless liquid, after purification by column chromatography on silica gel (hexane/EtOAc 100:0 to 8:2). ^{1}H NMR (400 MHz, $CDCl_3$) δ 8.71 – 8.65 (m, 1H, Ar_CC*H*), 7.91 (d, *J* = 8.3 Hz, 2H, Ar_AC*H*), 7.76 – 7.69 (m, 2H, Ar_CC*H*), 7.39 (d, *J* = 8.1 Hz, 2H, Ar_AC*H*), 7.32 (d, *J* = 7.7 Hz, 2H, Ar_BC*H*), 7.28 – 7.15 (m, 1H, Ar_CC*H* and 3H, Ar_BC*H*), 4.16 (s, 2H, CH_2); ^{13}C NMR (101 MHz, $CDCl_3$) δ 157.2 (Ar_C*C*), 149.8 (Ar_C*C*H), 138.6 (Ar_B*C*), 138.4 (Ar_A*C*), 136.9 (Ar_C*C*H), 136.2 (Ar_A*C*CH_2), 130.3 (Ar_B*C*H), 129.4 (Ar_A*C*H), 129.0 (Ar_B*C*H), 127.1 (Ar_A*C*H), 126.6 (Ar_B*C*H), 122.2 (Ar_C*C*H), 120.6 (Ar_C*C*H), 39.1 (*C*H_2). HRMS (APCI): Calculated for $C_{18}H_{16}NS$ $(M-H)^+$, 278.0994; found 278.0998.

(3,5-bis(Trifluoromethyl)benzyl)(phenyl)sulfide 1y

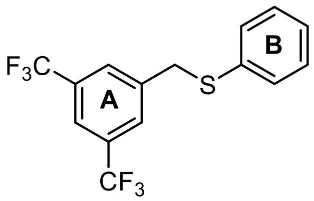

Following **GP1**, *1-(Bromomethyl)-3,5-bis(trifluoromethyl)benzene* (917 μL, 5.00 mmol) afforded **1y** (1.13 g, 67%) as a liquid, after purification by column chromatography on silica gel (hexane/EtOAc 100:0 to 9:1). ^{1}H NMR (500 MHz, $CDCl_3$) δ 7.72 (s, 1H, Ar_AC*H*), 7.60 (d, *J* = 1.8 Hz, 2H, Ar_AC*H*), 7.29 – 7.22 (m, 5H, Ar_BC*H*), 4.12 (s, 2H, CH_2); ^{13}C NMR (126 MHz, $CDCl_3$) δ 140.7 (Ar_A*C*), 134.1 (Ar_B*C*), 131.8 (Ar_B*C*H), 131.8 (q, *J* = 33.3 Hz, 2 x Ar_A*C*CF_3), 129.3 (Ar_B*C*H), 129.1 (q, *J* = 3.6 Hz, Ar_A*C*H), 127.8 (Ar_B*C*H), 123.3 (q, *J* = 272.8 Hz, 2 x *C*F_3), 121.3 – 121.1 (m, Ar_A*C*H), 39.3 (*C*H_2); ^{19}F

NMR (376 MHz, $CDCl_3$) δ -62.95. HRMS (APCI): Calculated for $C_{15}H_9F_6S$ $(M-H)^-$, 335.0345; found 335.0353.

(4-Fluorobenzyl)(4-methoxyphenyl)sulfide 1ca

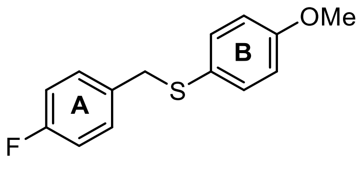

Following **GP1**, *1-(bromomethyl)-4-fluorobenzene* (623 μL, 5.00 mmol) afforded **1ca** (1.1 g, 87%) as a white solid, after purification by column chromatography on silica gel (hexane/EtOAc 100:0 to 9:1). ^1H NMR (500 MHz, $CDCl_3$) δ 7.25 – 7.22 (m, 2H, Ar_BCH), 7.15 – 7.10 (m, 2H, Ar_ACH), 6.93 (m, 2H, Ar_BCH), 6.83 – 6.77 (m, 2H, Ar_ACH), 3.94 (s, 2H, CH_2), 3.78 (s, 3H, CH_3); ^{13}C NMR (126 MHz, $CDCl_3$) δ 162.0 (d, J = 245.1 Hz, Ar_ACF), 159.5 (Ar_BCOMe), 134.5 (Ar_BCH), 134.1 (d, J = 3.2 Hz, Ar_AC), 130.5 (d, J = 8.1 Hz, Ar_ACH), 125.7 (Ar_BC), 115.3 (d, J = 21.5 Hz, Ar_ACH), 114.6 (Ar_BCH), 55.4 (CH_3), 40.6 (CH_2). Data in accordance with the literature.[13]

(4-Fluorobenzyl)(4-fluorophenyl)sulfide 1cb

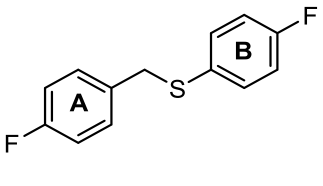

Following **GP1**, *1-(bromomethyl)-4-fluorobenzene* (623 μL, 5.00 mmol) afforded **1cb** (950 mg, 81%) as a yellow solid, after purification by column chromatography on silica gel (hexane/EtOAc 100:0 to 9:1). ^1H NMR (500 MHz, $CDCl_3$) δ 7.28 – 7.23 (m, 2H, Ar_BCH), 7.18 – 7.12 (m, 2H, Ar_ACH), 6.95 (m, 4H, Ar_ACH and Ar_BCH), 3.99 (s, 2H, CH_2); ^{13}C NMR (126 MHz, $CDCl_3$) δ 162.4 (d, J = 247.1 Hz, $ArCF$), 162.1 (d, J = 245.8 Hz, $ArCF$), 133.9 (d, J = 8.2 Hz, Ar_BCH), 133.5 (d, J = 3.1 Hz, Ar_BC), 130.5 (d, J = 8.2 Hz, Ar_ACH), 130.4 (d, J = 3.3 Hz, Ar_AC), 116.1 (d, J = 21.7 Hz, Ar_BCH), 115.5 (d, J = 21.5 Hz, Ar_ACH), 39.9 (CH_2). Data in accordance with the literature.[14]

tert-Butyl(4-fluorobenzyl)sulfide 1cc

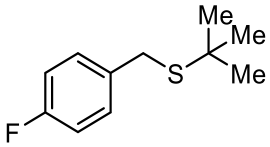

Following **GP1**, *1-(bromomethyl)-4-fluorobenzene* (623 μL, 5.00 mmol) afforded **1cc** (828 mg, 84%) as a yellow liquid, after purification by column chromatography on silica gel (hexane/EtOAc 100:0 to 9:1). ^{1}H NMR (500 MHz, $CDCl_3$) δ 7.40 – 7.28 (m, 2H, ArC*H*), 6.97 (t, *J* = 8.7 Hz, 2H, ArC*H*), 3.74 (s, 2H, CH_2), 1.35 (s, 9H, 3 x CH_3); ^{13}C NMR (126 MHz, $CDCl_3$) δ 161.9 (d, *J* = 245.0 Hz, Ar*C*F), 134.4 (d, *J* = 3.1 Hz, Ar*C*), 130.5 (d, *J* = 8.1 Hz, Ar*C*H), 115.4 (d, *J* = 21.5 Hz, Ar*C*H), 43.0 (S*C*CH_3), 32.8 (*C*H_2), 31.0 (3 x *C*H_3); ^{19}F NMR (471 MHz, $CDCl_3$) δ -116.07. HRMS (APCI): Calculated for $C_{11}H_{16}FS$ (M-H)$^+$, 199.0956; found 199.0951.

(4-Fluorobenzyl)(isopropyl)sulfide 1cd

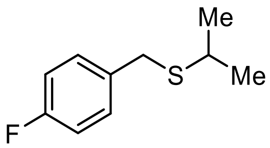

Following **GP1**, *1-(bromomethyl)-4-fluorobenzene* (623 μL, 5.00 mmol) afforded **1cd** (657 mg, 71%) as a colorless liquid, after purification by column chromatography on silica gel (hexane/EtOAc 100:0 to 9:1). ^{1}H NMR (500 MHz, $CDCl_3$) δ 7.32 – 7.27 (m, 2H, ArC*H*), 7.02 – 6.95 (m, 2H, ArC*H*), 3.71 (s, 2H, CH_2), 2.79 (h, *J* = 6.7 Hz, 1H, C*H*), 1.25 (d, *J* = 6.8 Hz, 6H, CH(CH_3)$_2$); ^{13}C NMR (126 MHz, $CDCl_3$) δ 161.9 (d, *J* = 245.1 Hz, Ar*C*F), 134.6 (d, *J* = 3.3 Hz, Ar*C*), 130.3 (d, *J* = 7.9 Hz, Ar*C*H), 115.5 (d, *J* = 21.4 Hz, Ar*C*H), 34.5 (CH_2), 34.4 (*C*H), 23.2 (2 x *C*H_3); ^{19}F NMR (471 MHz, $CDCl_3$) δ -115.95. HRMS (APCI): Calculated for $C_{10}H_{12}FS$ (M-H)$^+$, 183.0641; found 183.0638.

(4-Fluorobenzyl)(octyl)sulfide 1ce

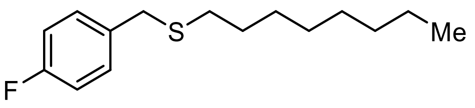

Following **GP1**, *1-(bromomethyl)-4-fluorobenzene* (623 μL, 5.00 mmol) afforded **1ce** (890 mg, 70%) as a colorless liquid, after purification by column chromatography on silica gel (hexane/EtOAc 100:0 to 9:1). ^{1}H NMR (500 MHz, $CDCl_3$) δ 7.31 – 7.24 (m, 2H, ArC*H*), 7.03 – 6.95 (m, 2H, ArC*H*), 3.67 (s, 2H, SC*H*$_2$), 2.43 – 2.36 (m, 2H, SC*H*$_2$CH$_2$), 1.59 – 1.50 (m, 2H, SCH$_2$C*H*$_2$), 1.34 – 1.20 (m, 10H, C*H*$_2$), 0.88 (t, *J* = 7.0 Hz, 3H, C*H*$_3$); ^{13}C NMR (126 MHz, $CDCl_3$) δ 161.9 (d, *J* = 245.2 Hz, Ar*C*F), 134.5 (d, *J* = 3.1 Hz, Ar*C*), 130.4 (d, *J* = 7.9 Hz, Ar*C*H), 115.4 (d, *J* = 21.4 Hz, Ar*C*H), 35.7 (S*C*H$_2$Ar), 31.9 (S*C*H$_2$), 31.5 (SCH$_2$*C*H$_2$), 29.3 (SCH$_2$CH$_2$*C*H$_2$), 29.3 (2 x *C*H$_2$), 29.0 (*C*H$_2$), 22.8 (*C*H$_2$), 14.2 (*C*H$_3$). Data in accordance with the literature.[15]

Benzylhydryl(phenyl)sulfide 1aa

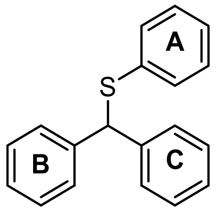

Following **GP2**, *(bromomethylene)dibenzene* (1.2 g, 5.00 mmol) afforded **1aa** (1.09 g, 79%) as a white solid, after purification by column chromatography on silica gel (hexane/EtOAc 100:0 to 98:2). ^{1}H NMR (400 MHz, CDCl3) δ 7.44 (d, *J* = 7.2 Hz, 4H, ArC*H*), 7.35 – 7.15 (m, 11H, ArC*H*), 5.57 (s, 1H, C*H*); ^{13}C NMR (126 MHz, $CDCl_3$) δ 141.1 (Ar*C*), 136.3 (Ar*C*), 130.6 (Ar*C*H), 128.9 (Ar*C*H), 128.7 (Ar*C*H), 128.5 (Ar*C*H), 127.4 (Ar*C*H), 126.7 (Ar*C*H), 57.6 (*C*H). Data in accordance with the literature.[16]

Phenyl(phenyl(4-(trifluoromethyl)phenyl)methyl)sulfide 1ab

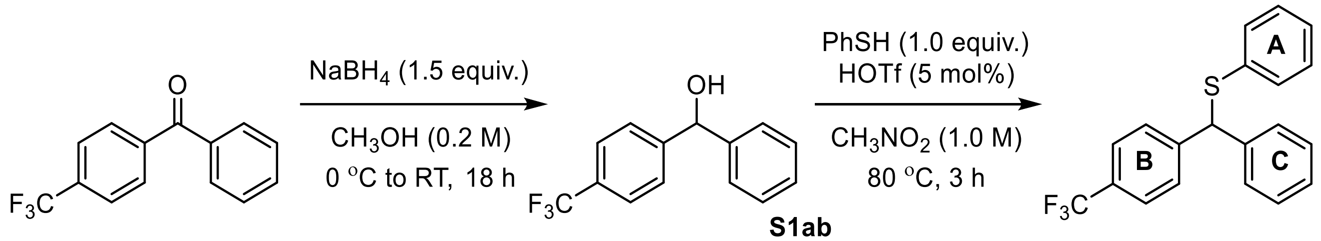

S1ab

In a one-necked round-bottomed flask, equipped with a stirring bar, $NaBH_4$ (284 mg, 7.50 mmol) was added portion-wise to a solution of *phenyl(4-(trifluoromethyl)phenyl)methanone* (1.2 g, 5.00 mmol) in methanol (25 mL, 0.2 M), at 0 °C. The reaction was allowed to reach RT and monitored by TLC. When all starting material was converted, the mixture was quenched with water (30 mL) and extracted with ethyl acetate (3 x 30 mL). The organic phases were collected and dried over $MgSO_4$. The solvent was removed in vacuo and the crude **S1z** was used in the next step without any further purification.

Following **GP5**, *phenyl(4-(trifluoromethyl)phenyl)methanol* **S1ab** (1.2 g, 5.0 mmol) afforded **1ab** (1.3 g, 74%) as a yellow solid, after purification by column chromatography on silica gel (hexane/EtOAc 100:0 to 98:2). ^{1}H NMR (400 MHz, $CDCl_3$) δ 7.62 (m, 4H, Ar_BC*H*), 7.49 (dt, *J* = 7.4, 2.3 Hz, 2H, Ar_CC*H*), 7.40 (tt, *J* = 7.8, 1.7 Hz, 2H, Ar_CC*H*), 7.37 – 7.24 (m, 6H, Ar_ACH and Ar_CC*H*), 5.67 – 5.64 (m, 1H, C*H*); ^{13}C NMR (101 MHz, $CDCl_3$) δ 145.3 (Ar_B*C*), 140.2 (Ar_A*C*), 135.4 (Ar_C*C*), 131.1 (Ar_ACH), 129.5 (q, *J* = 32.3 Hz, Ar_B*C*CF_3), 129.0 (Ar_ACH), 128.9 (Ar_BCH), 128.8 (Ar_CCH), 128.5 (Ar_CCH), 127.8 (Ar_C*C*H), 127.2 (Ar_ACH), 125.6 (q, *J* = 3.9 Hz, Ar_B*C*H), 124.2 (q, *J* = 271.9 Hz, CF_3), 57.3 (*C*H); ^{19}F NMR (376 MHz, $CDCl_3$) δ -62.41. HRMS (APCI): Calculated for $C_{20}H_{15}F_3S$ $(M-H)^-$, 343.0774; found 343.0771.

***iso*Propyl-2-(4-((4-chlorophenyl)(phenylthiol)methyl)phenoxy)-2-methylpropanoate 1ac**

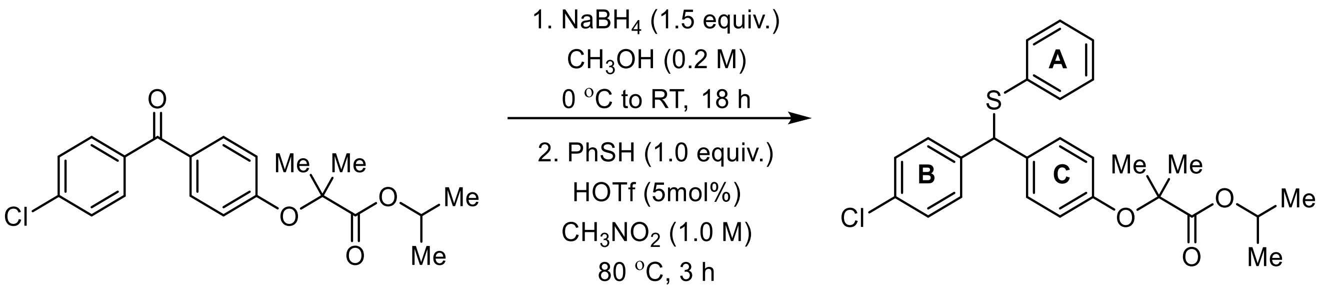

In a one-necked round-bottomed flask, equipped with a stirring bar, $NaBH_4$ (284 mg, 7.50 mmol) was added portion-wise to a solution of *isopropyl-2-(4-(4-chlorobenzoyl)phenoxy)-2-methylpropanoate* (1.8 g, 5.00 mmol) in methanol (25 mL, 0.2 M), at 0 °C. The reaction was allowed to reach RT and monitored by TLC. When all starting material was converted, the mixture was quenched with water (30 mL) and extracted with ethyl acetate (3 x 30 mL). The organic phases were collected and dried over $MgSO_4$. The solvent was removed in vacuo and the crude **S1aa** was used in the next step without any further purification.

Following **GP5**, *isopropyl-2-(4-((4-chlorophenyl)hydroxy)methyl)methylpropanoate* **S1ac** (1.8 g, 5.00 mmol) afforded **1ac** (1.2 g, 53%) as a yellow solid, after purification by column chromatography on silica gel (hexane/EtOAc 100:0 to 9:1). ^{1}H NMR (400 MHz, $CDCl_3$) δ 7.26 (m, 2H, Ar_BC*H*), 7.18 – 7.04 (m 9H, Ar_AC*H* and Ar_CC*H*), 6.73 – 6.68 (m, 2H, Ar_BC*H*), 5.38 (s, 1H, SC*H*), 4.99 (p, *J* = 6.3 Hz, 1H, OC*H*), 1.50 (s, 6H, 2 x CC*H*$_3$), 1.11 (d, *J* = 6.3 Hz, 6H, 2 x CHC*H*$_3$); ^{13}C NMR (101 MHz, $CDCl_3$) δ 173.6 (*C*O), 154.9 (Ar_B*C*), 139.8 (Ar_C*C*CHS), 135.7 (Ar_A*C*S), 133.7 (Ar_B*C*CHS), 133.0 (Ar_C*C*O), 130.9 (Ar_ACH or Ar_CCH), 129.8 (Ar_BCH), 129.1 (Ar_ACH or Ar_CCH), 128.8 (Ar_ACH or Ar_CCH), 128.7 (Ar_A*C*H or Ar_C*C*H), 126.9 (Ar_A*C*H), 118.8 (Ar_BCH), 79.2 (*C*(CH_3)$_2$), 69.0 (O*C*H), 56.3 (S*C*H), 25.4 (2 x C*C*H$_3$), 21.6 (2 x CH*C*H$_3$). HRMS (APCI): Calculated for $C_{26}H_{27}ClO_3SNa$ (M-Na)$^+$, 477.1267; found 477.1266.

Methyl-(4-(phenyl(phenylthio)methyl)benzoyl)-*D*-prolinate 1ad

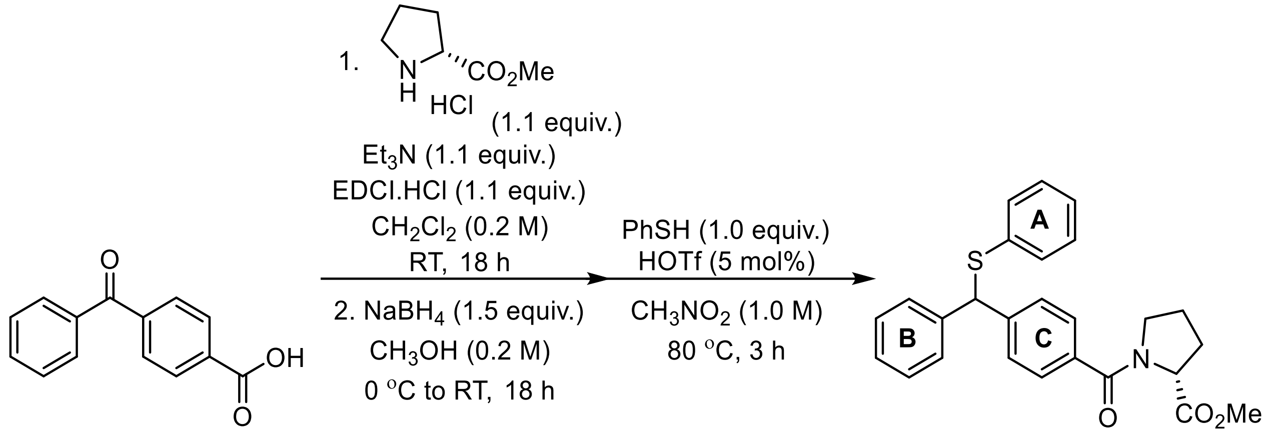

In a one-necked round-bottomed flask, equipped with a stirring bar, Et_3N (532 μL, 3.82 mmol) was added to *D*-proline methyl ester hydrochloride (632 mg, 3.82 mmol) in CH_2Cl_2 (17 mL, 0.2 M) at RT. After 1 h, EDCI•HCl (732 mg, 3.82 mmol) and 4-benzoyl benzoic acid (785 mg, 3.47 mmol) were added and the mixture was stirred overnight. The mixture was quenched with aqueous saturated $NaHCO_3$ (15 mL) and HCl 1M (15 mL) and extracted with CH_2Cl_2 (3 x 15 mL). The organic phases were collected and dried over $MgSO_4$. The solvent was removed in vacuo and the crude was used in the next step without any further purification.

In a one-necked round-bottomed flask, equipped with a stirring bar, $NaBH_4$ (197 mg, 5.20 mmol) was added portion-wise to a solution of *methyl-(4-(benzoyl)benzoyl)-D-prolinate* (1.17 g, 3.47 mmol) in methanol (14 mL, 0.2 M), at 0 °C. The reaction mixture was allowed to reach RT and monitored by TLC. When all starting material was converted, the mixture was quenched with water (15 mL) and extracted with ethyl acetate (3 x 15 mL). The organic phases were collected and dried over $MgSO_4$. The solvent was removed in vacuo and the crude product was used in the next step without any further purification.

Following **GP5**, *methyl-(4-(hydroxy(phenyl)methyl)benzoyl)-D-prolinate* (681 mg, 2.00 mmol) afforded **1ad** (519 mg, 60%) as a colorless liquid, after purification by column chromatography on silica gel (hexane/EtOAc 100:0 to 1:1). ^{1}H NMR (500 MHz, $CDCl_3$) δ 7.43 (d, *J* = 8.0 Hz, 2H, Ar_CC*H*), 7.37 (d, *J* = 8.1 Hz, 2H, Ar_CC*H*), 7.30 (d, *J* = 7.7 Hz, 2H, Ar_AC*H or* Ar_BC*H*), 7.20 (t, *J* = 7.5 Hz, 2H, Ar_AC*H or* Ar_BC*H*), 7.16 – 7.12 (m, 3H, Ar_AC*H or* Ar_BC*H*), 7.10 – 7.03 (m, 3H, Ar_AC*H or* Ar_BC*H*), 5.46

(s, 1H, SC*H*), 4.56 (dd, *J* = 8.4, 5.1 Hz, 1H, C*H*), 3.67 (s, 3H, OC*H*$_3$), 3.53 (dtd, *J* = 10.3, 7.1, 3.0 Hz, 1H, C*H*$_2$), 3.45 – 3.37 (m, 1H, C*H*$_2$), 2.21 (td, *J* = 10.1, 5.3 Hz, 1H, C*H*$_2$), 1.97 – 1.83 (m, 2H, C*H*$_2$), 1.83 – 1.72 (m, 1H, C*H*$_2$); ^{13}C NMR (126 MHz, CDCl$_3$) δ 172.8 (*C*OOCH$_3$), 169.2 (CO), 143.4 (Ar$_C$*C*), 140.4 (Ar$_A$*C* or Ar$_B$*C*), 135.6 (Ar$_A$*C* or Ar$_B$*C*), 134.9 (Ar$_C$*C*CO), 130.9 (Ar$_A$CH or Ar$_B$CH), 128.9 (Ar$_A$*C*H or Ar$_B$CH), 128.7 (Ar$_A$*C*H or Ar$_B$*C*H), 128.4 (Ar$_A$*C*H or Ar$_B$*C*H), 128.3 (Ar$_A$CH or Ar$_B$*C*H), 127.8 (Ar$_C$*C*H), 127.5 (Ar$_C$*C*H), 126.9 (Ar$_A$*C*H or Ar$_B$*C*H), 59.2 (*C*HCO), 57.3 (S*C*H), 52.3 (O*C*H$_3$), 50.0 (*C*H$_2$), 29.4 (*C*H$_2$), 25.4 (*C*H$_2$). HRMS (APCI): Calculated for $C_{26}H_{26}O_3NS$ (M-H)$^+$, 432.1616; found 432.1628.

(2*S*)-2-(6-Methoxynaphthalen-2-yl)-*N*-(4-(phenyl(phenylthiol)methyl)phenyl)propanamide 1ae

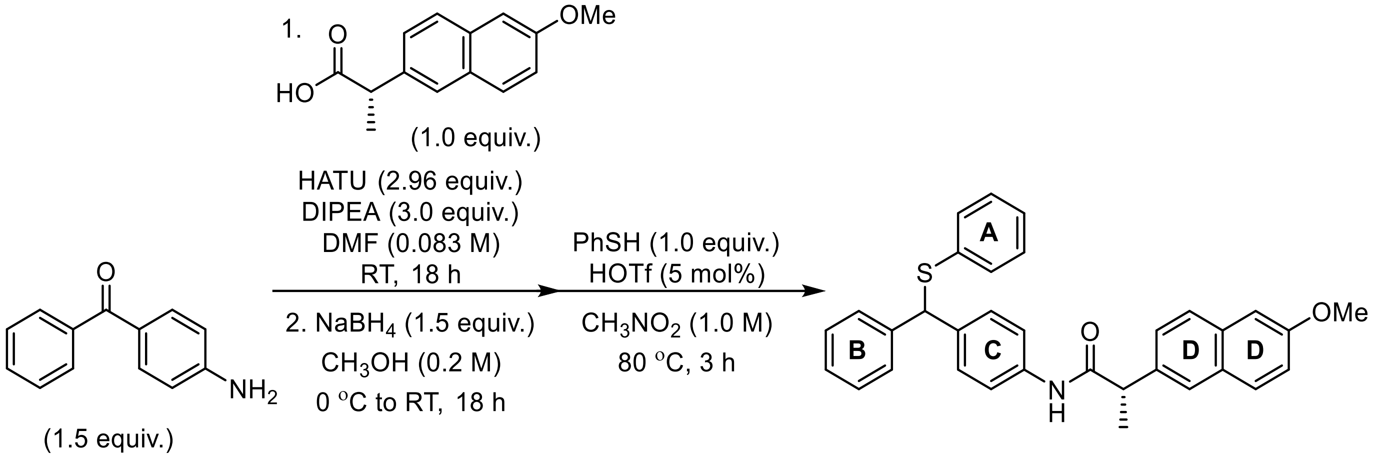

The first step was carried out according to a reported literature procedure[17] and the crude was used in the next step without any further purification. In a one-necked round-bottomed flask, equipped with a stirring bar, NaBH$_4$ (113 mg, 3.00 mmol) was added portion-wise to a solution of *(S)-N-(4-benzoylphenyl)-2-(6-methoxynaphthalen-2-yl)propanamide* (819 mg, 2.00 mmol) in methanol (8 mL, 0.2 M), at 0 °C. The reaction was allowed to reach RT and monitored by TLC. When all starting material was converted, the mixture was quenched with water (30 mL) and extracted with ethyl acetate (3 x 30 mL). The organic phases were collected and dried over MgSO$_4$. The solvent was removed in vacuo and the crude **S1ae** was used in the next step without any further purification.

Following **GP5**, *(2S)-N-(4-hydroxy(phenyl)methyl)phenyl)-2-(6-methoxynaphthalen-2-yl)propanamide* (823 mg, 2.00 mmol) afforded **1ae** (275 mg, 28%) as a orange solid, after purification

by column chromatography on silica gel (hexane/EtOAc 100:0 to 1:1). ^{1}H NMR (500 MHz, $CDCl_3$) δ 7.68 – 7.63 (m, 3H, Ar_DC*H*), 7.35 – 7.32 (m, 1H, Ar_DC*H*), 7.28 – 7.20 (m, 7H, ArC*H*), 7.19 (d, *J* = 1.1 Hz, 1H, ArC*H*), 7.17 – 7.15 (m, 1H, ArC*H*), 7.14 – 7.03 (m, 7H, Ar_DC*H* and ArC*H*), 6.95 (s, 1H, N*H*), 5.40 (s, 1H, C*H*), 3.85 (s, 3H, OCH_3), 3.75 (q, *J* = 7.1 Hz, 1H, C*H*CH_3), 1.58 (d, *J* = 7.1 Hz, 3H, CH_3); ^{13}C NMR (126 MHz, $CDCl_3$) δ 172.5 (*C*O), 158.0 (Ar_D*C*OCH_3), 141.0 (Ar*C*CH), 137.0 (Ar*C*CH), 136.9 (Ar*C*N), 136.1 (Ar*C*CH), 136.0 (Ar_D*C*CH), 134.0 (Ar_D*C*), 130.9 (Ar_D*C*), 130.6 (ArCH), 129.4 (Ar_DCH), 129.1 (ArCH), 128.9 (ArCH), 128.7 (ArCH), 128.4 (ArCH), 128.0 (Ar_DCH), 127.4 (ArCH), 126.7 (Ar_DCH), 126.5 (Ar_DCH), 126.2 (Ar_DCH), 119.8 (ArCH), 119.5 (ArCH), 105.8 (Ar_DCH), 56.9 (*C*H), 55.5 (O*C*H_3), 48.2 (*C*HCH_3), 18.6 (*C*H_3). HRMS (APCI): Calculated for $C_{33}H_{30}NO_2S$ $(M-H)^+$, 504.1992; found 504.1977.

(2,2-Dimethyl-1-phenylpropyl)(phenyl)sulfide 1af

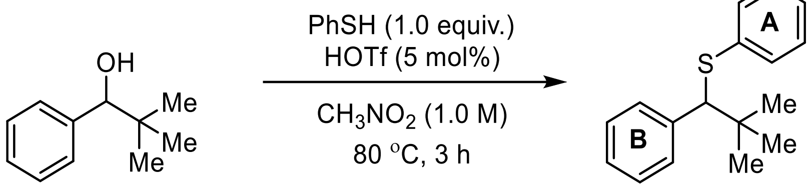

Following **GP5**, *2,2-dimethyl-1-phenylpropan-1-ol* (821 g, 5.00 mmol) afforded **1af** (535 mg, 42%) as a colorless liquid, after purification by column chromatography on silica gel (hexane/EtOAc 100:0 to 9:1). ^{1}H NMR (400 MHz, $CDCl_3$) δ 7.35 (d, *J* = 7.5 Hz, 2H, Ar_BC*H*), 7.25 – 7.15 (m, 5H, Ar_BC*H* and Ar_AC*H*), 7.14 – 7.05 (m, 3H, Ar_AC*H*), 4.02 (s, 1H, C*H*), 1.09 (s, 9H, 3 x CH_3); ^{13}C NMR (101 MHz, $CDCl_3$) δ 141.2 (Ar_B*C*), 136.9 (Ar_A*C*), 130.8 (Ar_ACH), 129.8 (Ar_BCH), 128.6 (Ar_ACH), 127.6 (Ar_BCH), 126.8 (Ar_BCH), 126.2 (Ar_ACH), 66.2 (*C*H), 36.2 (*C*$(CH_3)_3$), 28.6 (3 x *C*H_3). HRMS (APCI): Calculated for $C_{17}H_{20}SNa$ $(M-Na)^+$, 279.1178; found 279.1171.

tert-Butyl (3-(phenylthio)cyclobutyl)carbamate 1ag

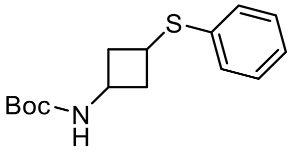

Following **GP4**, *tert-butyl (3-hydroxycyclobutyl)carbamate* (374 mg, 2.00 mmol) afforded **1ag** (475 mg, 85%, d.r. = 1.9:1) as a colorless liquid, after purification by column chromatography on deactivated silica gel (hexane/EtOAc 100:0 to 1:1). ^{1}H NMR (400 MHz, $CDCl_3$) major δ 7.30 – 7.12 (m, 5H, ArC*H*), 4.72 (brs, 1H, N*H*), 4.48 – 4.31 (m, 1H, C*H*NH), 3.82 (dddd, *J* = 12.0, 6.6, 5.7, 1.1 Hz, 1H, C*H*SPh), 2.43 – 2.35 (m, 4H, 2 x CH_2), 1.43 (s, 9H, 3 x CH_3), minor δ 7.30 – 7.12 (m, 5H, ArC*H*), 4.72 (brs, 1H, N*H*), 4.09 – 4.06 (m, 1H, C*H*NH), 3.45 (tt, *J* = 9.1, 7.3 Hz, 1H, C*H*SPh), 2.88 – 2.85 (m, 2H, CH_2), 1.95 – 1.82 (m, 2H, CH_2), 1.42 (s, 9H, 3 x CH_3); ^{13}C NMR (101 MHz, $CDCl_3$) major δ 154.8 (*C*O), 136.0 (Ar*C*), 129.8 (Ar*C*H), 129.0 (Ar*C*H), 126.1 (Ar*C*H), 79.7 (*C*CH_3), 44.4 (*C*HNH), 37.4 (2 x *C*H_2), 34.8 (*C*HSPh), 28.5 (3 x *C*H_3), minor δ 154.8 (*C*O), 136.0 (Ar*C*), 129.8 (Ar*C*H), 129.0 (Ar*C*H), 126.4 (Ar*C*H), 79.7 (*C*CH_3), 42.3 (*C*HNH) 40.0 (2 x *C*H_2), 33.2 (*C*HSPh), 28.5 (3 x *C*H_3). HRMS (APCI): Calculated for $C_{15}H_{21}NO_2SNa$ (M-Na)$^+$, 302.1185; found 302.1180.

Ethyl-3-(phenylthio)cyclobutene-1-carboxylate 1ah

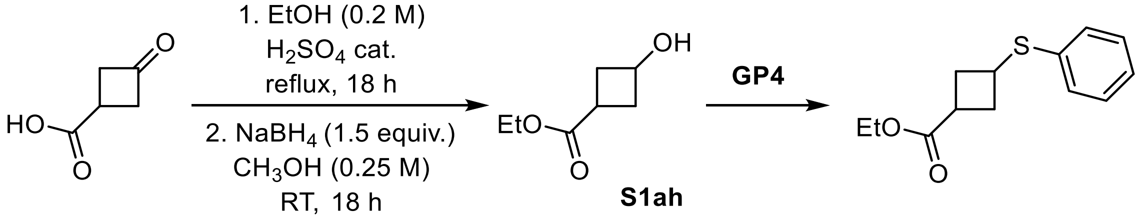

Ethyl-3-hydroxycyclobutane-1-carboxylate **S1ah** was prepared in two steps. In a one-necked round-bottomed flask, equipped with a stirring bar, cat. H_2SO_4 was added to a solution of *3-oxocyclobutane-1-carboxylic acid* (571 mg, 5.0 mmol) in ethanol (25 mL, 0.2 M) and the mixture was refluxed for 18 h. The mixture was allowed to cool to room temperature and was quenched with water (30 mL) and extracted with Et_2O (3 x 30 mL). The organic phases were collected and dried over $MgSO_4$ and the solvent was removed in vacuo. Then, in a one-necked round-bottomed flask, equipped with a stirring

bar, $NaBH_4$ (284 mg, 3.00 mmol) was added portion-wise to a solution of the crude (5.00 mmol) in methanol (20 mL, 0.25 M), at 0 °C. The reaction was allowed to reach room temperature and monitored by TLC. When all starting material was converted, the mixture was quenched with water (30 mL) and extracted with ethyl acetate (3 x 30 mL). The organic phases were collected and dried over $MgSO_4$. The solvent was removed in vacuo and the crude **S1ah** was used in the next step without any further purification.

Following **GP4**, *ethyl-3-hydroxycyclobutane-1-carboxylate* (360 mg, 2.50 mmol) afforded **1ah** (490 mg, 83%, d.r. > 20:1) as a colorless liquid, after purification by column chromatography (hexane/EtOAc 100:0 to 95:5). ^{1}H NMR (400 MHz, $CDCl_3$) δ 7.31 – 7.24 (m, 4H, ArC*H*), 7.21 – 7.17 (m, 1H, ArC*H*), 4.17 (q, *J* = 7.1 Hz, 2H, OCH_2CH$_3$), 4.02 (dddd, *J* = 12.4, 8.2, 6.0, 1.1 Hz, 1H, C*H*COCH$_2$CH$_3$), 3.29 (ttd, *J* = 9.4, 6.0, 1.1 Hz, 1H, C*H*SAr), 2.82 – 2.73 (m, 2H, CH_2), 2.35 – 2.28 (m, 2H, CH_2), 1.27 (t, *J* = 7.2 Hz, 3H, OCH$_2$CH_3); ^{13}C NMR (101 MHz, $CDCl_3$) δ 175.2 (*C*O), 136.1 (Ar*C*), 129.2 (Ar*C*H), 129.0 (Ar*C*H), 126.1 (Ar*C*H), 60.7 (O*C*H$_2$CH$_3$), 37.5 (*C*HCOCH$_2$CH$_3$), 35.3 (*C*HSAr), 32.5 (2 x CH$_2$), 14.3 (OCH$_2$*C*H$_3$). HRMS (APCI): Calculated for $C_{13}H_{17}O_2S$ $(M\text{-}H)^+$, 237.0935; found 237.0944.

4 Substrate Scope of Aminoallylation

4-Methoxy-*N*-(1-phenylbut-3-en-1-yl)aniline 3a

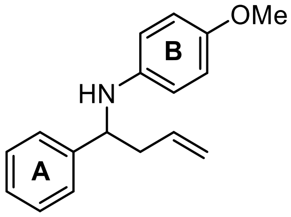

Following **GP6**, *benzyl phenyl sulfide* **1a** (60 mg, 0.30 mmol), 1,4-dinitrobenzene **A1** (101 mg, 0.60 mmol), *para*-methoxy aniline (74 mg, 0.60 mmol), potassium allyltrifluoroborate (133 mg, 0.90 mmol) and $BF_3 \bullet OEt_2$ (55 µL, 0.30 mmol) afforded **3a** (46 mg, 61%) as a liquid, after purification by column chromatography on deactivated silica gel (toluene/hexane 9:1). ^{1}H NMR (500 MHz, $CDCl_3$) δ 7.32 –

7.22 (m, 4H, Ar_ACH), 7.20 – 7.10 (m, 1H, Ar_ACH), 6.64 – 6.57 (m, 2H, Ar_BCH), 6.43 – 6.35 (m, 2H, Ar_BCH), 5.70 (dddd, *J* = 16.6, 10.2, 7.8, 6.3 Hz, 1H, CH_2=C*H*), 5.14 – 5.02 (m, 2H, CH_2=CH), 4.24 (dd, *J* = 8.1, 5.0 Hz, 1H, NC*H*), 3.86 (brs, 1H, N*H*), 3.62 (s, 3H, OCH_3), 2.57 – 2.35 (m, 2H, CH_2); ^{13}C NMR (126 MHz, $CDCl_3$) δ 152.1 (Ar_BCOCH_3), 144.0 (Ar_AC), 141.8 (Ar_BC), 134.9 (CH_2=*C*H), 128.7 (Ar_ACH), 127.1 (Ar_ACH), 126.5 (Ar_ACH), 118.4 (CH_2=CH), 114.9 (Ar_BCH), 114.8 (Ar_BCH), 58.1 (N*C*H), 55.9 (CH_3), 43.6 (CH_2). Data in accordance with the literature.[19]

***N*-(1-(4-Bromophenyl)but-3-en-1-yl)-4-methoxyaniline 3b**

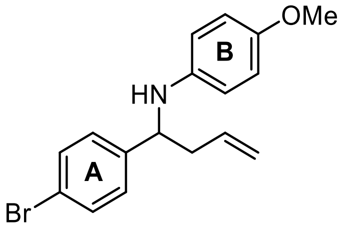

Following **GP6**, *(4-bromobenzyl)(phenyl)sulfide* **1b** (84 mg, 0.30 mmol), 1,4-dinitrobenzene **A1** (101 mg, 0.60 mmol), *para*-methoxy aniline (74 mg, 0.60 mmol), potassium allyltrifluoroborate (133 mg, 0.90 mmol) and BF_3•OEt_2 (55 µL, 0.30 mmol) afforded **3b** (55 mg, 55%) as a liquid, after purification by column chromatography on deactivated silica gel (toluene/hexane 9:1). ^{1}H NMR (500 MHz, $CDCl_3$) δ 7.47 – 7.38 (m, 2H, Ar_ACH), 7.28 – 7.22 (m, 2H, Ar_ACH), 6.74 – 6.59 (m, 2H, Ar_BCH), 6.46 – 6.39 (m, 2H, Ar_BCH), 5.75 (dddd, *J* = 16.7, 10.2, 7.8, 6.3 Hz, 1H, CH_2=C*H*), 5.23 – 5.13 (m, 2H, CH_2=CH), 4.27 (dd, *J* = 8.2, 5.0 Hz, 1H, NC*H*), 3.91 (brs, 1H, N*H*), 3.70 (s, 3H, OCH_3), 2.61 – 2.41 (m, 2H, CH_2); ^{13}C NMR (126 MHz, $CDCl_3$) δ 152.3 (Ar_BCOCH_3), 143.1 (Ar_AC), 141.4 (Ar_BC), 134.4 (CH_2=*C*H), 131.8 (Ar_ACH), 128.3 (Ar_ACH), 120.7 (Ar_ACBr), 118.8 (CH_2=CH), 114.9 (Ar_BCH), 114.8 (Ar_BCH), 57.6 (N*C*H), 55.9 (CH_3), 43.4 (CH_2). Data in accordance with the literature.[20]

N-(1-(4-Fluorophenyl)but-3-en-1-yl)-4-methoxyaniline 3c

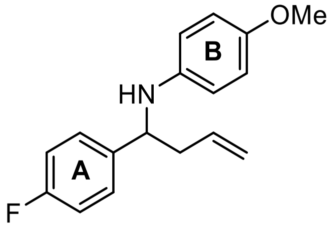

Following **GP6**, (*4-fluorobenzyl)(phenyl)sulfide* **1c** (66 mg, 0.30 mmol), 1,4-dinitrobenzene **A1** (101 mg, 0.60 mmol), *para*-methoxy aniline (74 mg, 0.60 mmol), potassium allyltrifluoroborate (133 mg, 0.90 mmol) and BF_3•OEt_2 (55 μL, 0.30 mmol) afforded **3c** (55 mg, 68%) as a liquid, after purification by column chromatography on deactivated silica gel (toluene/hexane 9:1). ^{1}H NMR (400 MHz, $CDCl_3$) δ 7.37 – 7.29 (m, 2H, Ar_AC*H*), 7.06 – 6.96 (m, 2H, Ar_AC*H*), 6.73 – 6.65 (m, 2H, Ar_BC*H*), 6.48 – 6.39 (m, 2H, Ar_BC*H*), 5.75 (dddd, *J* = 16.7, 10.1, 7.8, 6.3 Hz, 1H, CH_2=C*H*), 5.23 – 5.11 (m, 2H, CH_2=CH), 4.29 (dd, *J* = 8.1, 5.1 Hz, 1H, NC*H*), 3.93 (brs, 1H, N*H*), 3.70 (s, 3H, CH_3), 2.62 – 2.39 (m, 2H, CH_2); ^{13}C NMR (101 MHz, $CDCl_3$) δ 161.9 (d, *J* = 244.5 Hz, Ar_A*C*F), 152.2 (Ar_B*C*OCH_3), 141.5 (Ar_B*C*), 139.8 (Ar_A*C*), 134.6 (CH_2=*C*H), 127.9 (d, *J* = 8.0 Hz, Ar_A*C*H), 118.6 (*C*H_2=CH), 115.5 (d, *J* = 2.1 Hz, Ar_A*C*H), 114.9 (Ar_B*C*H), 114.8 (Ar_B*C*H), 57.5 (N*C*H), 55.8 (O*C*H_3), 43.6 (*C*H_2). Data in accordance with the literature.[21]

Methyl 4-(1-((4-methoxyphenyl)amino)but-3-en-1-yl)benzoate 3d

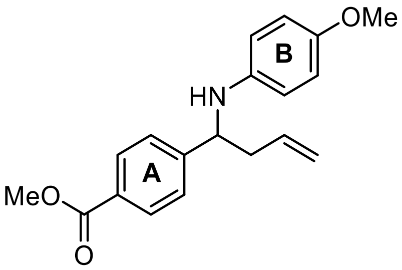

Following **GP6**, *methyl 4-((phenylthio)methyl)benzoate* **1d** (77 mg, 0.30 mmol), 1,4-dinitrobenzene **A1** (101 mg, 0.60 mmol), *para*-methoxy aniline (74 mg, 0.60 mmol), potassium allyltrifluoroborate (133 mg, 0.90 mmol) and BF_3•OEt_2 (55 μL, 0.30 mmol) afforded **3d** (57 mg, 61%) as a liquid, after

purification by column chromatography on deactivated silica gel (toluene/hexane 9:1). ^{1}H NMR (500 MHz, $CDCl_3$) δ 8.03 – 7.96 (m, 2H, Ar_AH), 7.46 – 7.41 (m, 2H, Ar_ACH), 6.70 – 6.65 (m, 2H, Ar_BCH), 6.45 – 6.35 (m, 2H, Ar_BCH), 5.79 – 5.70 (m, 1H, CH_2=C*H*), 5.24 – 5.14 (m, 2H, CH_2=CH), 4.35 (dd, *J* = 8.2, 4.9 Hz, 1H, NC*H*), 3.99 – 3.92 (brs, 1H, N*H*), 3.90 (s, 3H, CO_2CH_3), 3.68 (s, 3H, OCH_3), 2.63 – 2.43 (m, 2H, CH_2); ^{13}C NMR (126 MHz, $CDCl_3$) δ 167.1 (*C*O), 152.3 (Ar_BCOCH_3), 149.5 (Ar_AC), 141.3 (Ar_BC), 134.3 (CH_2=*C*H), 130.1 (Ar_ACH), 129.1 (Ar_ACCO), 126.5 (Ar_ACH), 118.9 (*C*H_2=CH), 114.9 (Ar_BCH), 114.8 (Ar_BCH), 58.0 (N*C*H), 55.8 (OCH_3), 52.2 (CO_2CH_3), 43.3 (*C*H_2). Data in accordance with the literature.[20]

4-(1-((4-Methoxyphenyl)amino)but-3-en-1-yl)benzonitrile 3e

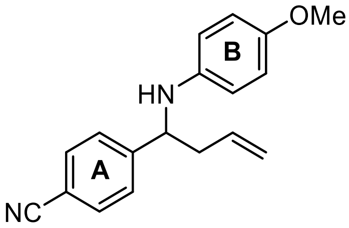

Following **GP6**, *4-((phenylthio)methyl)benzonitrile* **1e** (68 mg, 0.30 mmol), 1,4-dinitrobenzene **A1** (101 mg, 0.60 mmol), *para*-methoxy aniline (74 mg, 0.60 mmol), potassium allyltrifluoroborate (133 mg, 0.90 mmol) and BF_3•OEt_2 (55 μL, 0.30 mmol) afforded **3e** (43 mg, 53%) as a liquid, after purification by column chromatography on deactivated silica gel (toluene/hexane 9:1). ^{1}H NMR (400 MHz, $CDCl_3$) δ 7.66 – 7.56 (m, 2H, Ar_ACH), 7.53 – 7.45 (m, 2H, Ar_ACH), 6.73 – 6.64 (m, 2H, Ar_BCH), 6.43 – 6.34 (m, 2H, Ar_BCH), 5.73 (dddd, *J* = 15.9, 10.8, 7.8, 6.3 Hz, 1H, CH_2=C*H*), 5.24 – 5.14 (m, 2H, CH_2=CH), 4.34 (dd, *J* = 8.2, 4.9 Hz, 1H, NC*H*), 3.95 (s, 1H, N*H*), 3.69 (s, 3H, CH_3), 2.58 (dtt, *J* = 11.3, 6.3, 1.4 Hz, 1H, CH_2), 2.44 (dt, *J* = 14.3, 7.9 Hz, 1H, CH_2); ^{13}C NMR (101 MHz, $CDCl_3$) δ 152.5 (Ar_BCOCH_3), 149.8 (Ar_AC), 140.9 (Ar_BC), 133.9 (CH_2=*C*H), 132.6 (Ar_ACH), 127.3 (Ar_ACH), 119.3 (*C*H_2=CH), 119.1 (Ar_AC), 114.9 (Ar_BCH), 114.8 (Ar_BCH), 111.0 (*C*N), 57.9 (N*CH*), 55.8 (*C*H_3), 43.2 (*C*H_2). HRMS (APCI): Calculated for $C_{18}H_{19}ON_2$ $(M-H)^+$, 279.1492; found 279.1492.

4-Methoxy-*N*-(1-(4-(4,4,5,5-tetramethyl-1,3,2-dioxaborolan-2-yl)phenyl)but-3-en-1-yl)aniline 3f

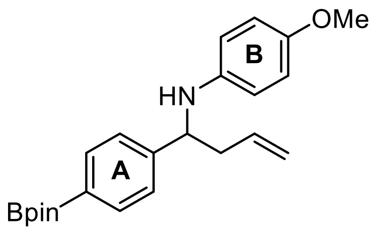

Following **GP6**, *4,4,5,5-tetramethyl-2-(4-((phenylthio)methyl)phenyl)-1,3,2-dioxaborolane* **1f** (99 mg, 0.30 mmol), 1,4-dinitrobenzene **A1** (101 mg, 0.60 mmol), *para*-methoxy aniline (74 mg, 0.60 mmol), potassium allyltrifluoroborate (133 mg, 0.90 mmol) and BF_3•OEt_2 (55 μL, 0.30 mmol) afforded **3f** (71 mg, 62%) as a liquid, after purification by column chromatography on deactivated silica gel (toluene/hexane 9:1 to hexane/EtOAc 9:1 to 3:7). ^{1}H NMR (400 MHz, $CDCl_3$) δ 7.80 – 7.74 (m, 2H, $Ar_AC\textit{H}$), 7.40 – 7.32 (m, 2H, $Ar_AC\textit{H}$), 6.70 – 6.61 (m, 2H, $Ar_BC\textit{H}$), 6.47 – 6.39 (m, 2H, $Ar_BC\textit{H}$), 5.84 – 5.68 (m, 1H, $CH_2{=}C\textit{H}$), 5.22 – 5.04 (m, 2H, $C\textit{H}_2{=}CH$), 4.31 (dd, *J* = 8.2, 5.0 Hz, 1H, NC*H*), 3.85 (s, 1H, N*H*), 3.68 (s, 3H, $OC\textit{H}_3$), 2.64 – 2.53 (m, 1H, $C\textit{H}_2$), 2.46 (dt, *J* = 14.8, 8.0 Hz, 1H, $C\textit{H}_2$), 1.33 (s, 12H, 4 x $C\textit{H}_3$); ^{13}C NMR (101 MHz, $CDCl_3$) δ 152.1 ($Ar_B\textit{C}OCH_3$), 147.4 ($Ar_A\textit{C}$), 141.7 ($Ar_B\textit{C}$), 135.2 ($Ar_A\textit{C}H$), 134.8 ($CH_2{=}\textit{C}H$), 125.9 ($Ar_A\textit{C}H$), 118.5 ($\textit{C}H_2{=}CH$), 114.8 ($Ar_B\textit{C}H$), 114.7 ($Ar_B\textit{C}H$), 83.8 (BO*C*), 58.2 (N*C*H), 55.9 ($O\textit{C}H_3$), 43.4 ($\textit{C}H_2$), 25.0 (4 x CH_3). The C directly bound to boron does not appear because of the quadrupolar moment of the B atom. ^{11}B NMR (128 MHz, $CDCl_3$) δ 30.99. HRMS (APCI): Calculated for $C_{23}H_{31}O_3NB$ $(M-H)^+$, 380.2402; found 380.2392.

4-Methoxy-*N*-(1-(4-(phenylthio)phenyl)but-3-en-1-yl)aniline 3g

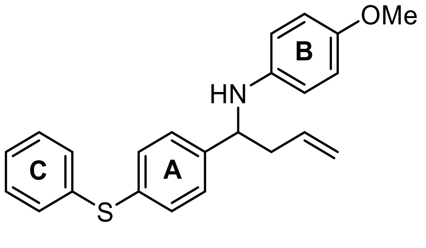

Following **GP6**, *phenyl(4-(phenylthio)benzyl)sulfide* **1g** (93 mg, 0.30 mmol), 1,4-dinitrobenzene **A1** (101 mg, 0.60 mmol), *para*-methoxy aniline (74 mg, 0.60 mmol), potassium allyltrifluoroborate (133

mg, 0.90 mmol) and BF_3•OEt_2 (55 mL, 0.30 mmol) afforded **3g** (66 mg, 61%) as a liquid, after purification by column chromatography on deactivated silica gel (toluene/hexane 9:1). ^{1}H NMR (400 MHz, $CDCl_3$) δ 7.29 – 7.10 (m, 9H, Ar_CC*H* and Ar_AC*H*), 6.65 – 6.57 (m, 2H, Ar_BC*H*), 6.41 – 6.32 (m, 2H, Ar_BC*H*), 5.68 (dddd, *J* = 16.6, 10.1, 7.8, 6.3 Hz, 1H, CH_2=C*H*), 5.15 – 5.03 (m, 2H, CH_2=CH), 4.21 (dd, *J* = 8.1, 5.0 Hz, 1H, NC*H*), 3.82 (d, *J* = 13.3 Hz, 1H, N*H*), 3.62 (s, 3H, CH_3), 2.50 (dddt, *J* = 14.2, 6.3, 5.0, 1.4 Hz, 1H, CH_2), 2.38 (dtt, *J* = 14.3, 8.0, 1.2 Hz, 1H, CH_2); ^{13}C NMR (101 MHz, $CDCl_3$) δ 152.2 (Ar_B*C*OCH_3), 143.2 (Ar_A*C*), 141.6 (Ar_B*C*), 136.0 (Ar_C*C*), 134.7 (CH_2=*C*H), 134.0 (Ar_A*C*S), 131.4 (Ar_C*C*H), 131.0 (Ar_C*C*H), 129.3 (Ar_A*C*H), 127.4 (Ar_A*C*H), 127.1 (Ar_C*C*H), 118.6 (*C*H_2=CH), 114.9 (Ar_B*C*H), 114.8 (Ar_B*C*H), 57.7 (N*C*H), 55.9 (*C*H_3), 43.4 (*C*H_2). HRMS (APCI): Calculated for $C_{23}H_{24}ONS$ $(M-H)^+$, 362.1574; found 362.1573.

4-(1-((4-Methoxyphenyl)amino)but-3-en-1-yl)-*N,N*-dipropylbenzenesulfonamide 3h

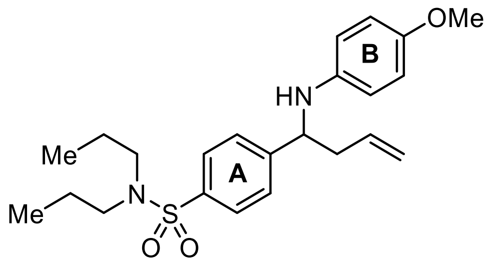

Following **GP6**, *4-((phenylthio)methyl)-N,N-dipropylbenzenesulfonamide* **1h** (109 mg, 0.30 mmol), 1,4-dinitrobenzene **A1** (101 mg, 0.60 mmol), *para*-methoxy aniline (74 mg, 0.60 mmol), potassium allyltrifluoroborate (133 mg, 0.90 mmol) and BF_3•OEt_2 (55 mL, 0.30 mmol) afforded **3h** (82 mg, 53%) as a liquid, after purification by column chromatography on deactivated silica gel (toluene/hexane 9:1). ^{1}H NMR (400 MHz, $CDCl_3$) δ 7.79 – 7.71 (m, 2H, Ar_AC*H*), 7.55 – 7.45 (m, 2H, Ar_AC*H*), 6.72 – 6.63 (m, 2H, Ar_BC*H*), 6.43 – 6.35 (m, 2H, Ar_BC*H*), 5.73 (dddd, *J* = 16.8, 10.4, 7.8, 6.3 Hz, 1H, CH_2=C*H*), 5.23 – 5.13 (m, 2H, CH_2=CH), 4.35 (dd, *J* = 8.2, 5.0 Hz, 1H, NC*H*), 3.94 (s, 1H, N*H*), 3.69 (s, 3H, OCH_3), 3.13 – 3.01 (m, 4H, 2 x NCH_2), 2.59 (dddt, *J* = 14.2, 6.4, 5.0, 1.4 Hz, 1H, CH_2), 2.52 – 2.39 (m, 1H, CH_2), 1.60 – 1.45 (m, 4H, 2 x NCH_2CH_2), 0.85 (t, *J* = 7.4 Hz, 6H, 2 x CH_3); ^{13}C NMR (101 MHz, $CDCl_3$) δ 152.4 (Ar_B*C*OCH_3), 149.0 (Ar_A*C*), 141.2 (Ar_B*C*), 139.0 (Ar_A*C*S), 134.1 (CH_2=*C*H), 127.6 (Ar_A*C*H), 127.1 (Ar_A*C*H), 119.1 (*C*H_2=CH), 114.9 (Ar_B*C*H), 119.8 (Ar_B*C*H), 57.8 (N*C*H), 55.9

(OCH_3), 50.1 (2 x NCH_2), 43.3 (CH_2), 22.1 (2 x NCH_2CH_2), 11.3 (2x CH_3). HRMS (APCI): Calculated for $C_{23}H_{33}O_3N_2S$ $(M-H)^+$, 417.2195; found 417.2206.

N-(1-(2-Iodophenyl)but-3-en-1-yl)-4-methoxyaniline 3i

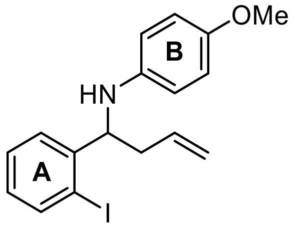

Following **GP6** but using 2 x Tuna blue lamps as light source for 5 days, without fan, *((3-iodonaphthalen-2-yl)methyl)(phenyl)sulfide* **1i** (99 mg, 0.30 mmol), 1,4-dinitrobenzene **A1** (151 mg, 0.90 mmol), *para*-methoxy aniline (74 mg, 0.60 mmol), potassium allyltrifluoroborate (133 mg, 0.90 mmol) and $BF_3{\bullet}OEt_2$ (55 μL, 0.30 mmol) afforded **3i** (68 mg, 61%) as a liquid, after purification by column chromatography on deactivated silica gel (toluene/hexane 9:1). ^1H NMR (400 MHz, $CDCl_3$) δ 7.85 (dd, J = 7.8, 1.2 Hz, 1H, Ar_ACH), 7.40 (dd, J = 7.8, 1.7 Hz, 1H, Ar_ACH), 7.30 – 7.22 (m, 1H, Ar_ACH), 6.94 (td, J = 7.6, 1.8 Hz, 1H, Ar_ACH), 6.72 – 6.64 (m, 2H, Ar_BCH), 6.41 – 6.31 (m, 2H, Ar_BCH), 5.90 – 5.76 (m, 1H, $CH_2{=}CH$), 5.27 – 5.15 (m, 2H, $CH_2{=}CH$), 4.51 (dd, J = 8.8, 3.9 Hz, 1H, NCH), 4.06 (s, 1H, NH), 3.68 (s, 3H, CH_3), 2.71 – 2.60 (m, 1H, CH_2), 2.34 – 2.22 (m, 1H, CH_2); ^{13}C NMR (101 MHz, $CDCl_3$) δ 152.2 (Ar_BCOCH_3), 144.6 (Ar_AC), 141.3 (Ar_BC), 139.9 (Ar_ACH), 134.7 ($CH_2{=}CH$), 129.0 (Ar_ACH), 128.8 (Ar_ACH), 127.7 (Ar_ACH), 118.7 ($CH_2{=}CH$), 114.9 (Ar_BCH), 114.7 (Ar_BCH), 98.8 (Ar_ACI), 61.3 (NCH), 55.9 (CH_3), 41.3 (CH_2). HRMS (ESI): Calculated for $C_{17}H_{19}ONI$ $(M-H)^+$, 380.0501; found 380.0506.

Benzyl (3-(1-((4-methoxyphenyl)amino)but-3-en-1-yl)phenyl)carbamate 3j

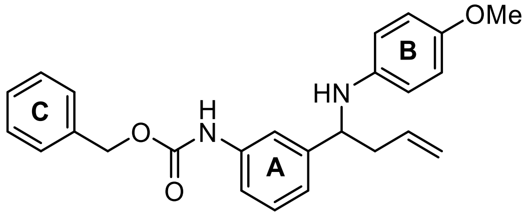

Following **GP6**, *benzyl (3-((phenylthio)methyl)phenyl)carbamate* **1j** (105 mg, 0.30 mmol), 1,4-dinitrobenzene **A1** (101 mg, 0.60 mmol), *para*-methoxy aniline (74 mg, 0.60 mmol), potassium allyltrifluoroborate (133 mg, 0.90 mmol) and BF_3•OEt_2 (55 mL, 0.30 mmol), gave **3j** (75 mg, 63%) as a liquid, after purification by column chromatography on deactivated silica gel (toluene/hexane 9:1). ^{1}H NMR (400 MHz, $CDCl_3$) δ 7.28 – 7.19 (m, 5H, Ar_CC*H*), 7.13 (dd, *J* = 7.8, 3.1 Hz, 1H, Ar_AC*H*), 6.94 (dt, *J* = 6.9, 1.6 Hz, 1H, Ar_AC*H*), 6.66 – 6.57 (m, 2H, Ar_AC*H*), 6.56 – 6.49 (m, 2H, Ar_BC*H*), 6.33 – 6.25 (m, 2H, Ar_BC*H*), 5.63 (dddd, *J* = 16.6, 10.1, 7.8, 6.2 Hz, 1H, CH_2=C*H*), 5.09 – 4.96 (m, 4H, CH_2=CH and CH_2), 4.14 (dd, *J* = 8.2, 4.9 Hz, 1H, NC*H*), 3.61 (s, 2H, 2 x N*H*), 3.55 (s, 3H, OCH_3), 2.55 – 2.22 (m, 2H, CHCH_2); ^{13}C NMR (101 MHz, $CDCl_3$) δ 153.4 (*C*O)*, 152.9 (*C*O)*, 152.1 (Ar_B*C*OCH_3), 145.3 (Ar_A*C*C), 141.6 (Ar_B*C*), 140.0 (Ar_A*C*)*, 138.2 (Ar_A*C*)*, 136.2 (Ar_C*C*), 134.8 (CH_2=*C*H), 129.4 (Ar_A*C*H), 128.7 (Ar_C*C*H), 128.4 (Ar_A*C*H), 128.3 (Ar_C*C*H), 121.6 (Ar_A*C*H), 118.4 (*C*H_2=CH), 116.5 (Ar_C*C*H), 114.9 (Ar_A*C*H), 114.8 (Ar_B*C*H), 114.7 (Ar_B*C*H), 67.0 (*C*H_2), 57.9 (N*C*H), 55.8 (O*C*H_3), 43.4 (CH*C*H_2). Some carbon atoms (highlighted with the asterisk) appear as two signals rather than one due to the presence of rotamers around the carbamate bond. HRMS (APCI): Calculated for $C_{25}H_{27}O_3N_2$ (M-H)$^+$ 403.2003; found 403.2016.

_N_-(1-(3,5-Di-_tert_-butylphenyl)but-3-en-1-yl)-4-methoxyaniline 3k

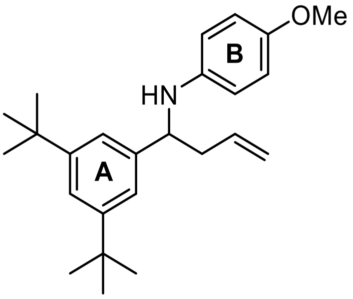

Following **GP6**, *(3,5-di-tert-butylbenzyl)(phenyl)sulfide* **1k** (94 mg, 0.30 mmol), 1,4-dinitrobenzene **A1** (101 mg, 0.60 mmol), *para*-methoxy aniline (74 mg, 0.60 mmol), potassium allyltrifluoroborate (133 mg, 0.90 mmol) and $BF_3•OEt_2$ (55 μL, 0.30 mmol) afforded **3k** (58 mg, 53%) as a liquid, after purification by column chromatography on deactivated silica gel (toluene/hexane 9:1). ^{1}H NMR (400 MHz, $CDCl_3$) δ 7.30 (t, *J* = 1.8 Hz, 1H, $Ar_AC\mathit{H}$), 7.20 (d, *J* = 1.8 Hz, 2H, $Ar_AC\mathit{H}$), 6.71 (d, *J* = 8.9 Hz, 2H, $Ar_BC\mathit{H}$), 6.51 (d, *J* = 8.9 Hz, 2H, $Ar_BC\mathit{H}$), 5.86 – 5.76 (m, 1H, CH_2=C*H*), 5.25 – 5.12 (m, 2H, $C\mathit{H}_2$=CH), 4.30 (dd, *J* = 8.3, 5.0 Hz, 1H, NC*H*), 3.72 (s, 3H, $C\mathit{H}_3$), 2.72 – 2.45 (m, 2H, $C\mathit{H}_2$), 1.33 (s, 18H, 6 x $C\mathit{H}_3$), 1.28 (s, 1H, N*H*) ; ^{13}C NMR (101 MHz, $CDCl_3$) δ 152.0 ($Ar_B\mathit{C}OCH_3$), 150.8 ($Ar_A\mathit{C}t$Bu), 142.9 ($Ar_A\mathit{C}$), 142.2 ($Ar_B\mathit{C}$), 135.5 (CH_2=CH), 120.9 ($Ar_A\mathit{C}H$), 120.7 ($Ar_A\mathit{C}H$), 118.0 ($\mathit{C}H_2$=CH), 115.0 ($Ar_B\mathit{C}H$), 114.7 ($Ar_B\mathit{C}H$), 58.9 (N*C*H), 55.8 (O*C*H_3), 43.4 ($\mathit{C}H_2$), 35.0 ($\mathit{C}(CH_3)_3$), 31.7 (6 x $\mathit{C}H_3$). HRMS (ESI): Calculated for $C_{25}H_{36}ON$ $(M-H)^+$, 366.2787; found 366.2791.

2-Methoxy-4-(1-((4-methoxyphenyl)amino)but-3-en-1-yl)phenyl acetate 3l

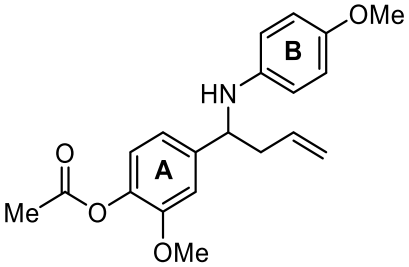

Following **GP6**, *2-methoxy-4-((phenylthio)methyl)phenyl acetate* **1l** (87 mg, 0.30 mmol), 1,4-dinitrobenzene **A1** (101 mg, 0.60 mmol), *para*-methoxy aniline (74 mg, 0.60 mmol), potassium allyltrifluoroborate (133 mg, 0.90 mmol) and $BF_3•OEt_2$ (55 mL, 0.30 mmol) afforded **3l** (51 mg, 50%)

as a liquid, after purification by column chromatography on deactivated silica gel (toluene/hexane 9:1 to hexane/EtOAc 9/1). ^{1}H NMR (400 MHz, $CDCl_3$) δ 7.00 – 6.91 (m, 3H, Ar_AC*H*), 6.74 – 6.65 (m, 2H, Ar_BC*H*), 6.50 – 6.42 (m, 2H, Ar_BC*H*), 5.78 (dddd, *J* = 16.4, 10.1, 7.9, 6.1 Hz, 1H, CH_2=C*H*), 5.25 – 5.12 (m, 2H, CH_2=CH), 4.26 (dd, *J* = 8.4, 4.8 Hz, 1H, NC*H*), 3.89 (s, 1H, N*H*), 3.80 (s, 3H, Ar_AOCH_3), 3.70 (s, 3H, Ar_BOCH_3), 2.65 – 2.52 (m, 1H, CH_2=CH), 2.45 (dt, *J* = 14.1, 8.2 Hz, 1H, CH_2=CH), 2.30 (s, 3H, C(O)CH_3); ^{13}C NMR (101 MHz, $CDCl_3$) δ 169.2 (*C*O), 152.2 (Ar_B*C*OCH_3), 151.3 (Ar_A*C*OCH_3), 143.1 (Ar_A*C*CH), 141.7 (Ar_B*C*), 138.6 (Ar_A*C*OAc), 134.8 (CH_2=*C*H), 122.8 (Ar_A*C*H), 118.5 (Ar_A*C*H), 118.4 (*C*H_2=CH), 114.9 (Ar_B*C*H), 114.8 (Ar_B*C*H), 110.4 (Ar_A*C*H), 58.1 (N*C*H), 56.0 (*C*H_3), 55.9 (*C*H_3), 43.6 (*C*H_2), 20.8 (Ar_AC(O)*C*H_3). HRMS (APCI): Calculated for $C_{20}H_{23}O_4NNa$ (M-Na)$^+$, 364.1518; found 364.1519.

4-Methoxy-*N*-(1-(naphthalen-2-yl)but-3-en-1-yl)aniline 3m

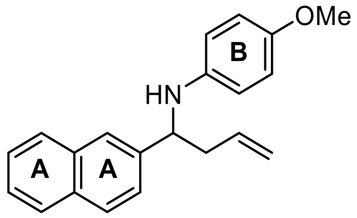

Following **GP6**, *(naphthalen-2-ylmethyl)(phenyl)sulfide* **1m** (75 mg, 0.30 mmol), 1,4-dinitrobenzene **A1** (101 mg, 0.60 mmol), *para*-methoxy aniline (74 mg, 0.60 mmol), potassium allyltrifluoroborate (133 mg, 0.90 mmol) and BF_3•OEt_2 (55 μL, 0.30 mmol) afforded **3m** (55 mg, 60%) as a liquid, after purification by column chromatography on deactivated silica gel (toluene/hexane 9:1). ^{1}H-NMR (400 MHz, $CDCl_3$) δ 7.86 – 7.77 (m, 4H, Ar_AC*H*), 7.54 – 7.42 (m, 3H, Ar_AC*H*), 6.70 – 6.64 (m, 2H, Ar_BC*H*), 6.54 – 6.49 (m, 2H, Ar_BC*H*), 5.81 (dddd, *J* = 16.6, 10.1, 7.8, 6.2 Hz, 1H, CH_2=C*H*), 5.25 – 5.13 (m, 2H, CH_2=CH), 4.48 (dd, *J* = 8.1, 5.0 Hz, 1H, NC*H*), 3.68 (s, 3H, CH_3), 2.73 – 2.52 (m, 2H, CH_2); ^{13}C-NMR (101 MHz, $CDCl_3$) δ 152.2 (Ar_B*C*OCH_3), 141.7 (Ar_A*C*), 141.5 (Ar_B*C*), 134.9 (CH_2=*C*H), 133.7 (Ar_A*C*), 132.9 (Ar_A*C*), 128.5 (Ar_ACH), 128.0 (Ar_ACH), 127.8 (Ar_A*C*H), 126.1 (Ar_A*C*H), 125.6 (Ar_A*C*H), 125.1 (Ar_A*C*H), 124.8 (Ar_A*C*H), 118.5 (*C*H_2=CH), 114.9 (2 x Ar_BCH), 58.4 (*C*NH), 55.8 (*C*H_3), 43.5 (*C*H_2). Data in accordance with the literature.[20]

4-Methoxy-*N*-(1-(thiophen-2-yl)but-3-en-1-yl)aniline 3n

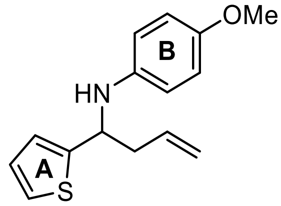

Following **GP6**, *2-((phenylthio)methyl)thiophene* **1n** (63 mg, 0.30 mmol), 1,4-dinitrobenzene **A1** (101 mg, 0.60 mmol), *para*-methoxy aniline (74 mg, 0.60 mmol), potassium allyltrifluoroborate (133 mg, 0.90 mmol) and BF_3•OEt_2 (55 μL, 0.30 mmol) afforded **3n** (49 mg, 64%) as a liquid, after purification by column chromatography on deactivated silica gel (toluene/hexane 9:1). ^{1}H NMR (400 MHz, $CDCl_3$) δ 7.17 (dd, *J* = 4.9, 1.4 Hz, 1H, Ar_AC*H*), 7.02 – 6.92 (m, 2H, Ar_AC*H*), 6.78 – 6.69 (m, 2H, Ar_BC*H*), 6.64 – 6.52 (m, 2H, Ar_BC*H*), 5.82 (ddt, *J* = 17.1, 10.1, 7.0 Hz, 1H, CH_2=C*H*), 5.24 – 5.12 (m, 2H, CH_2=CH), 4.67 – 4.57 (m, 1H, NC*H*), 3.89 (d, *J* = 8.2 Hz, 1H, N*H*), 3.72 (s, 3H, CH_3), 2.74 – 2.56 (m, 2H, CH_2); ^{13}C NMR (101 MHz, $CDCl_3$) δ 152.6 (Ar_B*C*OCH_3), 149.2 (Ar_A*C*), 141.4 (Ar_B*C*), 134.3 (CH_2=*C*H), 126.9 (Ar_A*C*H), 123.7 (Ar_A*C*H), 123.5 (Ar_A*C*H), 118.7 (*C*H_2=CH), 115.2 (Ar_B*C*H), 114.9 (Ar_B*C*H), 55.8 (CH_3), 54.5 (N*C*H), 43.4 (*C*H_2). HRMS (APCI): Calculated for $C_{15}H_{18}ONS$ $(M-H)^+$, 260.1100 found; 260.1104.

***N*-(2,2-Dimethylhex-5-en-3-yl)-4-methoxyaniline 3o**

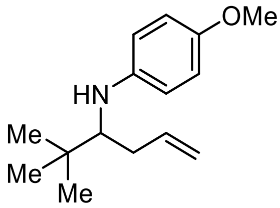

Following **GP6**, *neopentyl(phenyl)sulfide* **1o** (54 mg, 0.30 mmol), 1,4-dinitrobenzene **A1** (101 mg, 0.60 mmol), *para*-methoxy aniline (74 mg, 0.60 mmol), potassium allyltrifluoroborate (133 mg, 0.90 mmol) and BF_3•OEt_2 (55 μL, 0.30 mmol) afforded **3o** (21 mg, 32%) as a liquid, after purification by column chromatography on deactivated silica gel (toluene/hexane 9:1). ^{1}H NMR (400 MHz, $CDCl_3$) δ 6.77 – 6.69 (m, 2H, ArC*H*), 6.58 – 6.49 (m, 2H, ArC*H*), 5.80 (ddt, *J* = 17.0, 10.1, 6.9 Hz,

1H, CH_2=C*H*), 5.08 – 4.91 (m, 2H, C*H*$_2$=CH), 3.73 (s, 3H, OC*H*$_3$), 3.18 (brs, 1H, N*H*), 3.05 (dd, *J* = 9.8, 3.5 Hz, 1H, NC*H*), 2.54 – 2.43 (m, 1H, C*H*$_2$), 2.03 (ddd, *J* = 15.3, 9.3, 7.1 Hz, 1H, C*H*$_2$), 0.96 (s, 9H, 3 x C*H*$_3$); ^{13}C NMR (101 MHz, $CDCl_3$) δ 151.4 (Ar*C*OCH$_3$), 144.2 (Ar*C*NH), 137.3 (CH_2=*C*H), 116.3 (*C*H$_2$=CH), 115.0 (Ar*C*H), 114.1 (Ar*C*H), 63.2 (N*C*H), 56.0 (O*C*H$_3$), 36.7 (*C*H$_2$), 36.1 (*C*(CH_3)$_3$), 27.1 (3 x *C*H$_3$). Data in accordance with the literature.[21]

N-(1-((3,5,7)-Adamantan-1-yl)but-3-en-1-yl)-4-methoxyaniline 3p

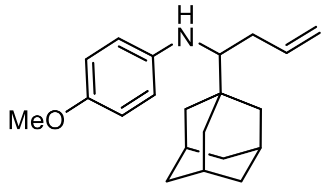

Following **GP6**, *adamantan-1-yl(methyl)(phenyl)sulfide* **1p** (78 mg, 0.30 mmol), 1,4-dinitrobenzene **A1** (101 mg, 0.60 mmol), *para*-methoxy aniline (74 mg, 0.60 mmol), potassium allyltrifluoroborate (133 mg, 0.90 mmol) and BF_3•OEt_2 (55 μL, 0.30 mmol) afforded **3p** (35 mg, 38%) as an orange liquid, after purification by column chromatography on deactivated silica gel (toluene/hexane 7:3). ^{1}H NMR (400 MHz, $CDCl_3$) δ 6.76 – 6.68 (m, 2H, ArC*H*), 6.54 (d, *J* = 8.3 Hz, 2H, ArC*H*), 5.78 (ddt, *J* = 17.0, 10.1, 6.9 Hz, 1H, CH_2=C*H*), 5.08 – 4.90 (m, 2H, C*H*$_2$=CH), 3.73 (s, 3H, OC*H*$_3$), 3.19 (s, 1H, N*H*), 2.88 (dd, *J* = 10.0, 3.4 Hz, 1H, NC*H*), 2.50 – 2.47 (m, 1H, C*H*$_2$CH=CH$_2$), 2.08 – 1.95 (m, 3H, C*H*$_2$CH=CH$_2$, C*H*$_2$ and C*H*), 1.77 – 1.52 (m, 13H, C*H*$_2$ and C*H*); ^{13}C NMR (101 MHz, $CDCl_3$) δ 151.2 (Ar*C*OCH$_3$), 144.4 (Ar*C*), 137.5 (CH_2=*C*H), 116.3 (*C*H$_2$=CH), 115.0 (Ar*C*H), 114.0 (Ar*C*H), 63.6 (N*C*H), 56.0 (O*C*H$_3$), 39.3 (*C*H$_2$ and *C*H), 37.9 (*C*), 37.3 (*C*H$_2$ and *C*H), 35.0 (*C*H$_2$CH=CH$_2$), 28.7 (*C*H$_2$ and *C*H). HRMS (APCI): Calculated for $C_{21}H_{30}NO$ (M-H)$^+$, 312.2322; found 312.2319.

***N*-(1-Cyclopropylbut-3-en-1-yl)-4-methoxyaniline 3q**

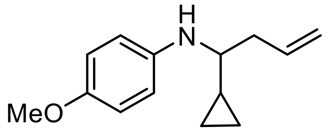

Following **GP6**, *cyclopropylmethyl(phenyl)sulfide* **1q** (49 mg, 0.30 mmol), 1,4-dinitrobenzene **A1** (101 mg, 0.60 mmol), *para*-methoxy aniline (74 mg, 0.60 mmol), potassium allyltrifluoroborate (133 mg, 0.90 mmol) and BF_3•OEt_2 (55 μL, 0.30 mmol) afforded **3q** (25 mg, 38%) as a yellow liquid, after purification by column chromatography on deactivated silica gel (toluene/hexane 9:1 then hexane/EtOAc 9:1). ^{1}H NMR (600 MHz, $CDCl_3$) δ 6.77 – 6.72 (m, 2H, ArC*H*), 6.59 – 6.53 (m, 2H, ArC*H*), 5.95 – 5.87 (m, 1H, CH_2=C*H*), 5.13 – 5.04 (m, 2H, CH_2=CH), 3.74 (s, 3H, OCH_3), 2.81 (dt, *J* = 7.6, 5.7 Hz, 1H, NC*H*), 2.42 – 2.34 (m, 2H, CH_2CH=CH_2), 1.29 – 1.25 (m, 1H, C*H*), 0.56 – 0.43 (m, 2H, CH_2), 0.31 – 0.22 (m, 2H, CH_2); ^{13}C NMR (151 MHz, $CDCl_3$) δ 152.1 (Ar*C*), 142.4 (Ar*C*), 135.3 (CH_2=*C*H), 117.4 (*C*H_2=CH), 115.2 (ArC*H*), 114.9 (ArC*H*), 57.9 (N*C*H), 55.9 (O*C*H_3), 39.8 (*C*H_2CH=CH_2), 16.2 (*C*H), 3.4 (*C*H_2), 2.9 (*C*H_2). HRMS (APCI): Calculated for $C_{14}H_{20}NO$ $(M\text{-}H)^+$, 218.1533; found 218.1539.

***N*-(1-(4-Fluorophenyl)but-3-en-1-yl)-4-morpholinoaniline 3r**

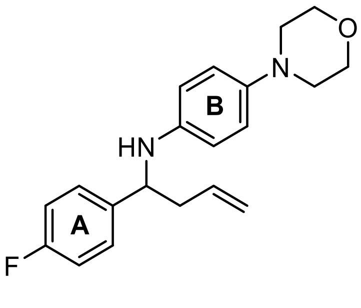

Following **GP6**, *(4-fluorobenzyl)(phenyl)sulfide* **1c** (66 mg, 0.30 mmol), 1,4-dinitrobenzene **A1** (101 mg, 0.60 mmol), 4-morpholinoaniline (107 mg, 0.60 mmol), potassium allyltrifluoroborate (133 mg, 0.90 mmol) and BF_3•OEt_2 (55 μL, 0.30 mmol) afforded **3r** (59 mg, 61%) as a brown liquid, after purification by column chromatography on deactivated silica gel (hexane/EtOAc 9:1). ^{1}H NMR (400 MHz, $CDCl_3$) δ 7.36 – 7.28 (m, 2H, Ar_AC*H*), 7.05 – 6.95 (m, 2H, Ar_AC*H*), 6.77 – 6.69 (m, 2H, Ar_BC*H*), 6.49 – 6.40 (m, 2H, Ar_BC*H*), 5.74 (dddd, *J* = 16.7, 10.2, 7.8, 6.3 Hz, 1H, CH_2=C*H*), 5.22 – 5.09 (m,

2H, CH_2=CH), 4.30 (dd, *J* = 8.1, 5.1 Hz, 1H, NC*H*), 3.93 (s, 1H, N*H*), 3.89 – 3.78 (m, 4H, C*H*$_2$), 3.00 – 2.91 (m, 4H, C*H*$_2$), 2.60 – 2.39 (m, 2H, CHC*H*$_2$); ^{13}C NMR (101 MHz, $CDCl_3$) δ 161.9 (d, *J* = 244.5 Hz, Ar$_A$*C*F), 143.7 (Ar$_B$*C*N), 141.8 (Ar$_B$*C*NH), 139.6 (d, *J* = 3.0 Hz, Ar$_A$*C*), 134.6 (CH_2=*C*H), 127.9 (d, *J* = 8.1 Hz, Ar$_A$*C*H), 118.6 (*C*H_2=CH), 118.3 (Ar$_B$*C*H), 115.5 (d, *J* = 21.3 Hz, Ar$_A$*C*H), 114.6 (Ar$_B$*C*H), 67.2 (*C*H_2), 57.2 (N*C*H), 51.2 (*C*H_2), 43.6 (CH*C*H_2); ^{19}F NMR (471 MHz, $CDCl_3$) δ -116.18. HRMS (APCI): Calculated for $C_{20}H_{24}ON_2F$ $(M-H)^+$, 327.1856; found 327.1867.

4-Cyclohexyl-*N*-(1-(4-fluorophenyl)but-3-en-1-yl)aniline 3s

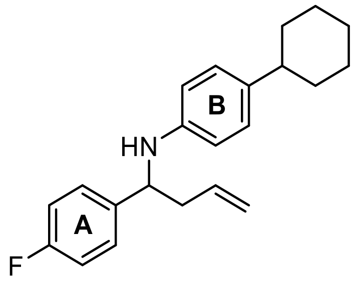

Following **GP6**, *(4-fluorobenzyl)(phenyl)sulfide* **1c** (66 mg, 0.30 mmol), 1,4-dinitrobenzene **A1** (101 mg, 0.60 mmol), 4-cyclohexylaniline (105 mg, 0.60 mmol), potassium allyltrifluoroborate (133 mg, 0.90 mmol) and BF_3•OEt_2 (55 μL, 0.30 mmol) afforded **3s** (63 mg, 65%) as a yellow liquid, after purification by column chromatography on deactivated silica gel (hexane/EtOAc 95:5 to 9:1). ^{1}H NMR (400 MHz, $CDCl_3$) δ 7.39 – 7.31 (m, 2H, Ar$_A$C*H*), 7.01 (t, *J* = 8.7 Hz, 2H, Ar$_A$C*H*), 6.94 (d, *J* = 8.1 Hz, 2H, Ar$_B$C*H*), 6.42 (d, *J* = 8.3 Hz, 2H, Ar$_B$C*H*), 5.83 – 5.67 (m, 1H, CH_2=C*H*), 5.20 – 5.13 (m, 2H, C*H*$_2$=CH), 4.32 (dd, *J* = 8.2, 5.0 Hz, 1H, NC*H*), 4.00 (brs, 1H, N*H*), 2.56 (dt, *J* = 11.8, 5.6 Hz, 1H, CHC*H*$_2$), 2.44 (dd, *J* = 14.6, 7.8 Hz, 1H, CHC*H*$_2$), 2.34 (q, *J* = 8.5 Hz, 1H, cHex-C*H*), 1.85 – 1.69 (m, 5H, cHex-C*H*$_2$), 1.43 – 1.18 (m, 5H, cHex-C*H*$_2$); ^{13}C NMR (101 MHz, $CDCl_3$) δ 162.0 (d, *J* = 244.5 Hz, Ar$_A$*C*F), 145.4 (Ar$_B$*CcHex*), 139.7 (d, *J* = 3.2 Hz, Ar$_A$*C*), 137.6 (Ar$_B$*C*), 134.6 (CH_2=*C*H), 127.9 (d, *J* = 7.9 Hz, Ar$_A$*C*H), 127.5 (Ar$_B$*C*H), 118.6 (*C*H_2=CH), 115.5 (d, *J* = 21.2 Hz, Ar$_A$*C*H), 113.6 (Ar$_B$*C*H), 57.0 (N*C*H), 44.0 (cHex-*C*H), 43.7 (CH*C*H_2), 34.8 (cHex-*C*H_2), 27.1 (cHex-*C*H_2), 26.3 (cHex-*C*H_2); ^{19}F NMR (471 MHz, $CDCl_3$) δ -116.20. HRMS (APCI): Calculated for $C_{22}H_{27}NF$ $(M-H)^+$, 324.2113; found 324.2122.

3-Benzyl-*N*-(1-(4-fluorophenyl)but-3-en-1-yl)aniline 3t

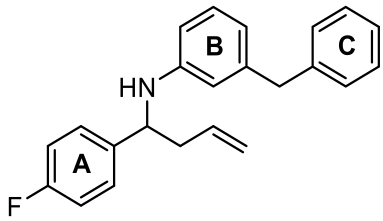

Following **GP6**, *(4-fluorobenzyl)(phenyl)sulfide* **1c** (66 mg, 0.30 mmol), 1,4-dinitrobenzene **A1** (101 mg, 0.60 mmol), 3-benzylaniline (110 mg, 0.60 mmol), potassium allyltrifluoroborate (133 mg, 0.90 mmol) and BF_3•OEt_2 (55 μL, 0.30 mmol) afforded **3t** (60 mg, 61%) as a yellow liquid, after purification by column chromatography on deactivated silica gel (hexane/EtOAc 95:5 to 9:1). ^{1}H NMR (400 MHz, $CDCl_3$) δ 7.33 – 7.18 (m, 4H, Ar_CC*H* and Ar_AC*H*), 7.11 (dd, *J* = 6.9, 1.8 Hz, 2H, Ar_CC*H*), 6.99 (tt, *J* = 8.7, 5.6 Hz, 4H, Ar_BC*H* and Ar_CC*H* and Ar_AC*H*), 6.52 (d, *J* = 7.5 Hz, 1H, Ar_BC*H*), 6.31 (d, *J* = 7.7 Hz, 2H, Ar_BC*H*), 5.81 – 5.68 (m, 1H, CH_2=C*H*), 5.21 – 5.12 (m, 2H, CH_2=CH), 4.33 (dd, *J* = 7.9, 5.3 Hz, 1H, NC*H*), 4.10 (brs, 1H, N*H*), 3.83 (s, 2H, CH_2), 2.62 – 2.40 (m, 2H, CHCH_2); ^{13}C NMR (101 MHz, $CDCl_3$) δ 161.9 (d, *J* = 244.5 Hz, Ar_A*C*F), 147.3 (Ar_B*C*N), 142.2 (Ar_B*C*CH_2), 141.2 (Ar_CC), 139.3 (d, *J* = 3.0 Hz, Ar_AC), 134.4 (CH_2=*C*H), 129.2 (Ar_BCH), 129.0 (Ar_CCH), 128.5 (Ar_CCH), 127.9 (d, *J* = 7.9 Hz, Ar_ACH), 126.0 (Ar_CCH), 118.5 (*C*H_2=CH), 118.4 (Ar_BCH), 115.50 (d, *J* = 21.5 Hz, Ar_ACH), 114.4 (Ar_BCH), 111.4 (Ar_BCH), 56.7 (N*C*H), 43.4 (CH*C*H_2), 42.1 (*C*H_2); ^{19}F NMR (471 MHz, $CDCl_3$) δ -116.18. HRMS (APCI): Calculated for $C_{23}H_{23}NF$ $(M-H)^+$, 332.1799; found 332.1801.

_N_-(1-(4-Fluorophenyl)but-3-en-1-yl)-4-(4,4,5,5-tetramethyl-1,3,2-dioxaborolan-2-yl)aniline 3u

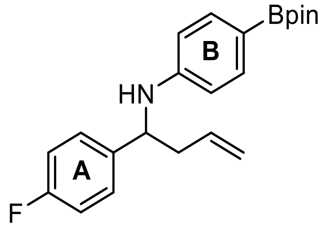

Following **GP6**, *(4-fluorobenzyl)(phenyl)sulfide* **1c** (66 mg, 0.30 mmol), 1,4-dinitrobenzene **A1** (101 mg, 0.60 mmol), 4-(4,4,5,5-tetramethyl-1,3,2-dioxaborolan-2-yl)aniline (131 mg, 0.60 mmol), potassium allyltrifluoroborate (133 mg, 0.90 mmol) and BF_3•OEt_2 (55 μL, 0.30 mmol) afforded **3u** (70 mg, 64%) as an orange liquid, after purification by column chromatography on deactivated silica gel (toluene/hexane 9:1 to toluene 100%). ^{1}H NMR (400 MHz, $CDCl_3$) δ 7.60 – 7.49 (m, 2H, Ar_BC*H*), 7.35 – 7.22 (m, 2H, Ar_AC*H*), 7.01 (dt, *J* = 17.4, 8.7 Hz, 2H, Ar_AC*H*), 6.50 – 6.39 (m, 2H, Ar_BC*H*), 5.82 – 5.66 (m, 1H, CH_2=C*H*), 5.22 – 5.11 (m, 2H, CH_2=CH), 4.44 (dd, *J* = 7.7, 5.2 Hz, 1H, NC*H*), 4.34 (s, 1H, N*H*), 2.68 – 2.40 (m, 2H, CH_2), 1.29 (s, 12H, 4 x CH_3); ^{13}C NMR (101 MHz, $CDCl_3$) δ 162.0 (d, *J* = 244.8 Hz, Ar_A*C*F), 149.6 (Ar_B*C*N), 138.8 (d, *J* = 3.0 Hz, Ar_A*C*), 136.3 (Ar_B*C*H), 134.2 (CH_2=*C*H), 127.9 (d, *J* = 7.9 Hz, Ar_A*C*H), 127.6 (d, *J* = 8.0 Hz, Ar_B*C*), 118.8 (*C*H_2=CH), 115.5 (d, *J* = 21.1 Hz, Ar_A*C*H), 112.8 (Ar_B*C*H), 83.3 (*C*$(CH_3)_2$), 56.0 (N*C*H), 43.2 (*C*H_2), 25.0 (2 x *C*H_3);, 24.9 (2 x *C*H_3); ^{11}B NMR (128 MHz, $CDCl_3$) δ 30.70; ^{19}F NMR (471 MHz, CDCl3) δ -115.97. HRMS (APCI): Calculated for $C_{22}H_{27}BNO_2F$ $(M\text{-}H)^+$, 368.2183; found 368.2192.

5 Substrate Scope of Olefination

Methyl cinnamate 4a

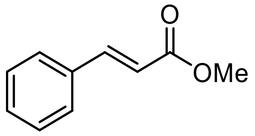

Following **GP7**, *benzyl phenyl sulfide* **1a** (60 mg, 0.30 mmol), 1,4-dinitrobenzene **A1** (101 mg, 0.60 mmol), and methyl (triphenylphosphoranylidene)acetate (201 mg, 0.60 mmol) afforded **4a** (35 mg, 71%, E/Z > 20:1) as a white solid, after purification by column chromatography on silica gel (hexane/EtOAc 9:1). ^{1}H NMR (400 MHz, $CDCl_3$) δ 7.70 (d, *J* = 16.0 Hz, 1H, C*H*=CH), 7.54 – 7.50 (m, 2H, ArC*H*), 7.40 – 7.37 (m, 3H, ArC*H*), 6.45 (d, *J* = 16.0 Hz, 1H, CH=C*H*CO), 3.81 (s, 3H, C*H*$_3$); ^{13}C NMR (101 MHz, $CDCl_3$) δ 167.6 (*C*O), 145.0 (*C*H=CH), 134.4 (Ar*C*), 130.4 (Ar*C*H), 129.0 (Ar*C*H), 128.2 (Ar*C*H), 117.8 (CH=*C*HCO), 51.8 (*C*H$_3$). Data in accordance with the literature.[22]

Methyl 3-(4-(2-hydroxypropan-2-yl)phenyl)acrylate 4v

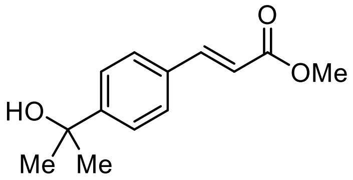

Following **GP7**, *2-(4-((phenylthio)methyl)phenyl)propan-2-ol* **1v** (78 mg, 0.30 mmol), 1,4-dinitrobenzene **A1** (101 mg, 0.60 mmol), and methyl (triphenylphosphoranylidene)acetate (201 mg, 0.60 mmol) afforded **4v** (48 mg, 72%, *E/Z* 12:1) as an orange liquid, after purification by column chromatography on silica gel (hexane/EtOAc 9:1 to 1:1). ^{1}H NMR (400 MHz, $CDCl_3$) δ 7.68 (d, *J* = 16.1 Hz, 1H, C*H*=CH), 7.55 – 7.43 (m, 4H, ArC*H*), 6.43 (d, *J* = 16.0 Hz, 1H, CH=C*H*CO), 3.80 (s, 3H, OC*H*$_3$), 1.83 (s, 1H, O*H*), 1.59 (s, 6H, 2 x C*H*$_3$); ^{13}C NMR (101 MHz, $CDCl_3$) δ 167.7 (*C*O), 151.7 (Ar*C*), 144.7 (*C*H=CH), 132.9 (Ar*C*CH), 128.2 (Ar*C*H), 124.3 (Ar*C*H), 117.6 (CH=*C*HCO), 72.6 (Ar*C*(OH)), 51.8 (O*C*H$_3$), 31.8 (2 x *C*H$_3$). HRMS (ESI): Calculated for $C_{13}H_{16}O_3N$ (M-Na)$^+$, 243.0991; found 243.0992.

Methyl 3-(4-benzoylphenyl)acrylate 4w

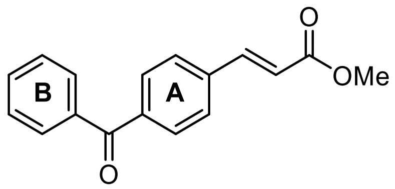

Following **GP7**, *phenyl(4-((phenylthio)methyl)phenyl)methanone* **1w** (91 mg, 0.30 mmol), 1,4-dinitrobenzene **A1** (101 mg, 0.60 mmol), and methyl (triphenylphosphoranylidene)acetate (201 mg, 0.60 mmol) afforded **4w** (54 mg, 68%, *E/Z* 10:1) as a yellow solid, after purification by column chromatography on silica gel (toluene/hexane 1:1). ^{1}H NMR (400 MHz, $CDCl_3$) δ 7.84 – 7.77 (m, 4H, $Ar_AC\mathit{H}$), 7.74 (d, J = 16.0 Hz, 1H, C*H*=CH), 7.69 – 7.55 (m, 3H, $Ar_BC\mathit{H}$), 7.53 – 7.44 (m, 2H, $Ar_BC\mathit{H}$), 6.54 (d, J = 16.0 Hz, 1H, CH=C*H*CO), 3.83 (s, 3H, $C\mathit{H}_3$); ^{13}C NMR (101 MHz, $CDCl_3$) δ 196.0 (*C*O), 167.1 ($\mathit{C}O_2CH_3$), 143.6 (*C*H=CH), 138.9 (Ar*C*), 138.2 (Ar_ACCH), 137.4 (Ar*C*), 132.8 (Ar_B*C*H), 130.7 (Ar_A*C*H), 130.1 (Ar_A*C*H), 128.5 (Ar_B*C*H), 128.0 (Ar_B*C*H), 120.3 (CH=*C*HCO), 52.0 ($\mathit{C}H_3$). Data in accordance with the literature.[23]

Methyl 3-(4-(pyridin-2-yl)phenyl)acrylate 4x

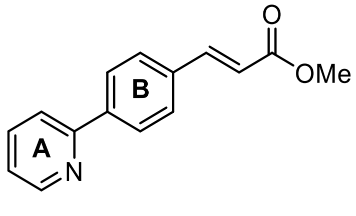

Following **GP7**, *2-(4-((phenylthio)methyl)phenyl)pyridine* **1x** (84 mg, 0.30 mmol), 1,4-dinitrobenzene **A1** (101 mg, 0.60 mmol), and methyl (triphenylphosphoranylidene)acetate (201 mg, 0.60 mmol) afforded **4x** (49 mg, 68%, *E/Z* 14:1) as a brown liquid, after purification by column chromatography on silica gel (hexane/EtOAc 9:1). ^{1}H NMR (400 MHz, $CDCl_3$) δ 8.70 (d, J = 4.7 Hz, 1H, $Ar_AC\mathit{H}$), 8.07 – 7.96 (m, 2H, $Ar_BC\mathit{H}$), 7.79 – 7.70 (m, 3H, C*H*=CH and $Ar_AC\mathit{H}$), 7.66 – 7.59 (m, 2H, $Ar_BC\mathit{H}$), 7.29 – 7.19 (m, 1H, $Ar_AC\mathit{H}$), 6.50 (d, J = 16.0 Hz, 1H, CH=C*H*CO), 3.81 (s, 3H, $C\mathit{H}_3$); ^{13}C NMR (101 MHz, $CDCl_3$) δ 167.5 (*C*O), 156.5 (Ar_A*C*), 149.9 (Ar_A*C*H), 144.4 (*C*H=CH), 141.2 (Ar_B*C*), 137.0 (Ar_A*C*H), 135.0 (Ar_B*C*CH), 128.6 (Ar_B*C*H), 127.4 (Ar_B*C*H), 122.7 (Ar_A*C*H), 120.7 (Ar_A*C*H), 118.3 (CH=*C*HCO), 51.9 ($\mathit{C}H_3$). HRMS (ESI): Calculated for $C_{15}H_{13}NO_2$ $(M-H)^+$, 240.1031; found 239.0957.

Methyl 3-(3,5-bis(trifluoromethyl)phenyl)acrylate 4y

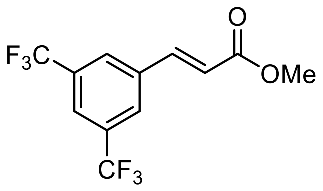

Following **GP7**, *(3,5-bis(trifluoromethyl)benzyl)(phenyl)sulfide* **1y** (10 mg, 0.30 mmol), 1,4-dinitrobenzene **A1** (101 mg, 0.60 mmol), and methyl (triphenylphosphoranylidene)acetate (201 mg, 0.60 mmol) afforded **4y** (50 mg, 56%, *E/Z* > 20:1) as a yellow liquid, after purification by column chromatography on silica gel (hexane/EtOAc 9:1 to 75:25) and washing with hexane to remove excess of 1,4-dinitrobenzene. ^{1}H NMR (500 MHz, $CDCl_3$) δ 7.93 (d, *J* = 1.6 Hz, 2H, ArC*H*), 7.88 (s, 1H, ArC*H*), 7.73 (d, *J* = 16.1 Hz, 1H, C*H*=CH), 6.58 (d, *J* = 16.0 Hz, 1H, CH=C*H*CO), 3.84 (s, 3H, CH_3); ^{13}C NMR (126 MHz, $CDCl_3$) δ 166.4 (*C*O), 141.4 (*C*H=CH), 136.6 (Ar*C*CH), 132.6 (q, *J* = 33.7 Hz, Ar*C*), 123.7 (q, *J* = 286.8 Hz, *C*F_3), 123.6 (m, 3 x Ar*CH*), 122.0 (CH=*C*HCO), 52.2 (*C*H_3). Data in accordance with the literature.[24]

Methyl 3-(4-fluorophenyl)acrylate 4z

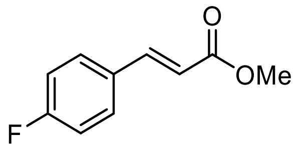

Following **GP7**, *(4-fluorobenzyl)(octyl)sulfide* **1ce** (76 mg, 0.30 mmol), 1,4-dinitrobenzene **A1** (150 mg, 0.60 mmol), and methyl (triphenylphosphoranylidene)acetate (201 mg, 0.60 mmol) afforded **4z** (34 mg, 63%, *E/Z* > 20:1) as a brown liquid, after purification by column chromatography on silica gel (toluene/hexane 2:8 to 100:0). ^{1}H NMR (400 MHz, $CDCl_3$) δ 7.65 (d, *J* = 16.0 Hz, 1H, C*H*=CH), 7.58 – 7.44 (m, 2H, ArC*H*), 7.12 – 7.02 (m, 2H, ArC*H*), 6.36 (dd, *J* = 16.0, 0.6 Hz, 1H, CH=C*H*CO), 3.80 (s, 3H, CH_3); ^{13}C NMR (101 MHz, $CDCl_3$) δ 167.4 (*C*O), 164.0 (d, *J* = 251.3 Hz, Ar*C*F), 143.7

(CH=CH), 130.8 (d, J = 3.4 Hz, ArC), 130.1 (d, J = 8.4 Hz, ArCH), 117.7 (d, J = 2.6 Hz, CH=CHCO), 116.2 (d, J = 21.9 Hz, ArCH), 51.9 (CH_3). Data in accordance with the literature.[25]

6 Substrate Scope of Alcohol Formation

Diphenylmethanol 5aa

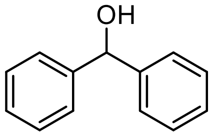

Following **GP8**, *benzylhydryl(phenyl)sulfide* **1aa** (84 mg, 0.30 mmol), 1,4-dinitrobenzene **A1** (150 mg, 0.60 mmol), and $NaBH_4$ (23 mg, 0.60 mmol) afforded **5aa** (44 mg, 80%) as a white solid, after purification by column chromatography on silica gel (hexane/EtOAc 100:0 to 95:5). ^{1}H NMR (400 MHz, $CDCl_3$) δ 7.41 – 7.32 (m, 8H, ArC*H*), 7.30 – 7.25 (m, 2H, ArC*H*), 5.86 (d, J = 3.5 Hz, 1H, C*H*), 2.25 (d, J = 3.5 Hz, 1H, O*H*); ^{13}C NMR (101 MHz, $CDCl_3$) δ 143.9 (Ar*C*), 128.6 (Ar*C*H), 127.7 (Ar*C*H), 126.9 (Ar*C*H), 76.4 (*C*H). Data in accordance with the literature.[26]

Phenyl(4-(trifluoromethyl)phenyl)methanol 5ab

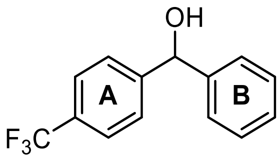

Following **GP8**, *phenyl(phenyl(4-(trifluoromethyl)phenyl)methyl)sulfide* **1ab** (84 mg, 0.30 mmol), 1,4-dinitrobenzene **A1** (150 mg, 0.60 mmol), and $NaBH_4$ (23 mg, 0.60 mmol) afforded **5ab** (65 mg, 86%) as an orange liquid, after purification by column chromatography on silica gel (hexane/EtOAc 100:0 to 1:1). ^{1}H NMR (400 MHz, $CDCl_3$) δ 7.60 (d, J = 8.1 Hz, 2H, Ar_AC*H*), 7.51 (d, J = 8.1 Hz, 2H, Ar_AC*H*), 7.38 – 7.28 (m, 5H, Ar_BC*H*), 5.88 (s, 1H, C*H*), 2.38 (s, 1H, O*H*); ^{13}C NMR (101 MHz, $CDCl_3$) δ 147.6 (Ar_A*C*), 143.3 (Ar_B*C*), 129.8 (q, J = 32.3 Hz, *C*CF_3), 128.9 (Ar_B*C*H), 128.2 (Ar_B*C*H), 126.8 (Ar_A*C*H),

126.8 (Ar$_B$*C*H), 125.5 (q, *J* = 3.9 Hz, Ar$_A$*C*H), 124.3 (q, *J* = 270 Hz, *C*F$_3$), 75.9 (*C*H); ^{19}F NMR (376 MHz, CDCl$_3$) δ -62.46. Data in accordance with the literature.[27]

isoPropyl-2-(4-((4-chlorophenyl)(hydroxy)methyl)phenoxy)-2-methylpropanoate 5ac

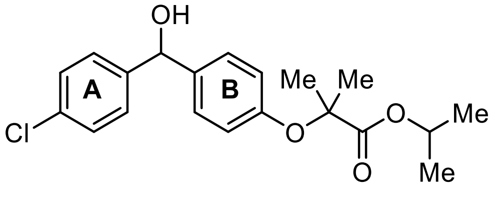

Following **GP8**, *isopropyl-2-(4-((4-chlorophenyl)(phenylthiol)methyl)phenoxy)-2-methylpropanoate* **1ac** (138 mg, 0.30 mmol), 1,4-dinitrobenzene **A1** (150 mg, 0.60 mmol), and NaBH$_4$ (23 mg, 0.60 mmol) afforded **5ac** (55 mg, 50%) as an orange liquid, after purification by column chromatography on silica gel (hexane/EtOAc 100:0 to 1:1). ^{1}H NMR (400 MHz, CDCl$_3$) δ 7.31 - 7.16 (m, 4H, Ar$_B$C*H*), 7.14 – 7.09 (m, 2H, Ar$_A$C*H*), 6.76 – 6.69 (m, 2H, Ar$_A$C*H*), 5.68 (s, 1H, C*H*), 4.99 (p, *J* = 6.3 Hz, 1H, C*H*(CH$_3$)$_2$), 2.09 (s, 1H, O*H*), 1.50 (s, 6H, 2 x C*H*$_3$), 1.13 (d, *J* = 6.2 Hz, 6H, CH(C*H*$_3$)$_2$); ^{13}C NMR (101 MHz, CDCl$_3$) δ 173.8 (*C*O), 155.3 (Ar$_A$*C*Cl), 142.5 (Ar$_B$*C*CH), 137.0 (Ar$_A$*C*CH), 133.3 (Ar$_B$*C*O), 128.6 (Ar$_B$*C*H), 127.9 (Ar$_B$*C*H), 127.6 (Ar$_A$*C*H), 119.0 (Ar$_A$*C*H), 79.3 (*C*(CH$_3$)$_2$), 75.3 (*C*H), 69.1 (*C*H(CH$_3$)$_2$), 25.5 (2 x *C*H$_3$), 21.9 (CH(*C*H$_3$)$_2$). Data in accordance with the literature.[28]

Methyl(4-(hydroxy(phenyl)methyl)benzoyl)-*D*-prolinate 5ad

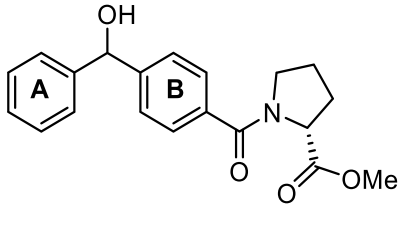

Following **GP8**, *methyl-(4-(phenyl(phenylthio)methyl)benzoyl)-D-prolinate* **1ad** (129 mg, 0.30 mmol), 1,4-dinitrobenzene **A1** (150 mg, 0.60 mmol), and NaBH$_4$ (23 mg, 0.60 mmol) afforded **5ad** (80 mg, 67%) as an orange liquid, after purification by column chromatography on silica gel (hexane/EtOAc 100:0 to 0:100). ^{1}H NMR (400 MHz, CDCl$_3$) δ 7.51 (d, *J* = 8.0 Hz, 2H, Ar$_B$C*H*), 7.40 (d, *J* = 7.9 Hz,

2H, Ar_BCH), 7.36 – 7.28 (m, 5H, Ar_ACH), 5.83 (s, 1H, C*H*), 4.63 (dd, *J* = 8.3, 5.1 Hz, 1H, C*H*$COCH_3$), 3.76 (s, 3H, OCH_3), 3.63 (m, 1H, CH_2), 3.55 – 3.43 (m, 1H, CH_2), 2.47 (brs, 1H, O*H*), 2.37 – 2.24 (m, 1H, CH_2), 2.00 (m, 2H, CH_2), 1.87 (m, 1H, CH_2); ^{13}C NMR (126 MHz, $CDCl_3$) δ 172.9 (*C*$OOCH_3$), 169.6 (*C*ON), 146.2 (Ar_BCCH), 143.6 (Ar_AC), 135.1 (Ar_BCCO), 128.7 (Ar_ACH), 127.9 (Ar_ACH), 127.7 (Ar_BCH), 126.8 (Ar_ACH), 126.4 (Ar_BCH), 76.0 (*C*H), 59.3 (*C*H$COCH_3$), 52.4 (OCH_3), 50.1 (CH_2), 29.5 (CH_2), 25.5 (CH_2). HRMS (APCI): Calculated for $C_{20}H_{21}O_4N$ $(M-H)^+$, 340.1543; found 340.1539.

(2S)-*N*-(4-(Hydroxy(phenyl)methyl)phenyl)-2-(6-methoxynaphthalen-2-yl)propanamide 5ae

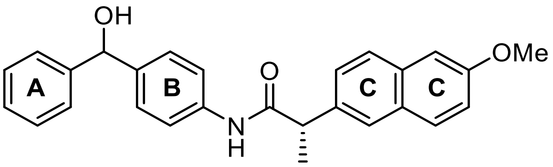

Following **GP8**, *(2S)-2-(6-methoxynaphthalen-2-yl)-N-(4-(phenyl(phenylthiol)methyl)phenyl)propenamide* **1ae** (150 mg, 0.30 mmol), 1,4-dinitrobenzene **A1** (150 mg, 0.60 mmol), and $NaBH_4$ (23 mg, 0.6 mmol) afforded **5ae** (56 mg, 46%) as a yellow liquid, after purification by column chromatography on silica gel (hexane/EtOAc 100:0 to 7:3). ^1H NMR (700 MHz, $CDCl_3$) δ 7.77 – 7.70 (m, 3H, ArC*H*), 7.43 – 7.28 (m, 7H, ArC*H*), 7.25 – 7.11 (m, 5H, ArC*H*), 5.77 (s, 1H, C*H*), 3.92 (s, 3H, OCH_3), 3.84 (q, *J* = 7.1 Hz, 1H, C*H*CH_3), 1.66 (d, *J* = 7.0 Hz, 3H, CH_3), 1.58 (s, 1H, O*H*); ^{13}C NMR (176 MHz, $CDCl_3$) δ 172.6 (*C*ONH), 158.1 (Ar_CCOCH_3), 143.9 (Ar_AC), 139.9 (Ar_BC), 137.3 (Ar_CC), 136.0 (d, *J* = 16.7 Hz, Ar_CCCHCH_3), 134.1 (Ar_CC), 129.4 (Ar*C*H), 129.2 (Ar*C*H), 128.6 (Ar_BCH), 128.5 (Ar_BCH), 128.1 (Ar*C*H), 127.7 (Ar*C*H), 127.3 (Ar*C*H), 126.6 (Ar*C*H), 126.5 (Ar_BCNH), 126.3 (Ar*C*H), 119.8 (Ar*C*H), 119.5 (Ar_BCH), 119.4 (Ar_BCH), 105.9 (Ar_CCH), 75.9 (Ar*C*H), 55.5 (*C*H), 48.2 (*C*HCH_3), 18.6 (CH_3). HRMS (APCI): Calculated for $C_{27}H_{25}O_3N$ $(M-H)^+$, 412.1907; found 412.1912.

2,2-Dimethyl-1-phenylpropan-1-ol 5af

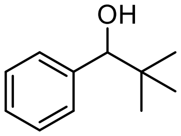

Following **GP8**, *(2,2-dimethyl-1-phenylpropyl)(phenyl)sulfide* **1af** (78 mg, 0.30 mmol), 1,4-dinitrobenzene **A1** (150 mg, 0.60 mmol), and $NaBH_4$ (34 mg, 0.90 mmol) afforded **5af** (27 mg, 55%) as a white solid, after purification by column chromatography on silica gel (hexane/EtOAc 100:0 to 9:1). ^{1}H NMR (400 MHz, $CDCl_3$) δ 7.31 – 7.19 (m, 5H, ArC*H*), 4.35 (s, 1H, C*H*), 1.71 (brs, 1H, O*H*), 0.88 (s, 9H, 3 x CH_3); ^{13}C NMR (101 MHz, $CDCl_3$) δ 142.3 (Ar*C*), 127.7 (Ar*C*H), 127.7 (Ar*C*H), 127.4 (Ar*C*H), 82.6 (*C*H), 35.8 (*C*(CH_3)$_3$), 26.1 (3 x *C*H_3). Data in accordance with the literature.[29]

***tert*-Butyl (3-hydroxycyclobutyl)carbamate 5ag**

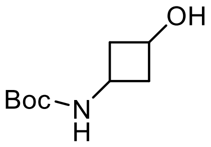

Following **GP8**, *tert-butyl (3-(phenylthio)cyclobutyl)carbamate* **1ag** (84 mg, 0.30 mmol), 1,4-dinitrobenzene **A1** (150 mg, 0.60 mmol), and $NaBH_4$ (34 mg, 0.90 mmol) afforded **5ag** (30 mg, 54%, syn:anti = 2.2:1) as a yellow liquid, after purification by column chromatography on deactivated silica gel (hexane/EtOAc 100:0 to 7:3). ^{1}H NMR (400 MHz, $CDCl_3$) major δ 4.68 (brs, 1H, N*H*), 4.01 (p, *J* = 7.1 Hz, 1H, C*H*NH), 3.64 (brs, 1H, C*H*OH), 2.75 (ddd, *J* = 12.7, 9.6, 6.8 Hz, 2H, CH_2), 1.79 (qd, *J* = 8.6, 3.1 Hz, 2H, CH_2), 1.43 (s, 9H, 3 x CH_3), minor δ 4.68 (brs, 1H, N*H*), 4.46 (ddd, *J* = 11.5, 6.9, 4.5 Hz, 1H, C*H*NH), 4.21 (brs, 1,H, C*H*OH), 2.36 – 2.02 (m, 4H, 2 x CH_2), 1.43 (s, 9H, 3 x CH_3); ^{13}C NMR (101 MHz, $CDCl_3$) major δ 155.2 (*C*O), 79.6 (*C*CH_3), 61.0 (*C*HNH), 41.9 (2 x *C*H_2), 37.4 (*C*HOH), 28.5 (3 x *C*H_3), minor δ 155.2 (*C*O), 79.6 (*C*CH_3), 65.0 (*C*HNH), 41.9 (*C*HOH), 40.3 (2 x *C*H_2), 28.5 (3 x *C*H_3). HRMS (APCI): Calculated for $C_9H_{18}NO_3$ (M-H)$^+$ 188.1281; found 188.1283.

Ethyl-3-hydroxycyclobutane-1-carboxylate 5ah

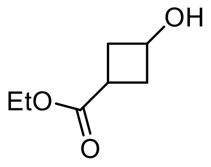

Following **GP8**, *ethyl-3-(phenylthio)cyclobutene-1-carboxylate* **1ah** (71 mg, 0.30 mmol), 1,4-dinitrobenzene **A1** (150 mg, 0.60 mmol), and $NaBH_4$ (34 mg, 0.90 mmol) afforded **5ah** (20 mg, 47%, syn:anti > 20:1) as a yellow liquid, after purification by column chromatography on deactivated silica gel (hexane/EtOAc 100:0 to 7:3). ^{1}H NMR (400 MHz, $CDCl_3$) δ 4.23 – 4.10 (m, 3H, OCH_2CH_3 and $CHCOCH_2CH_3$), 2.65 – 2.53 (m, 3H, CH_2 and CHOH), 2.24 – 2.11 (m, 2H, CH_2), 1.89 (m, 1H, OH), 1.26 (t, J = 7.1 Hz, 3H, CH_3); ^{13}C NMR (101 MHz, $CDCl_3$) δ 175.0 (CO), 63.7 (CHCO), 60.8 (OCH_2CH_3), 37.2 (2 x CH_2), 29.2 (CHOH), 14.4 (OCH_2CH_3). Data in accordance with the literature.[30]

7 NMR Spectra

7.1 NMR Spectra of Starting Materials

1b – ^{1}H-NMR (500 MHz, $CDCl_3$)

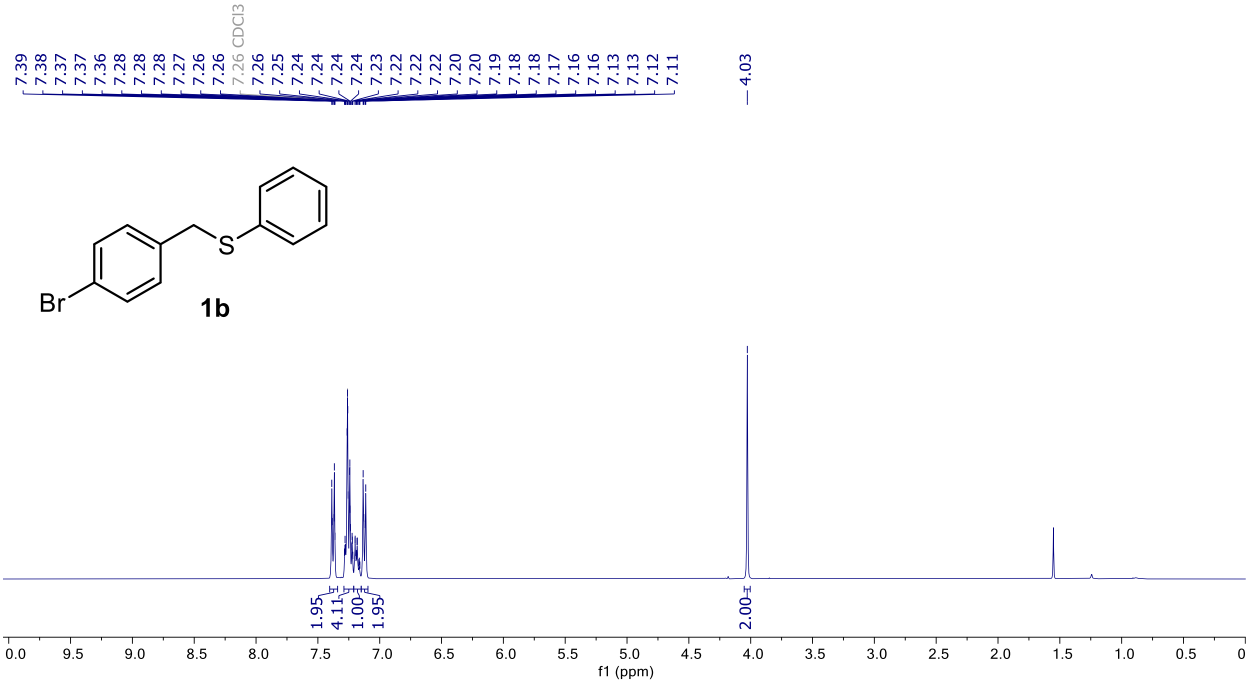

1b – ^{13}C-NMR (126 MHz, $CDCl_3$)

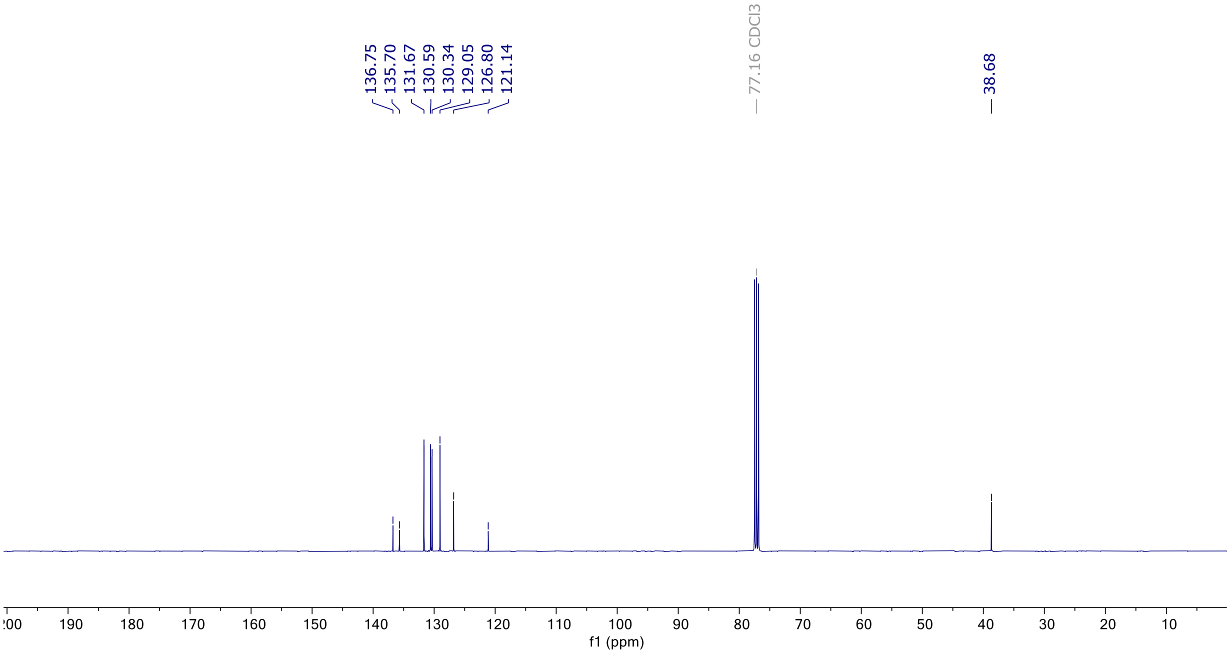

1c – ^{1}H-NMR (400 MHz, $CDCl_3$)

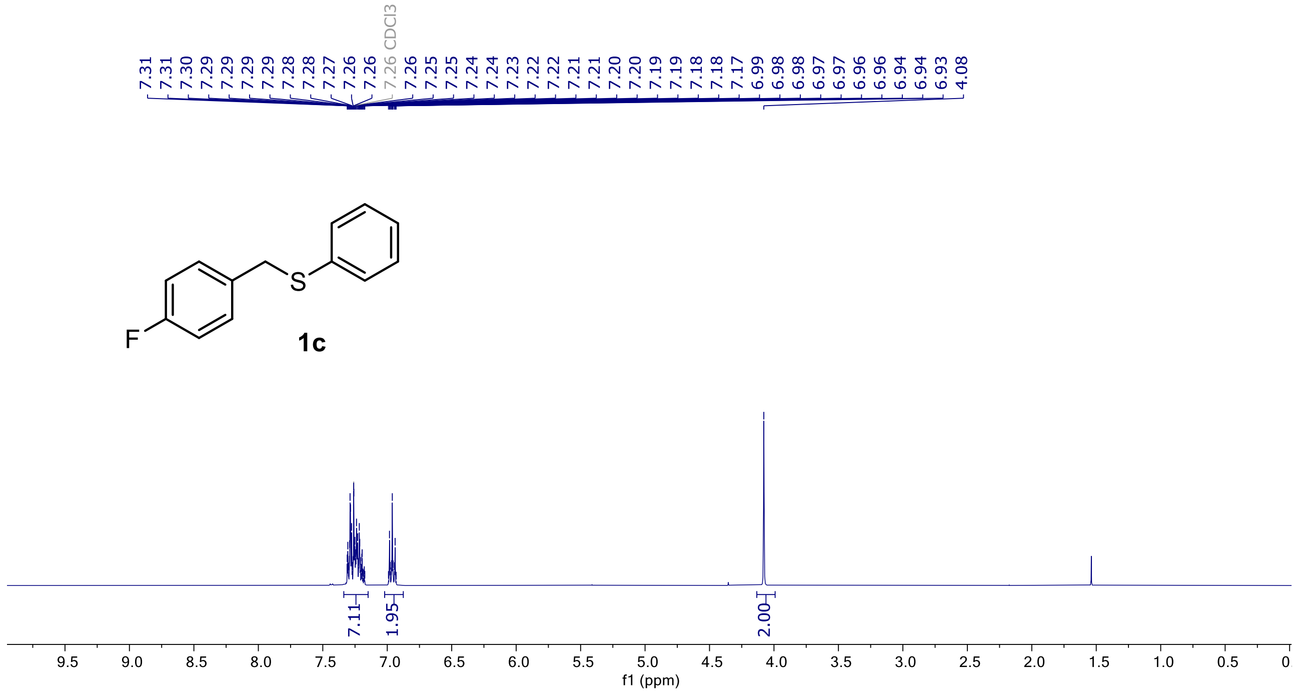

1c – ^{13}C-NMR (101 MHz, $CDCl_3$)

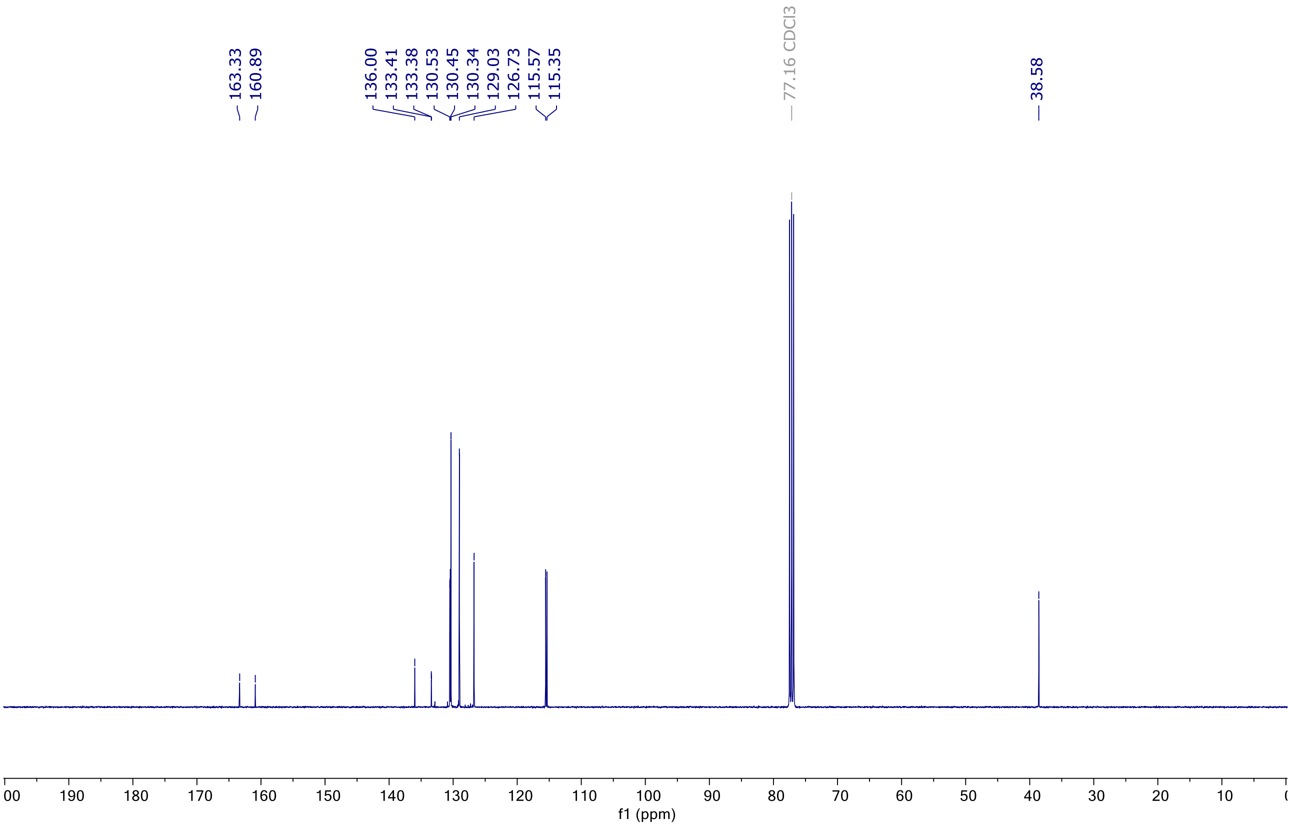

1d – ^{1}H-NMR (500 MHz, $CDCl_3$)

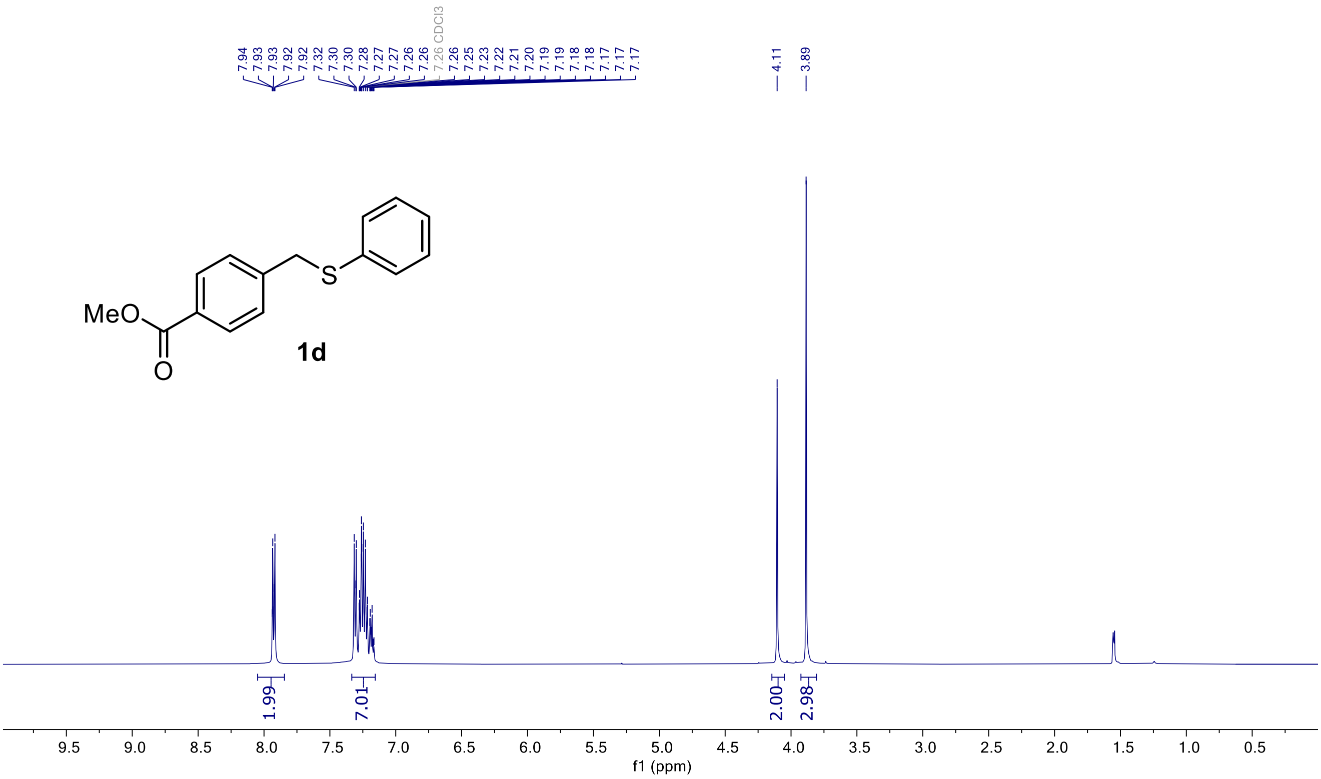

1d – ^{13}C-NMR (126 MHz, $CDCl_3$)

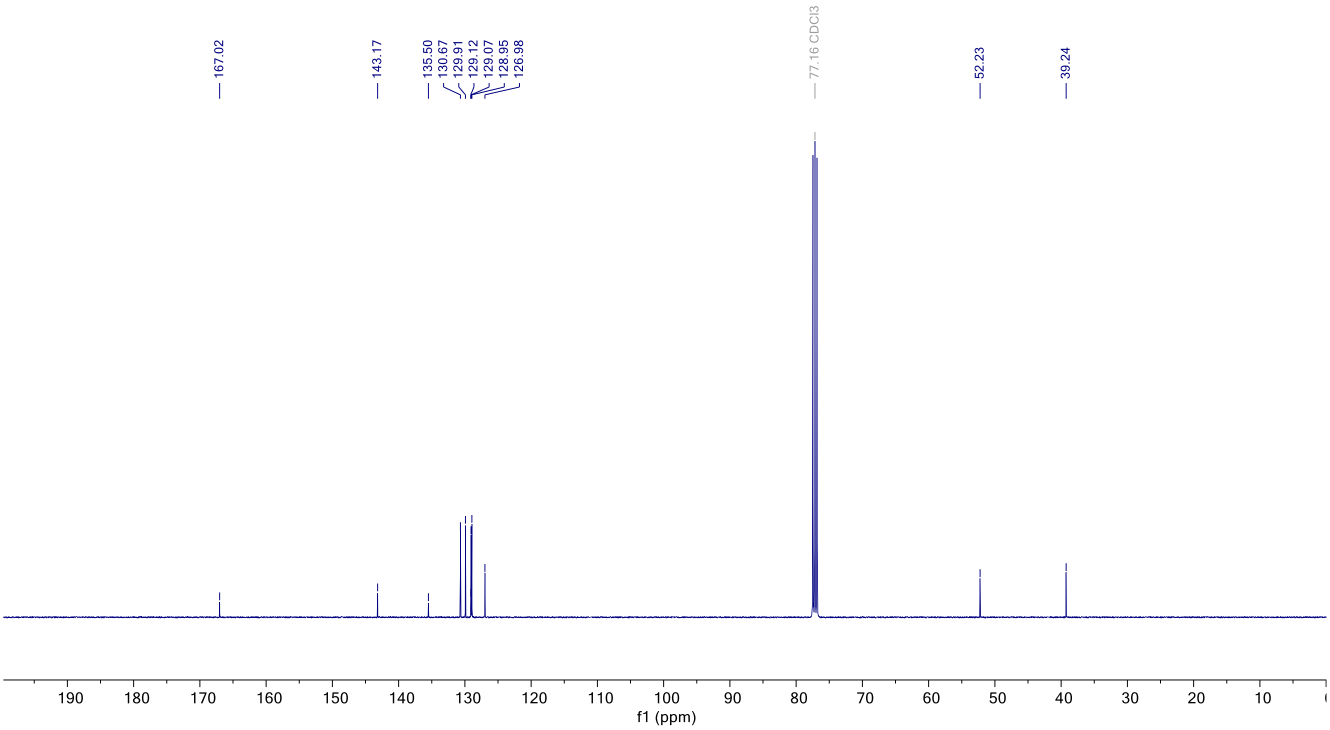

1e – ^{1}H-NMR (400 MHz, $CDCl_3$)

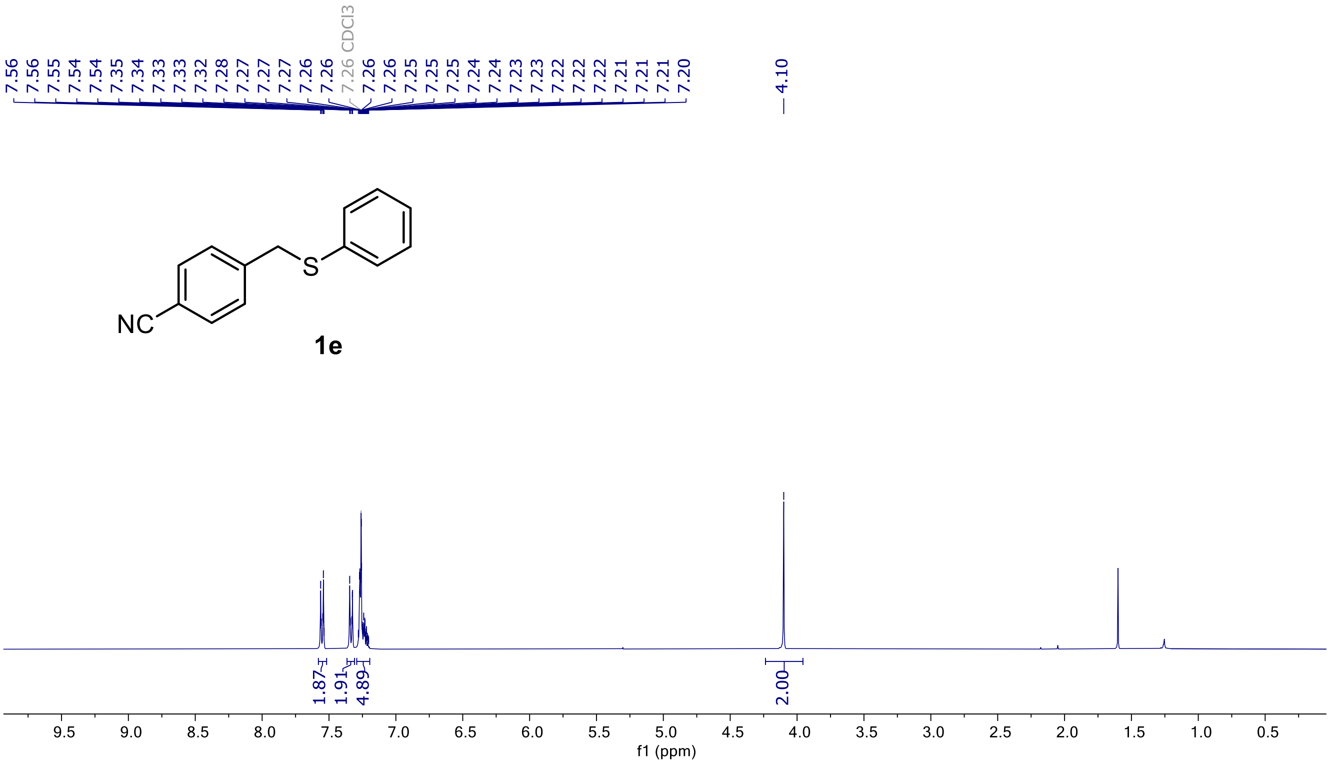

1e – ^{13}C-NMR (101 MHz, $CDCl_3$)

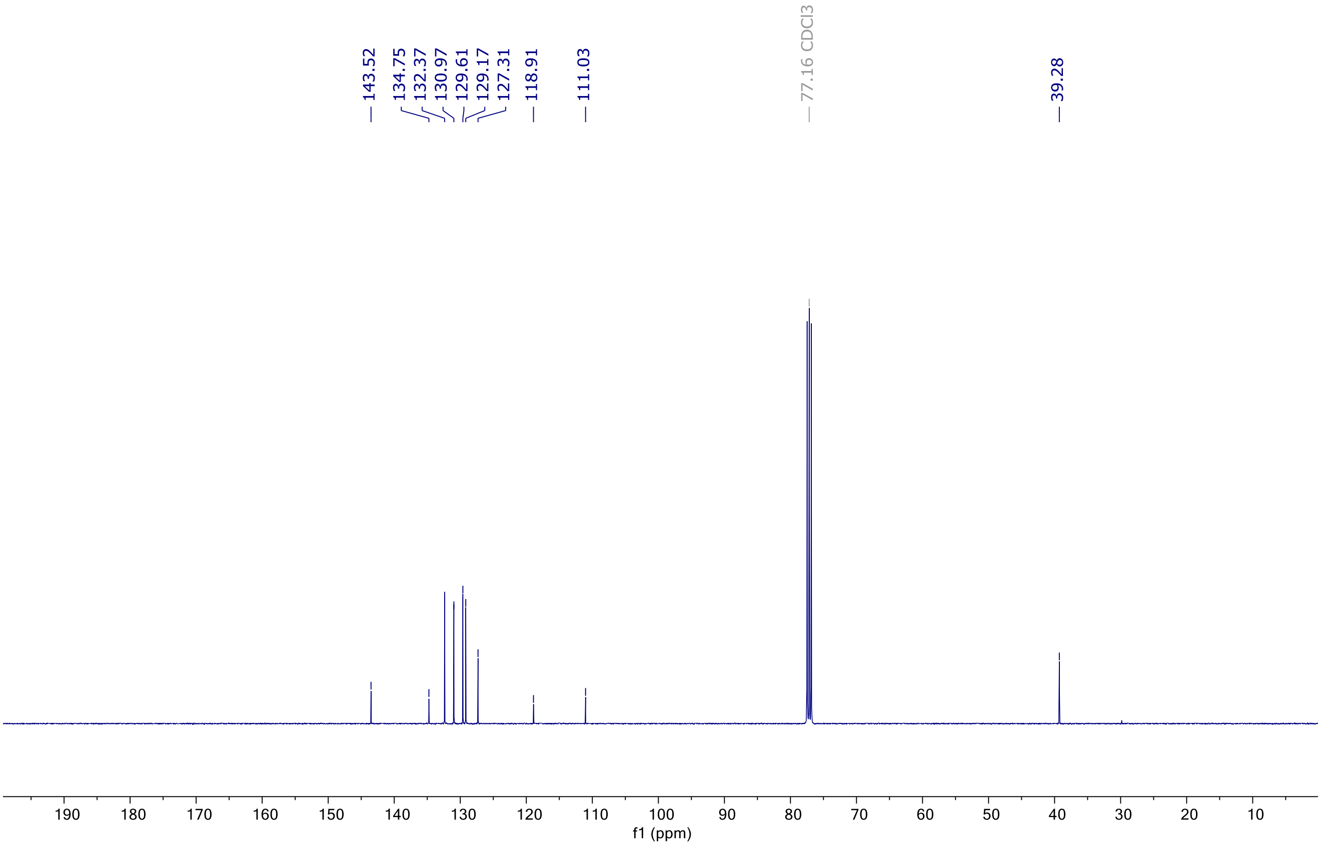

1f – ¹H-NMR (400 MHz, $CDCl_3$)

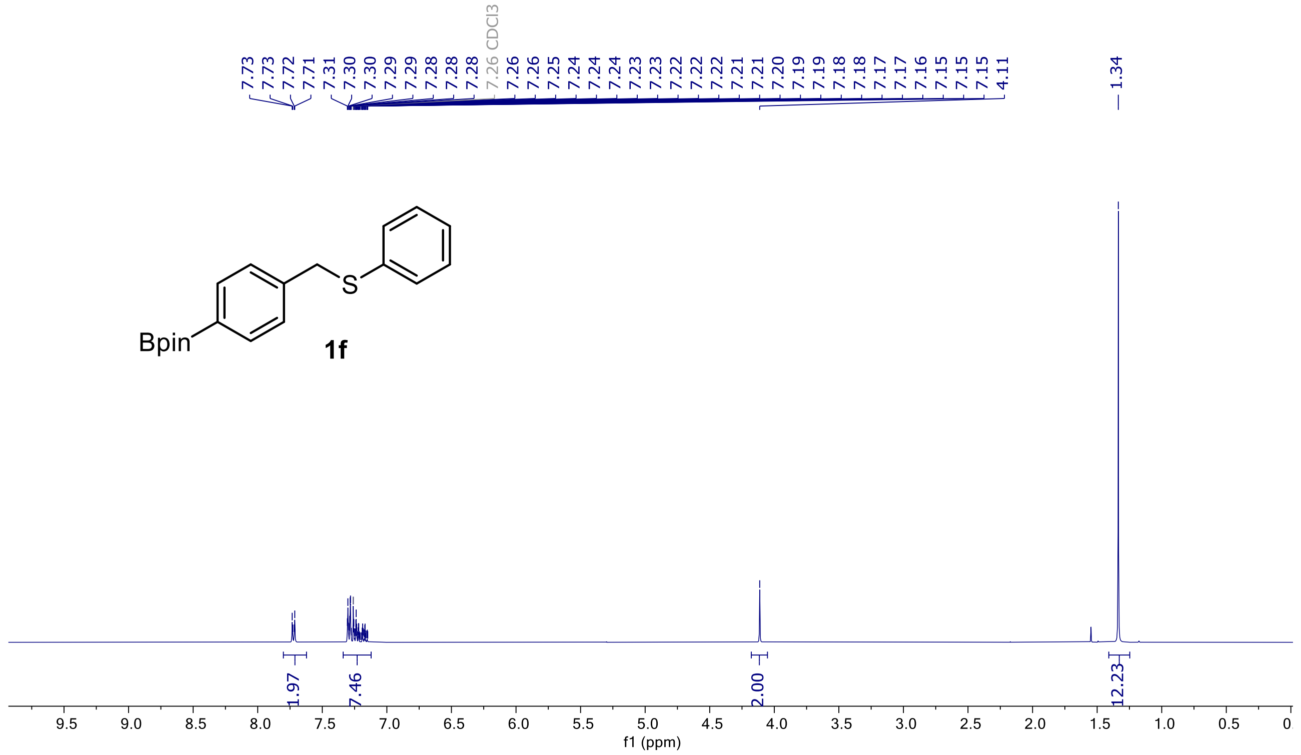

1f – ¹³C-NMR (101 MHz, $CDCl_3$)

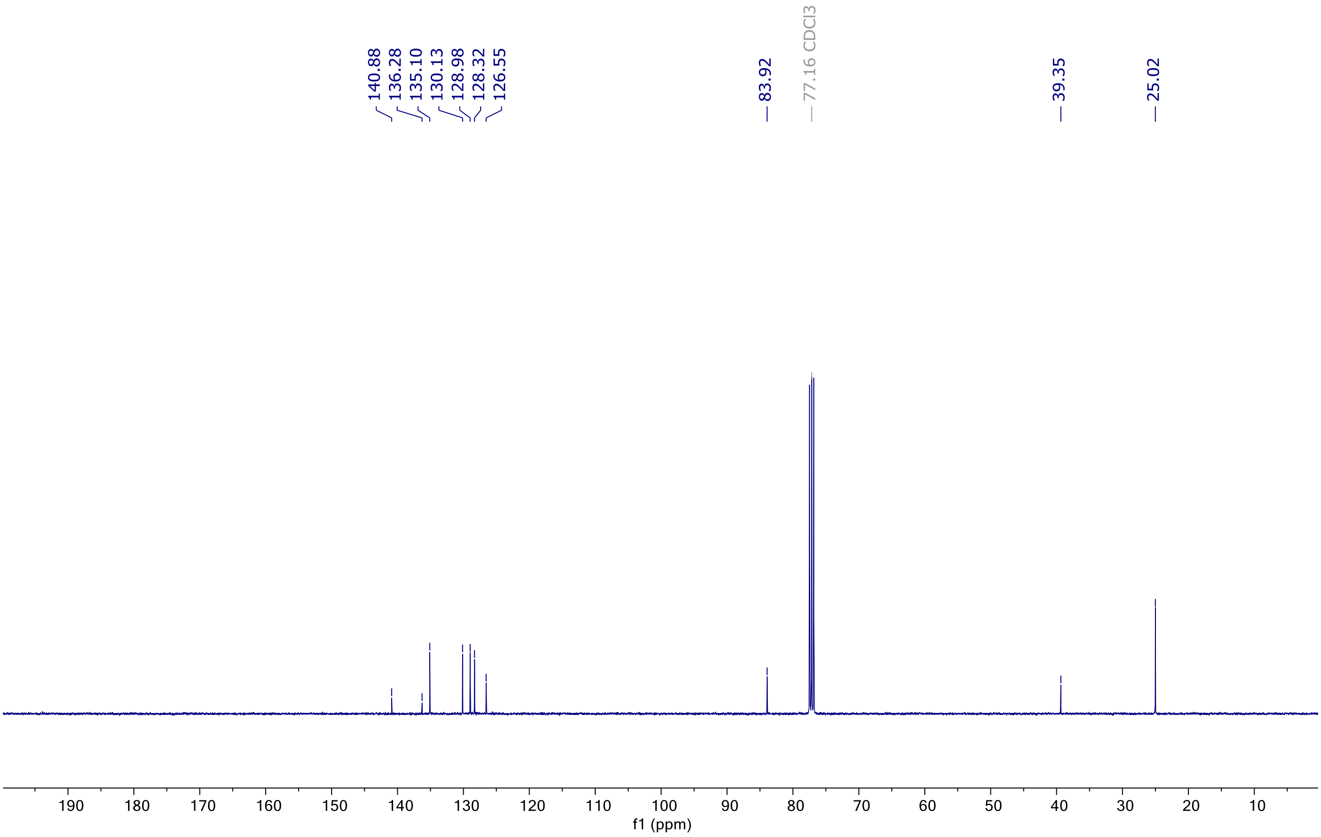

1g – ^{1}H-NMR (400 MHz, $CDCl_3$)

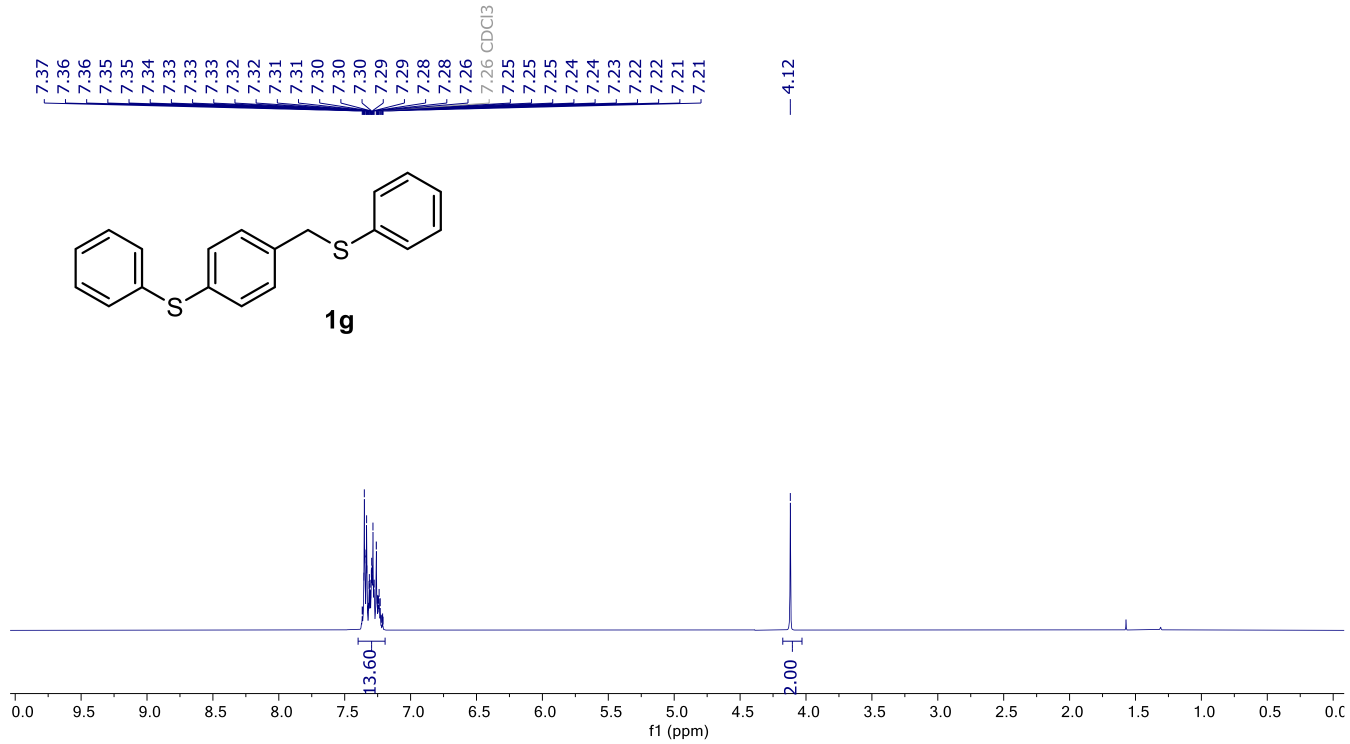

1g – ^{13}C-NMR (101 MHz, $CDCl_3$)

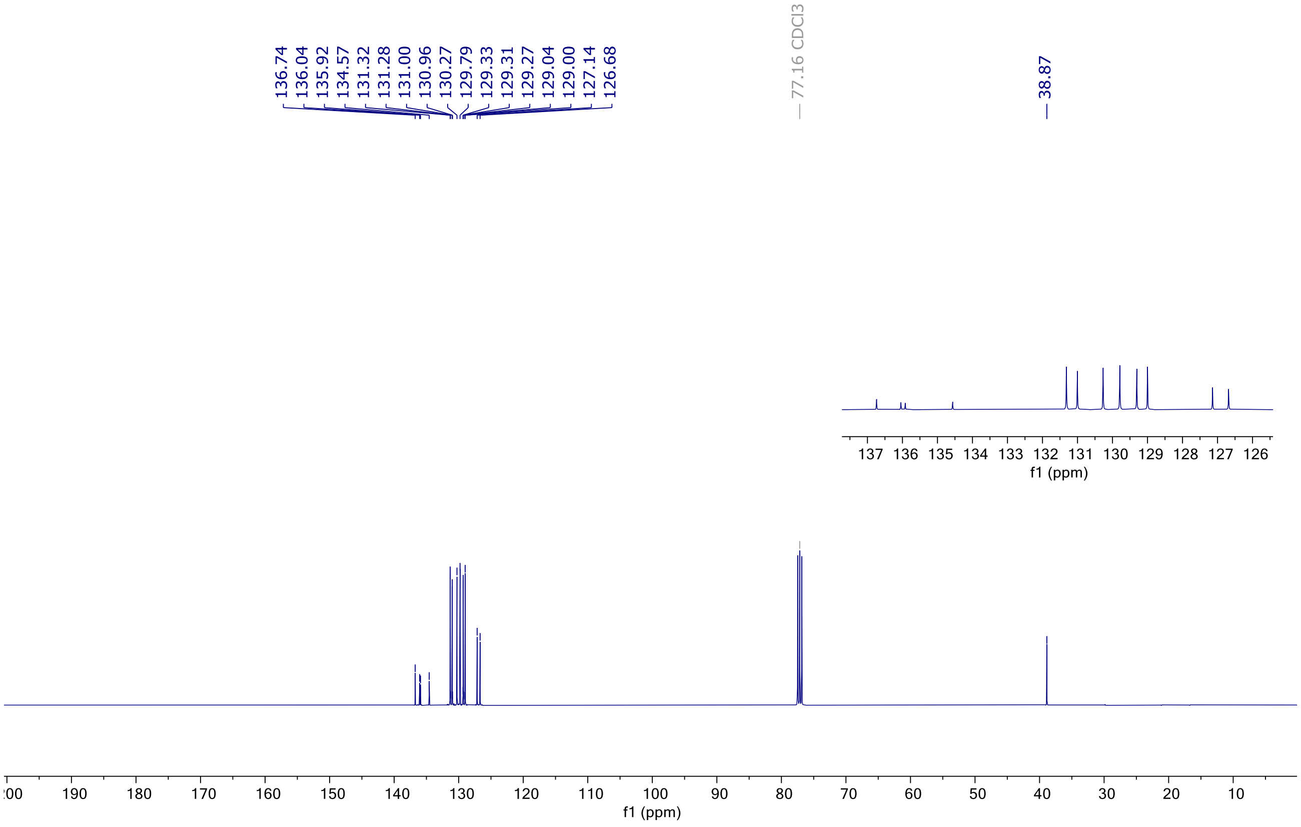

1h – ^{1}H-NMR (400 MHz, $CDCl_3$)

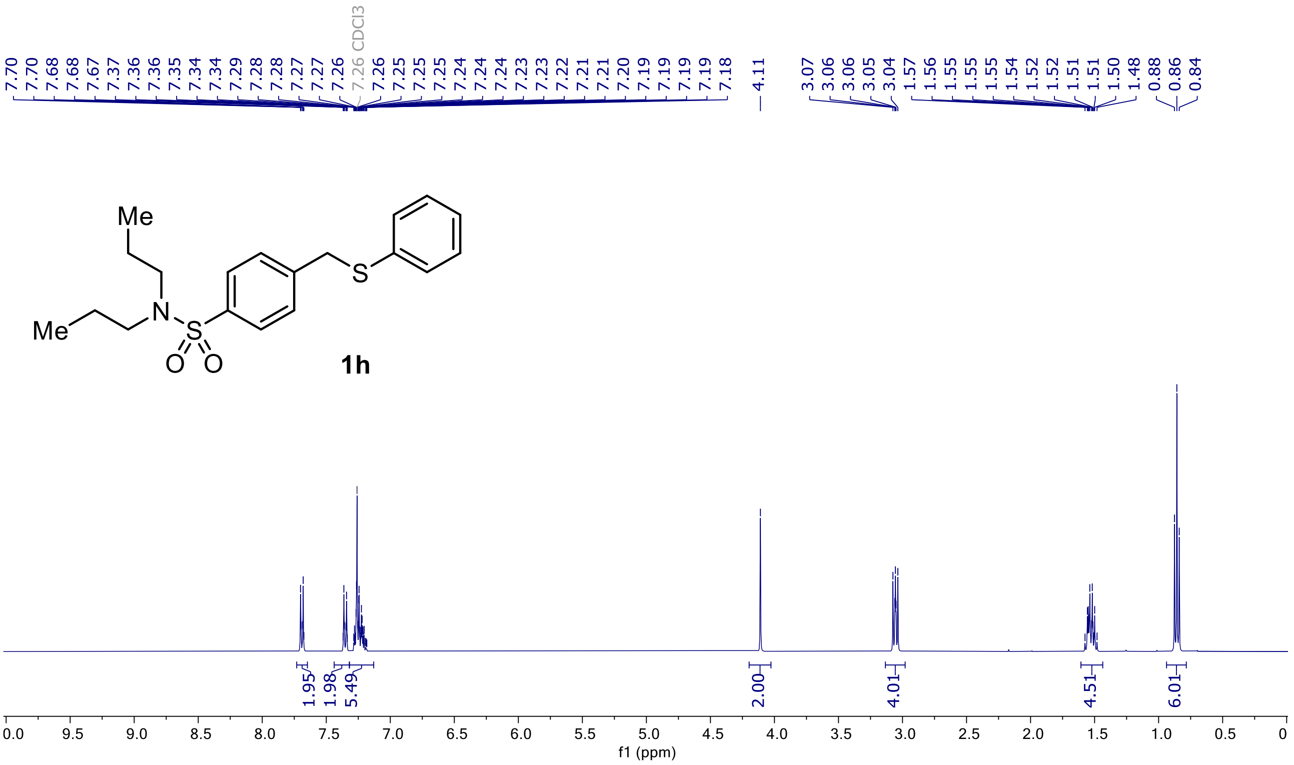

1h – ^{13}C-NMR (101 MHz, $CDCl_3$)

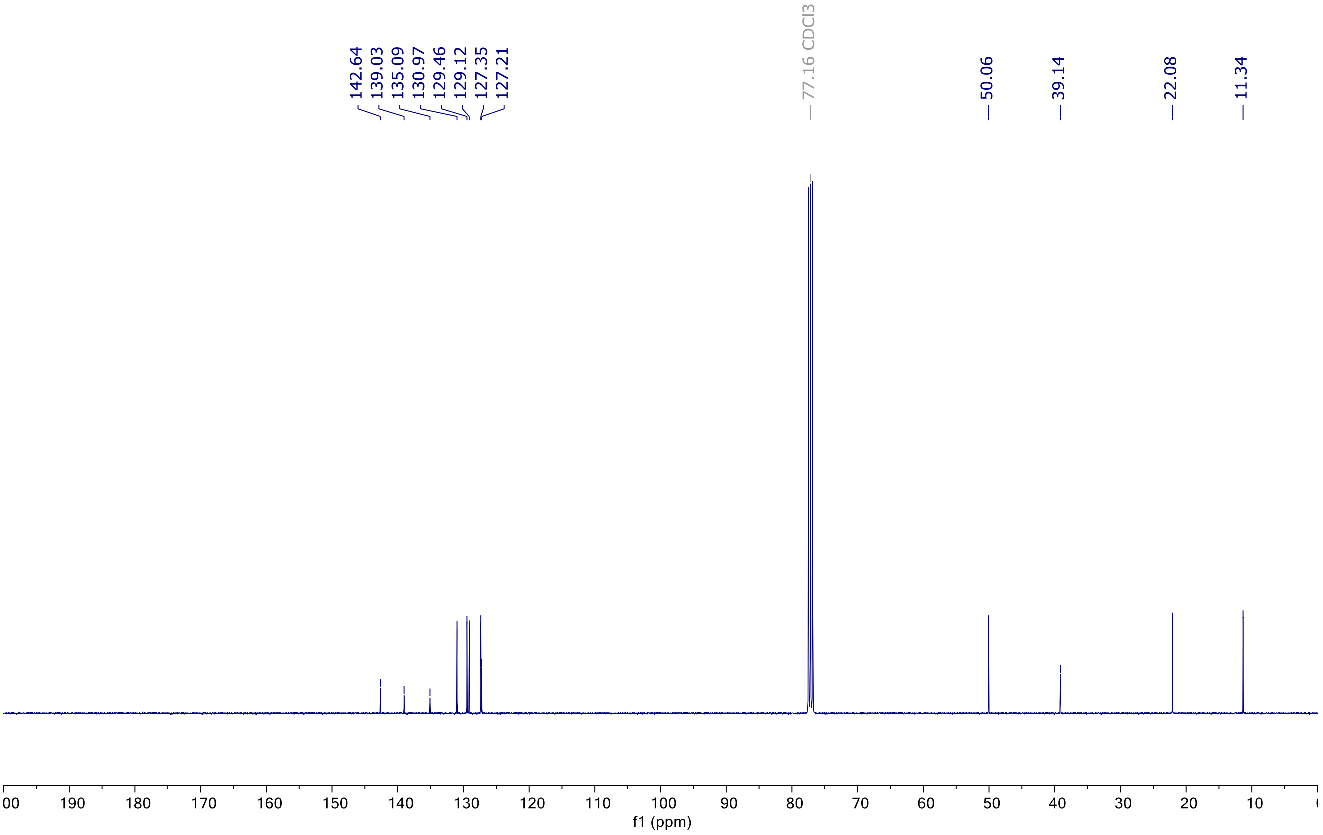

1i – ^{1}H-NMR (400 MHz, $CDCl_3$)

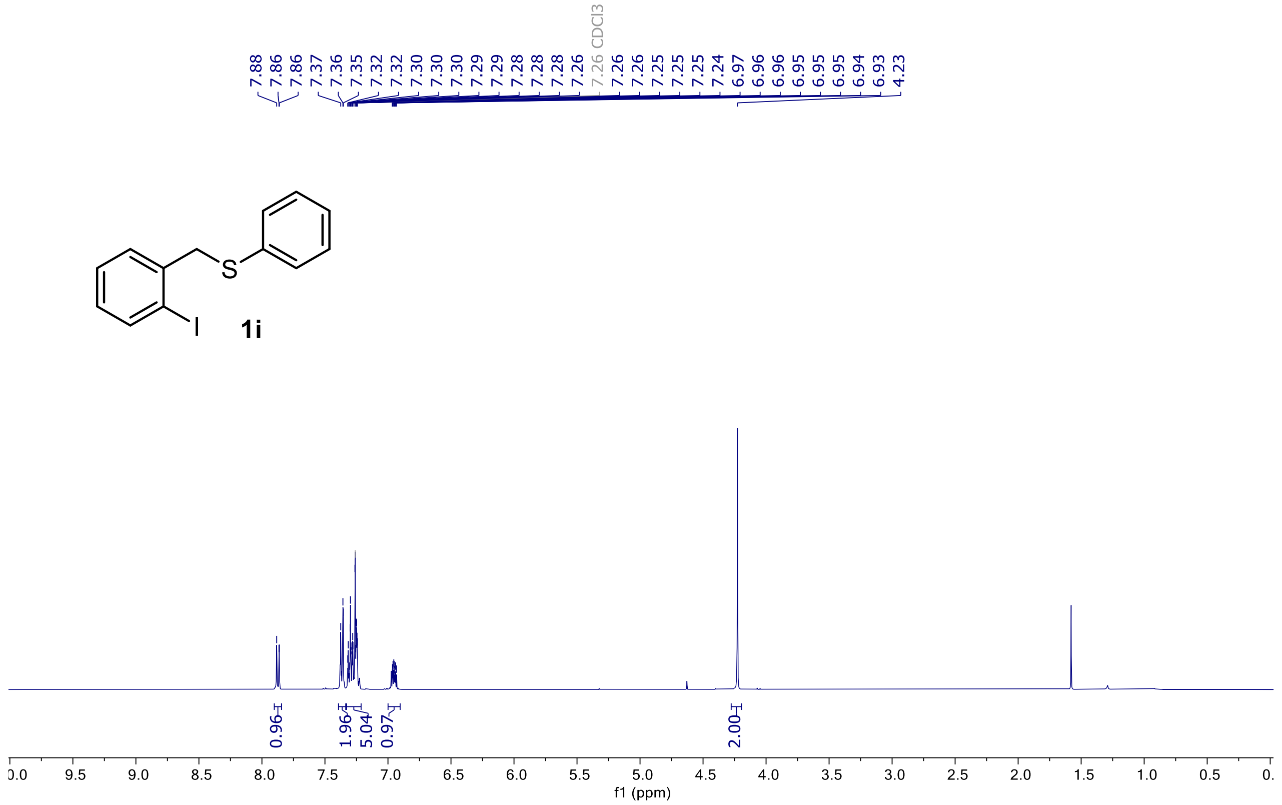

1i – ^{13}C-NMR (101 MHz, $CDCl_3$)

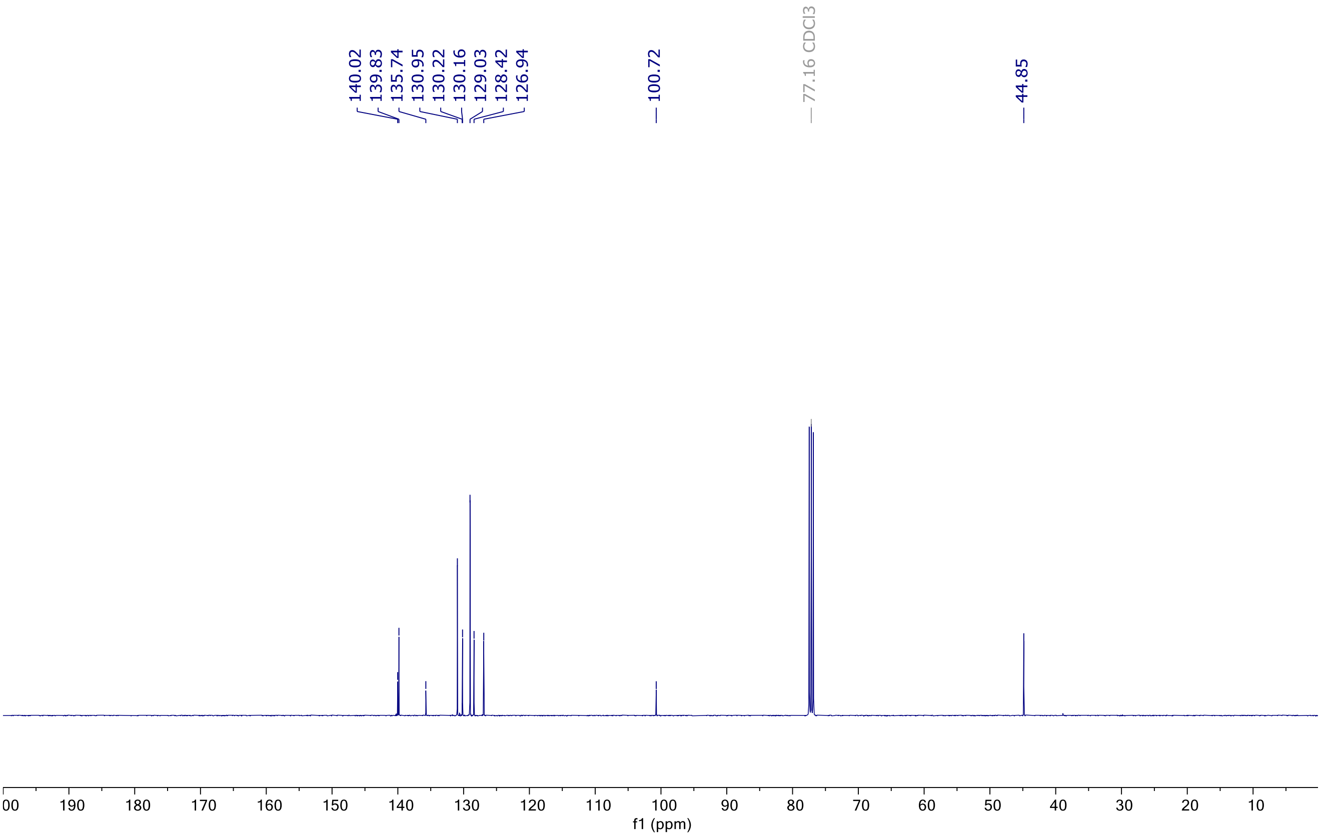

1j – ^{1}H-NMR (500 MHz, $CDCl_3$) - mixture of rotamers

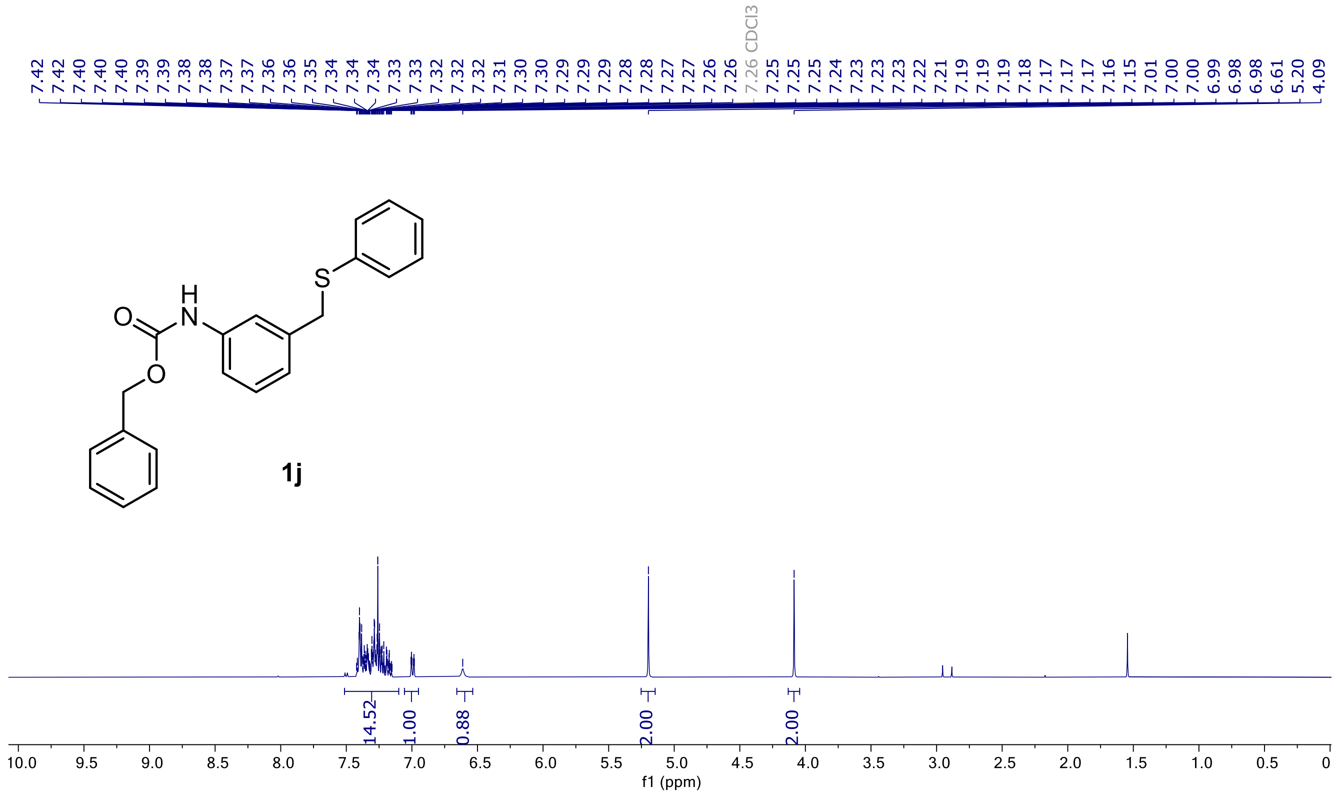

1j – ^{13}C-NMR (126 MHz, $CDCl_3$) - mixture of rotamers

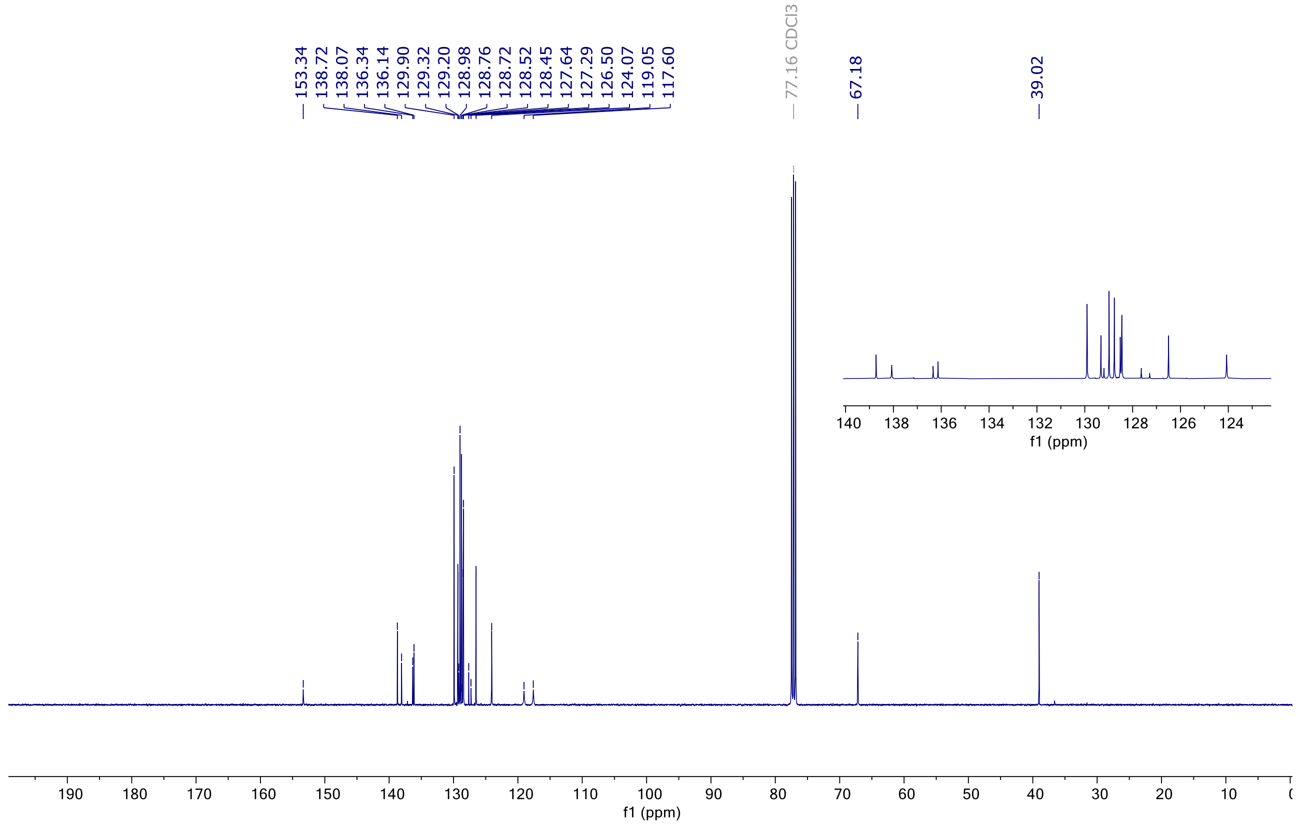

1k – ^{1}H-NMR (500 MHz, $CDCl_3$)

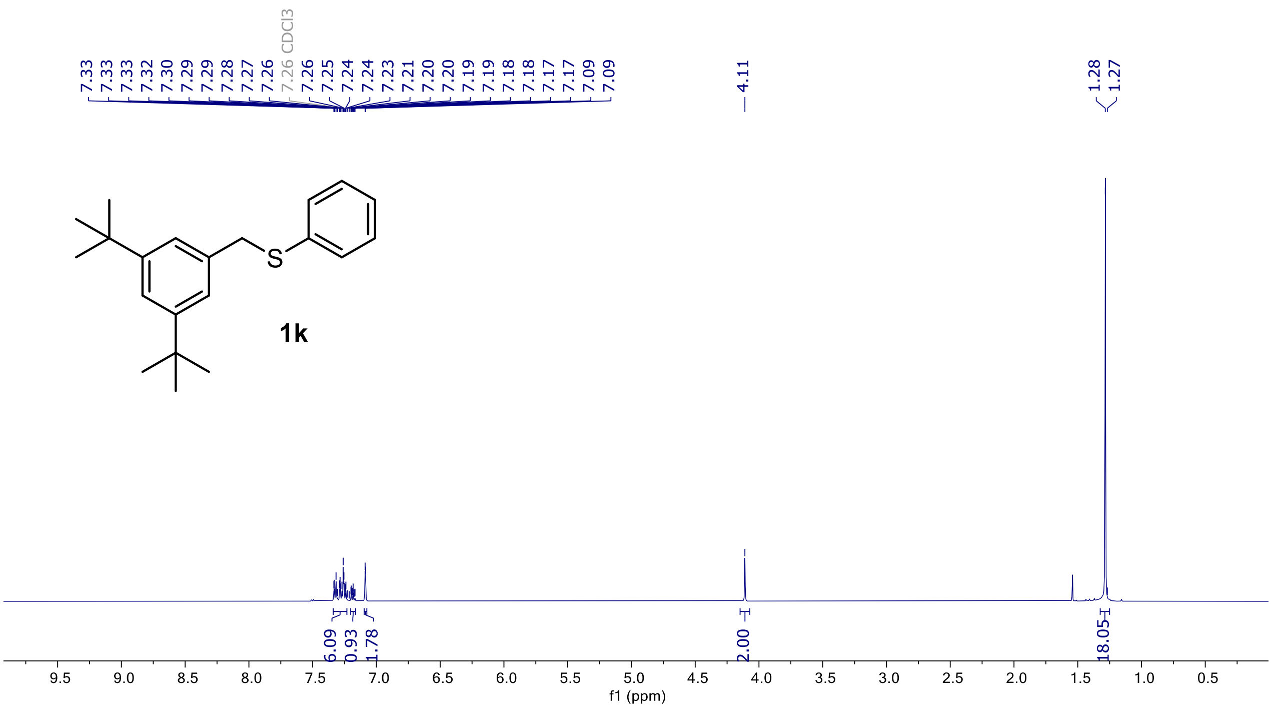

1k – ^{13}C-NMR (126 MHz, $CDCl_3$)

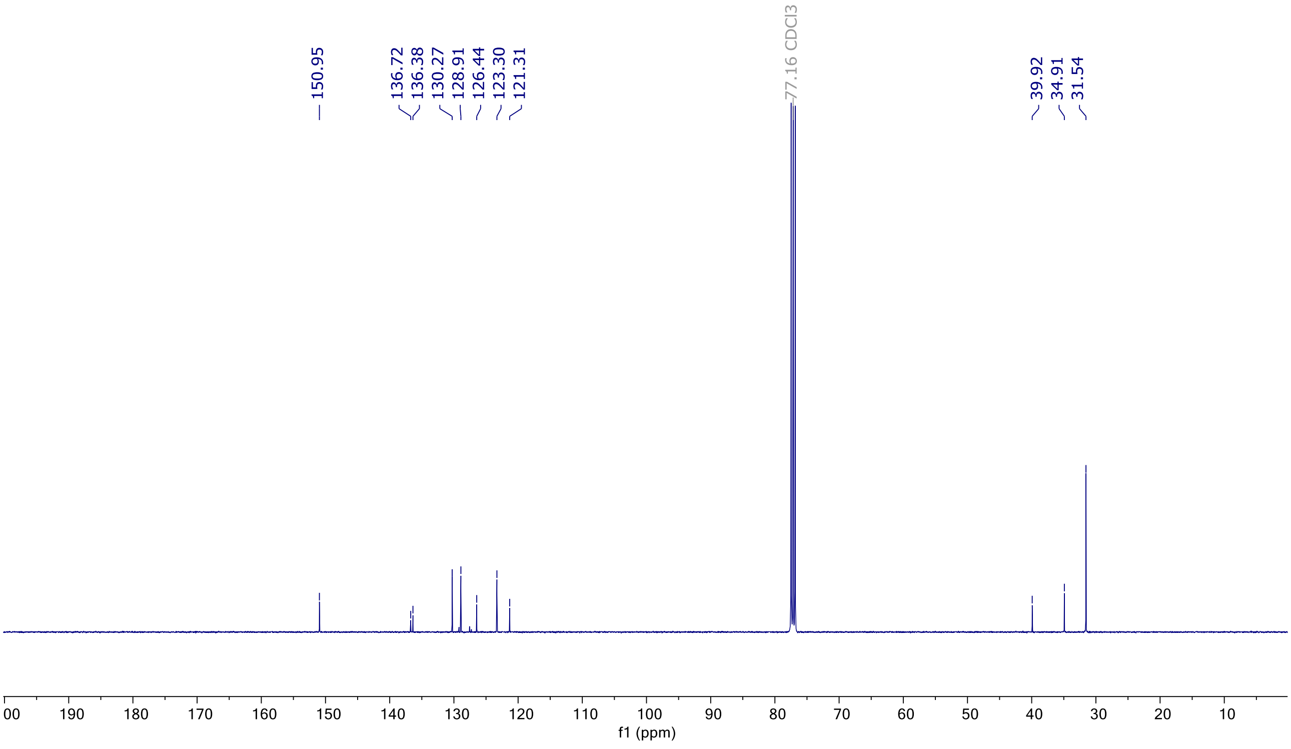

1l – ^{1}H-NMR (400 MHz, $CDCl_3$)

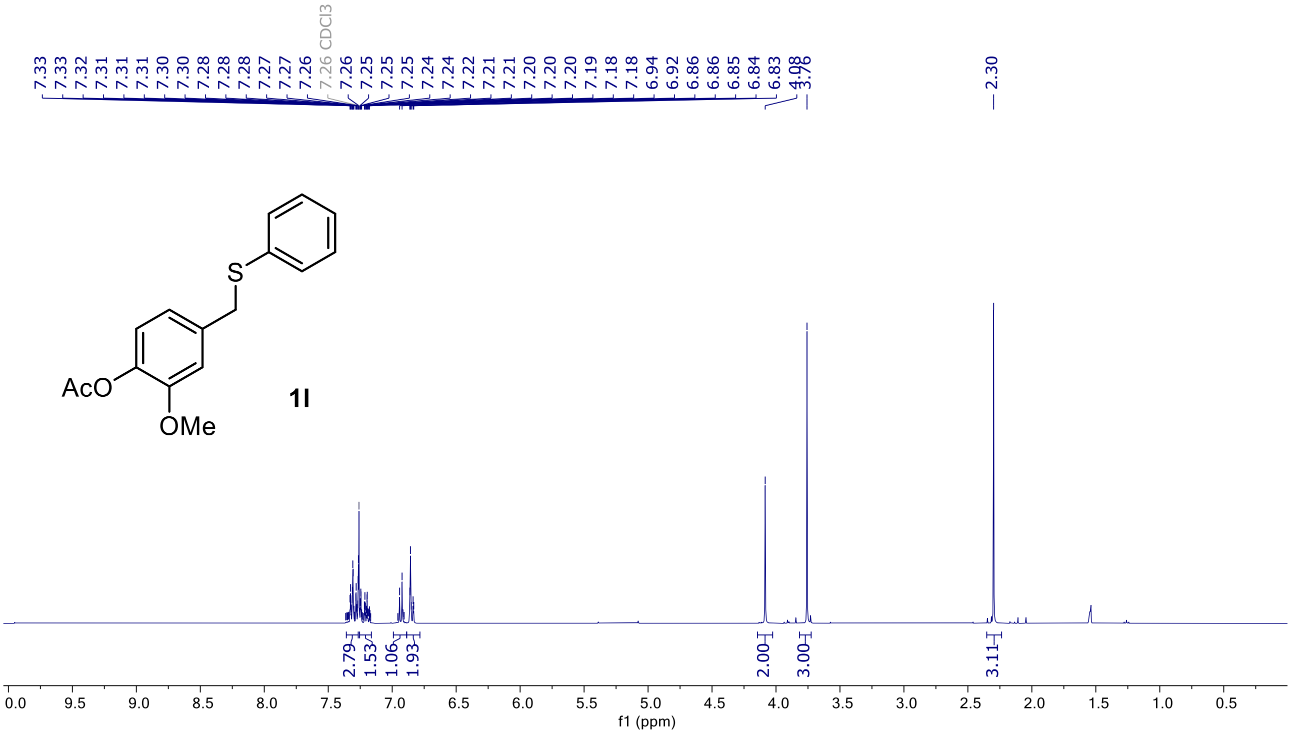

1l – ^{13}C-NMR (101 MHz, $CDCl_3$)

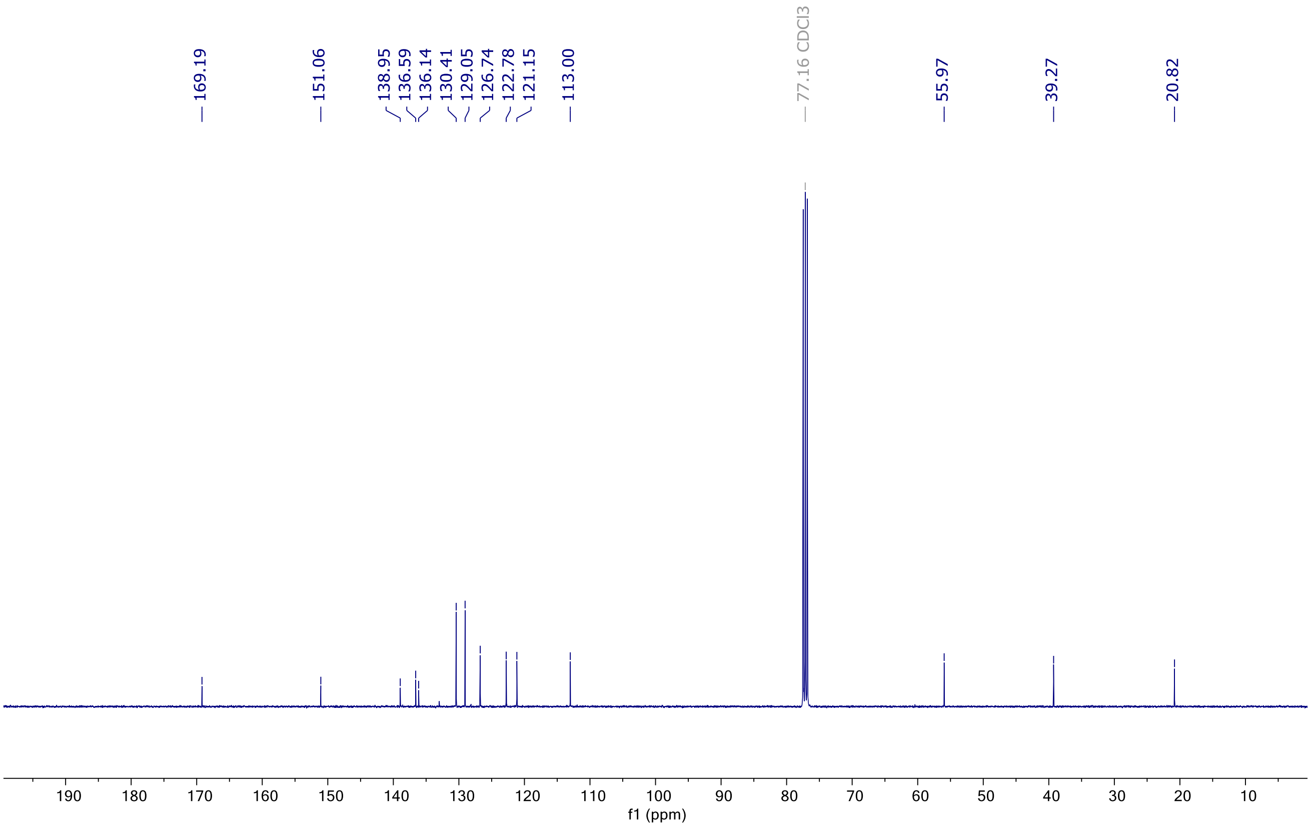

1m – ^{1}H-NMR (500 MHz, $CDCl_3$)

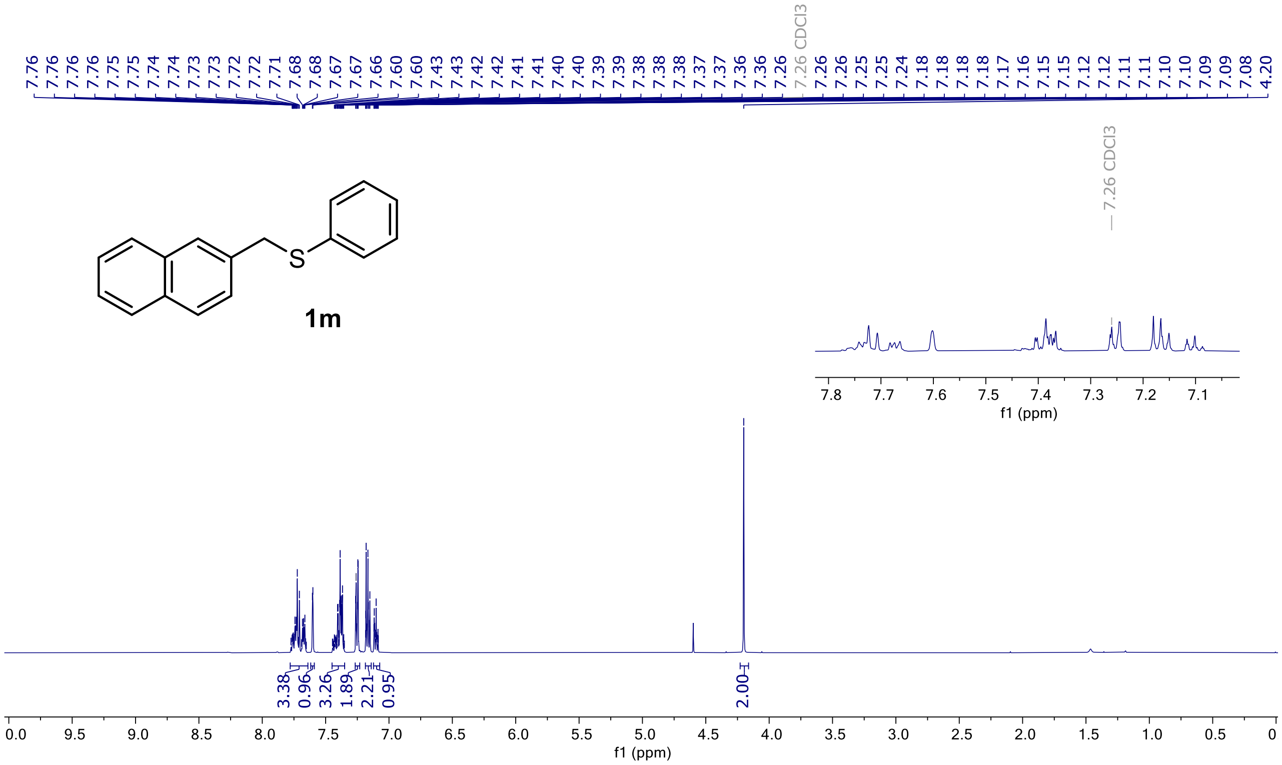

1m – ^{13}C-NMR (126 MHz, $CDCl_3$)

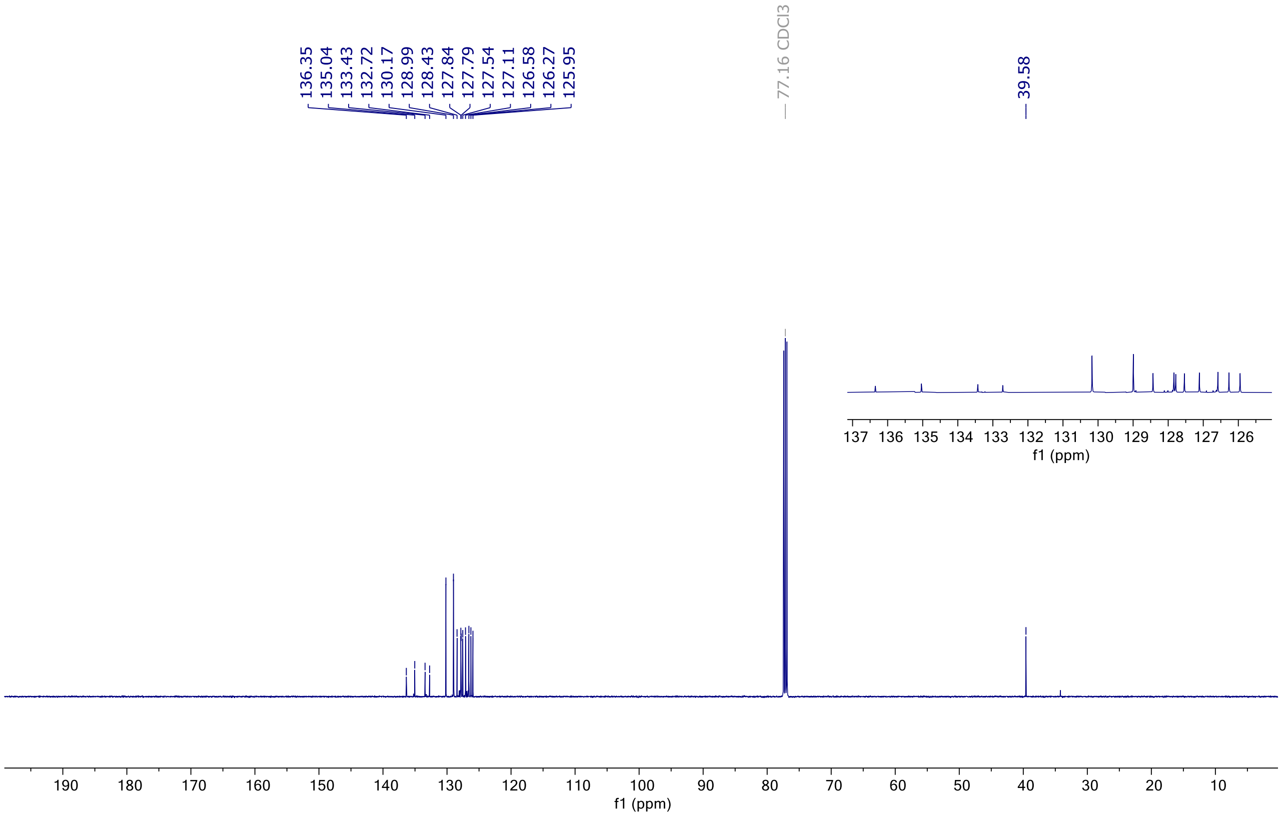

1n – ^{1}H-NMR (400 MHz, $CDCl_3$)

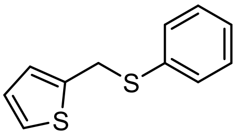

1n

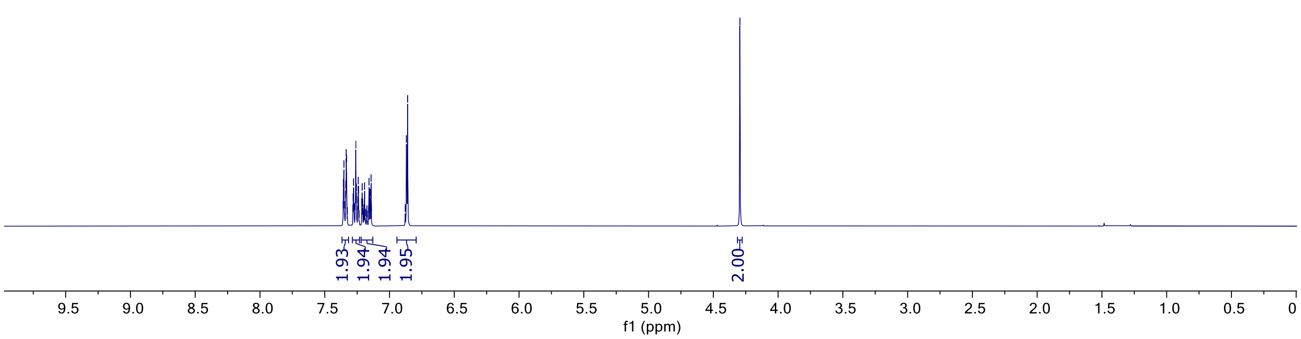

1n – ^{13}C-NMR (101 MHz, $CDCl_3$)

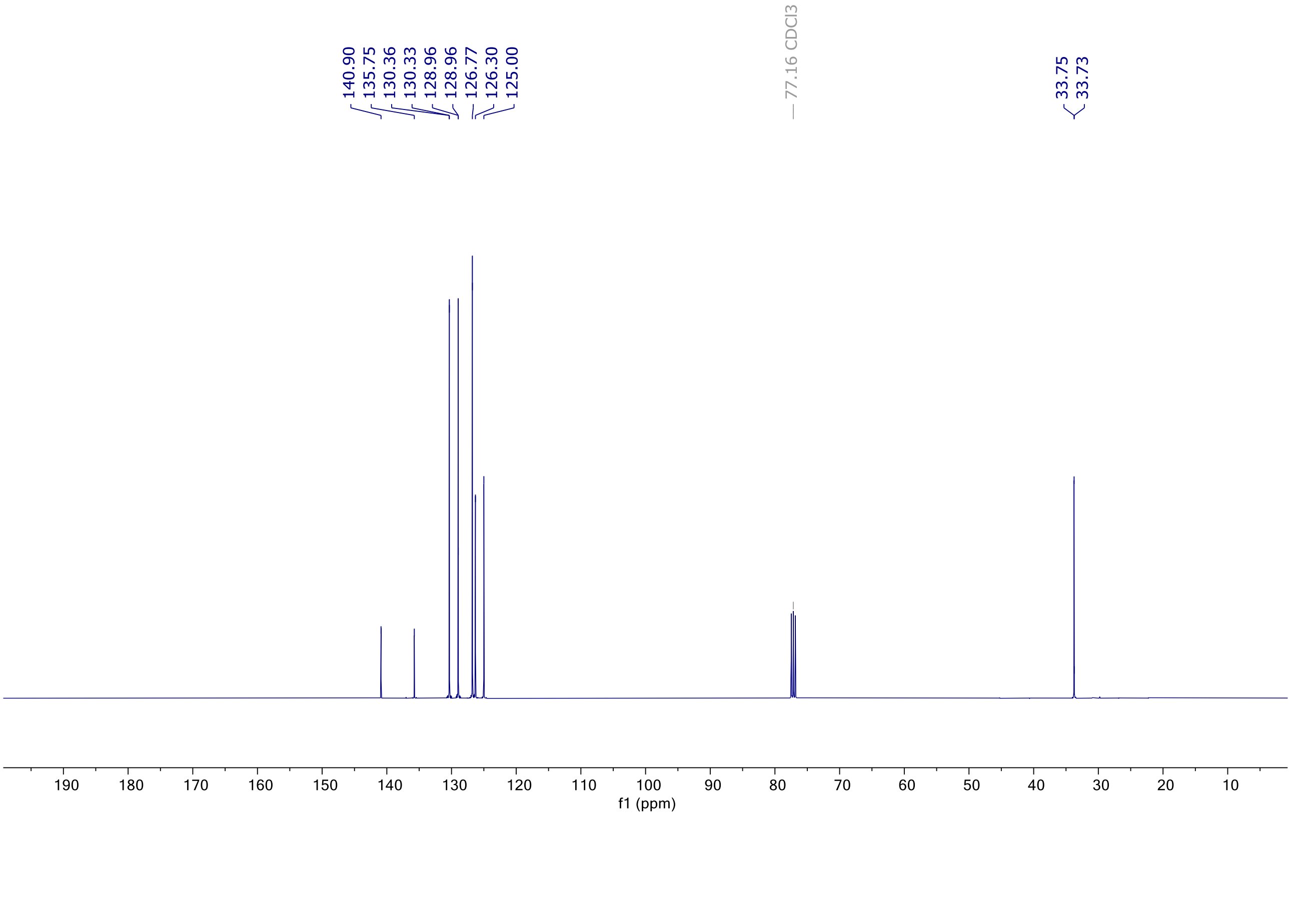

1o – ^{1}H-NMR (500 MHz, CDCl$_3$)

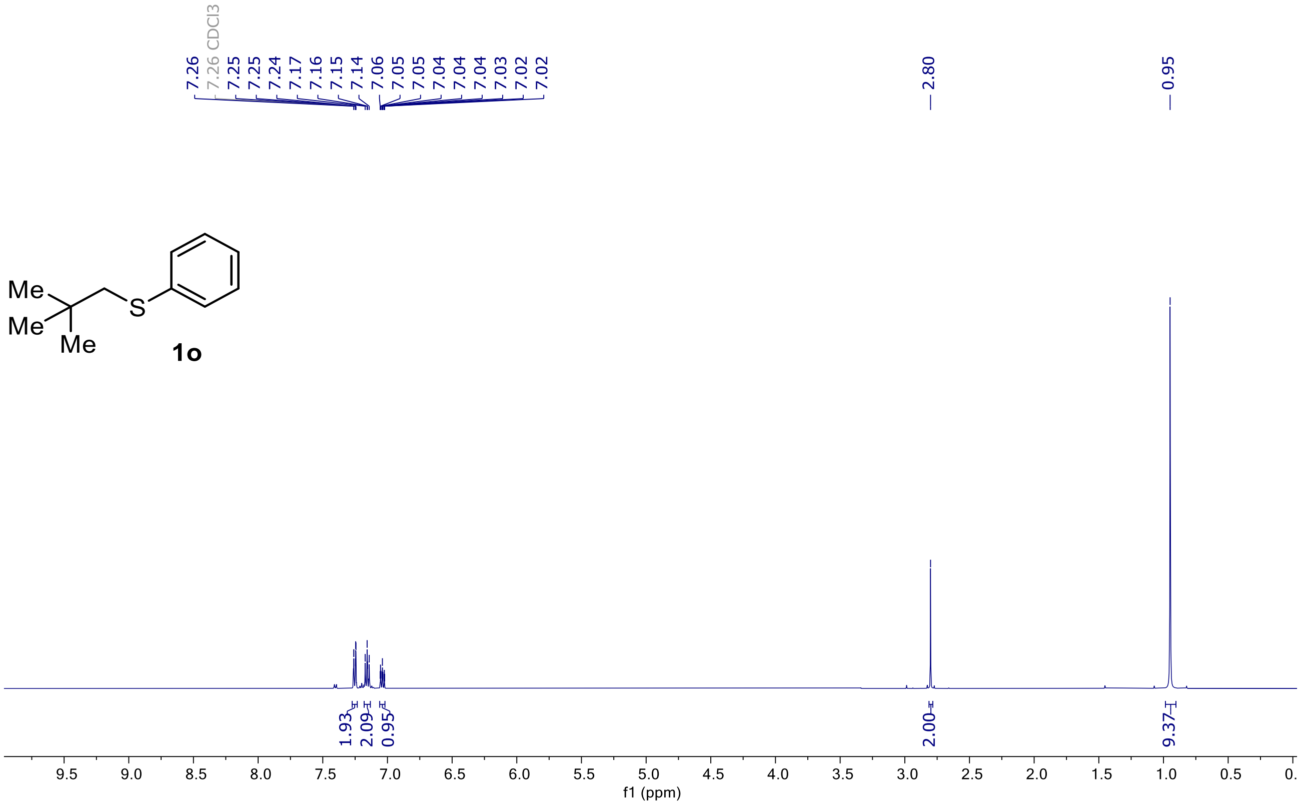

1o – ^{13}C-NMR (126 MHz, CDCl$_3$)

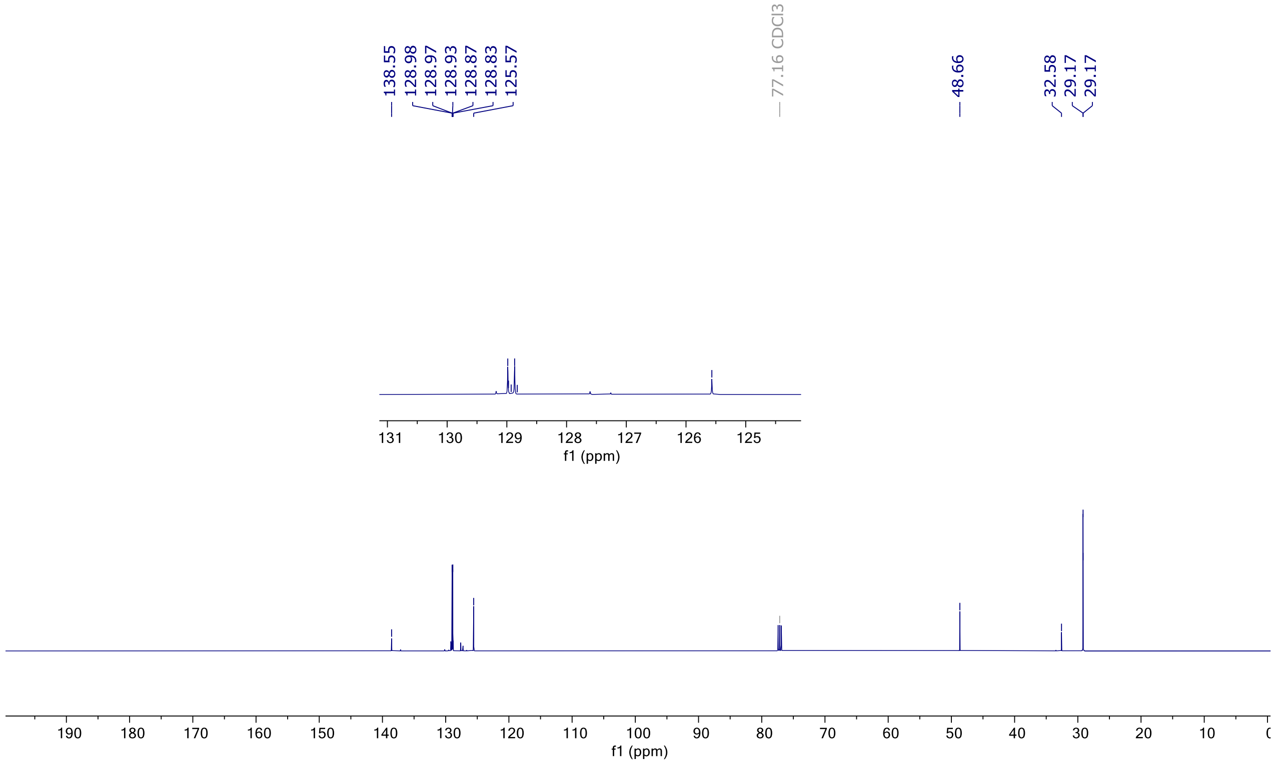

1p – ^{1}H-NMR (500 MHz, $CDCl_3$)

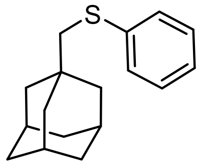

1p

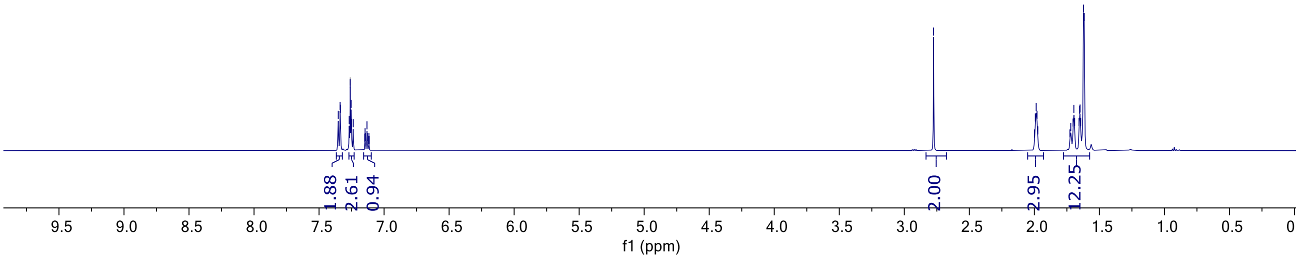

1p – ^{13}C-NMR (126 MHz, $CDCl_3$)

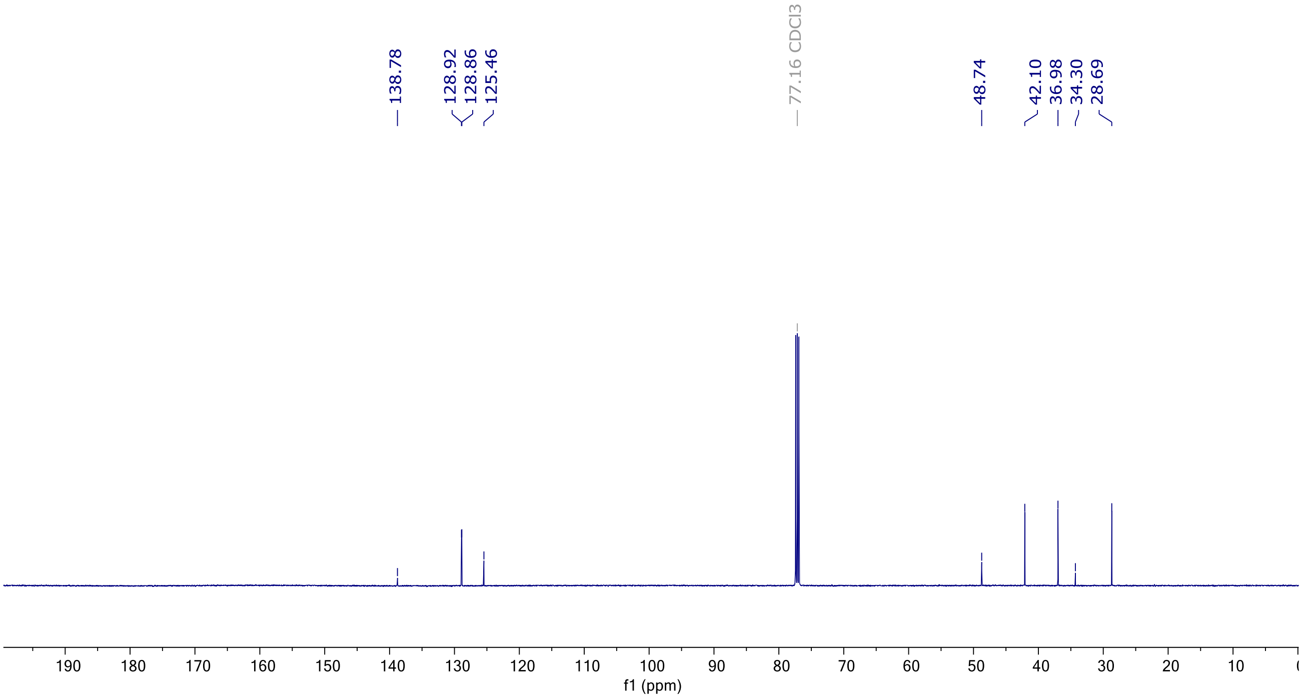

1q – ^{1}H-NMR (400 MHz, $CDCl_3$)

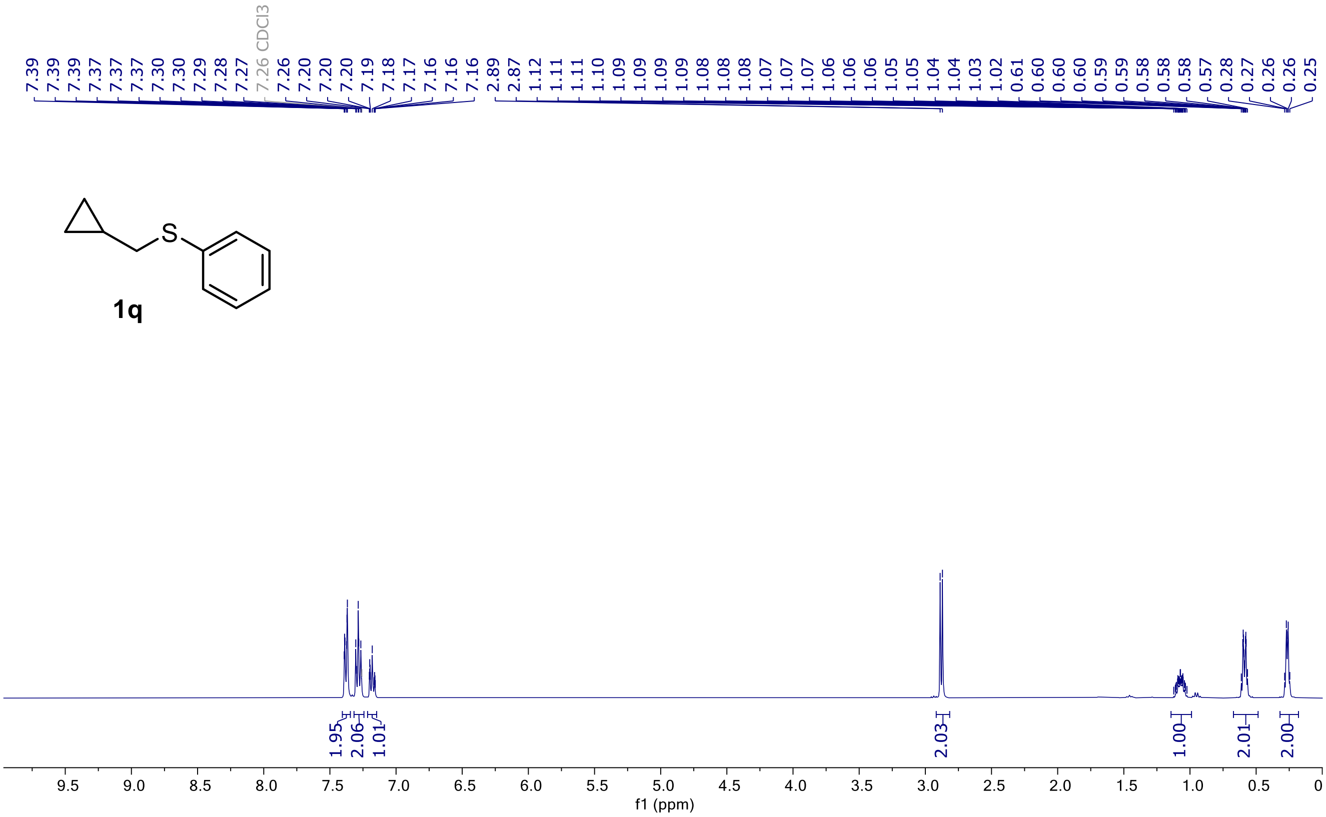

1q – ^{13}C-NMR (101 MHz, $CDCl_3$)

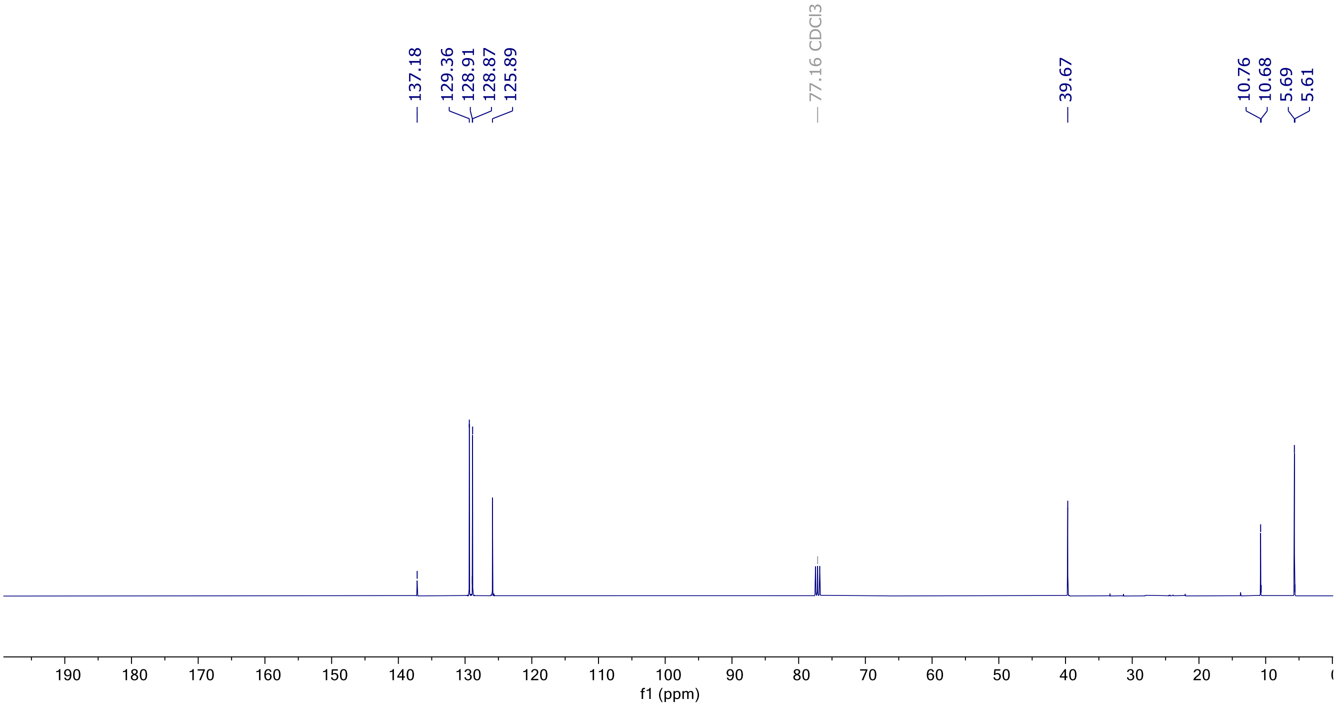

1v – ^{1}H-NMR (400 MHz, CDCl$_3$)

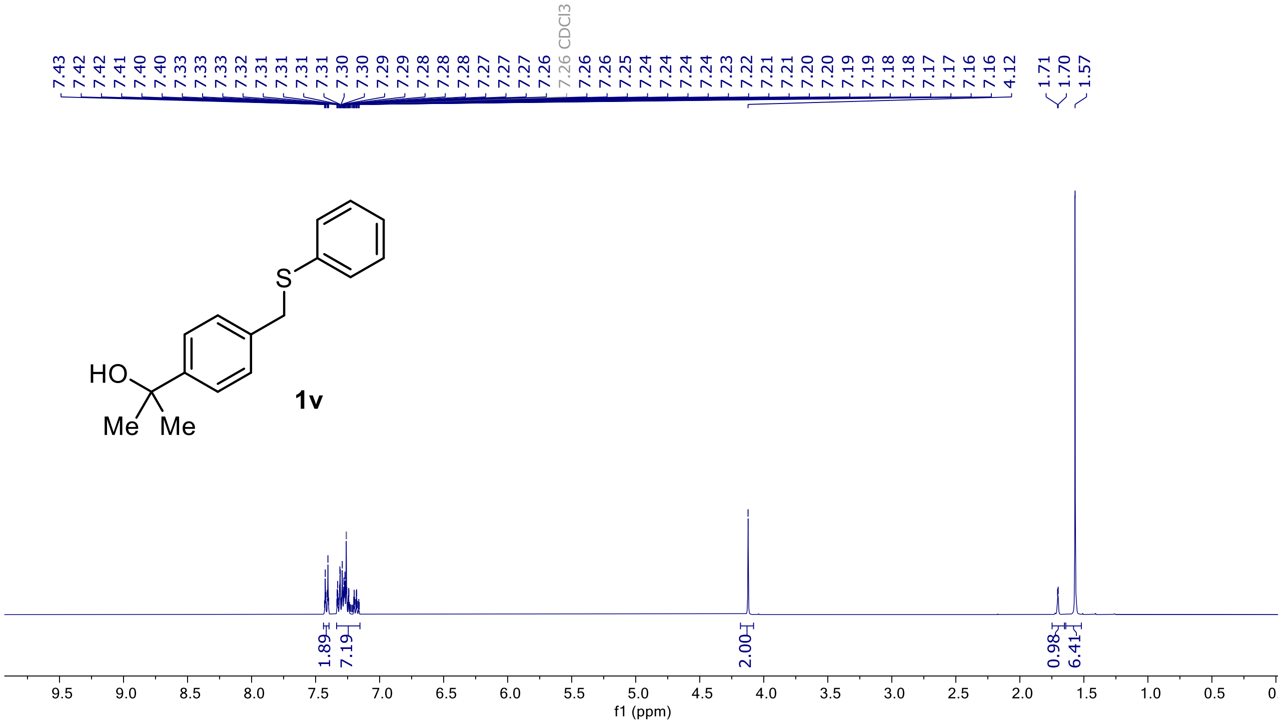

1v – ^{13}C-NMR (101 MHz, CDCl$_3$)

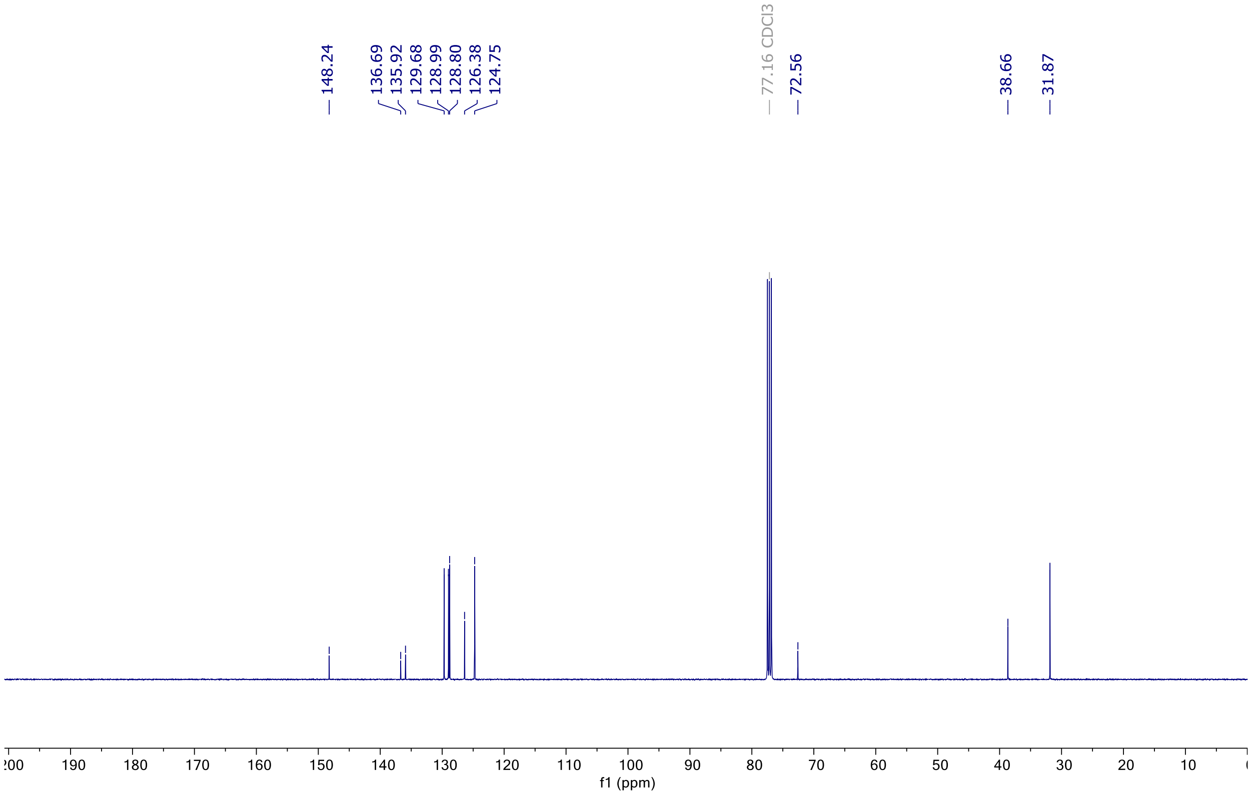

1w – ^{1}H-NMR (400 MHz, $CDCl_3$)

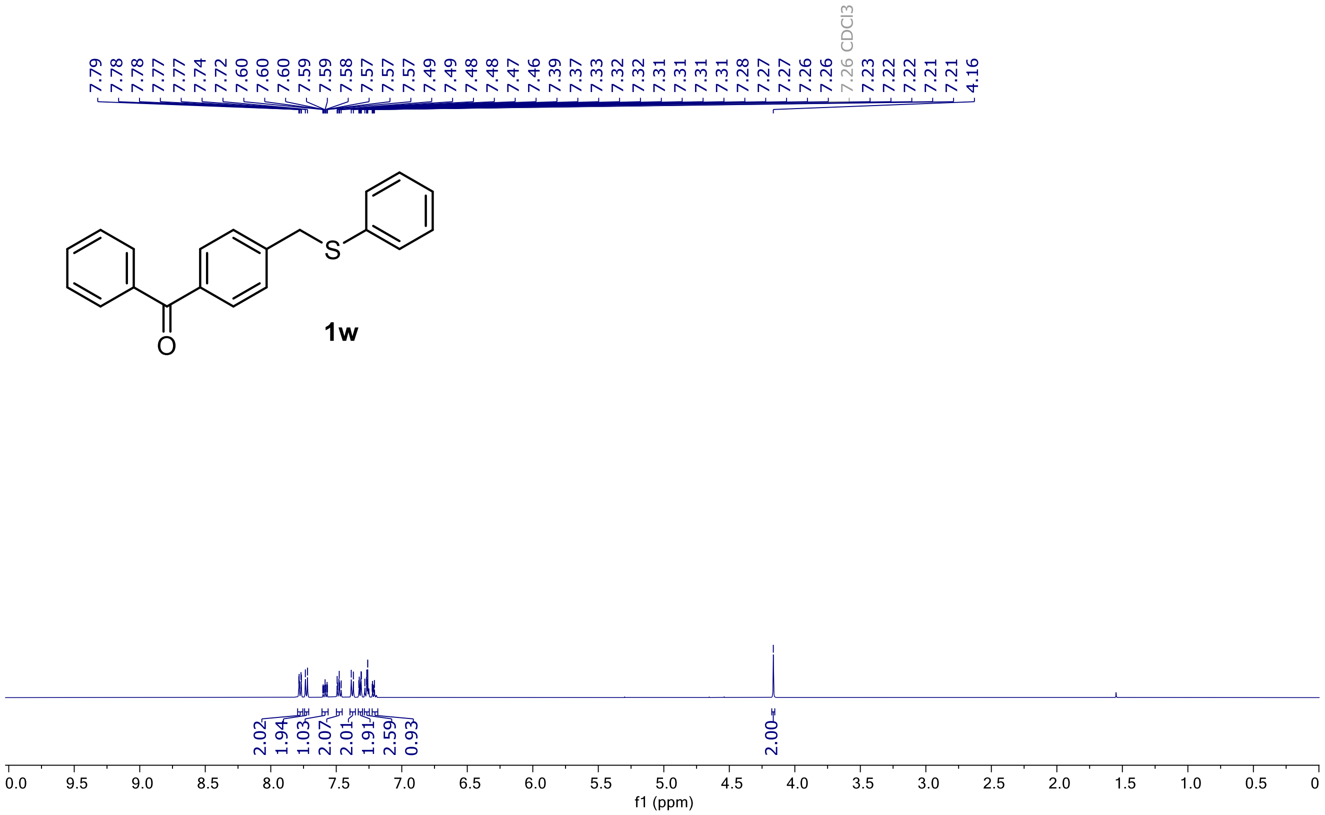

1w – ^{13}C-NMR (101 MHz, $CDCl_3$)

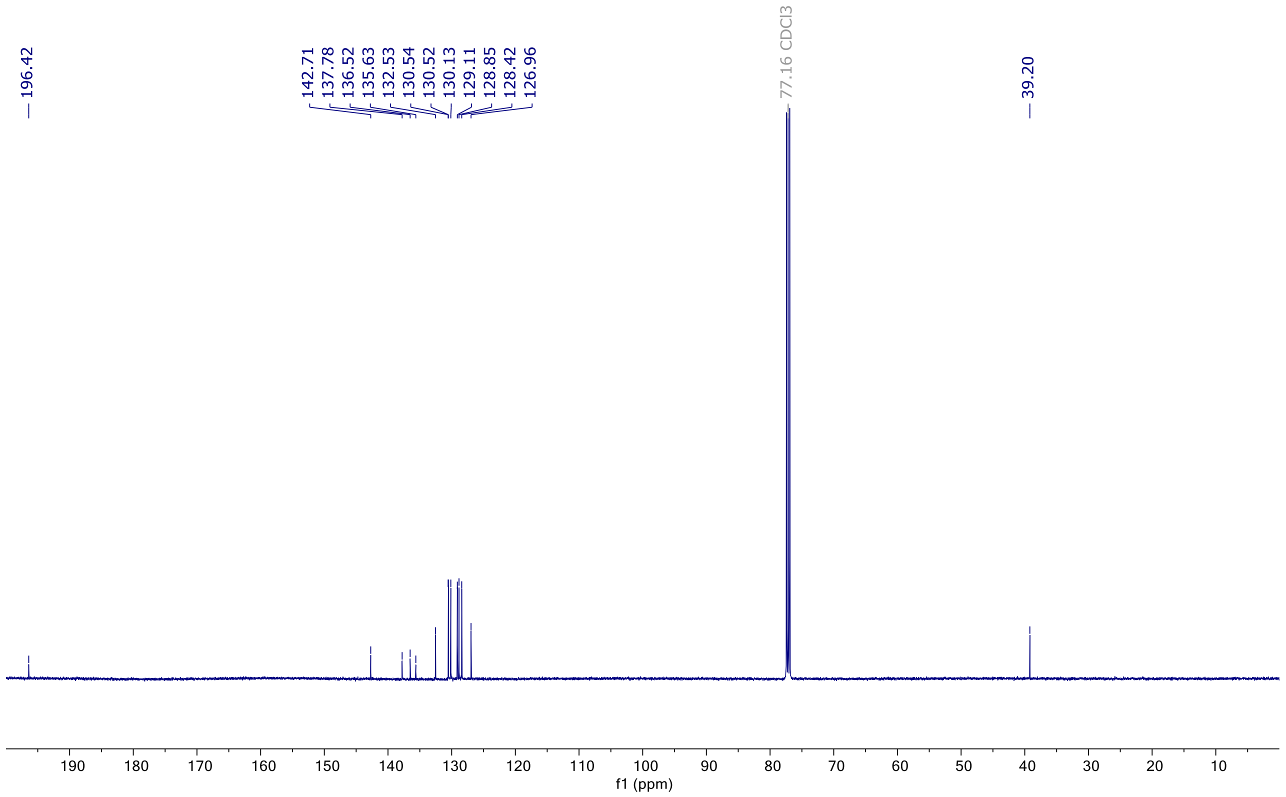

1x – ^{1}H-NMR (400 MHz, $CDCl_3$)

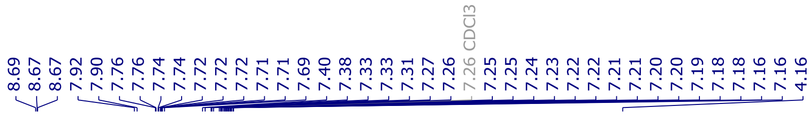

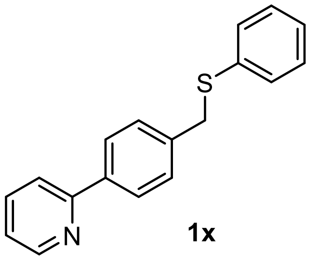

1x

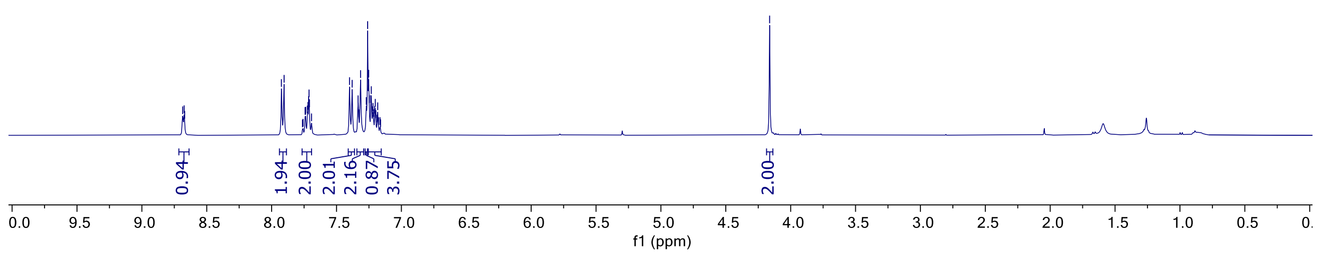

1x – ^{13}C-NMR (101 MHz, $CDCl_3$)

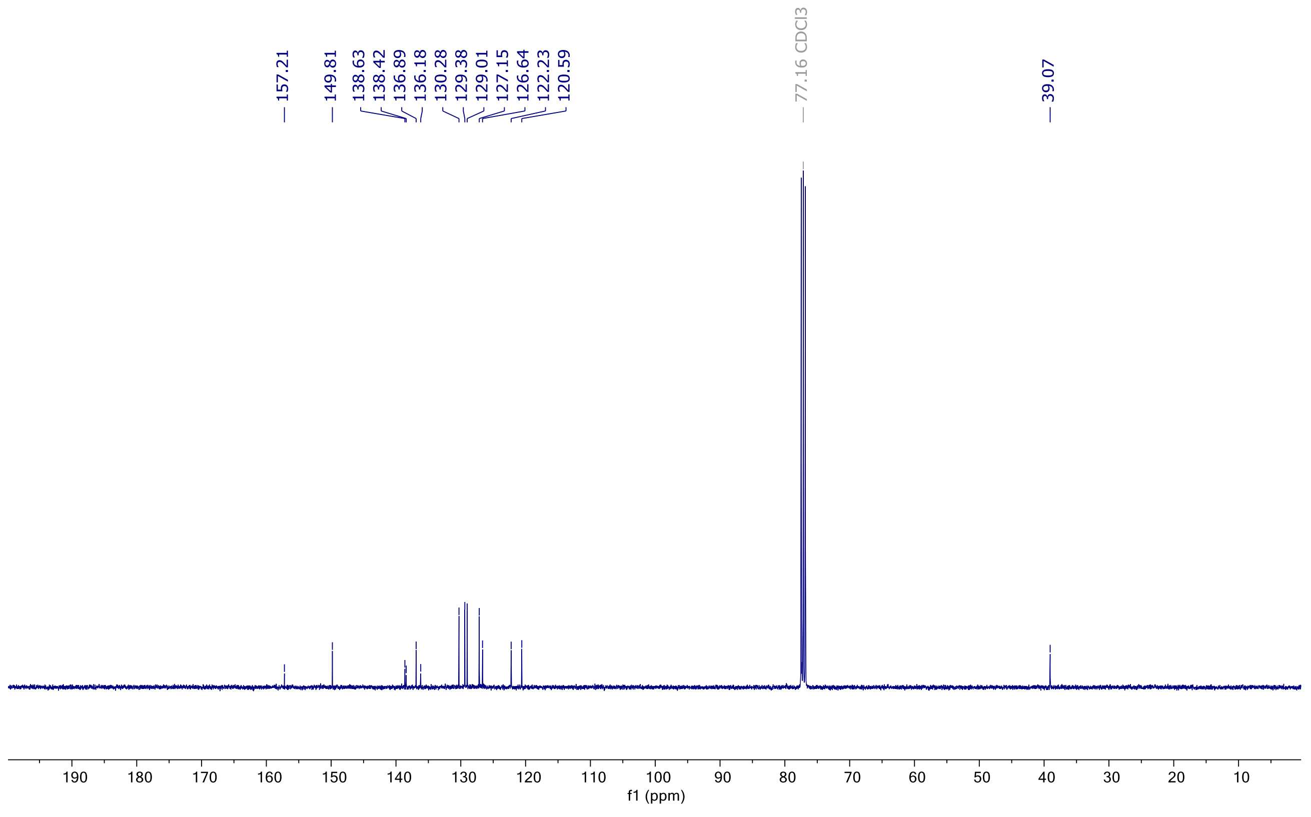

1y – ^{1}H-NMR (500 MHz, CDCl$_3$)

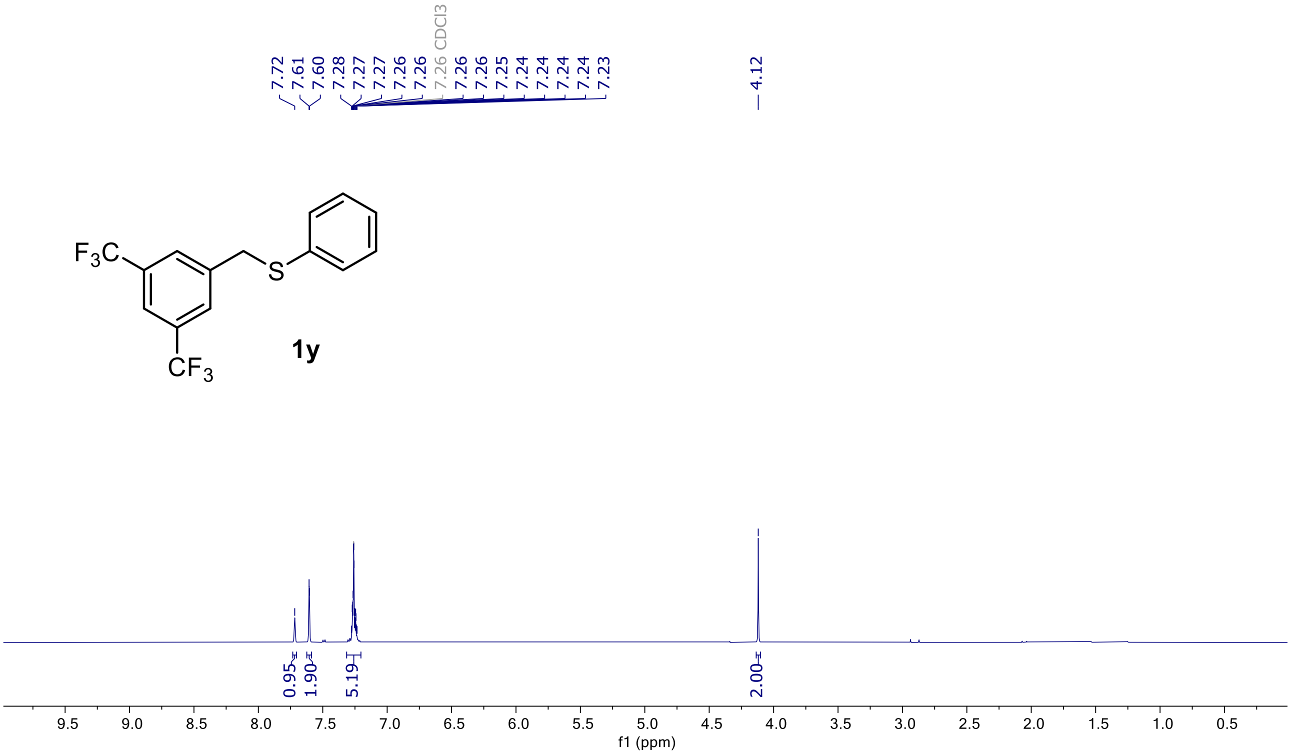

1y – ^{13}C-NMR (126 MHz, CDCl$_3$)

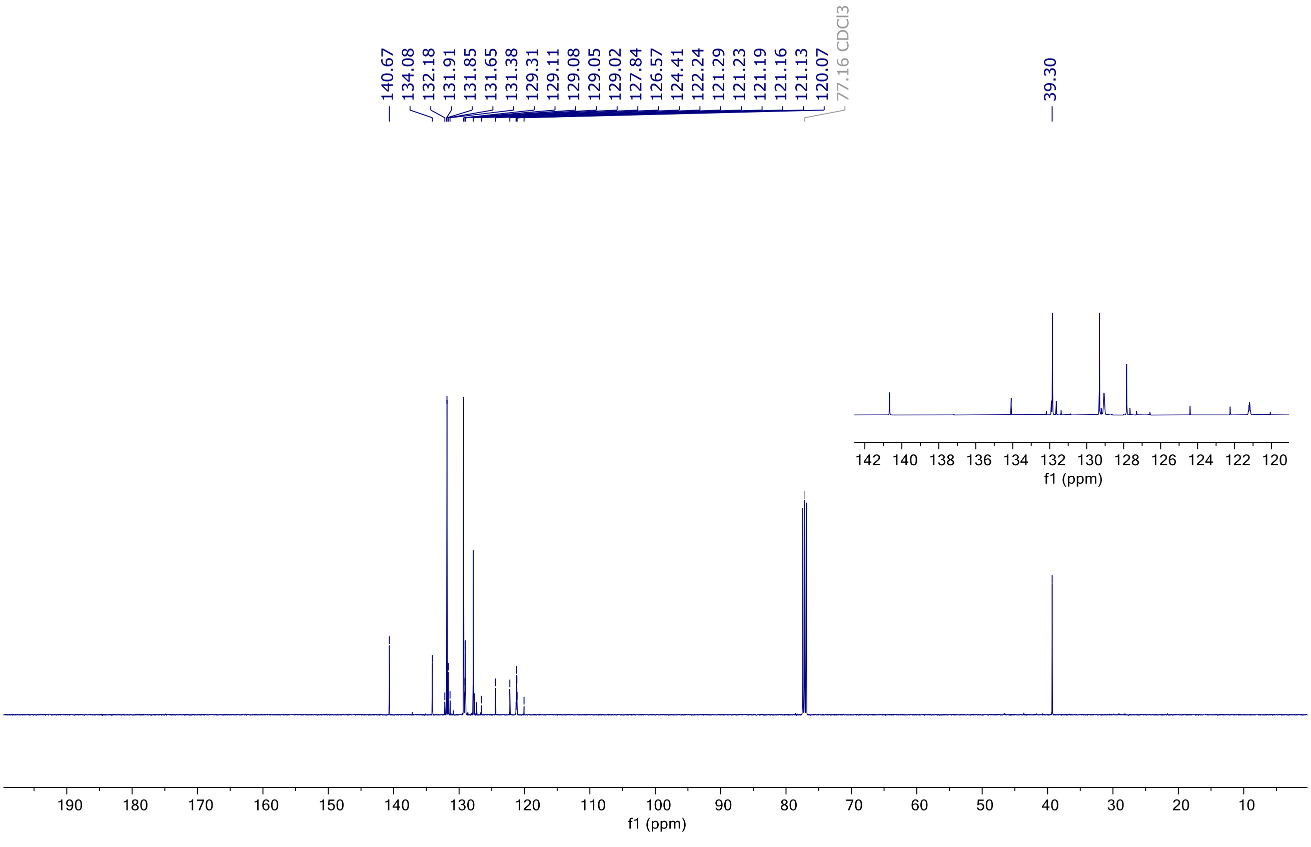

1y – ^{19}F-NMR (376 MHz, $CDCl_3$)

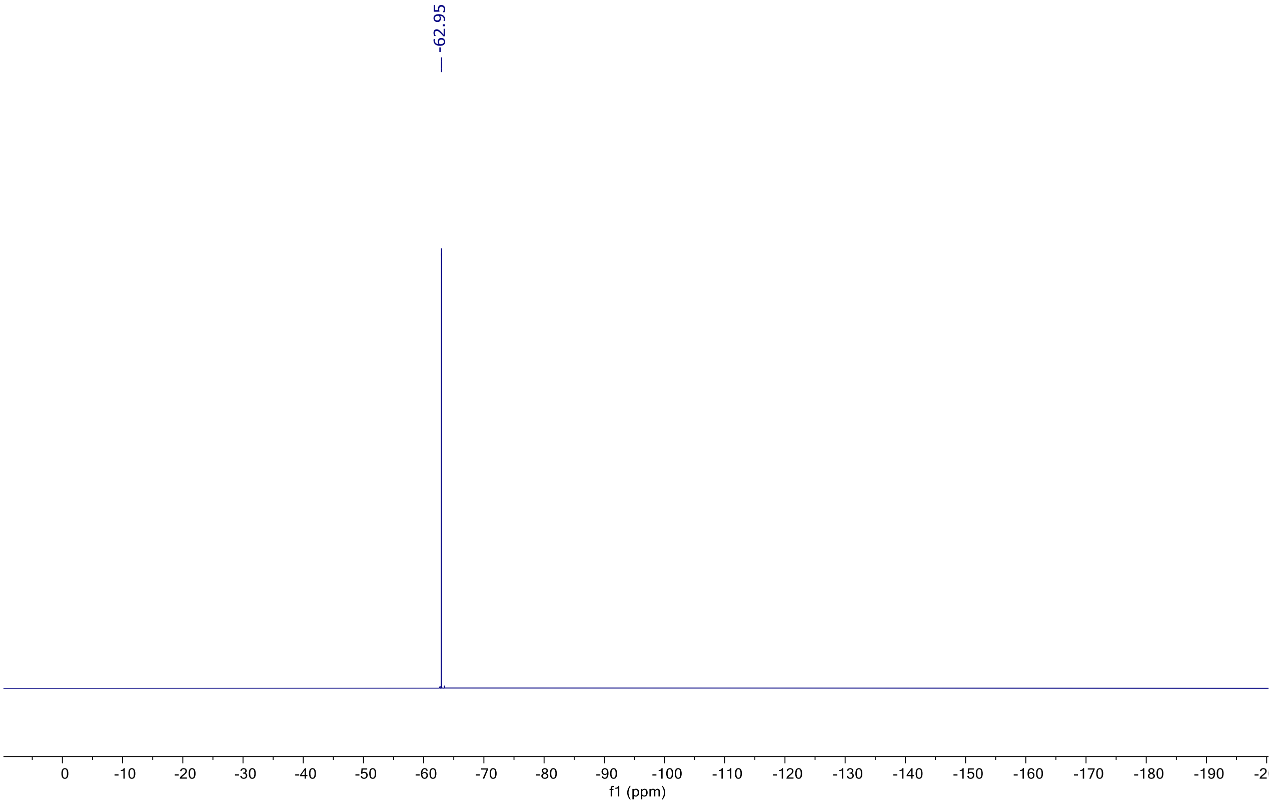

1ca – ^{1}H-NMR (500 MHz, CDCl3)

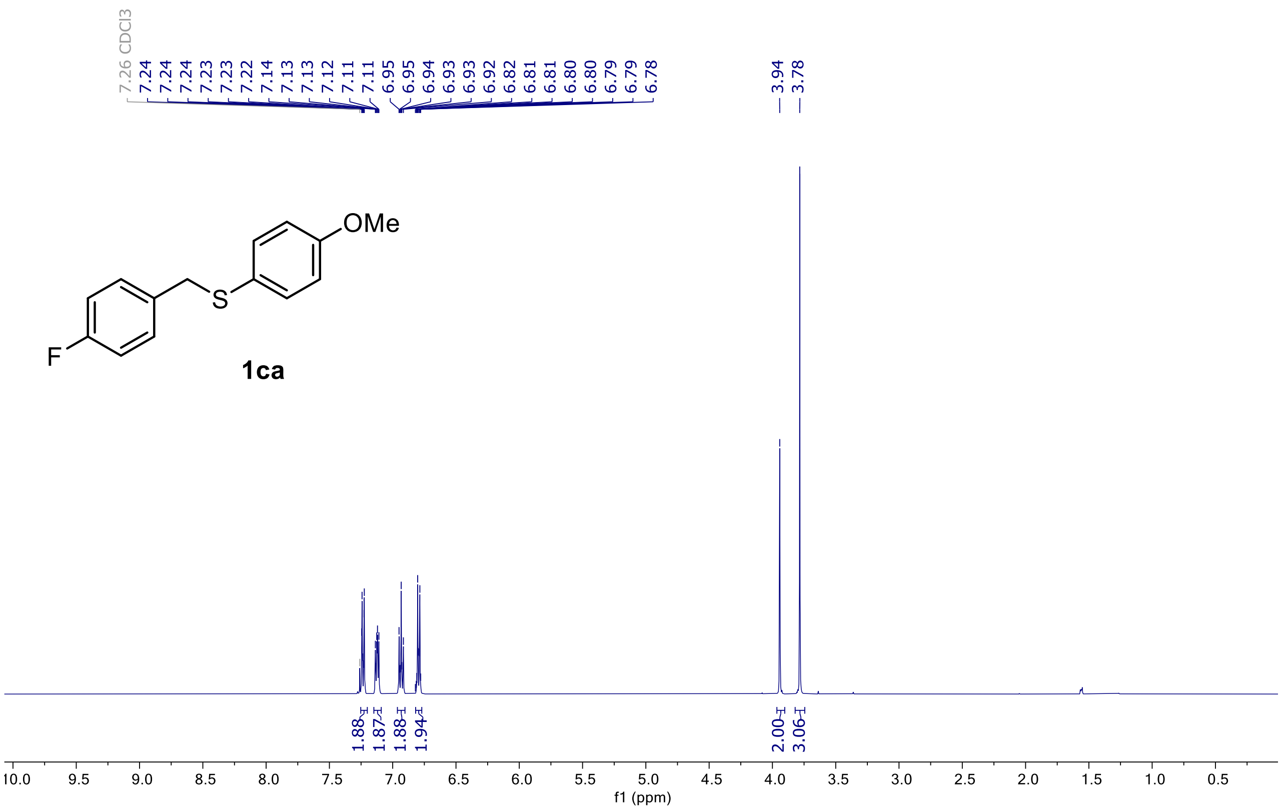

1ca – ^{13}C-NMR (126 MHz, CDCl3)

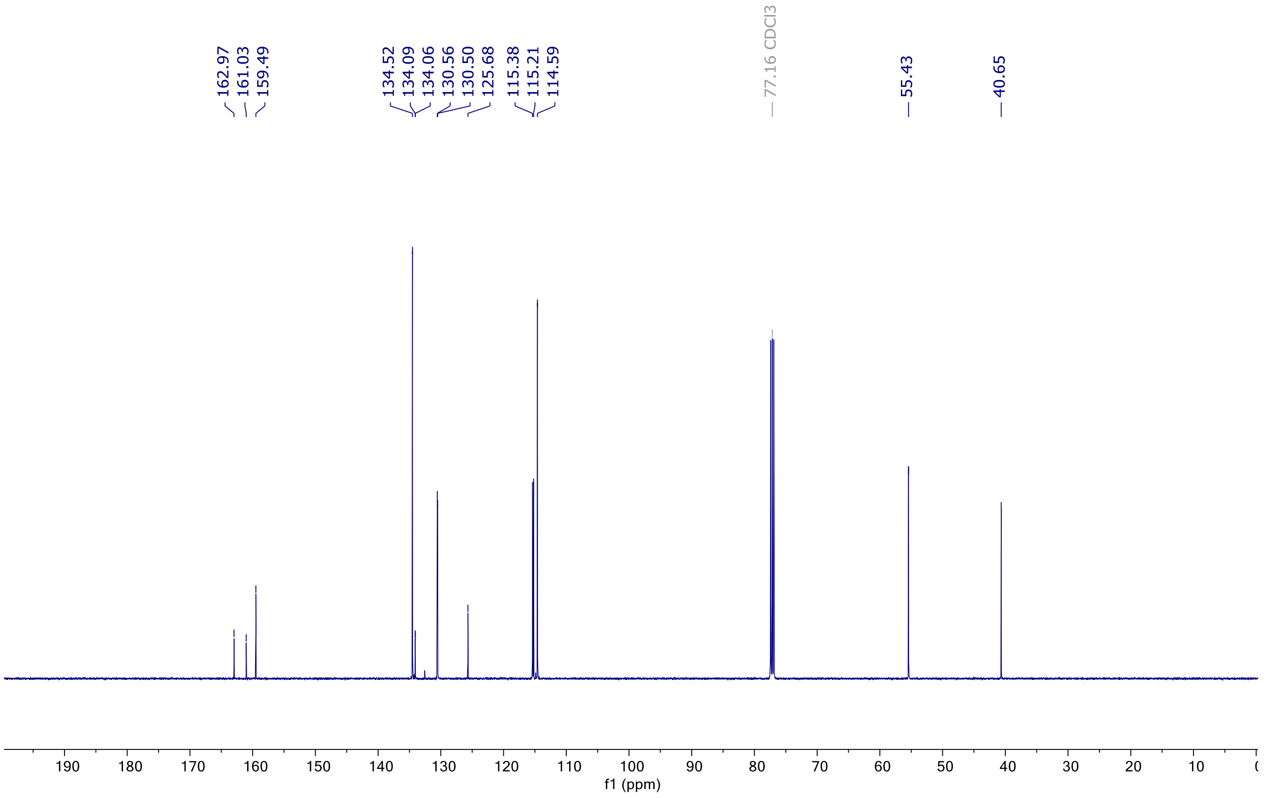

1cb – ¹H-NMR (500 MHz, $CDCl_3$)

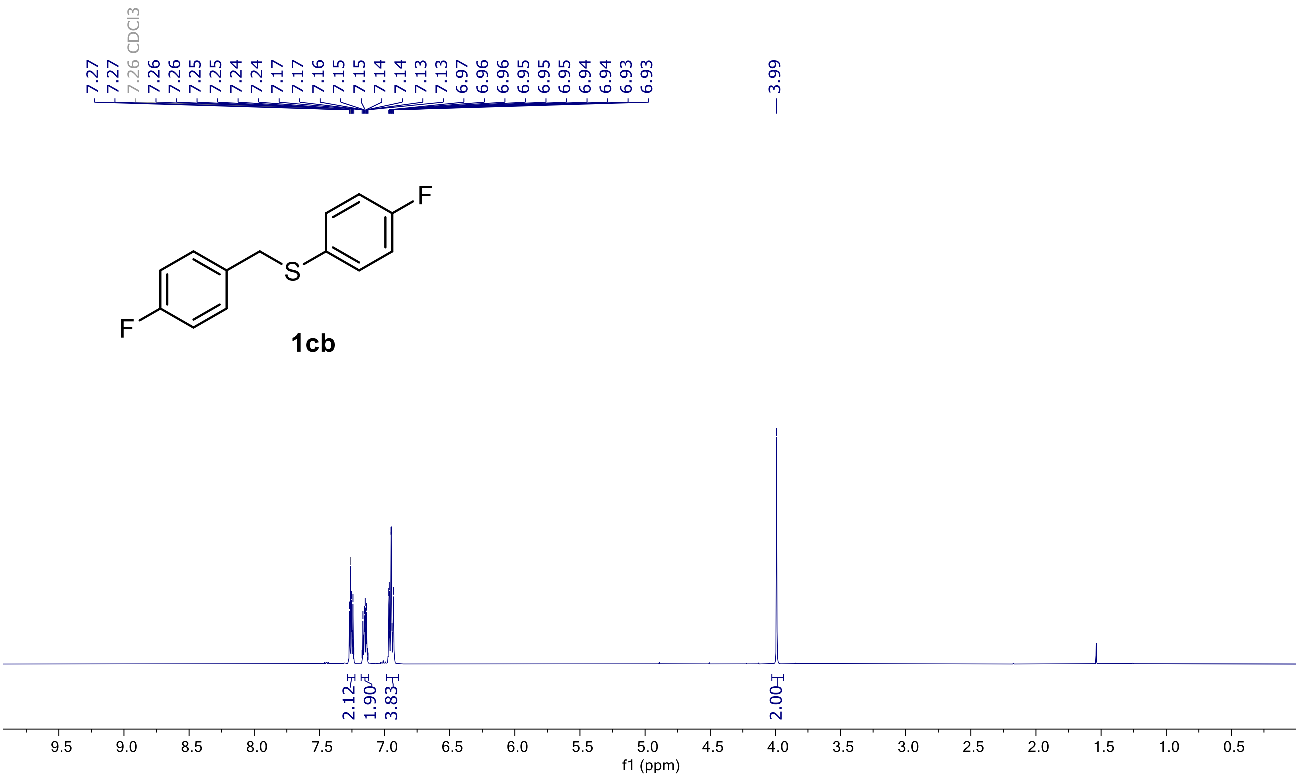

1cb – ¹³C-NMR (126 MHz, $CDCl_3$)

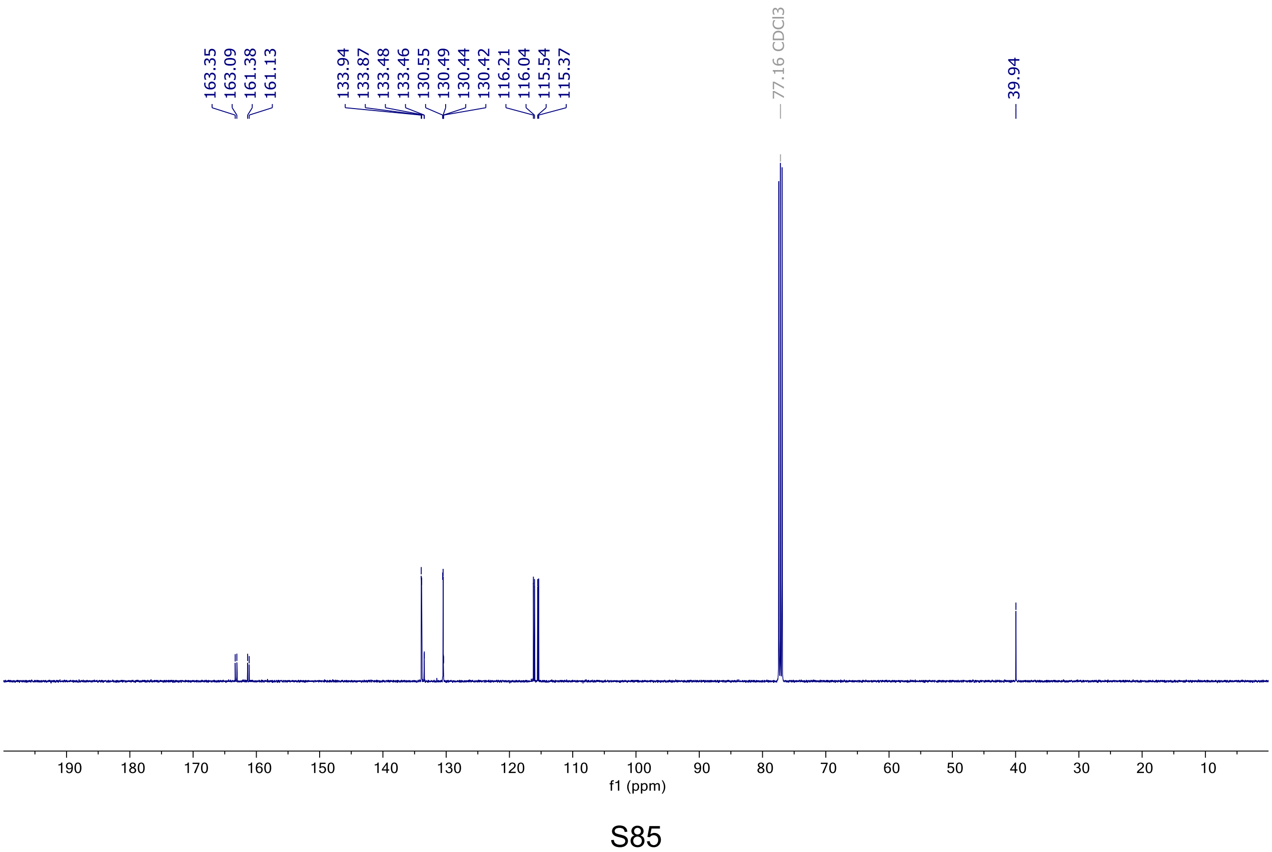

1cc – ^{1}H-NMR (500 MHz, $CDCl_3$)

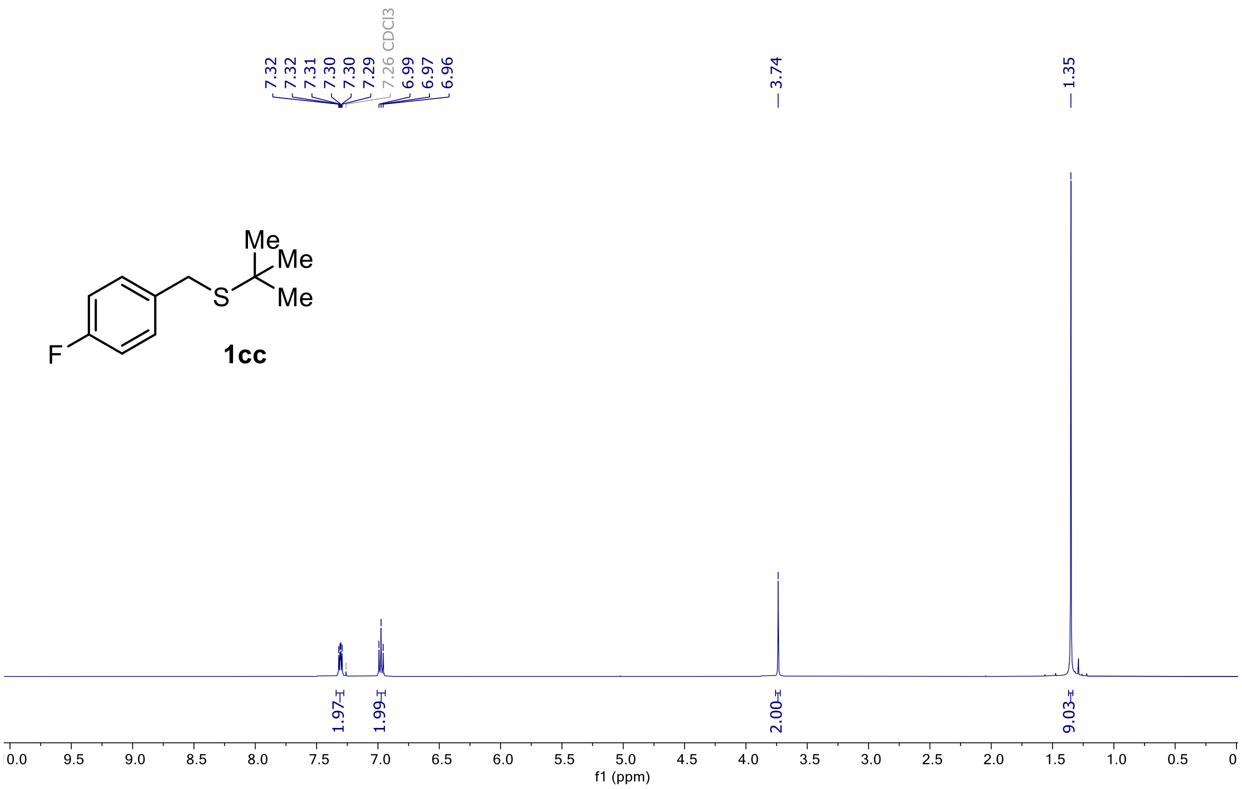

1cc – ^{13}C-NMR (126 MHz, $CDCl_3$)

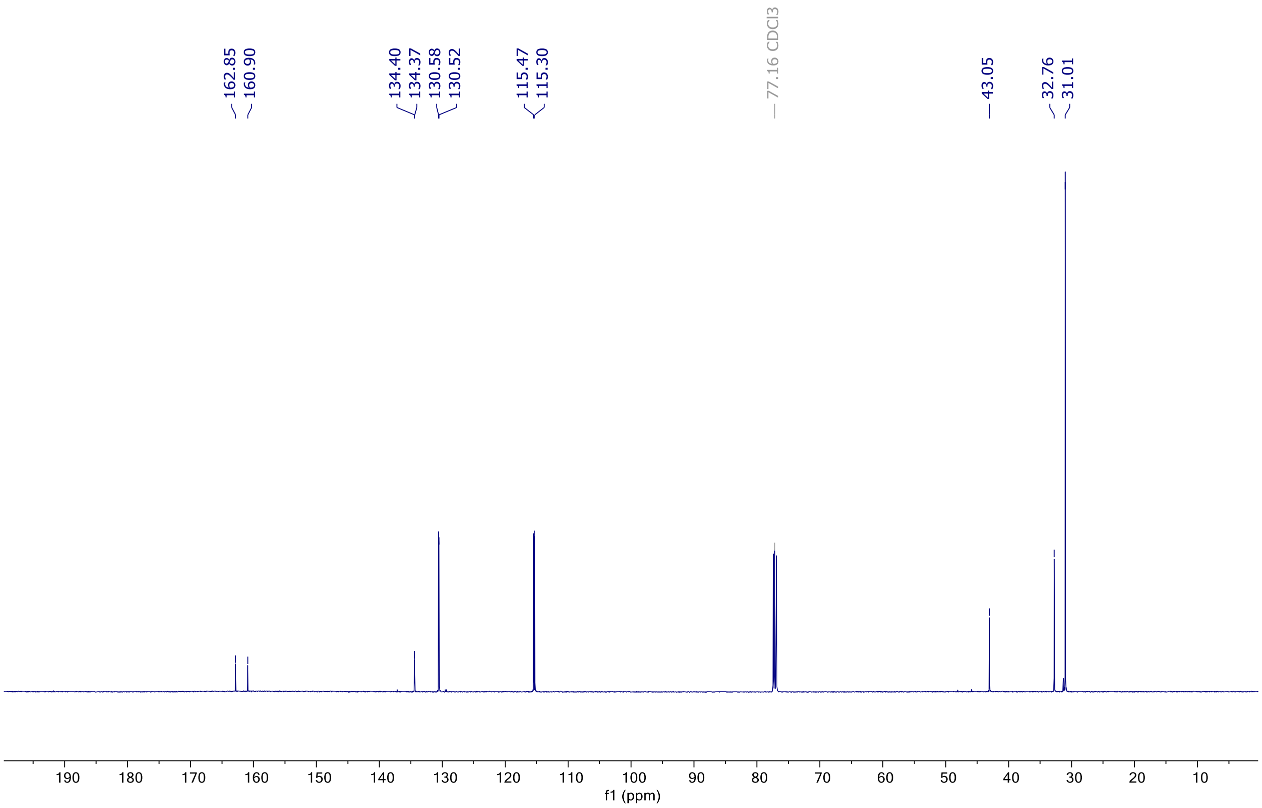

1cc – ^{19}F NMR (471 MHz, $CDCl_3$)

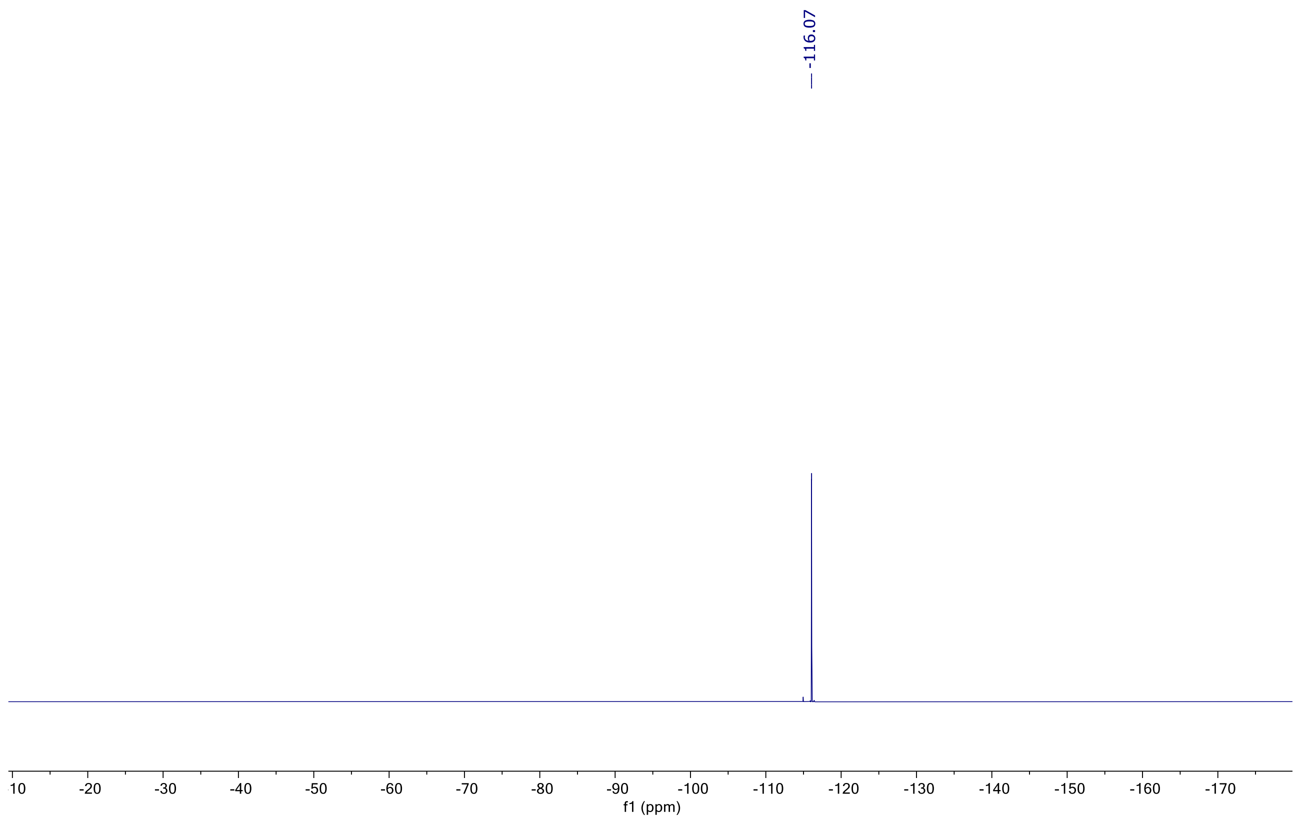

1cd – ^{1}H-NMR (500 MHz, $CDCl_3$)

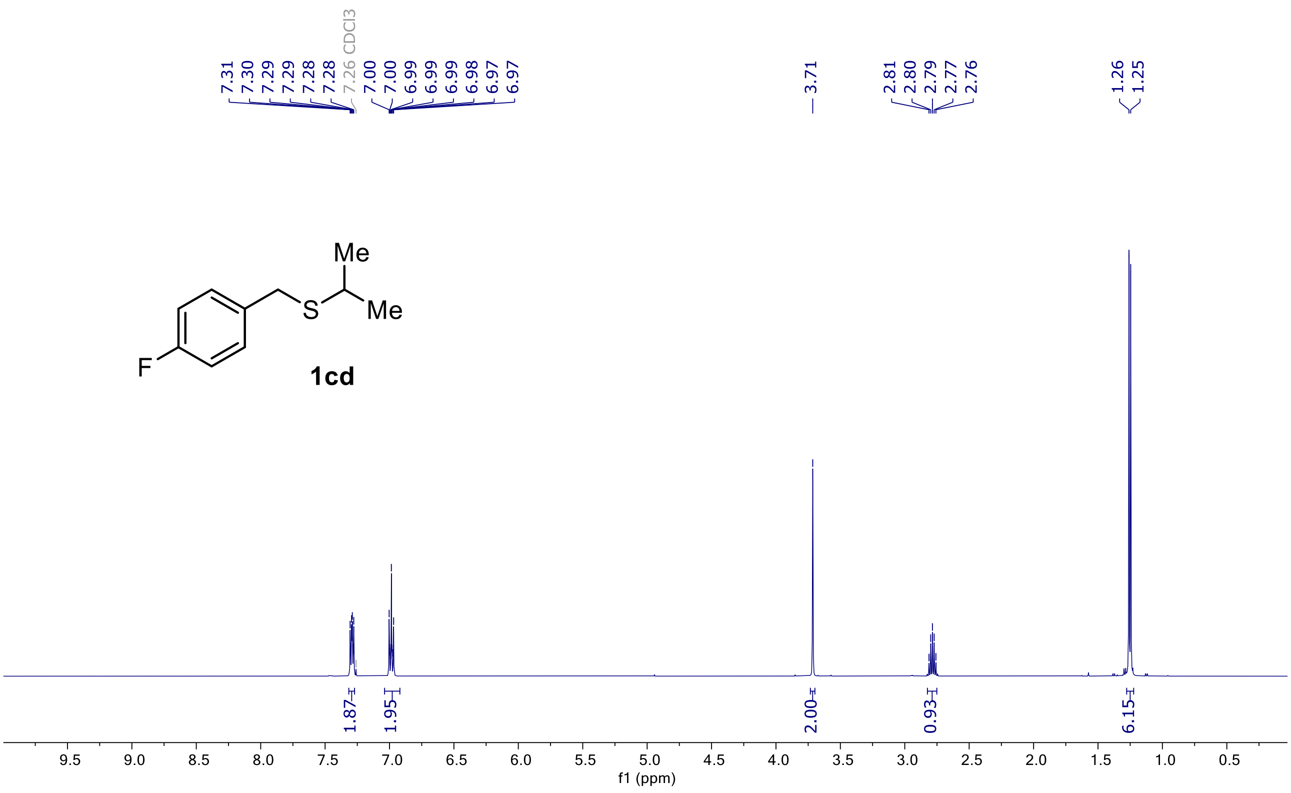

1cd – ^{13}C-NMR (126 MHz, $CDCl_3$)

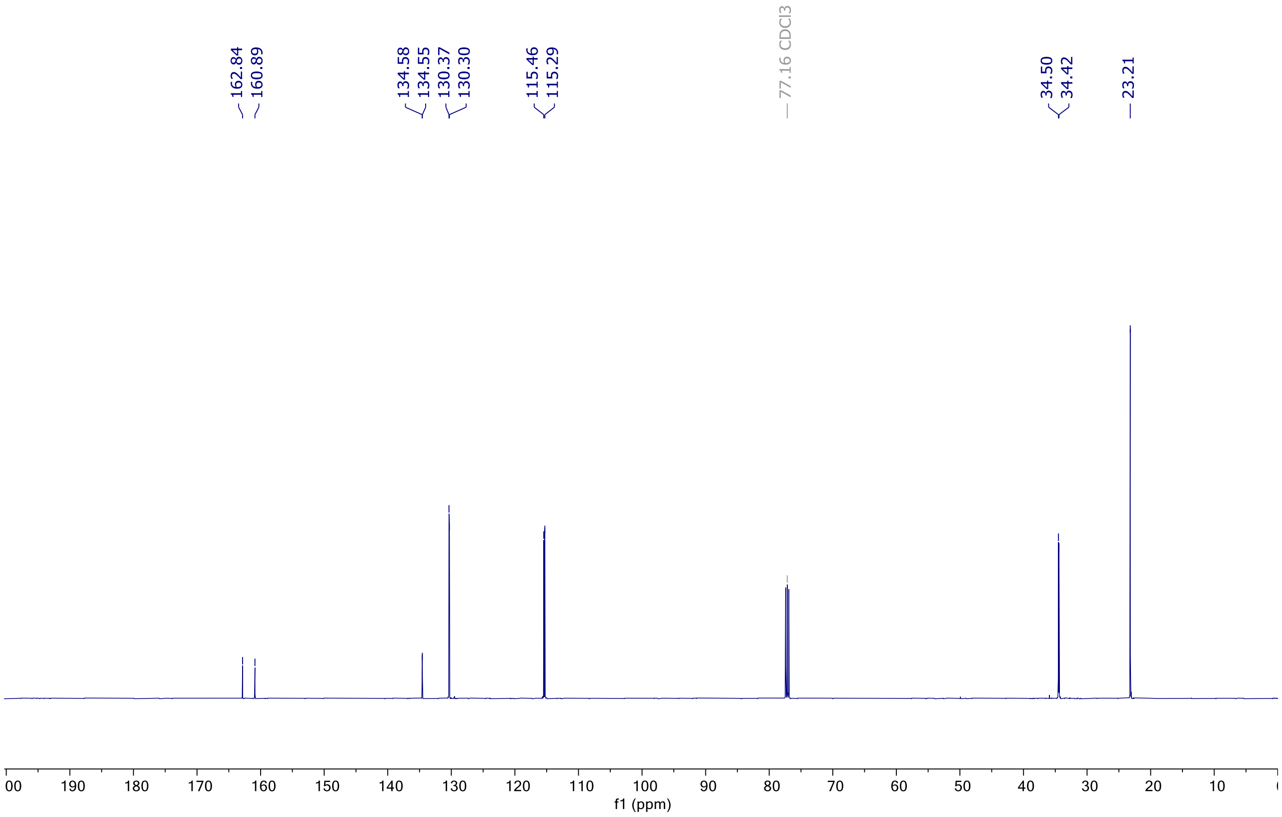

1cd – ^{19}F NMR (471 MHz, $CDCl_3$)

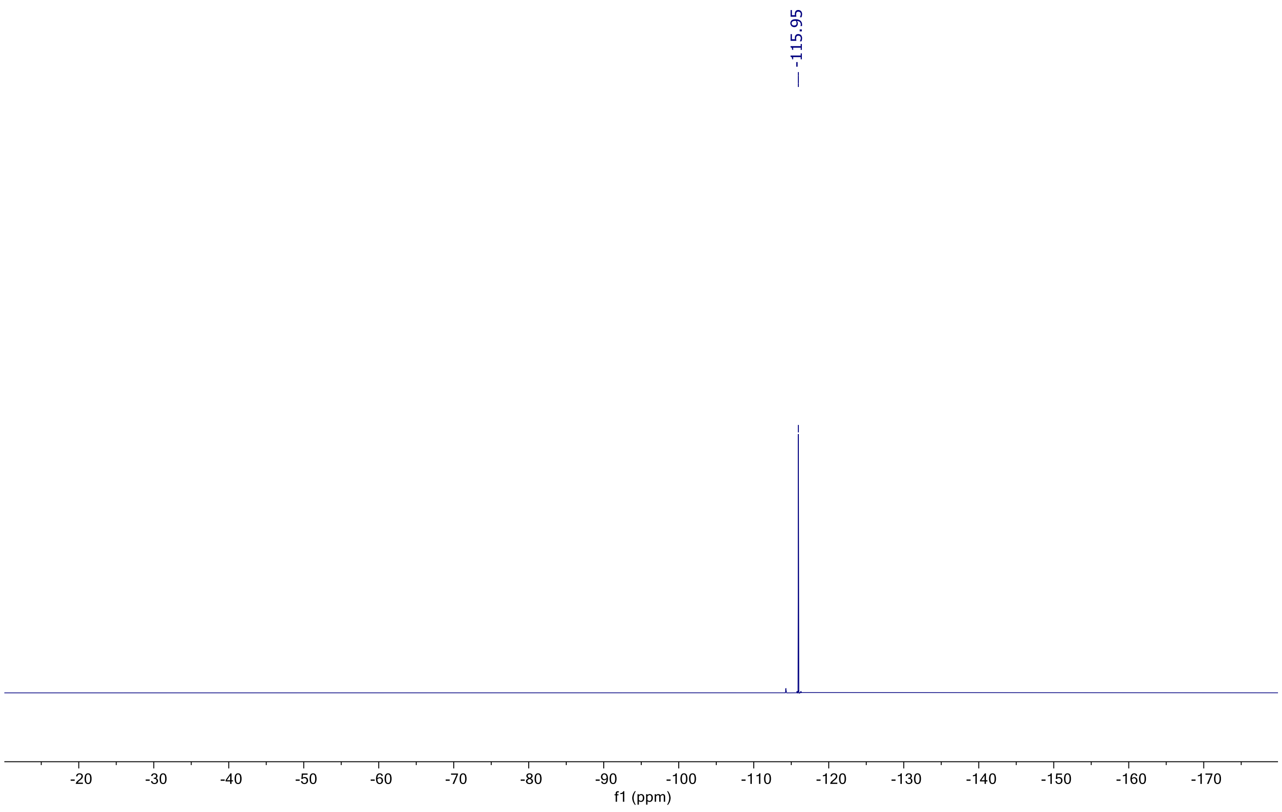

1ce – ^{1}H-NMR (500 MHz, CDCl$_3$)

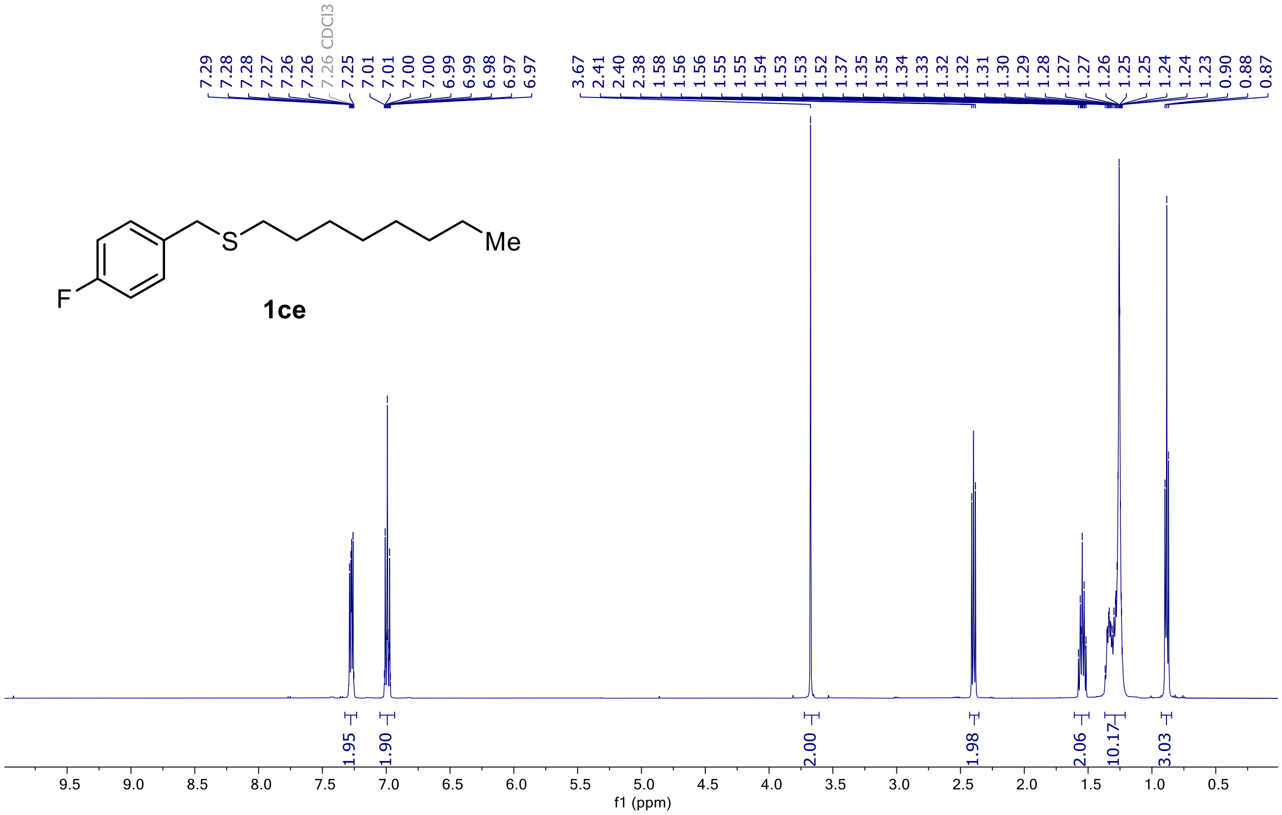

1ce – ^{13}C-NMR (126 MHz, CDCl$_3$)

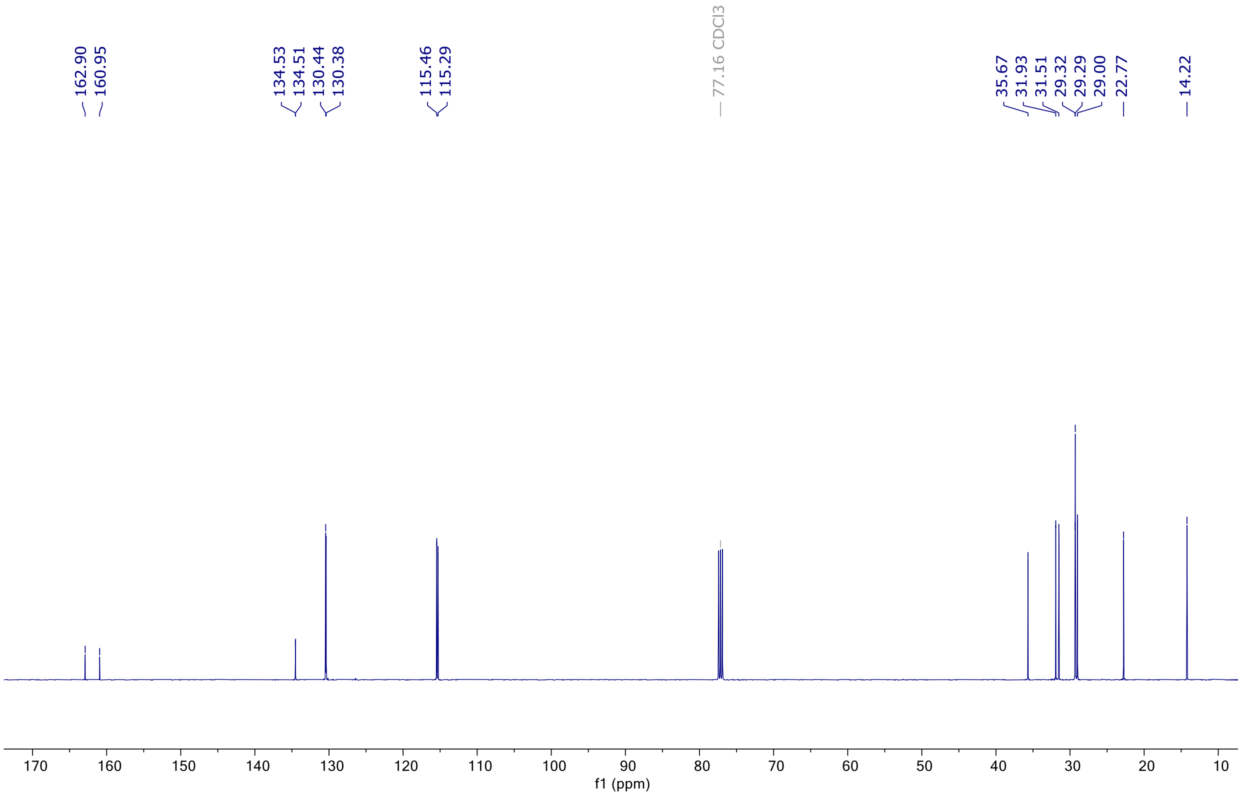

1aa – ^{1}H-NMR (400 MHz, $CDCl_3$)

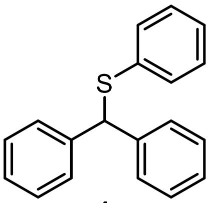

1aa

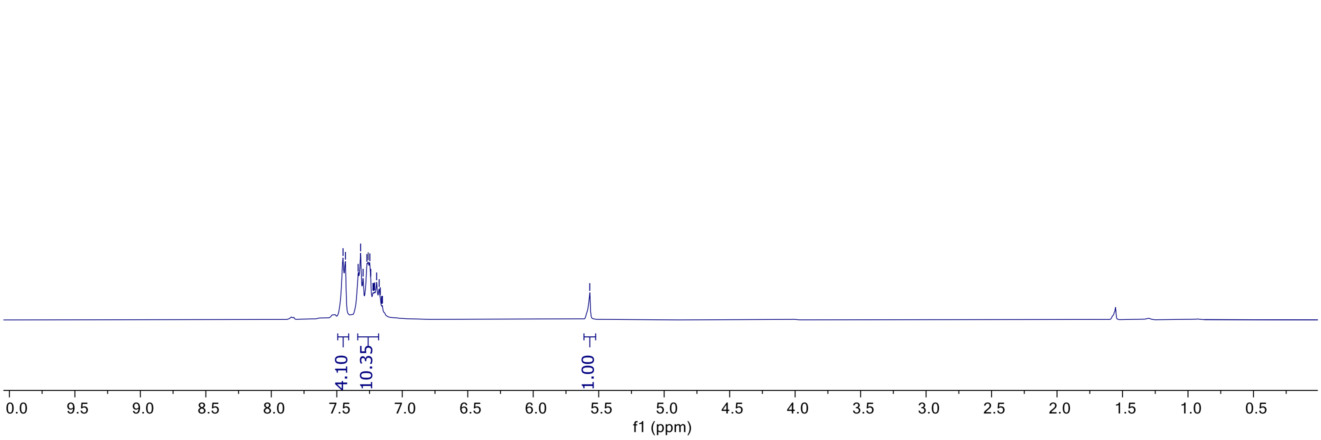

1aa – ^{13}C-NMR (101 MHz, $CDCl_3$)

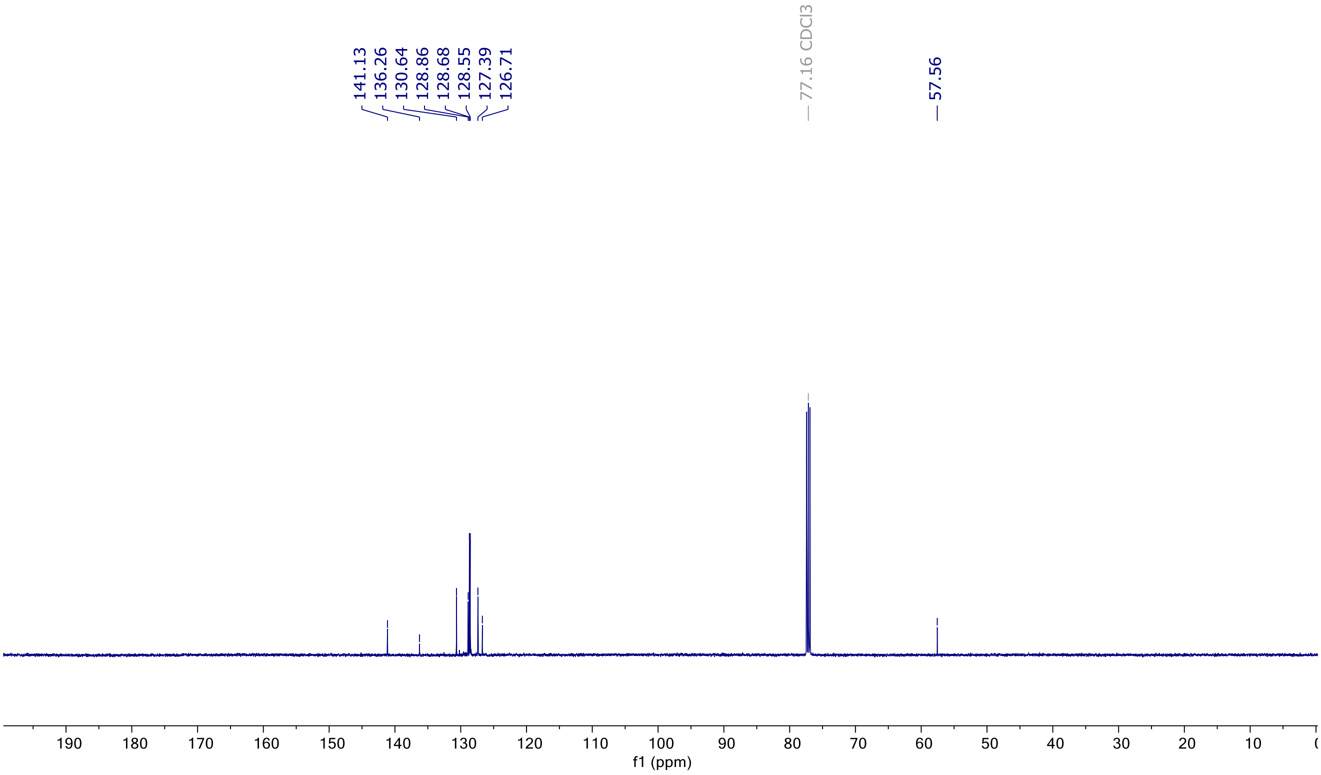

1ab – ¹H-NMR (400 MHz, CDCl₃)

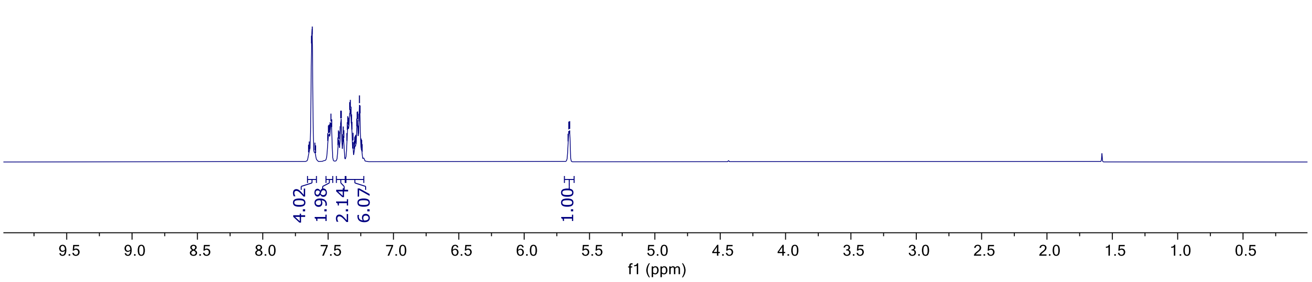

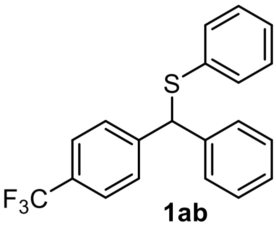

1ab

1ab – ¹³C-NMR (101 MHz, CDCl₃)

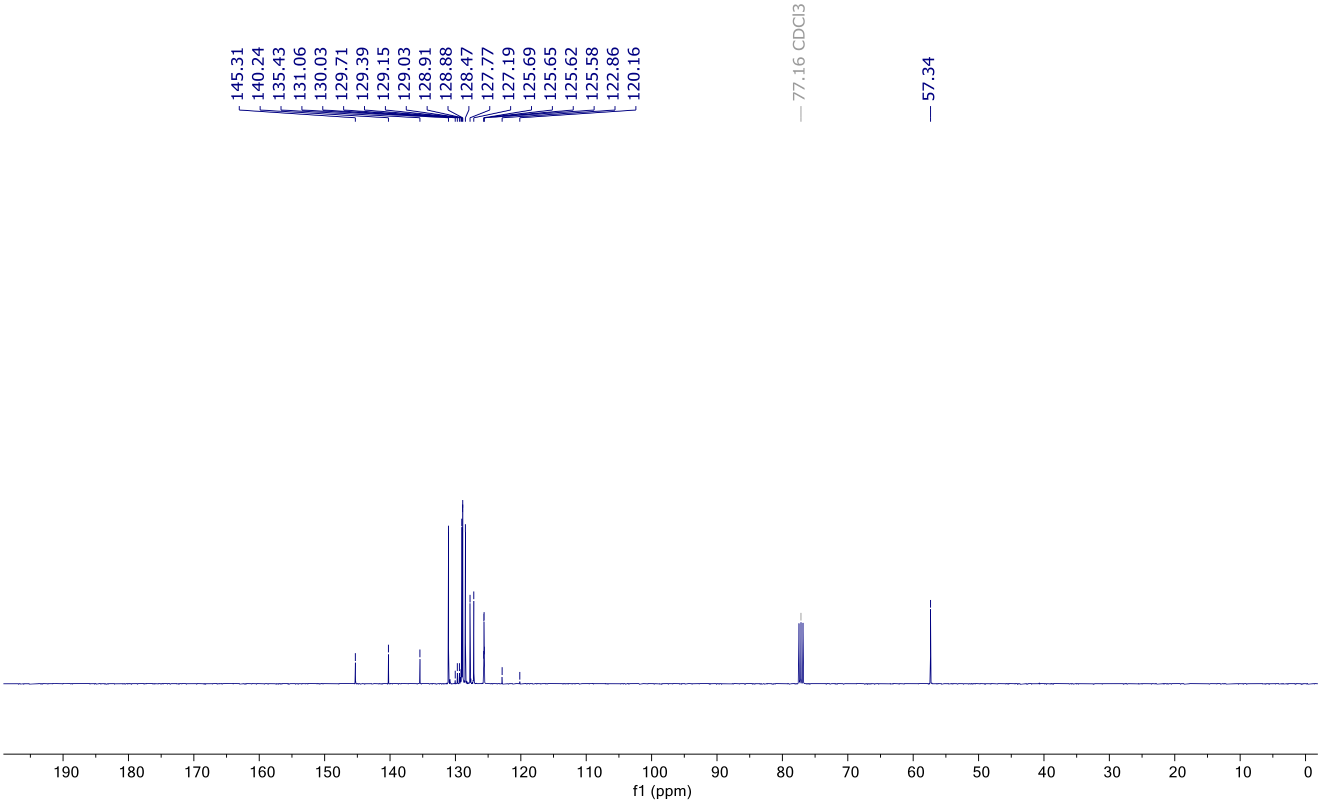

1ab – ^{19}F NMR (376 MHz, $CDCl_3$)

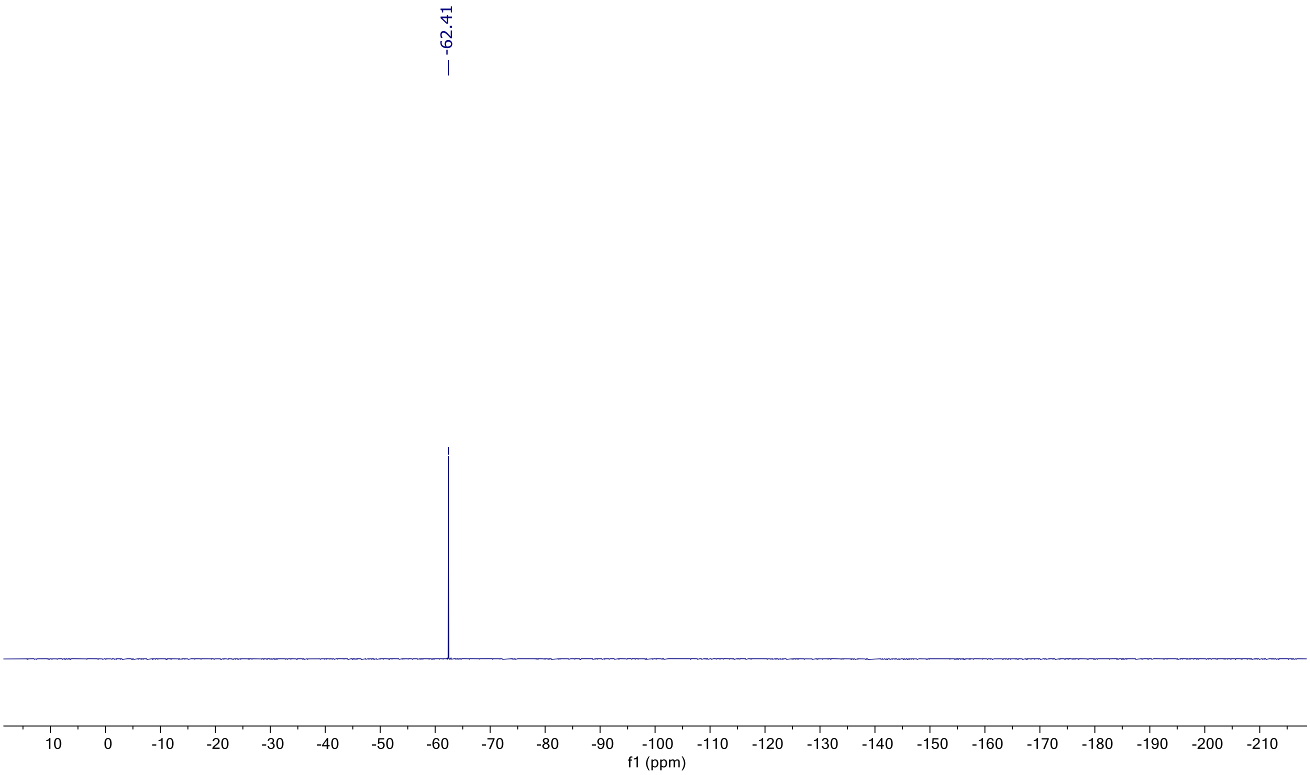

1ac – ^{1}H-NMR (400 MHz, $CDCl_3$)

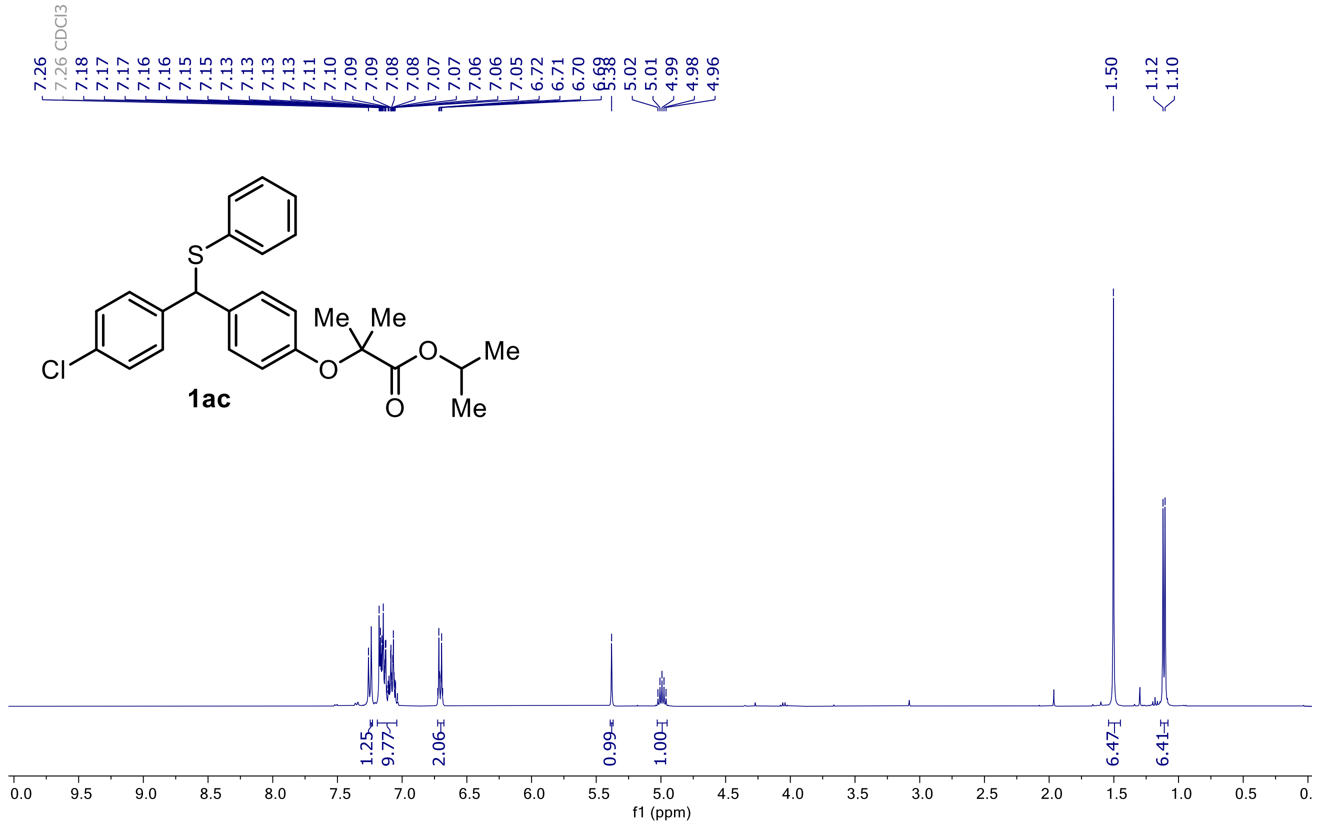

1ac – ^{13}C-NMR (101 MHz, $CDCl_3$)

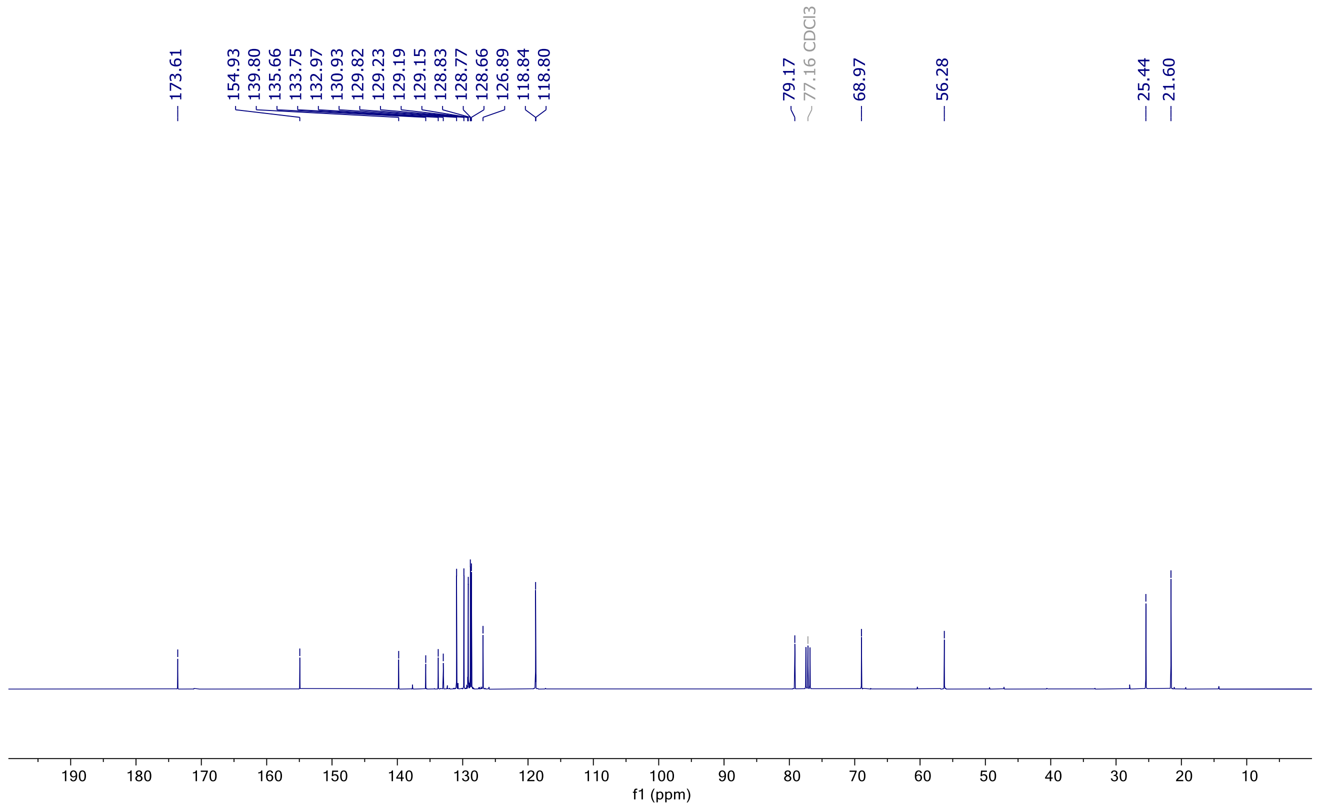

1ad – ^{1}H-NMR (400 MHz, $CDCl_3$) – mixture of diastereoisomers

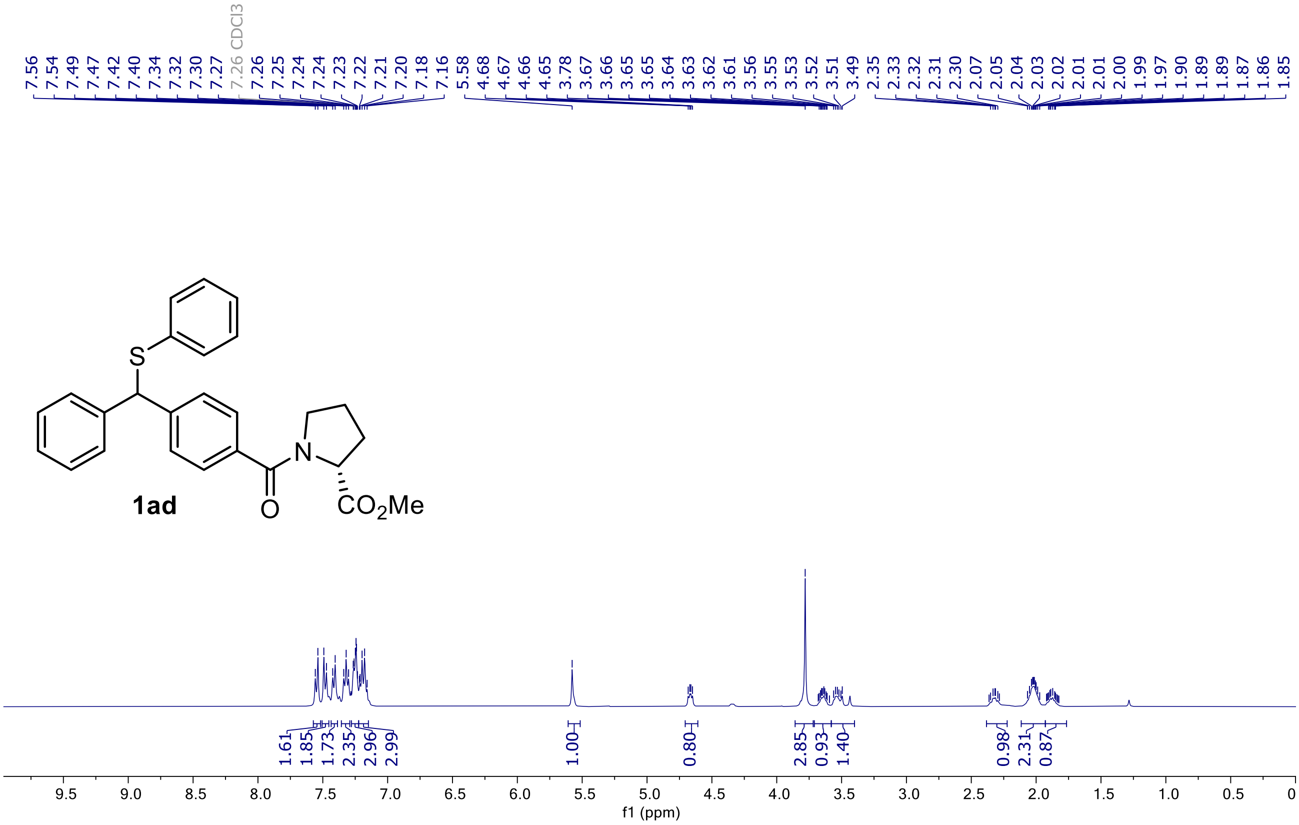

1ad – ^{13}C-NMR (101 MHz, $CDCl_3$) – mixture of diastereoisomers

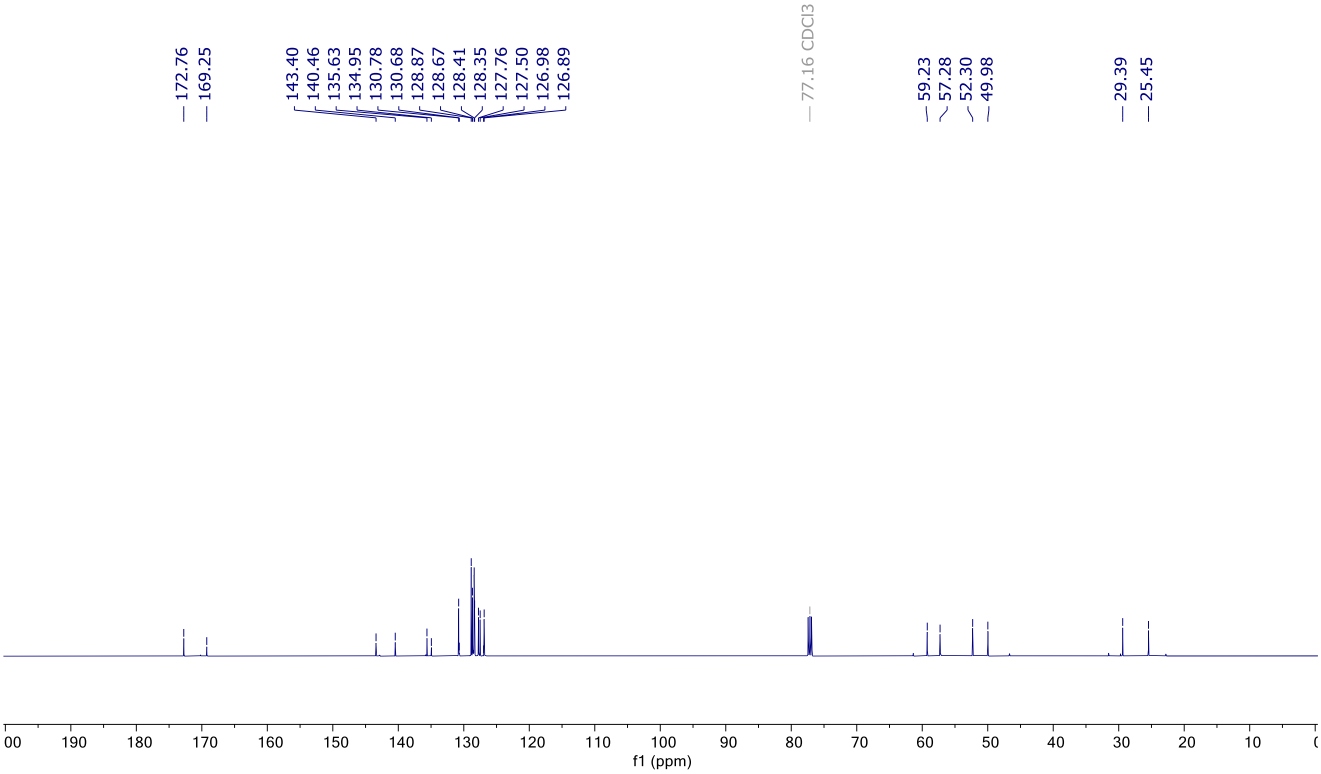

1ae – ^{1}H-NMR (400 MHz, $CDCl_3$) – mixture of diastereoisomers

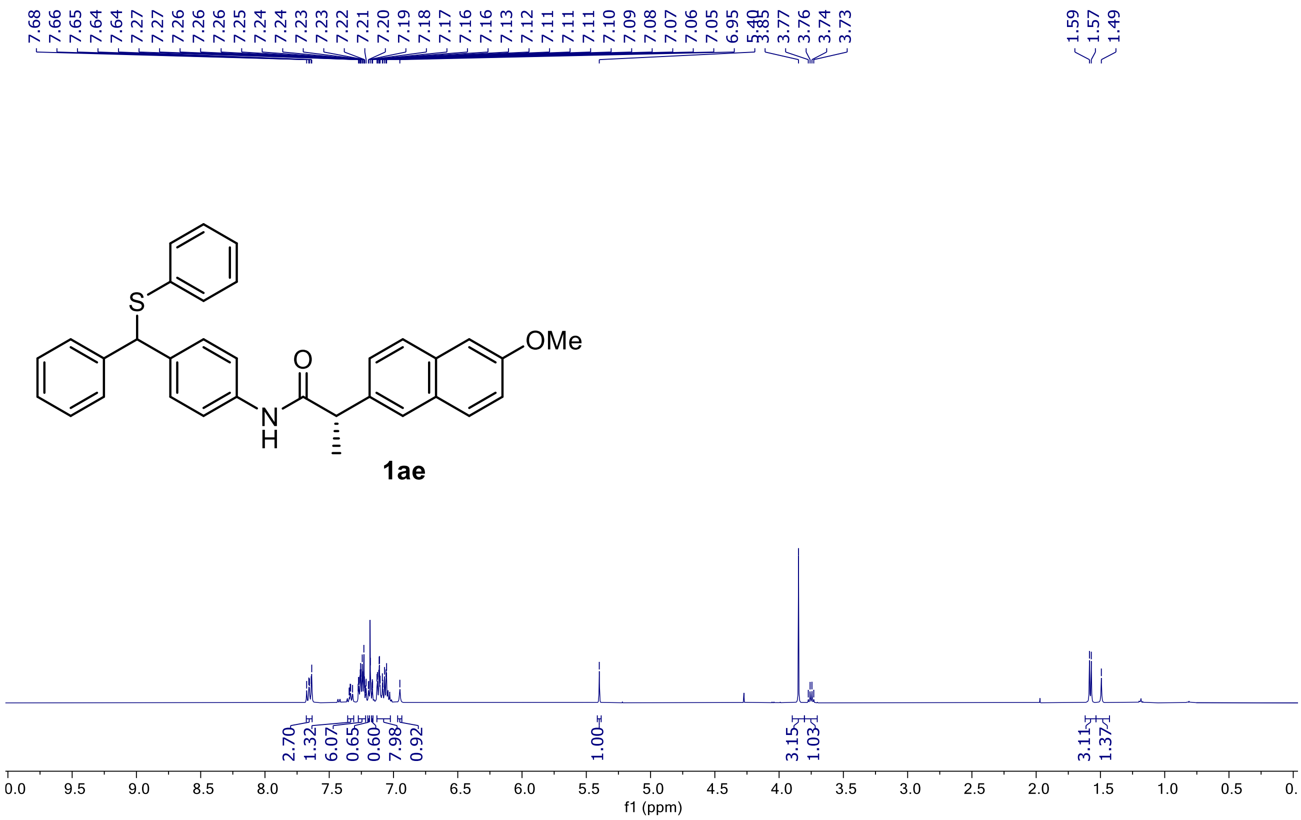

1ae – ^{13}C-NMR (101 MHz, $CDCl_3$) – mixture of diastereoisomers

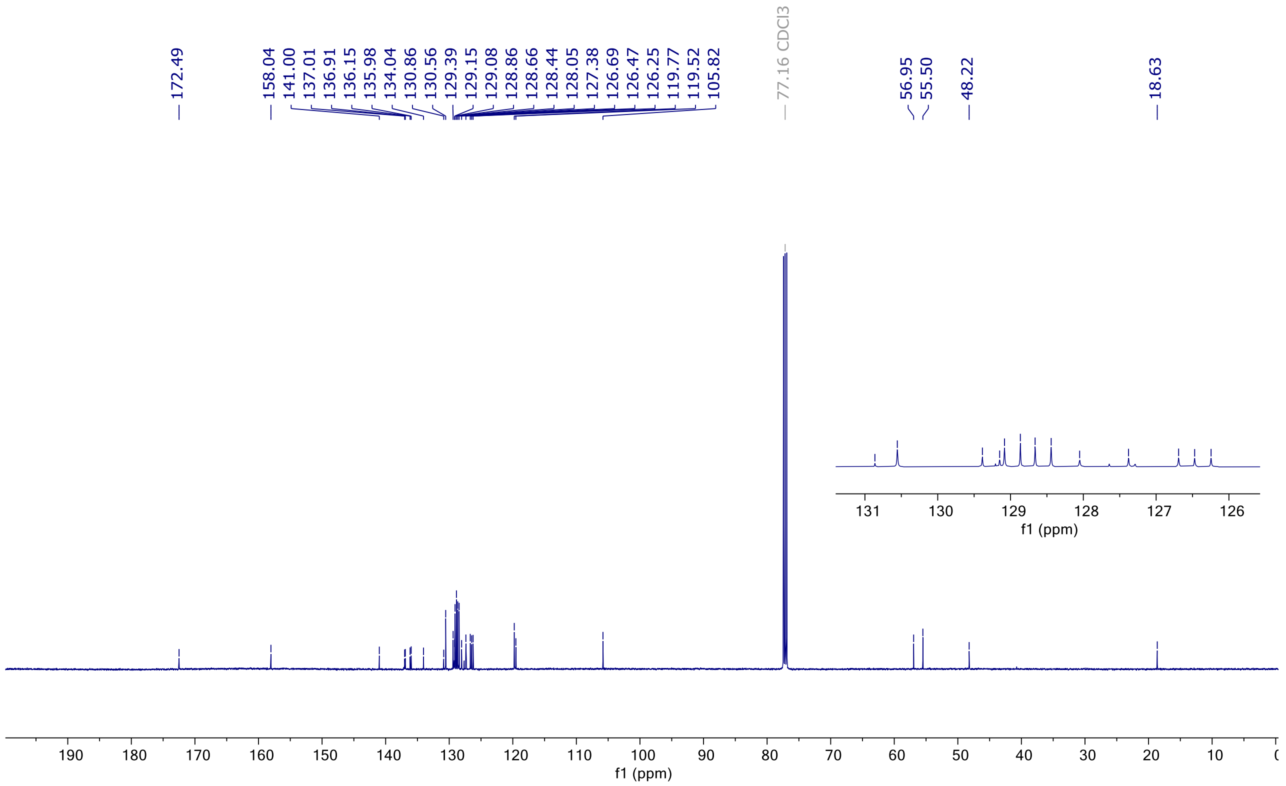

1af – ^{1}H-NMR (400 MHz, $CDCl_3$)

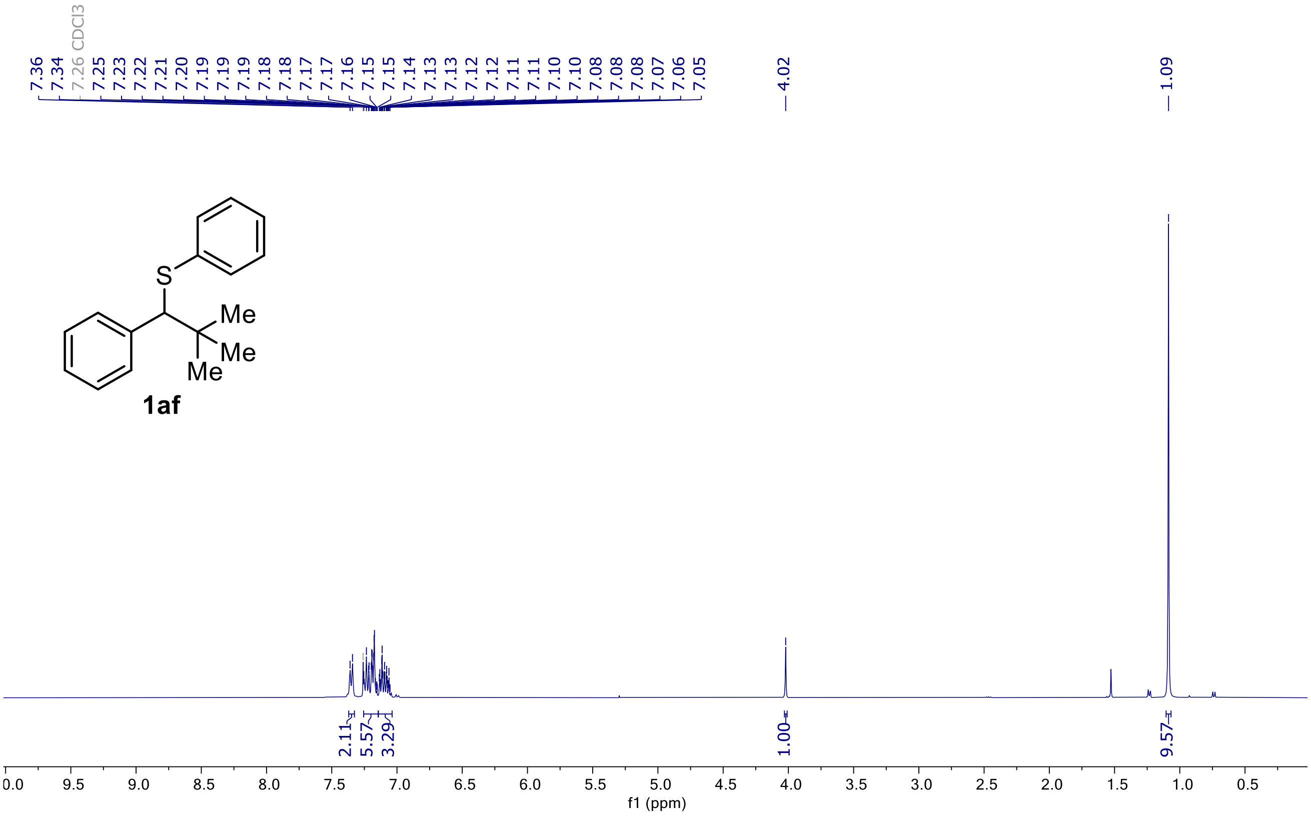

1af – ^{13}C-NMR (101 MHz, $CDCl_3$)

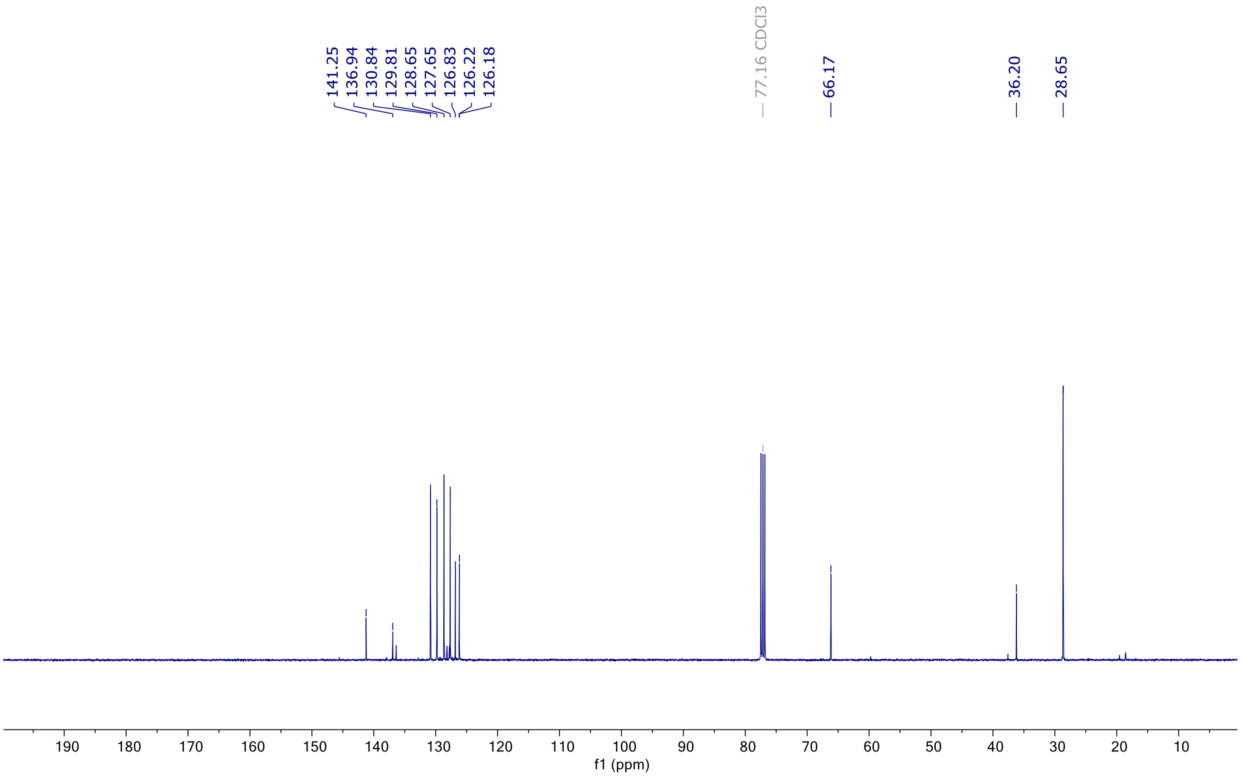

1ag – ^{1}H-NMR (400 MHz, $CDCl_3$) – mixture of diastereoisomers

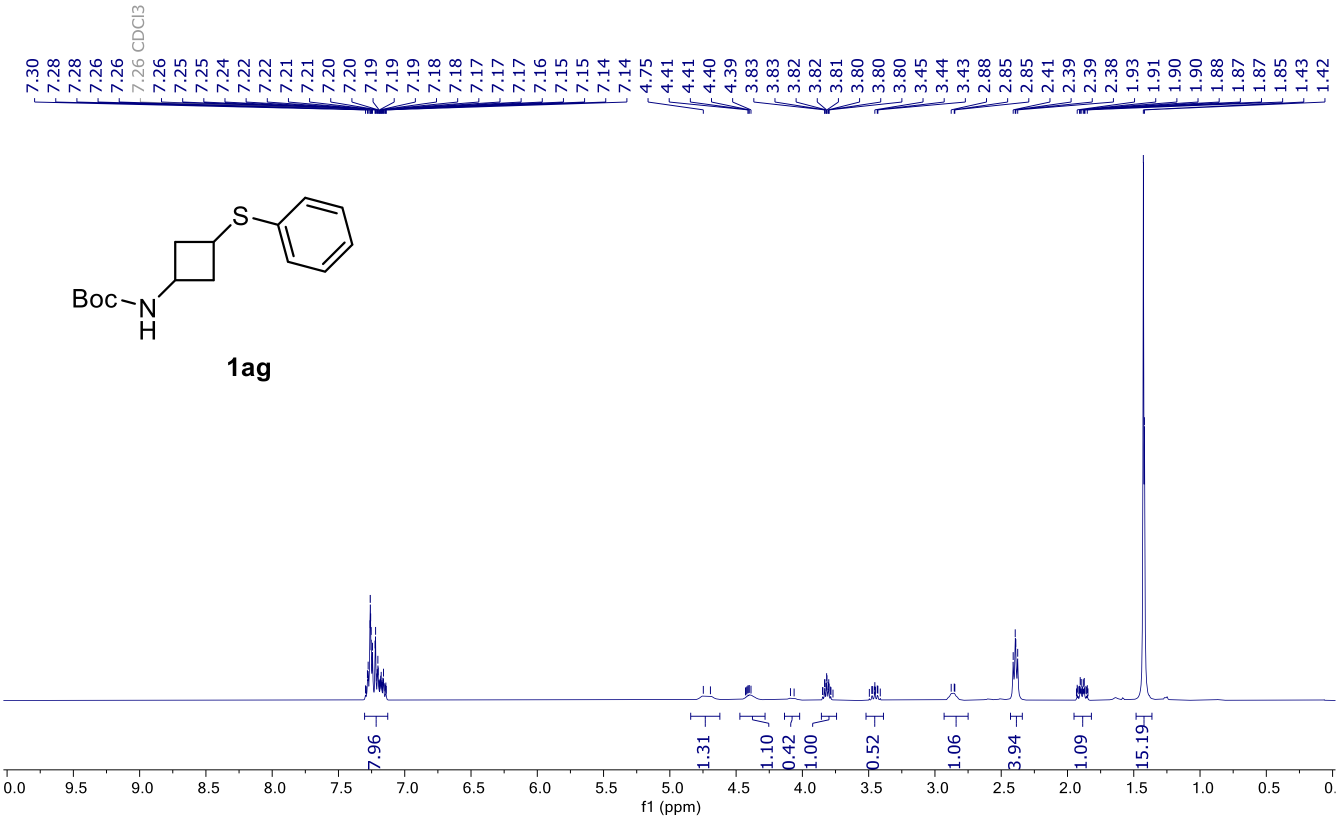

1ag – ^{13}C-NMR (101 MHz, $CDCl_3$) – mixture of diastereoisomers

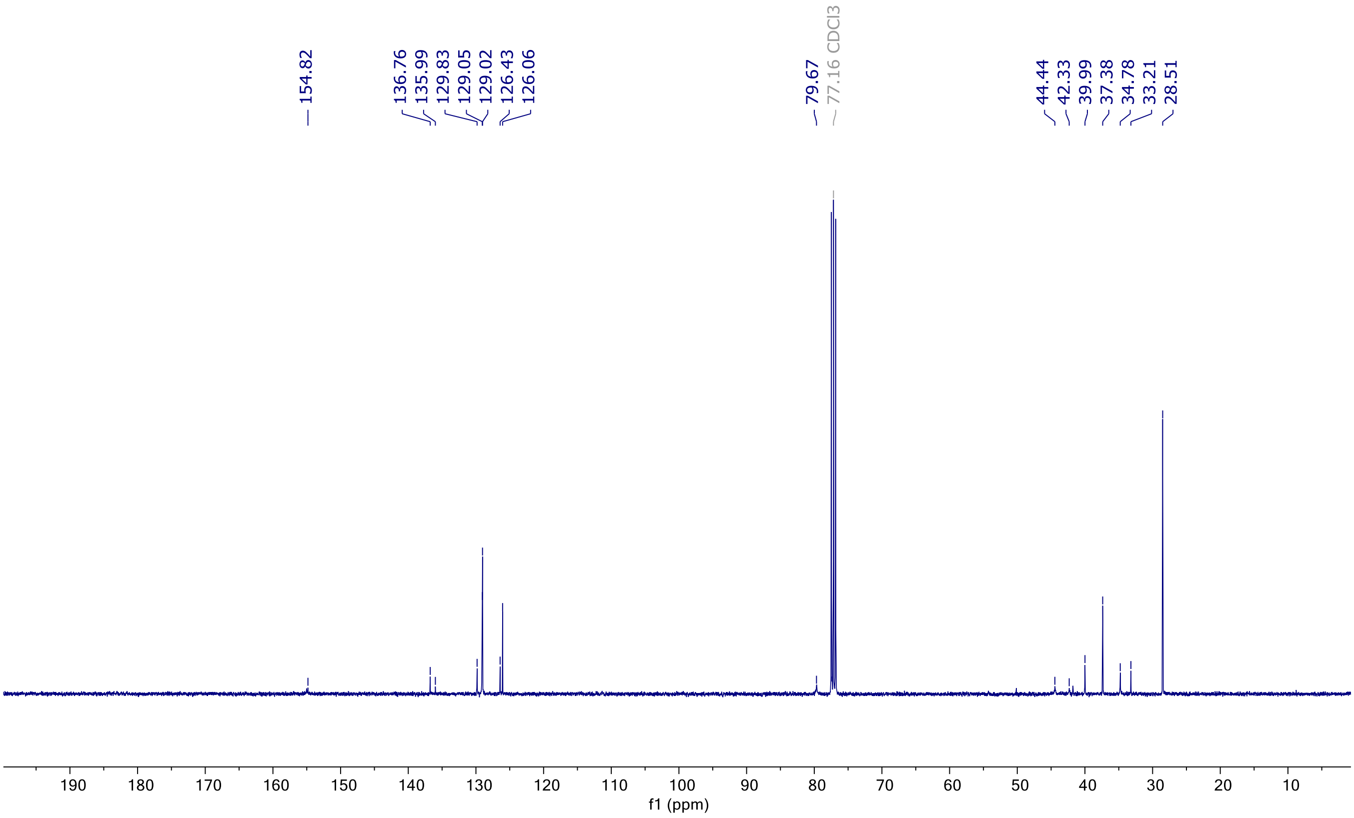

1ah – ^{1}H-NMR (400 MHz, CDCl$_3$) – single diastereoisomer

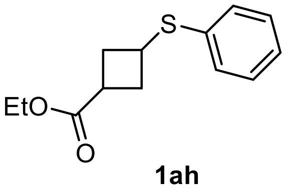

1ah

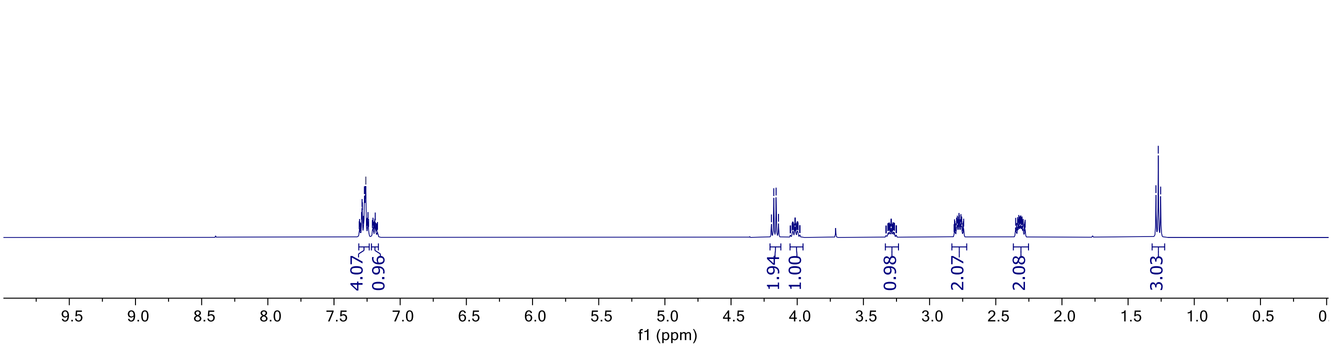

1ah – ^{13}C-NMR (101 MHz, CDCl$_3$) – single diastereoisomer

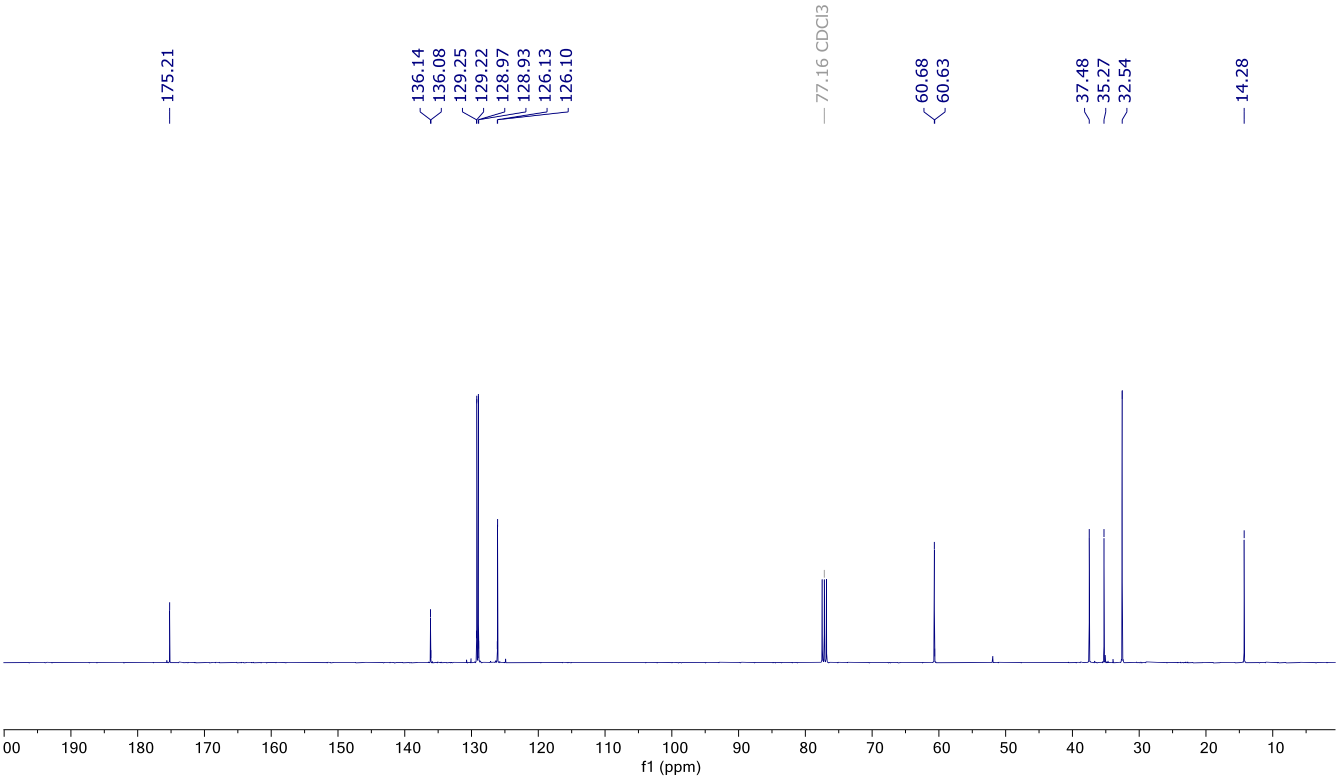

1ai – ^{1}H-NMR (400 MHz, CDCl$_3$)

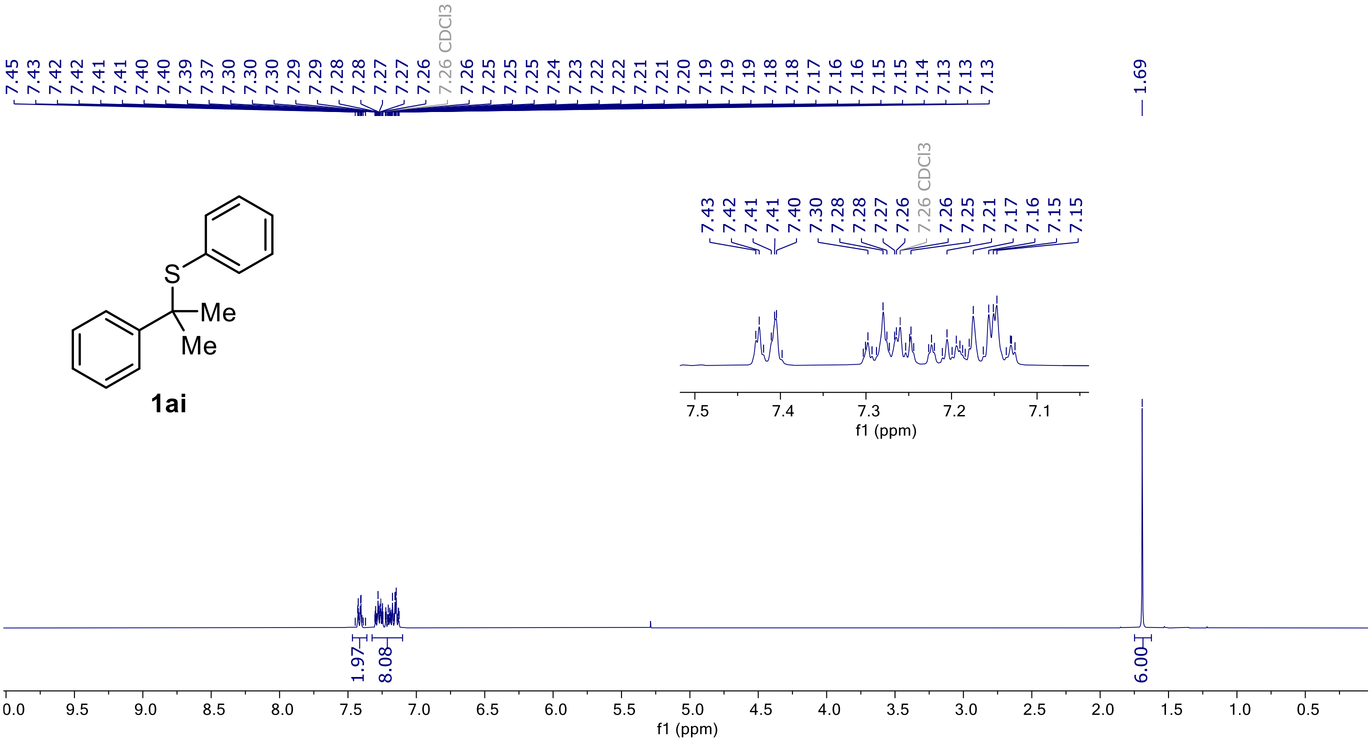

1ai – ^{13}C-NMR (101 MHz, CDCl$_3$)

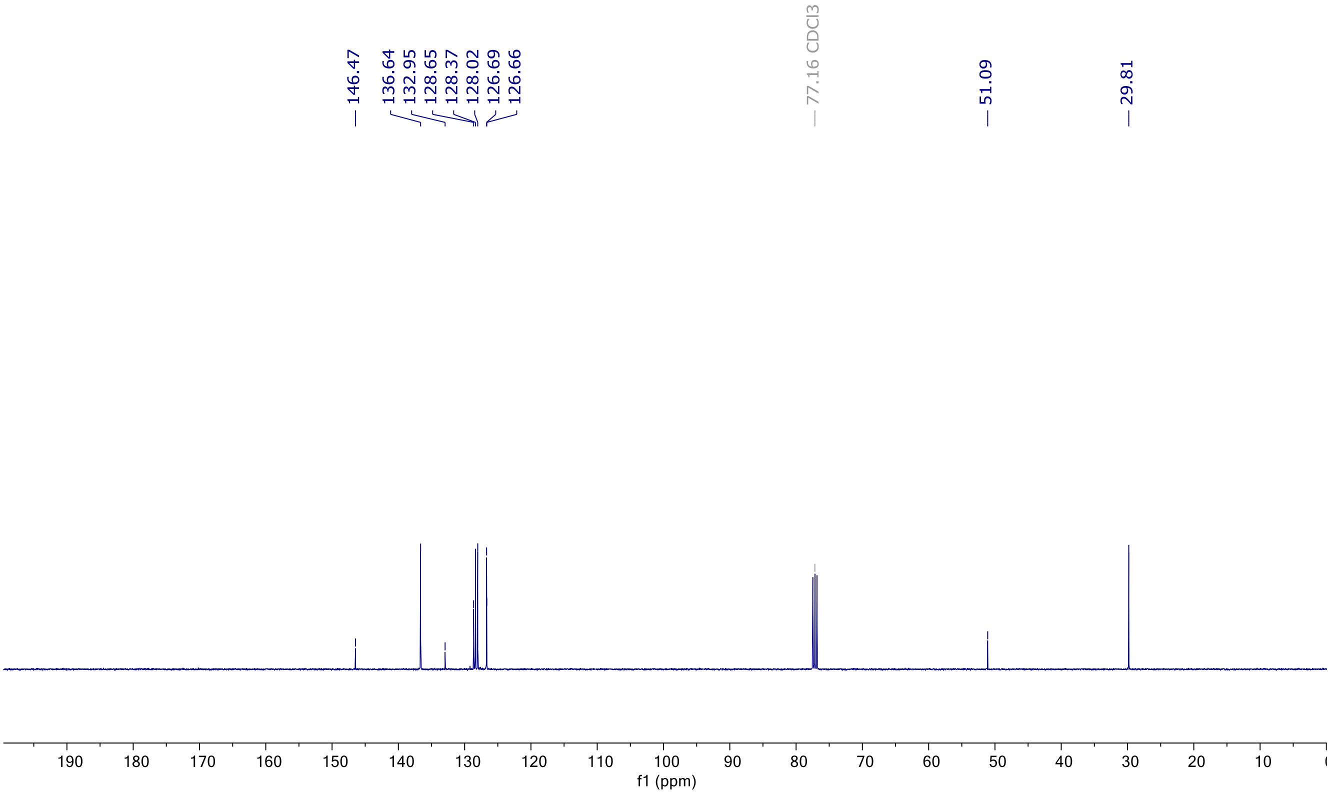

7.2 NMR Spectra of Aminoallylation Products

3a – ¹H-NMR (500 MHz, $CDCl_3$)

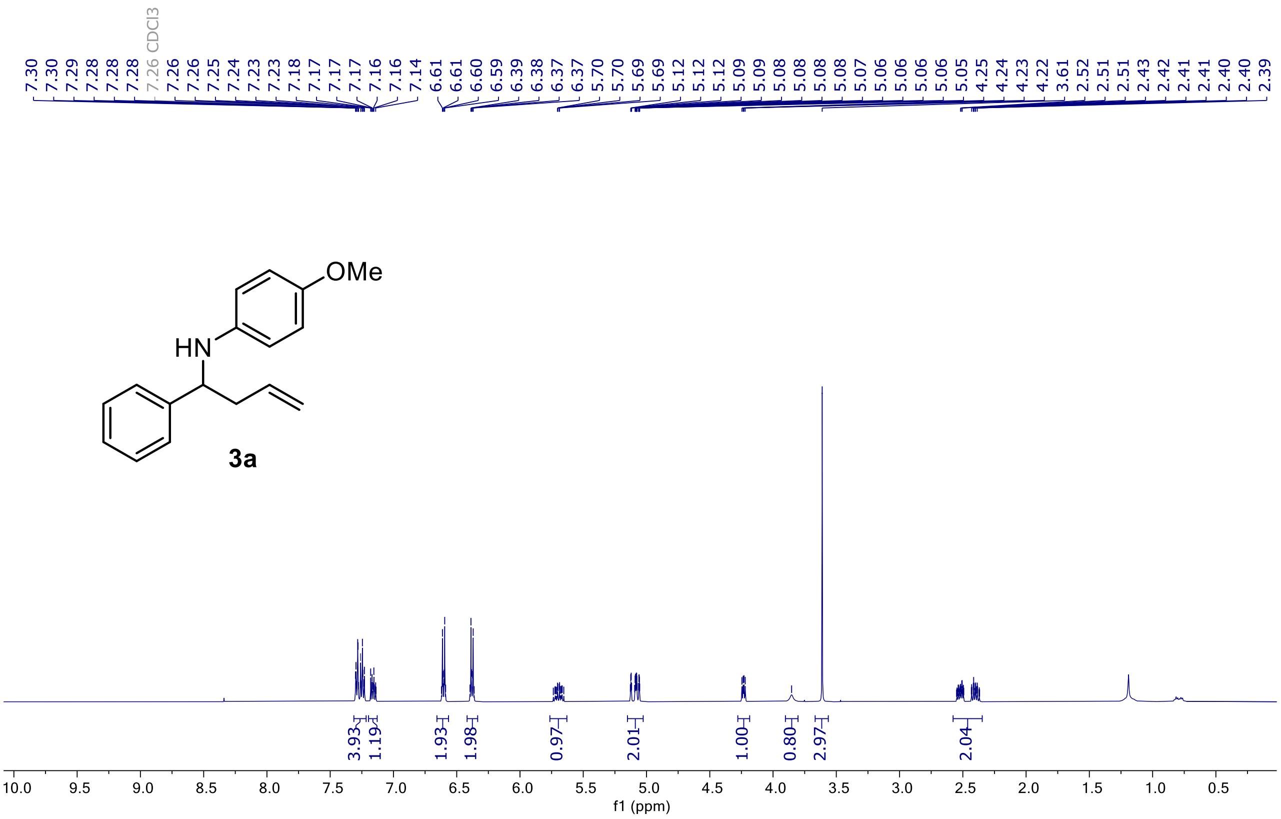

3a – ¹³C-NMR (126 MHz, $CDCl_3$)

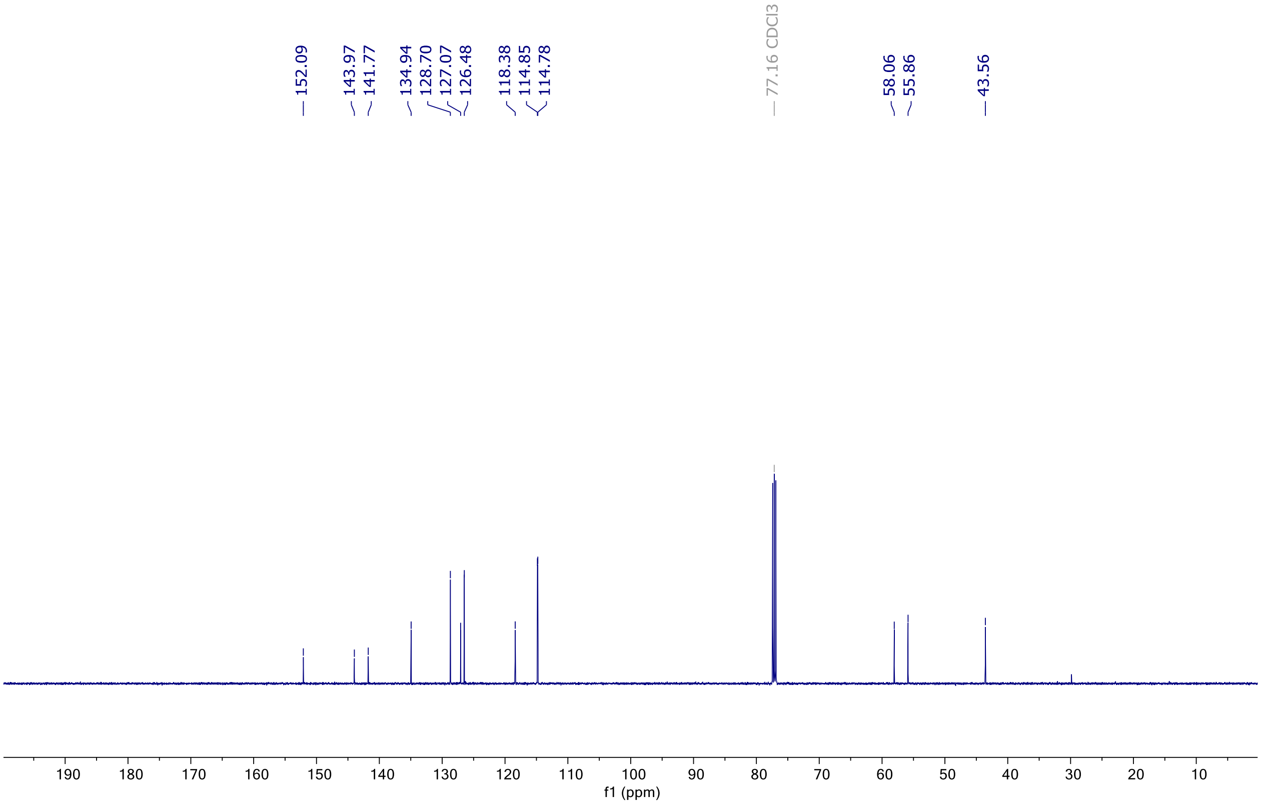

3b – ^{1}H-NMR (500 MHz, $CDCl_3$)

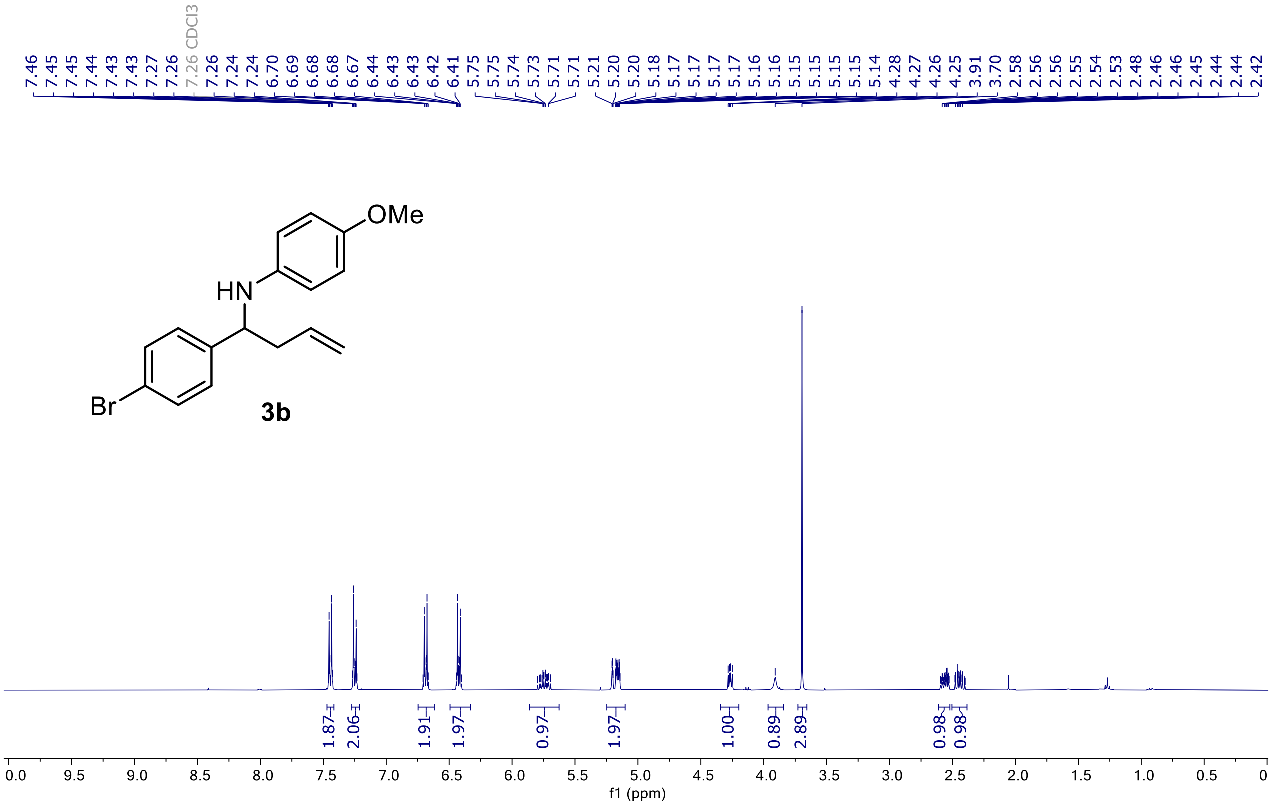

3b – ^{13}C-NMR (126 MHz, $CDCl_3$)

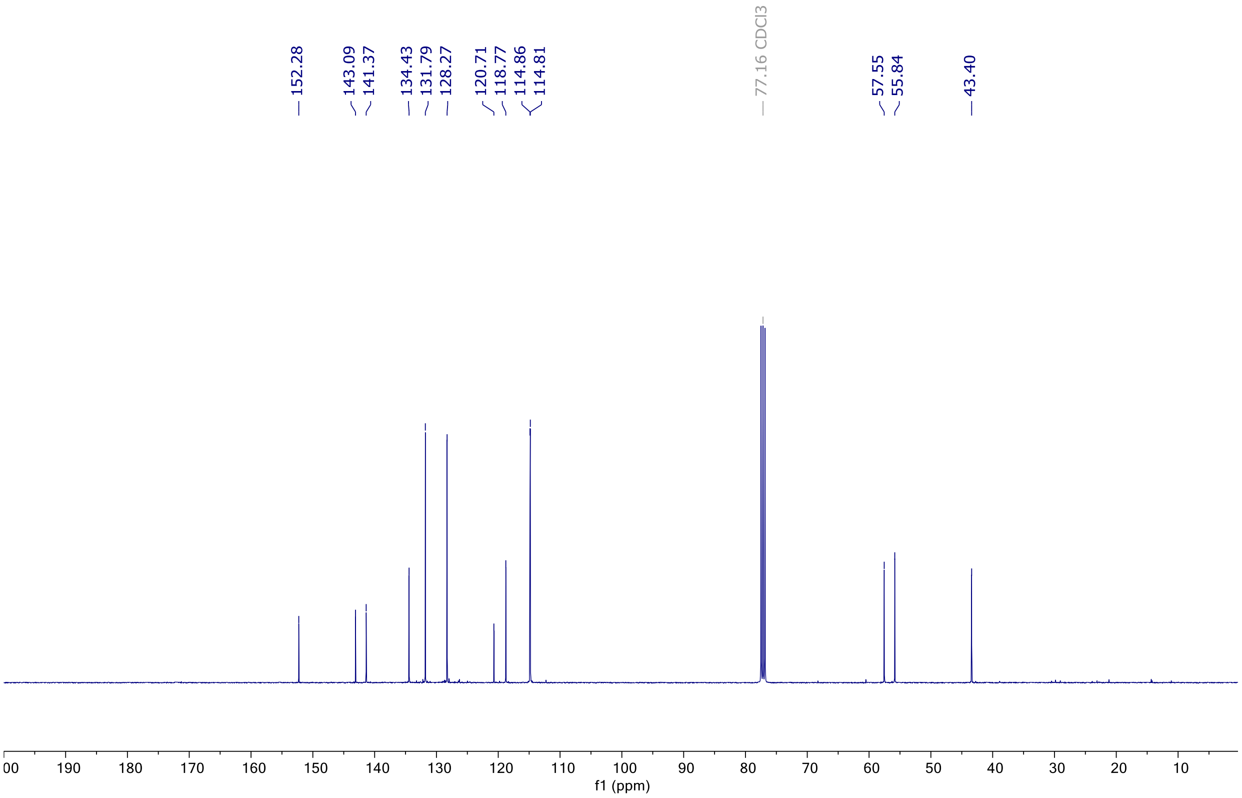

3c – ^{1}H-NMR (400 MHz, $CDCl_3$)

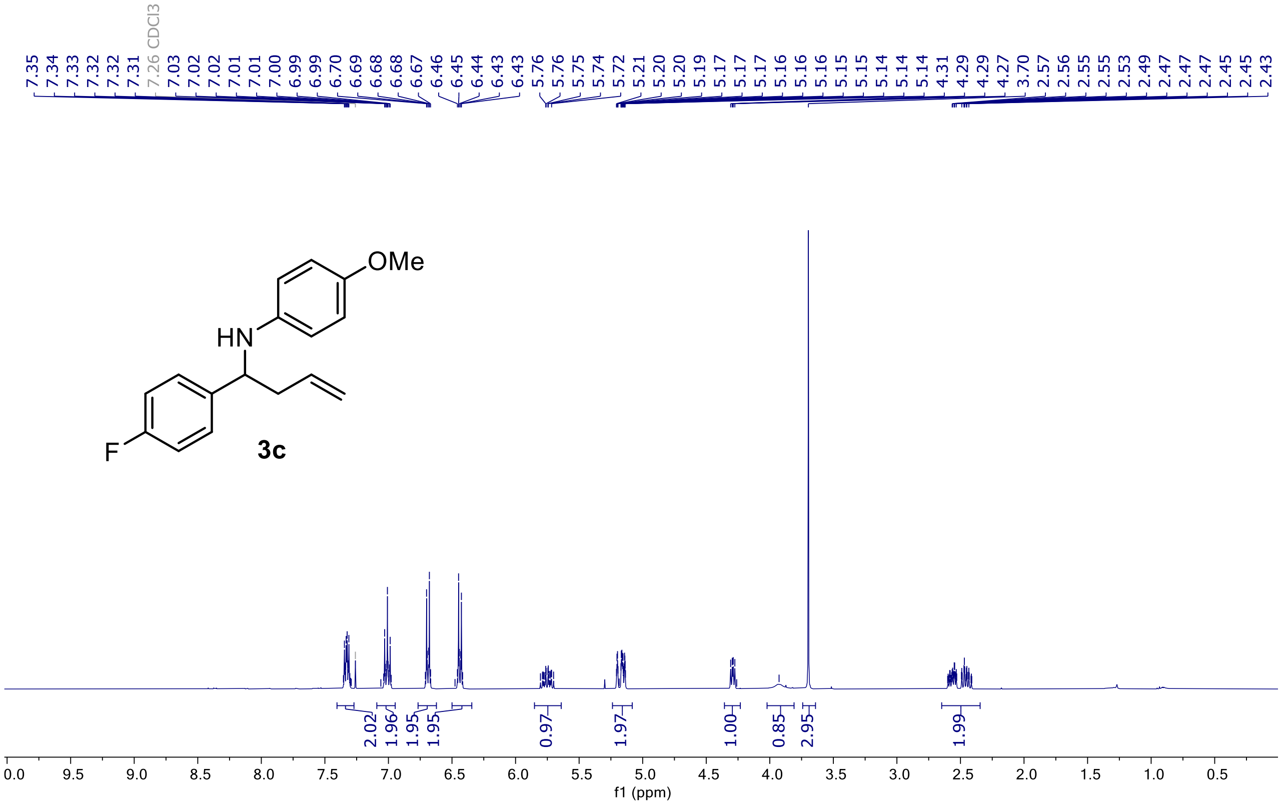

3c – ^{13}C-NMR (101 MHz, $CDCl_3$)

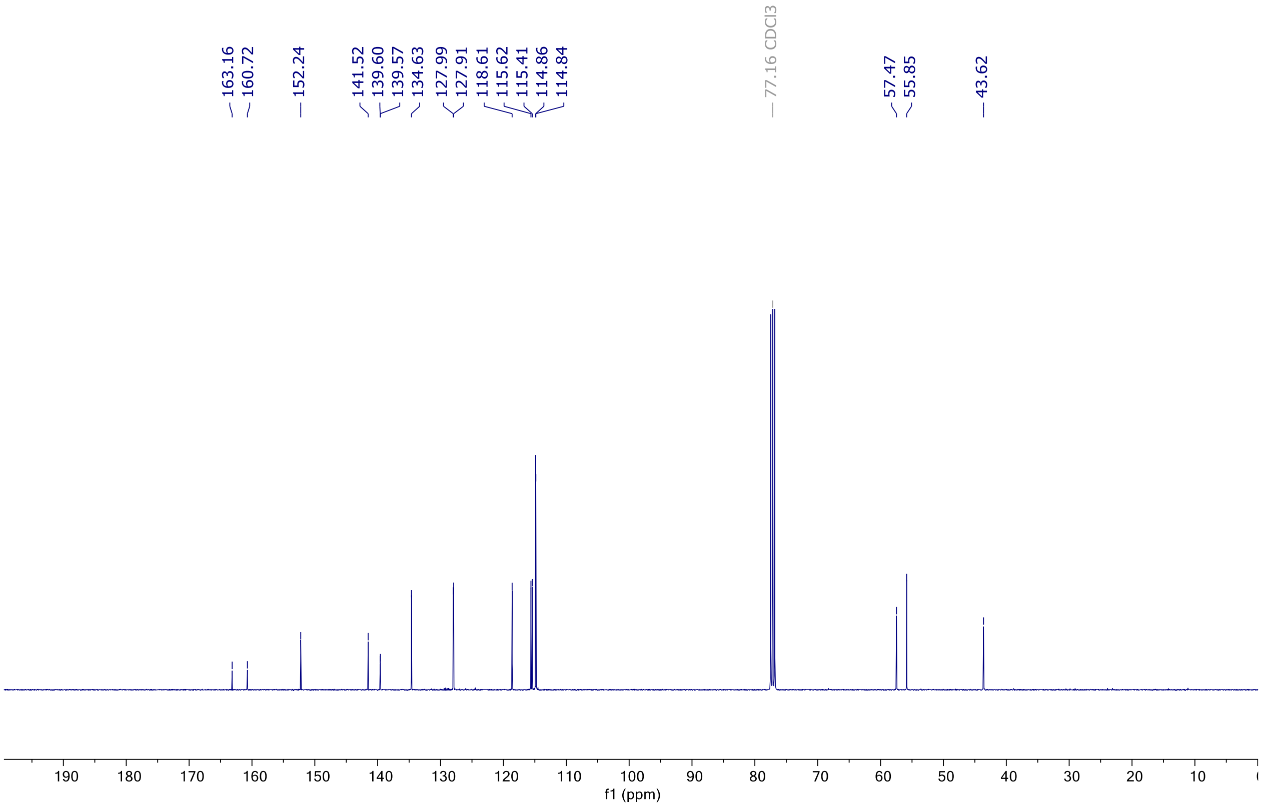

3d – ^{1}H-NMR (500 MHz, $CDCl_3$)

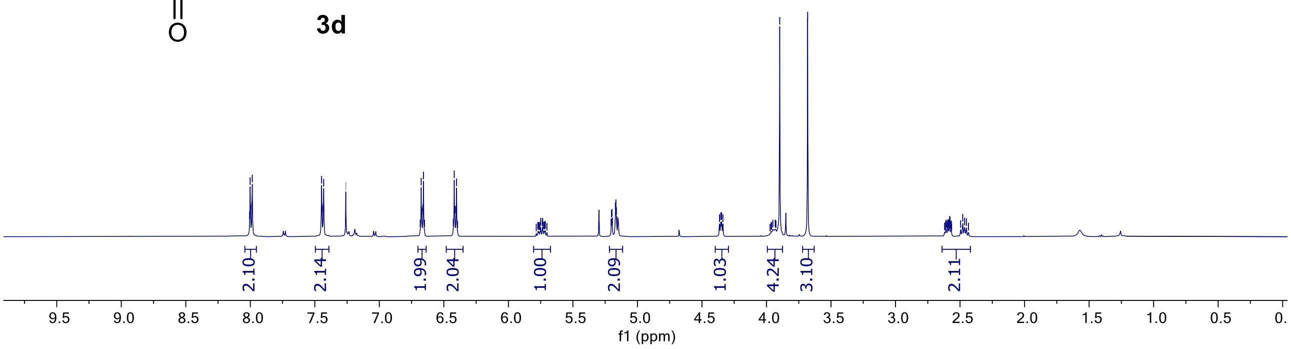

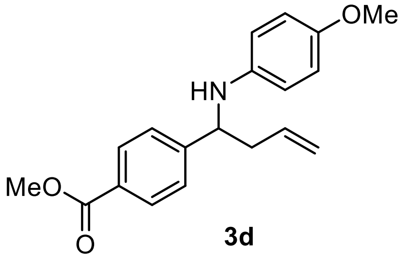

3d

3d – ^{13}C-NMR (126 MHz, $CDCl_3$)

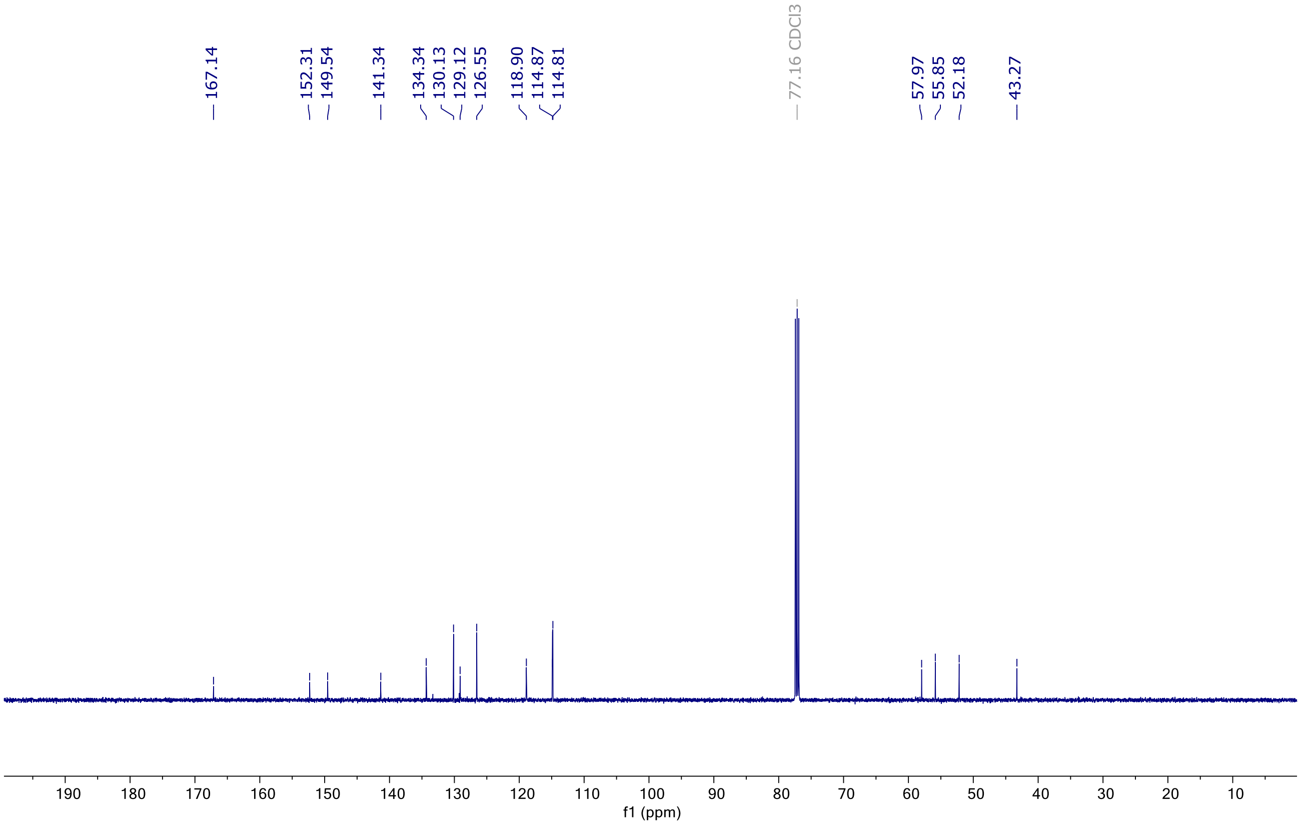

3e – ^{1}H-NMR (400 MHz, $CDCl_3$)

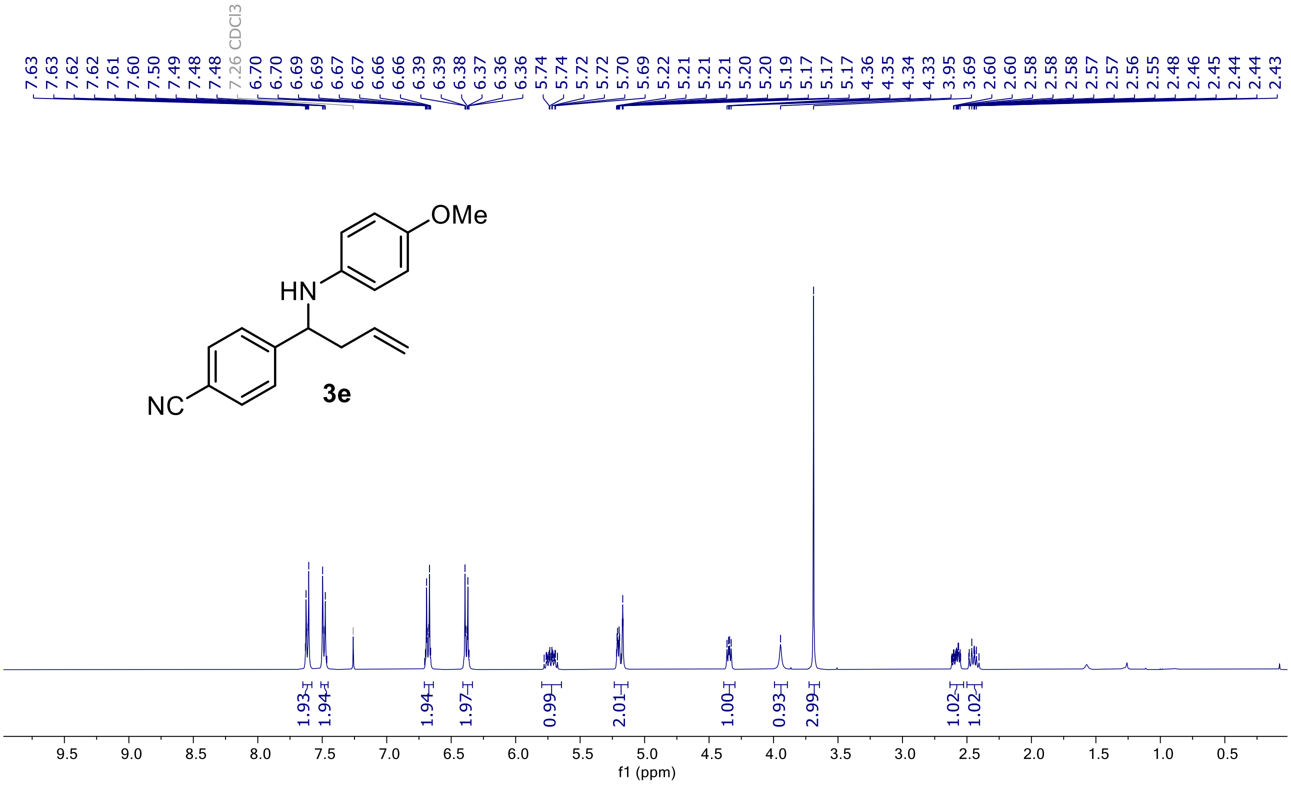

3e – ^{13}C-NMR (101 MHz, $CDCl_3$)

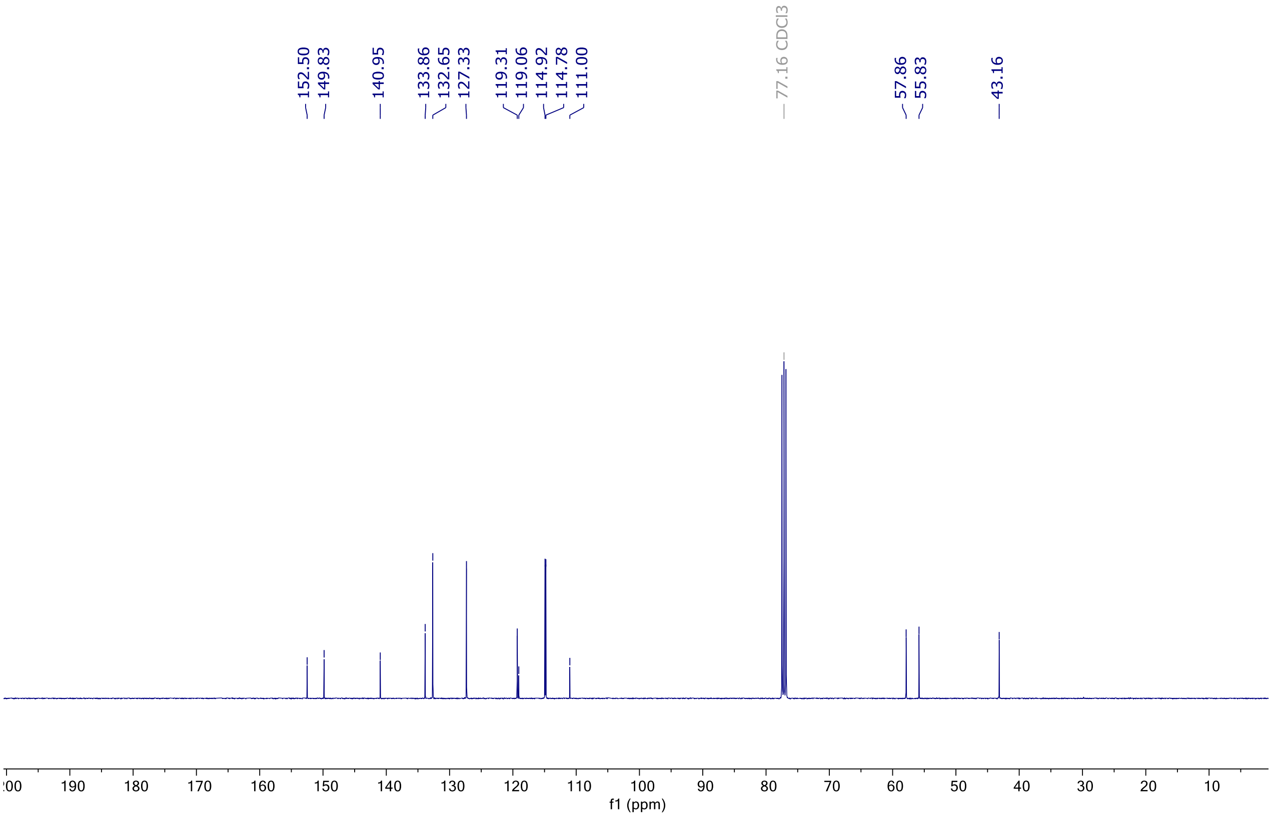

3f – ^{1}H-NMR (400 MHz, $CDCl_3$)

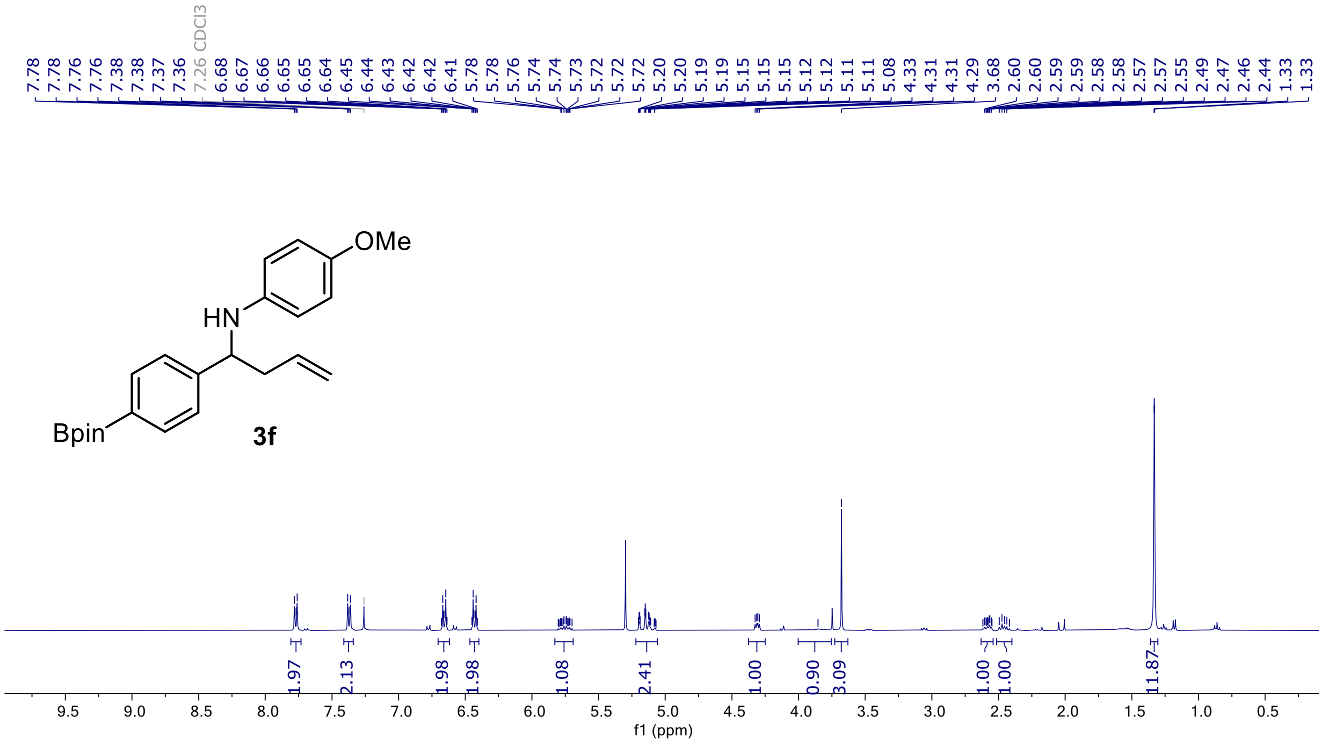

3f – ^{13}C-NMR (101 MHz, $CDCl_3$)

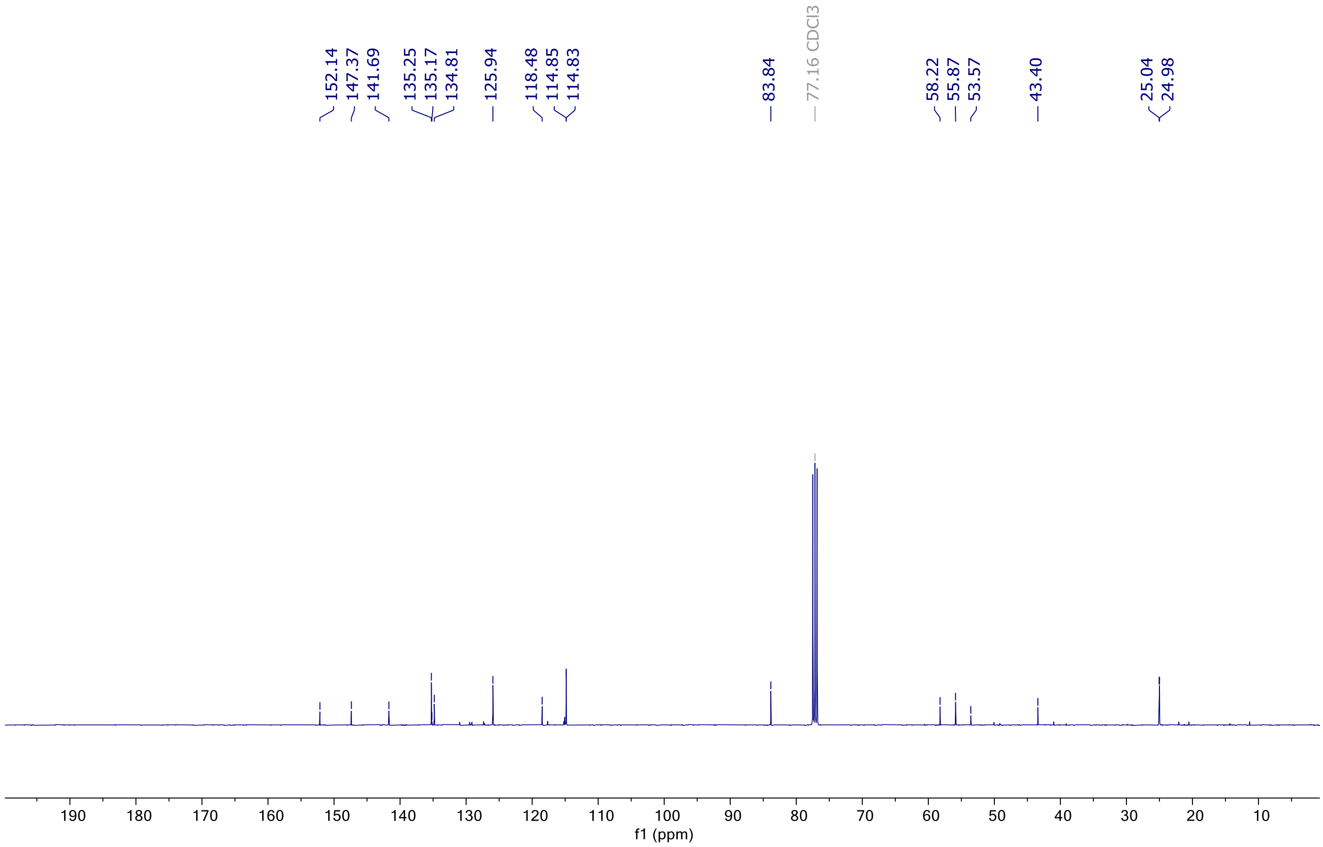

3f – ^{11}B-NMR (128 MHz, $CDCl_3$)

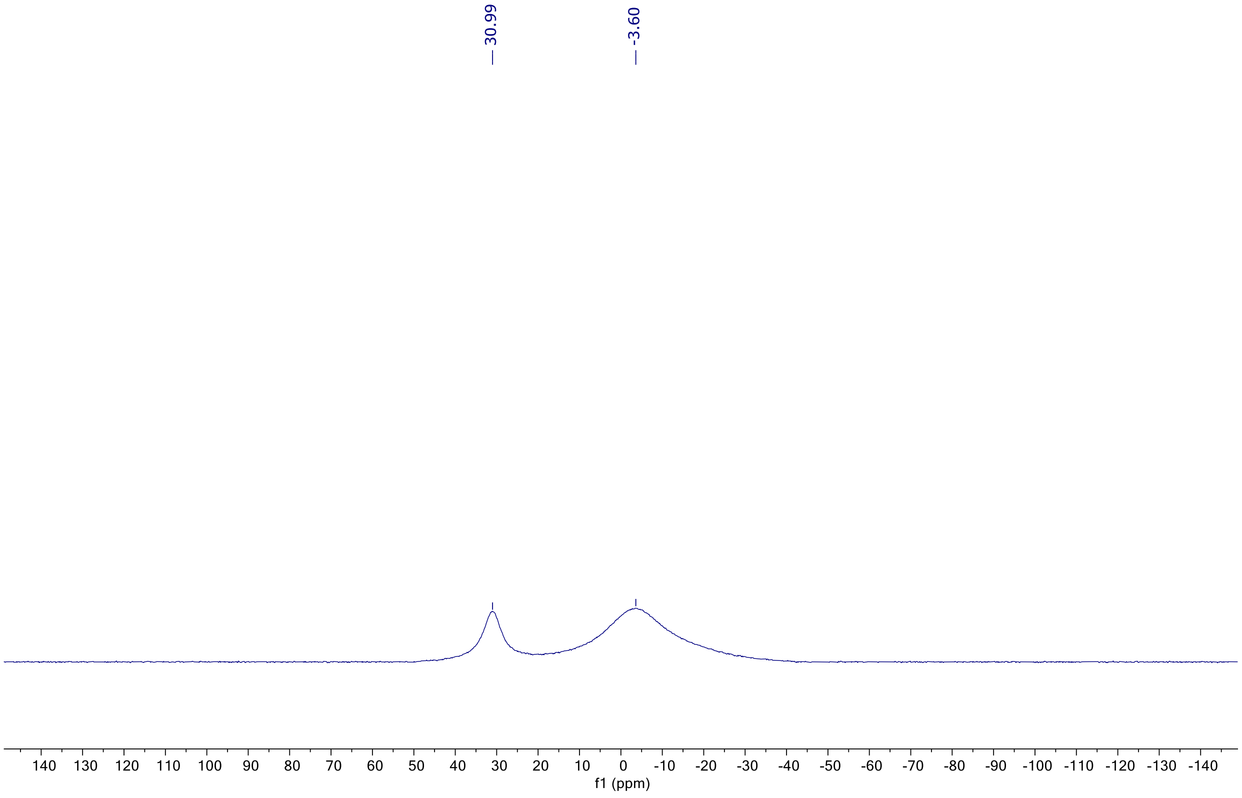

3g – ^{1}H-NMR (400 MHz, $CDCl_3$)

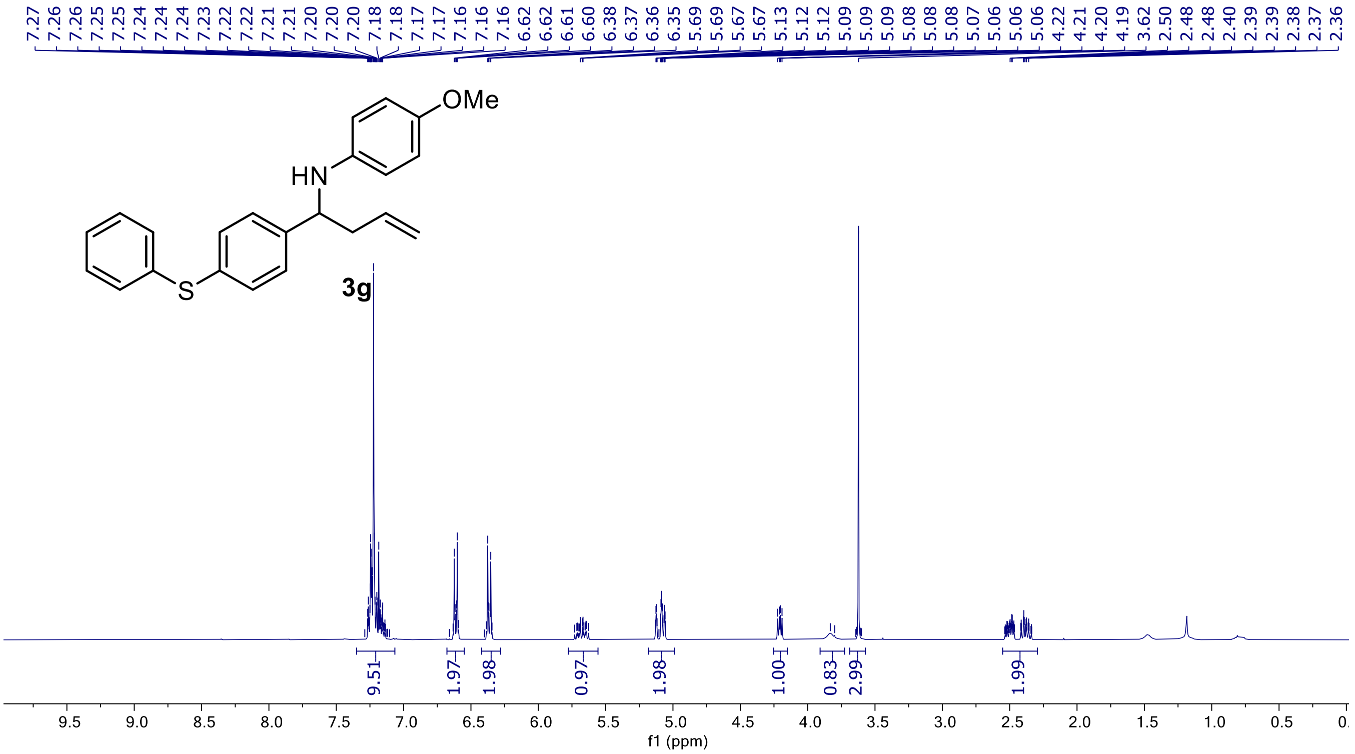

3g – ^{13}C-NMR (101 MHz, $CDCl_3$)

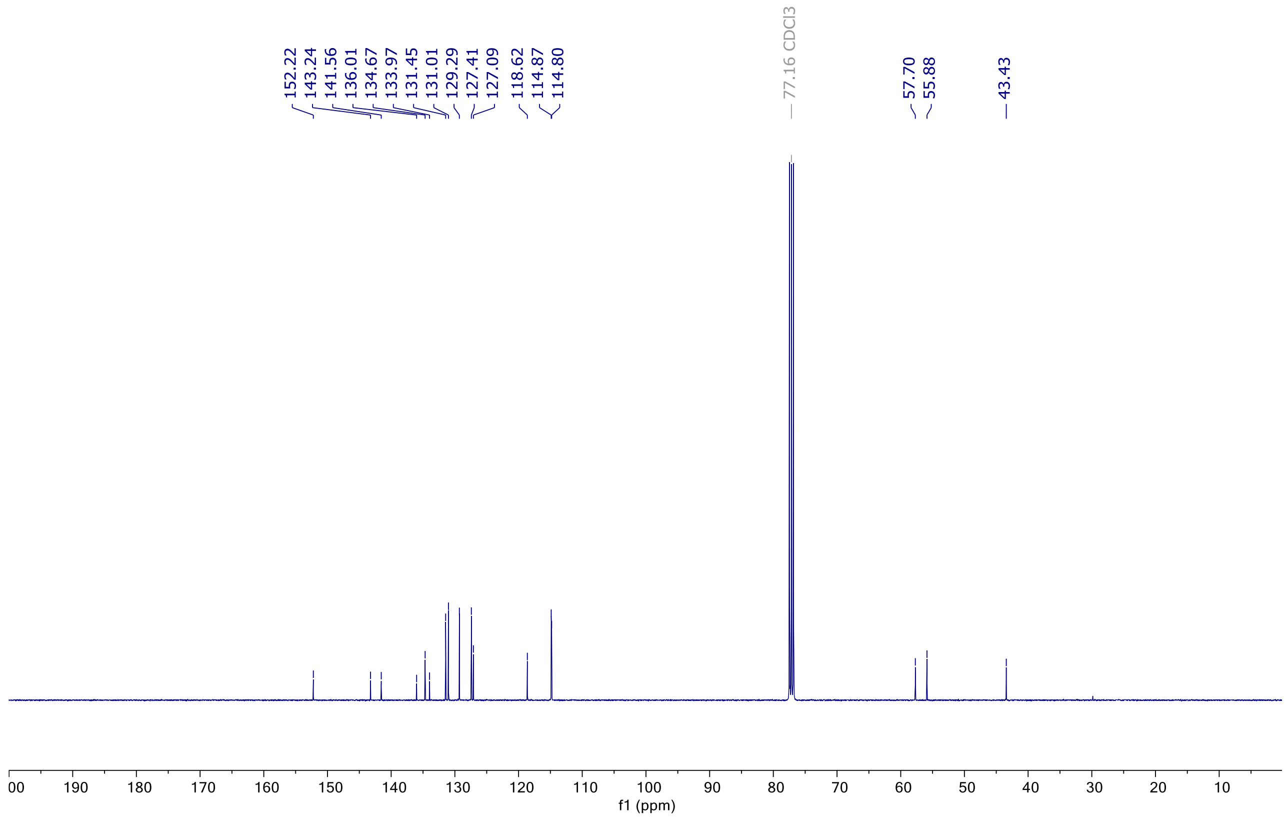

3h – ^{1}H-NMR (400 MHz, $CDCl_3$)

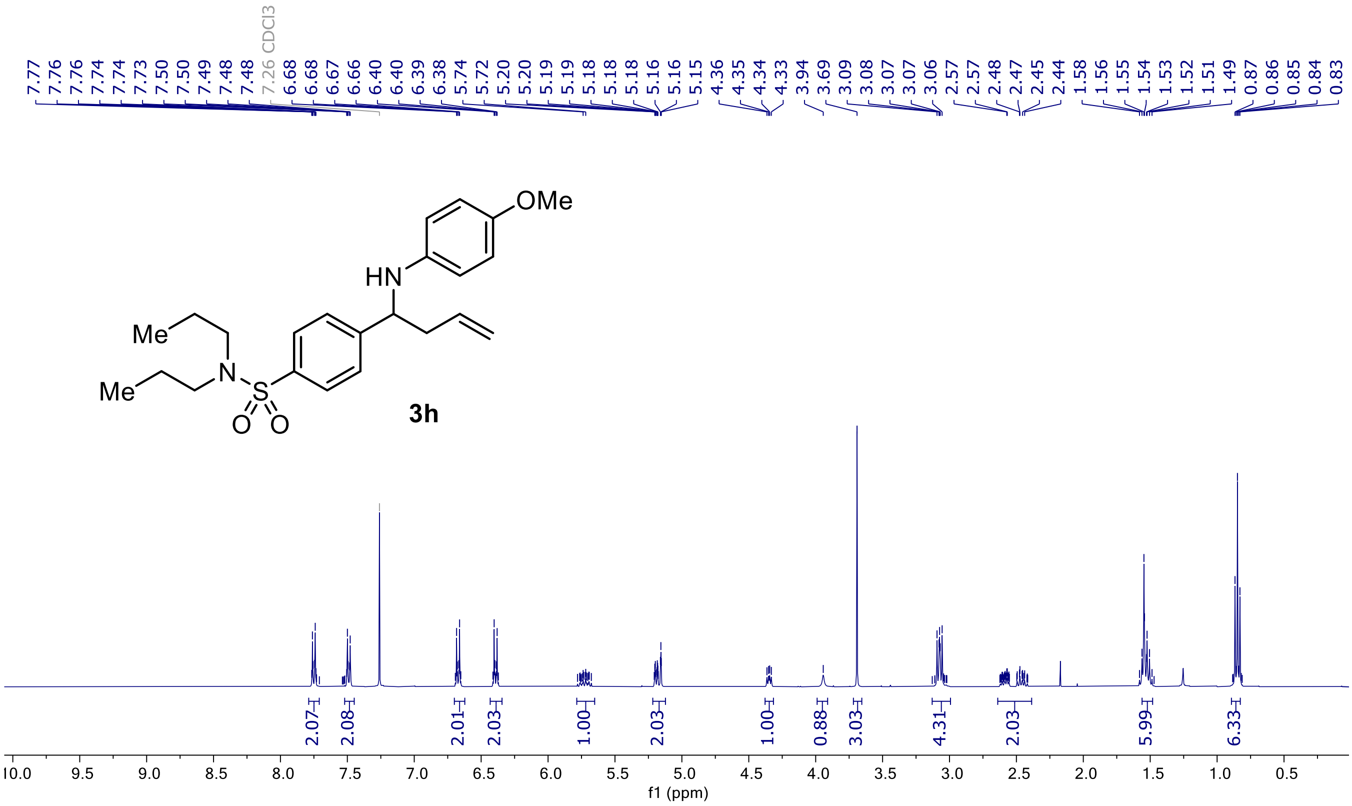

3h – ^{13}C-NMR (101 MHz, $CDCl_3$)

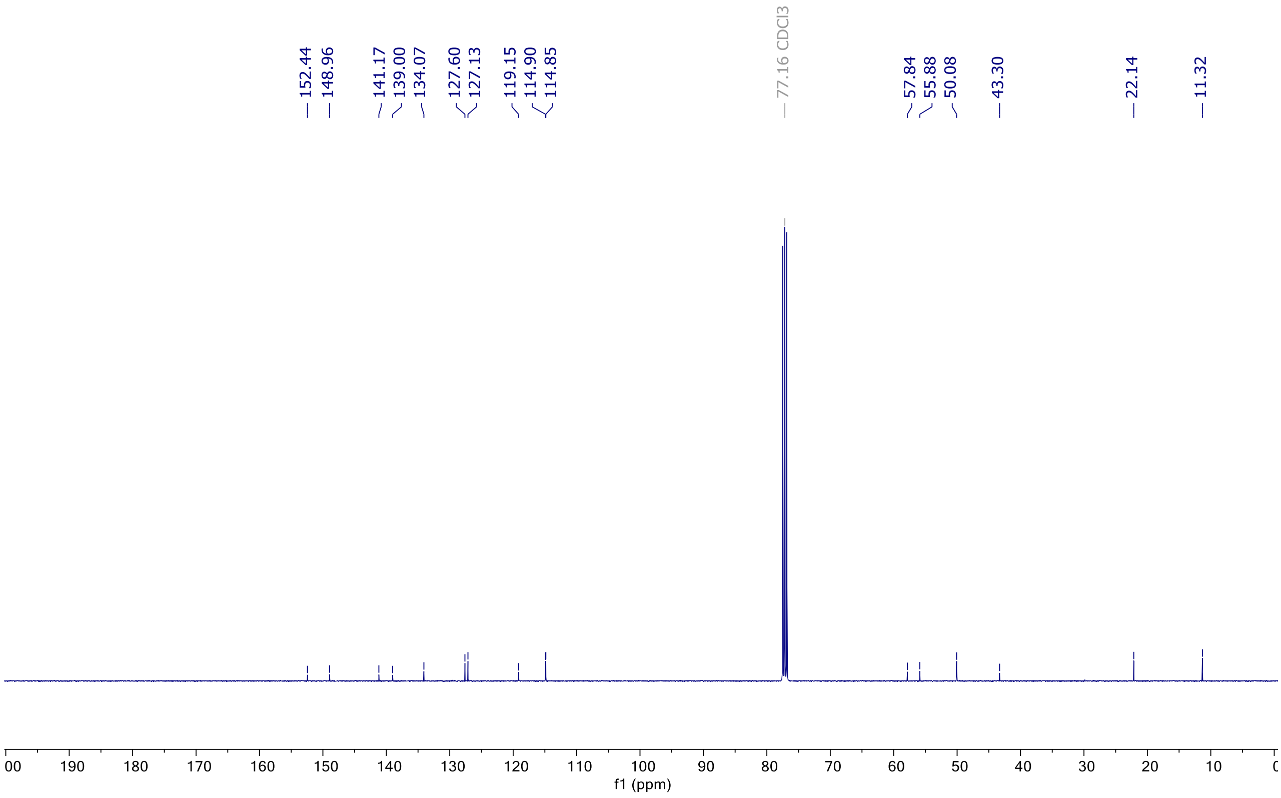

3i – ^{1}H-NMR (500 MHz, $CDCl_3$)

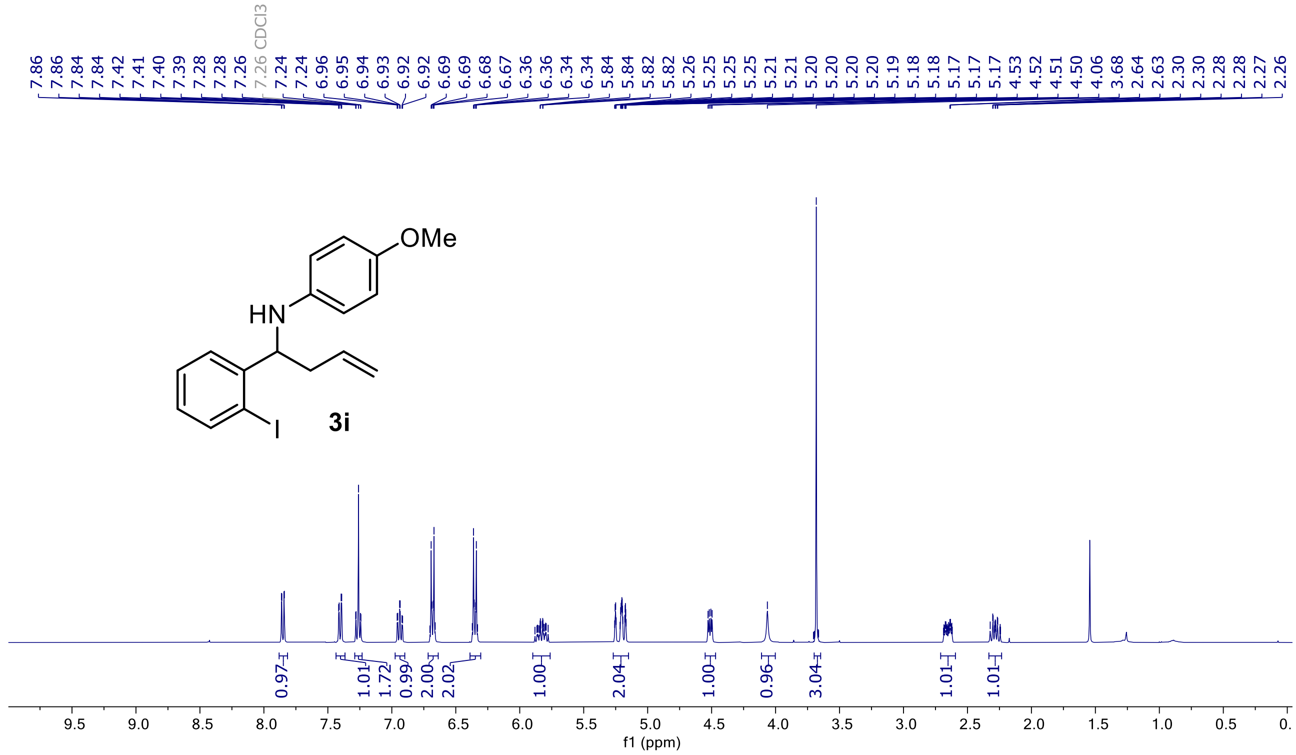

3i – ^{13}C-NMR (126 MHz, $CDCl_3$)

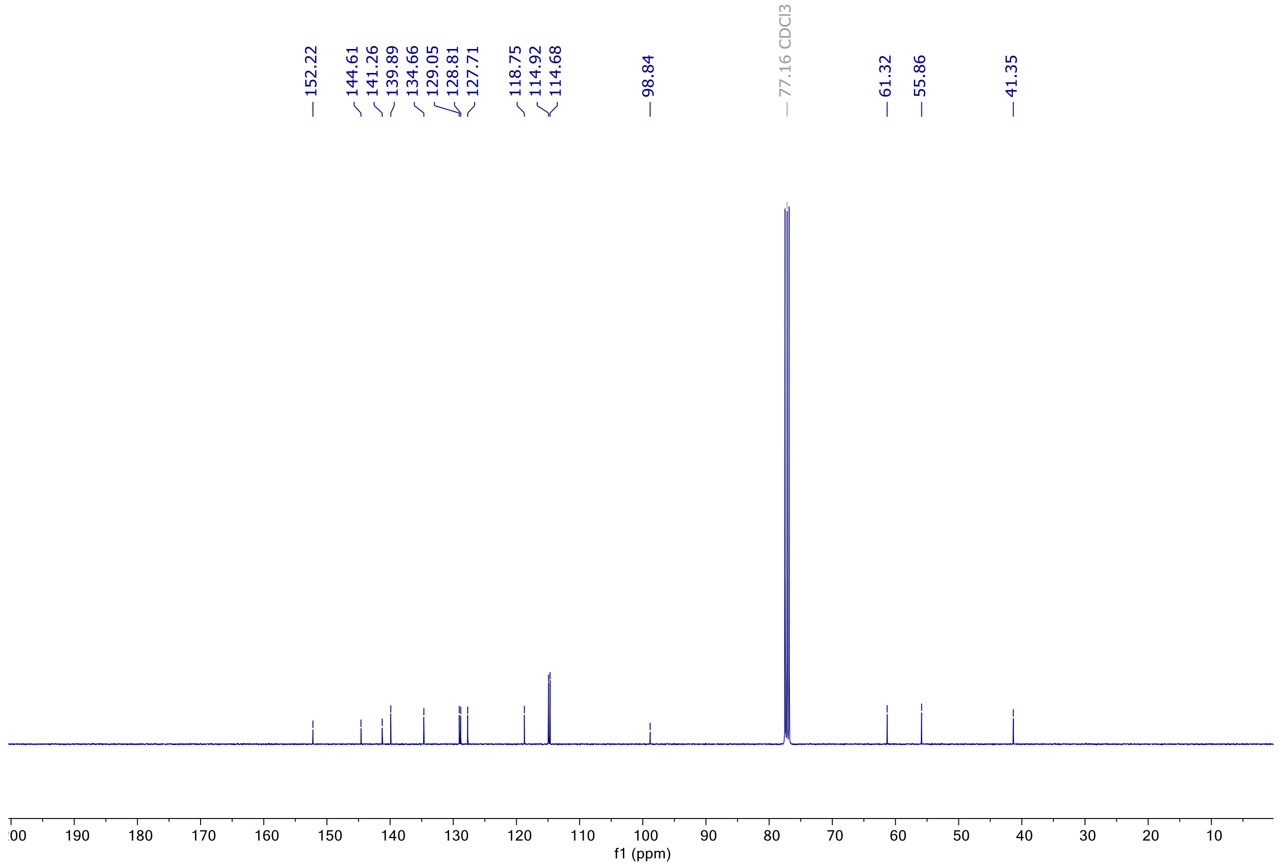

3j – ^{1}H-NMR (400 MHz, $CDCl_3$)

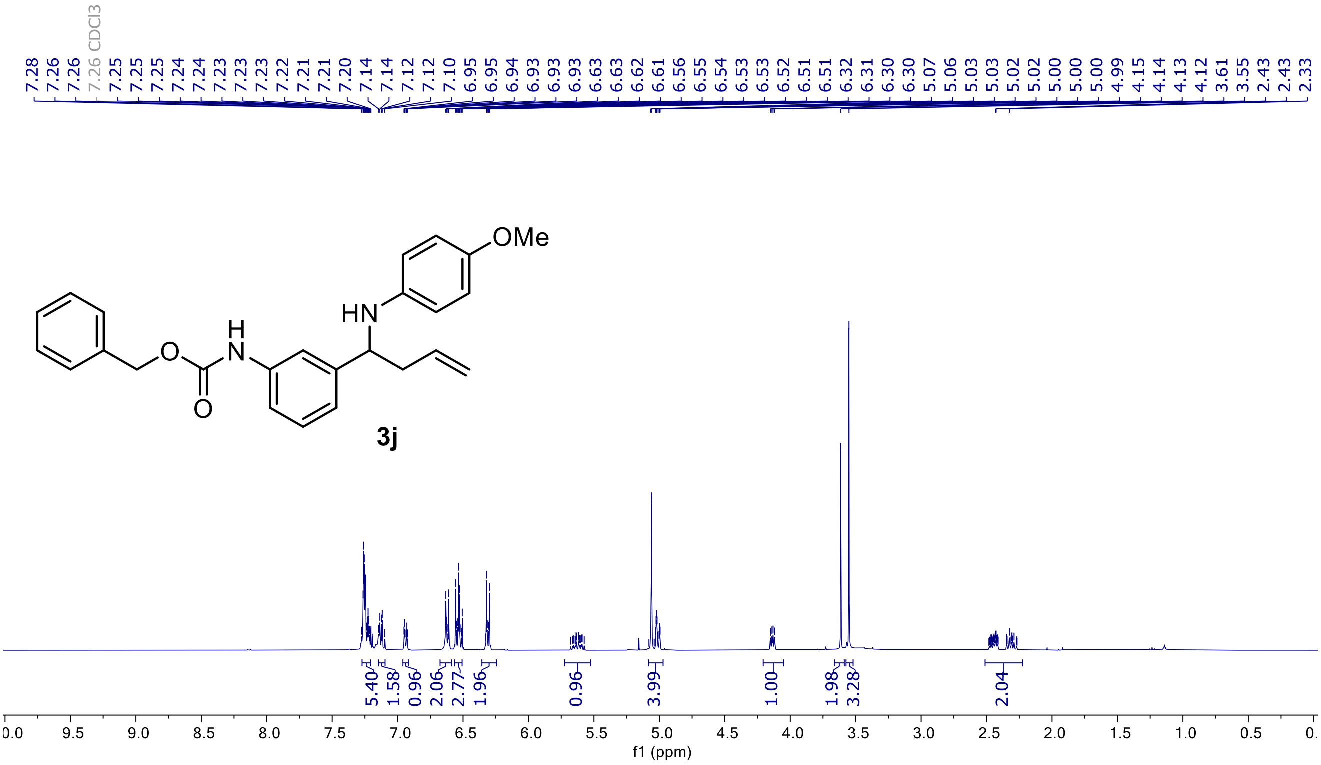

3j – ^{13}C-NMR (101 MHz, $CDCl_3$)

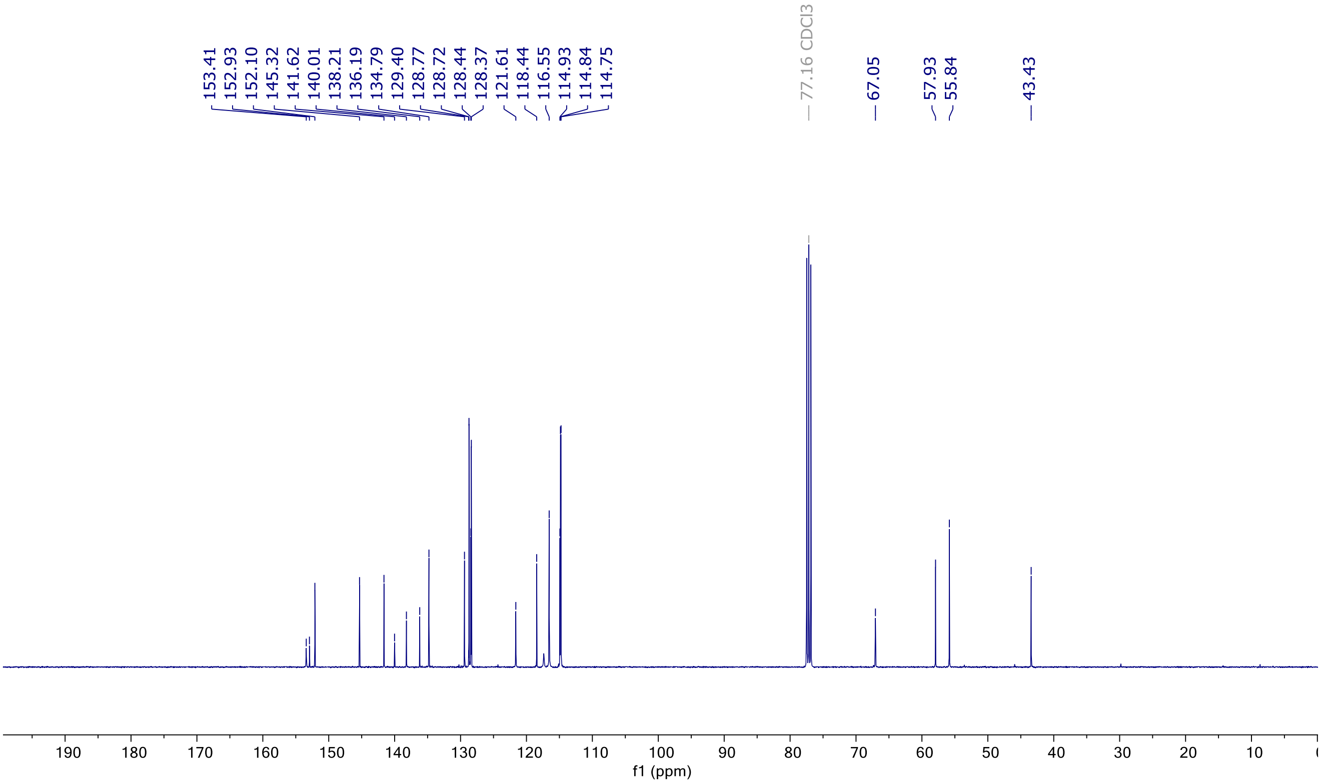

3k – ^{1}H-NMR (400 MHz, $CDCl_3$)

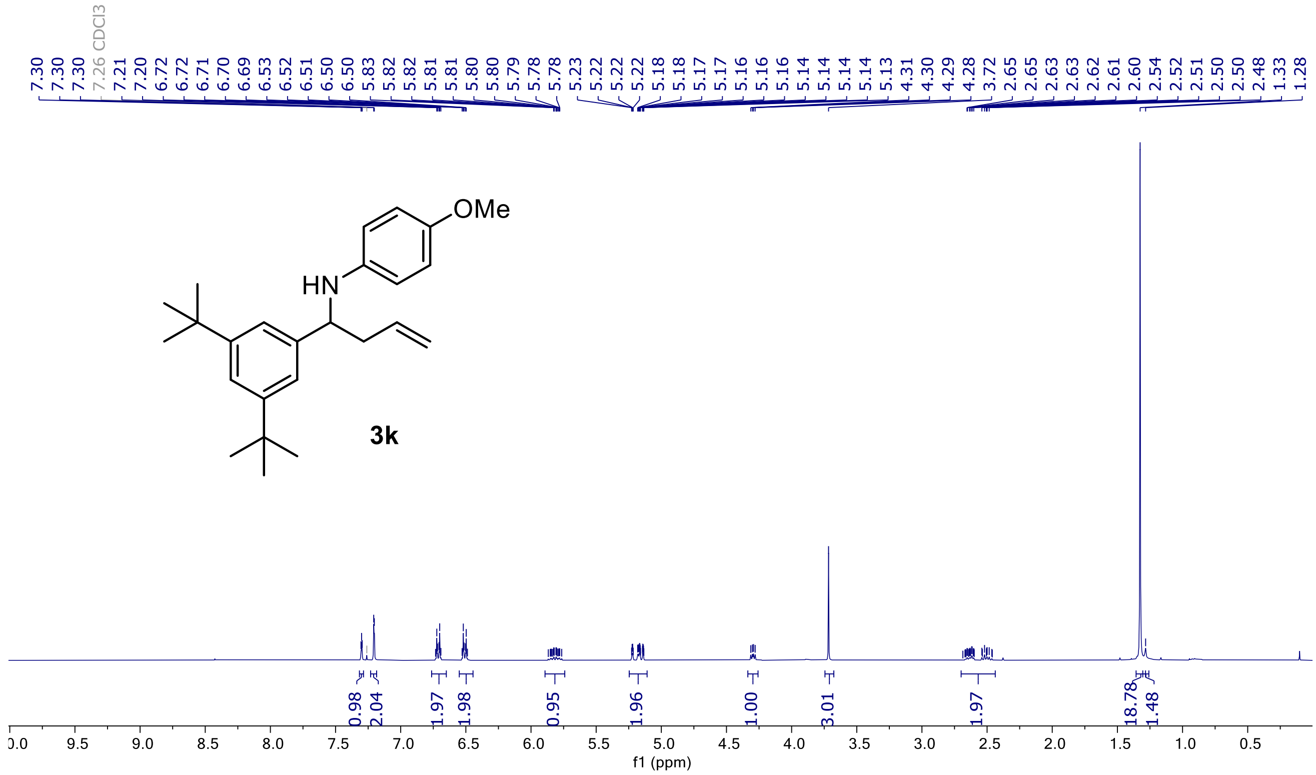

3k – ^{13}C-NMR (101 MHz, $CDCl_3$)

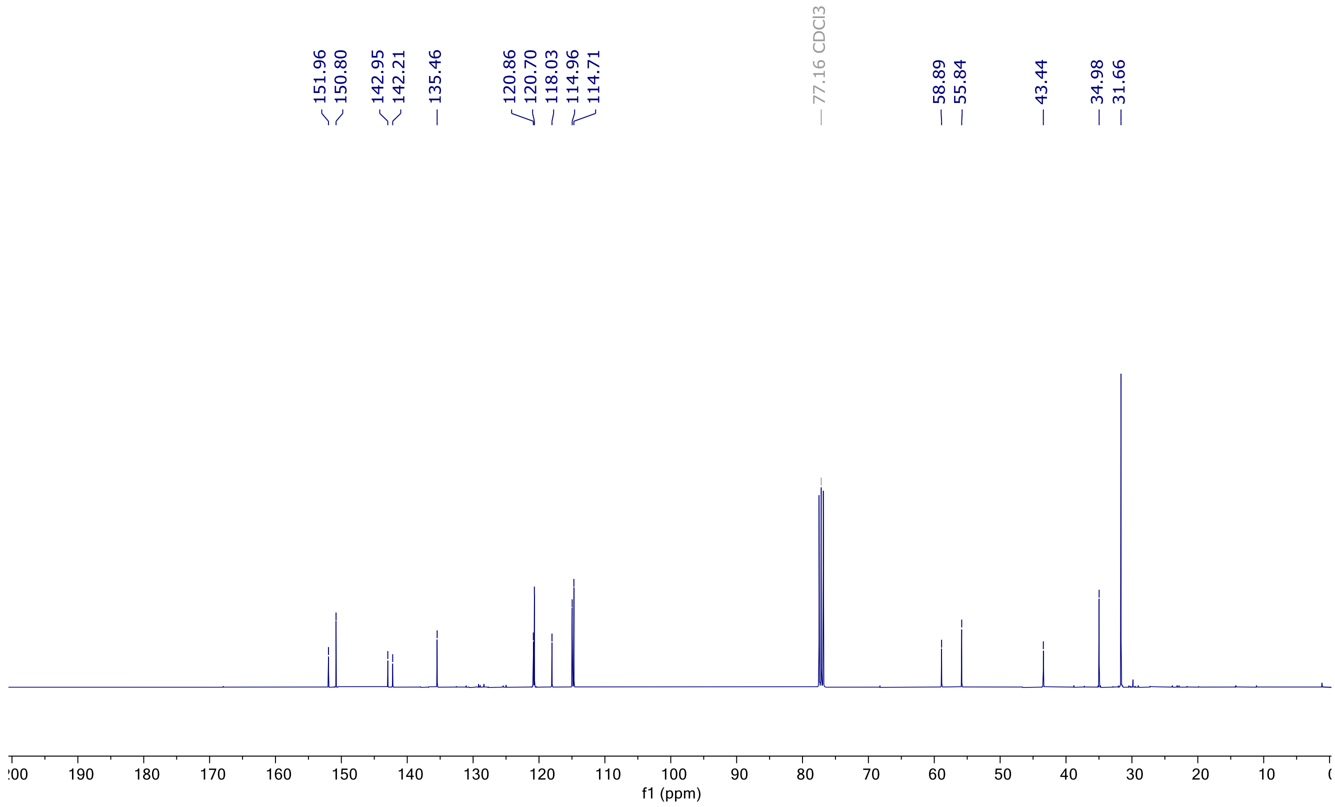

3l – ^{1}H-NMR (400 MHz, CDCl$_3$)

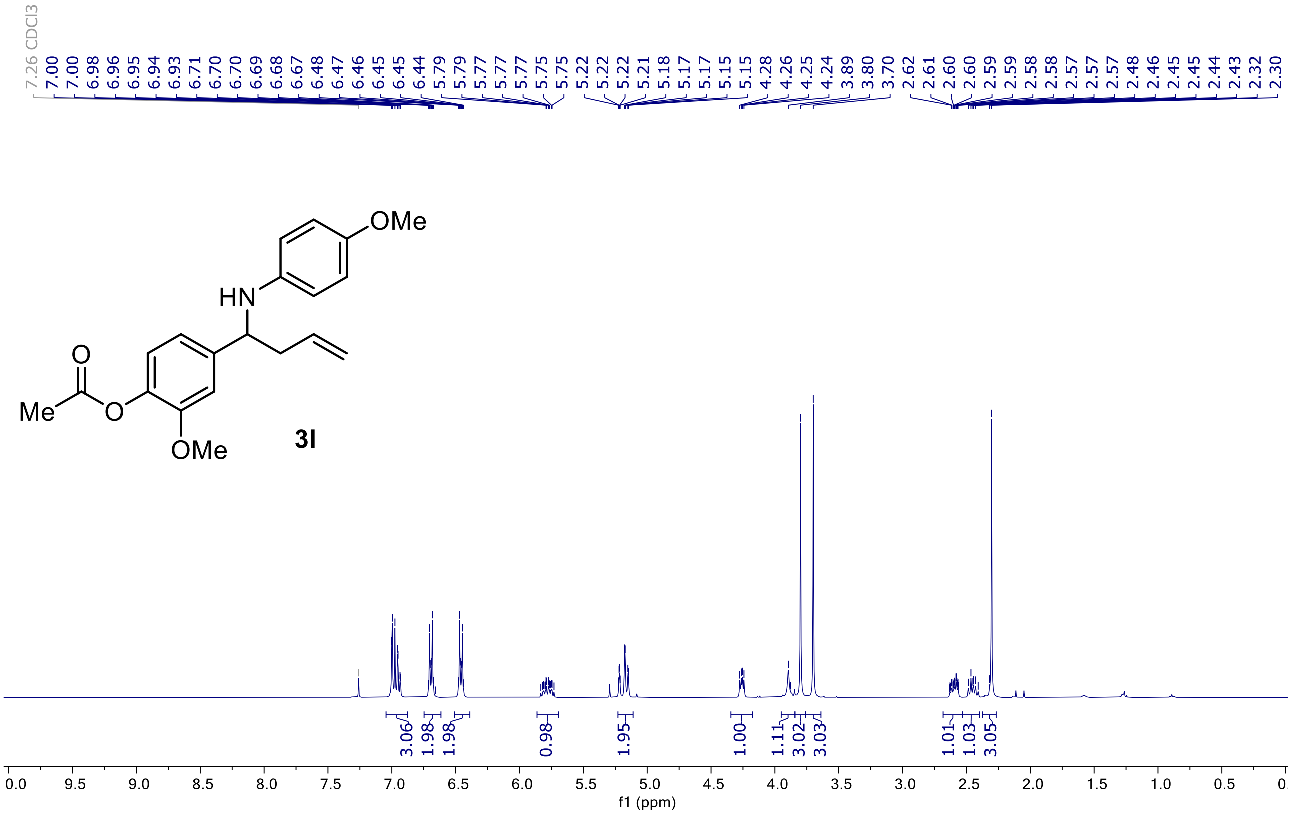

3l – ^{13}C-NMR (101 MHz, CDCl$_3$)

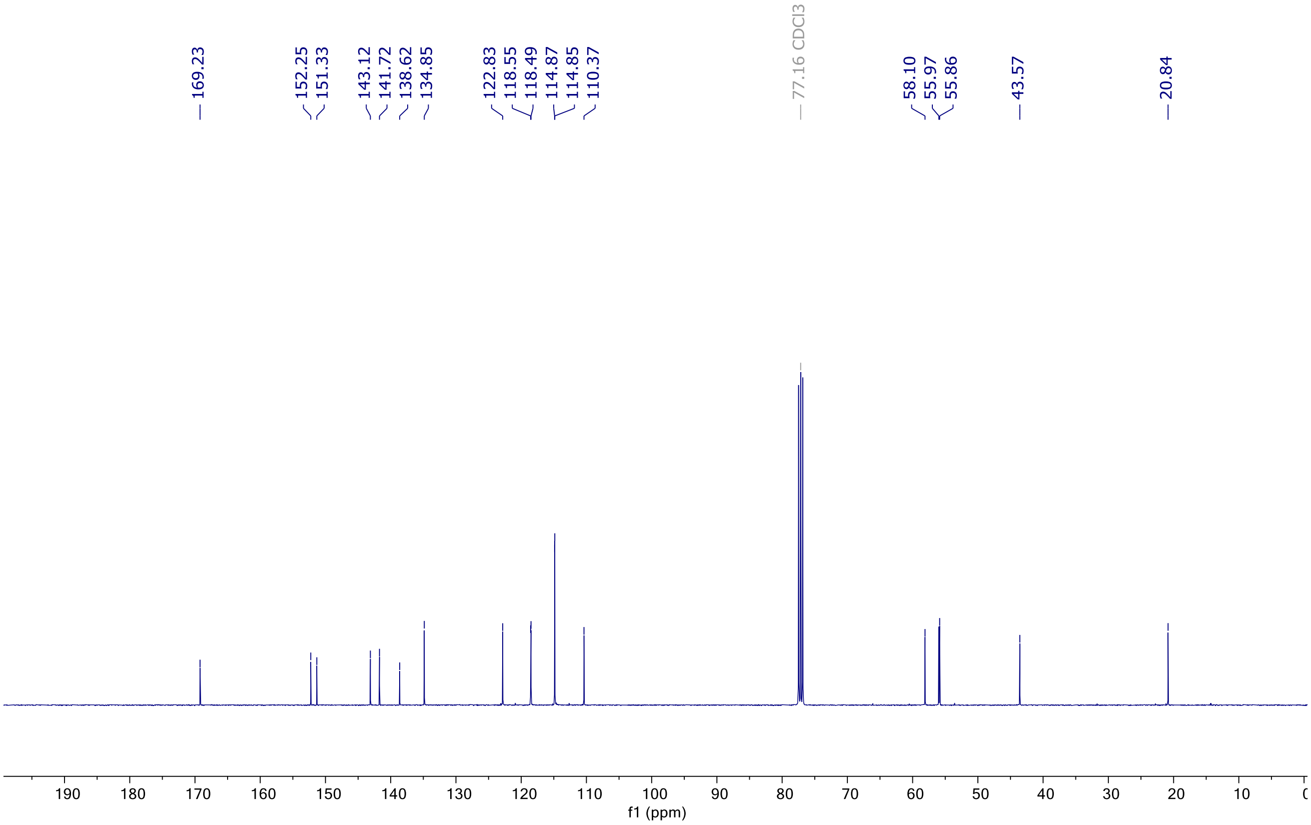

3m – ¹H-NMR (500 MHz, $CDCl_3$)

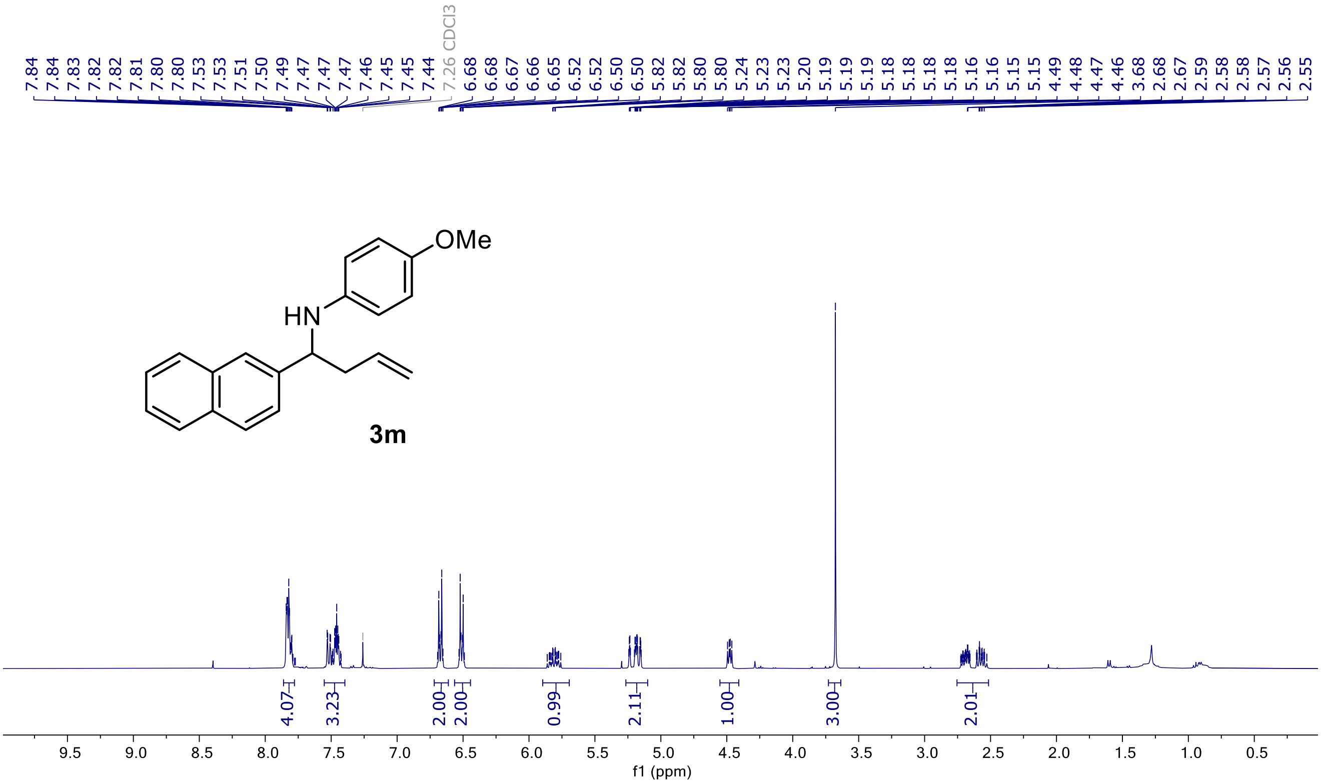

3m – ¹³C-NMR (126 MHz, $CDCl_3$)

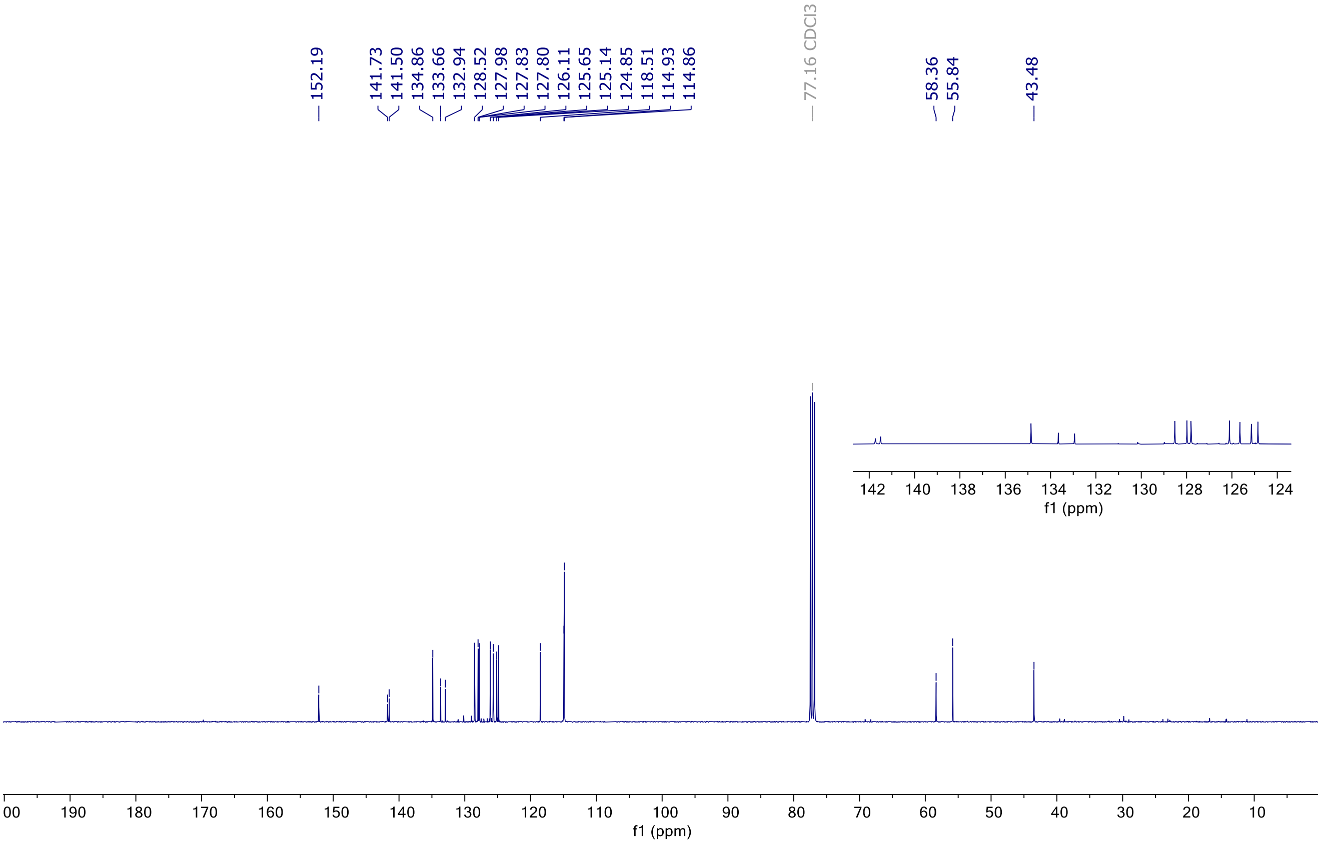

3n – ^{1}H-NMR (400 MHz, CDCl$_3$)

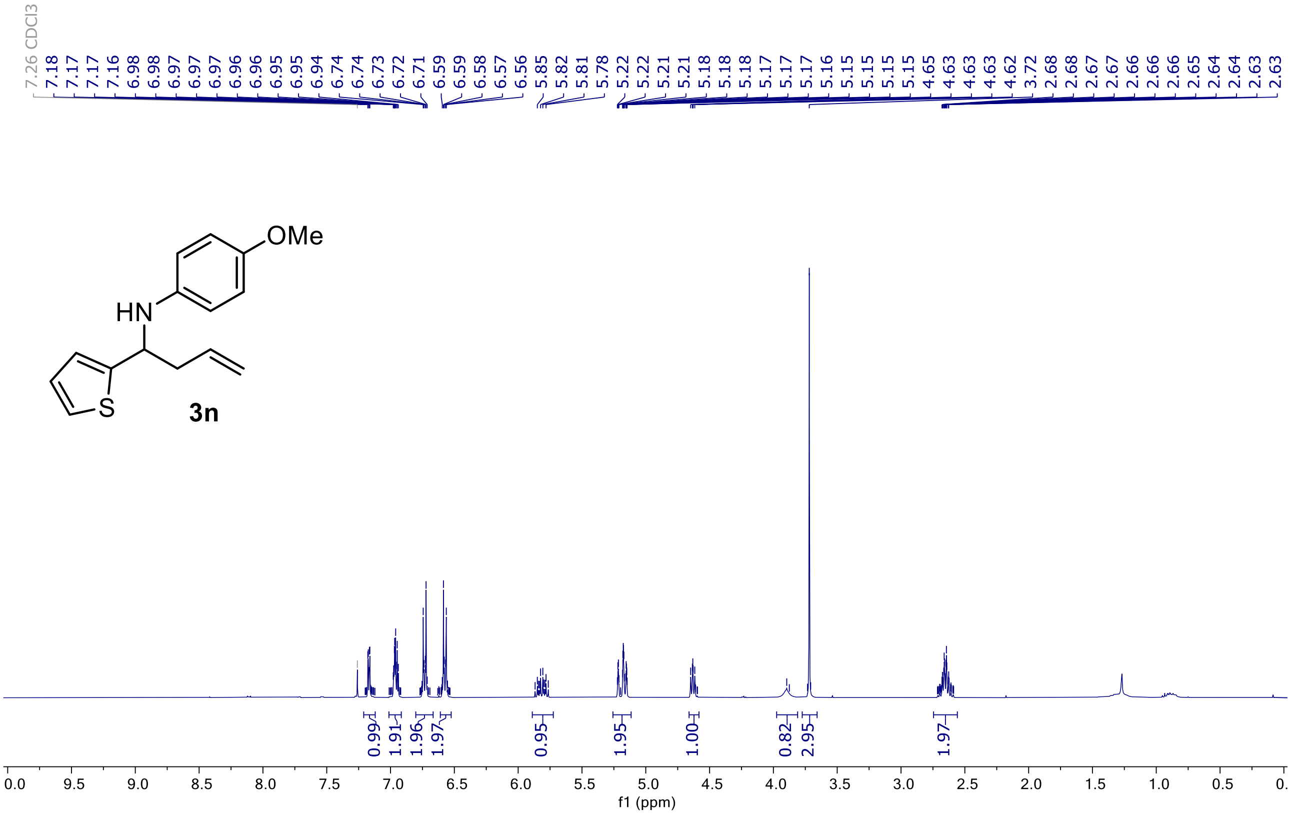

3n – ^{13}C-NMR (101 MHz, CDCl$_3$)

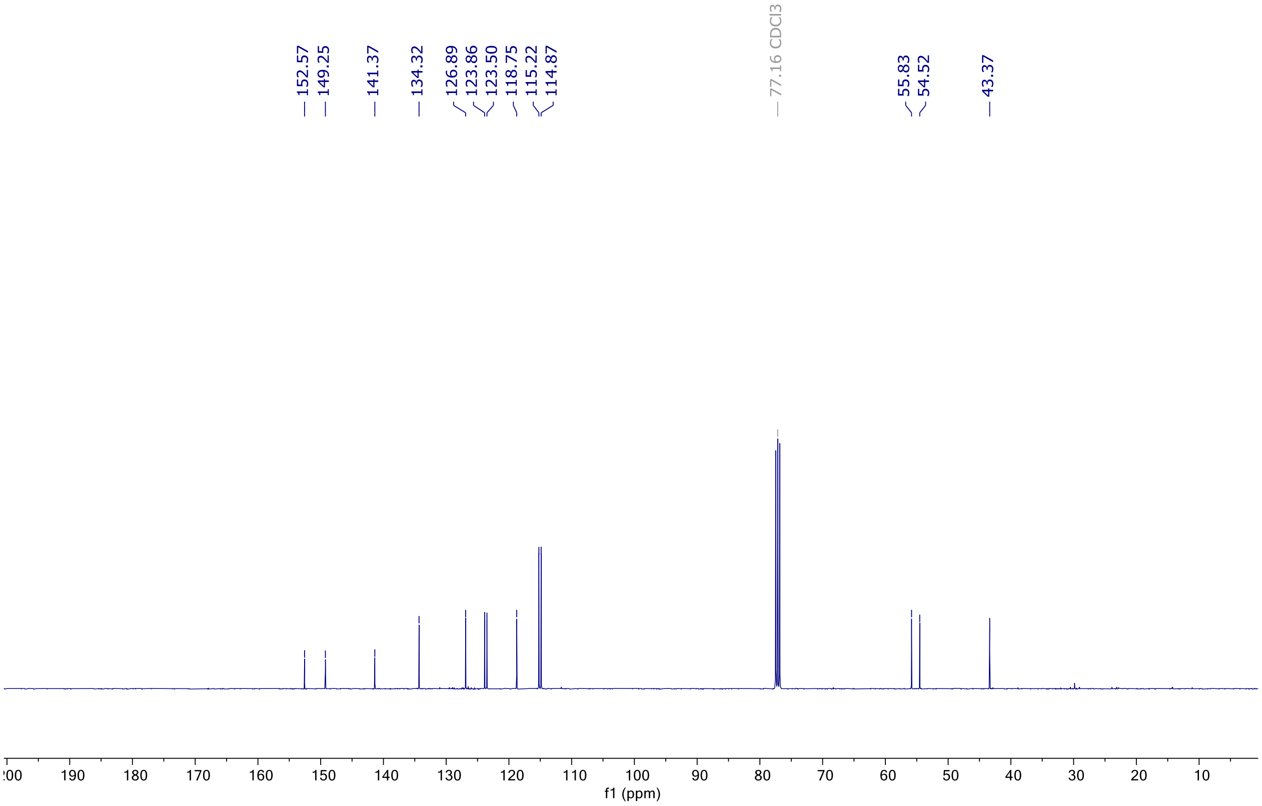

3o – ^{1}H-NMR (400 MHz, CDCl₃)

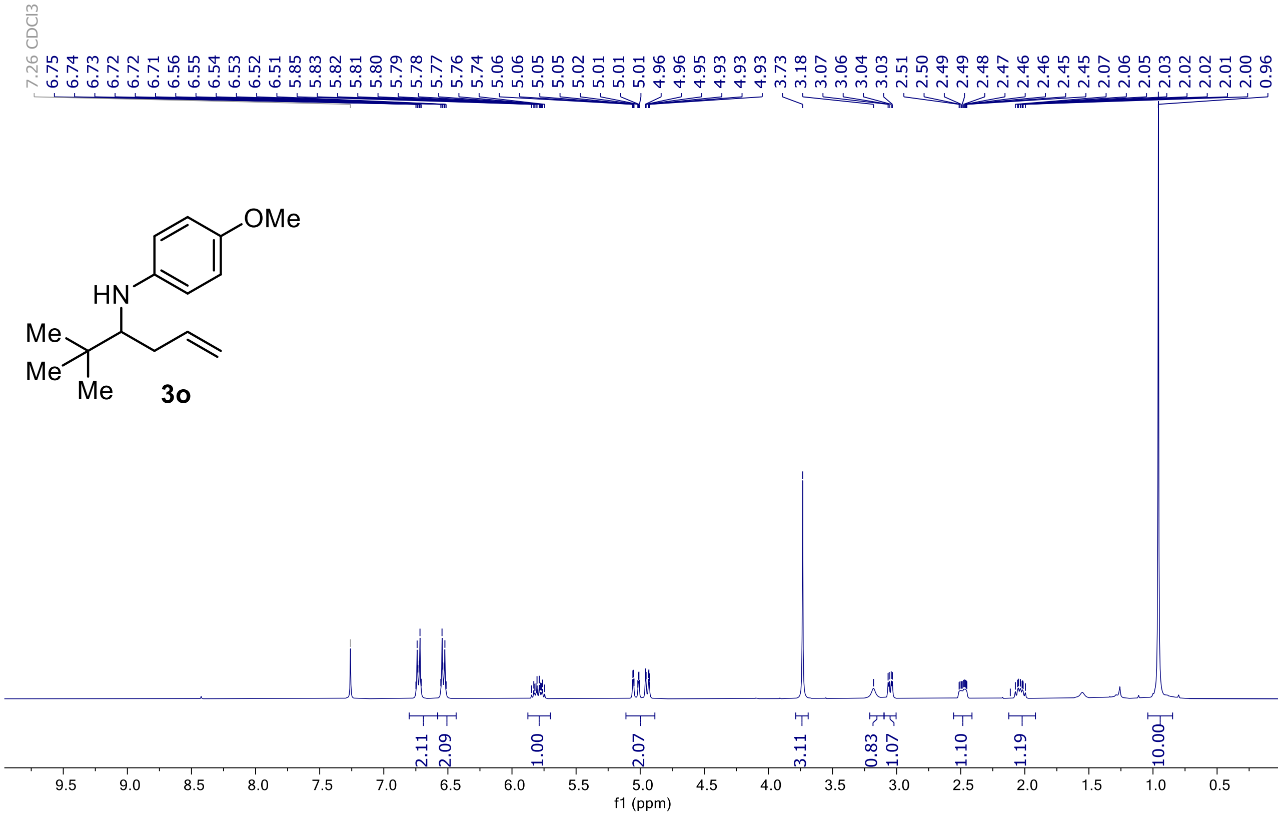

3o – ^{13}C-NMR (101 MHz, CDCl₃)

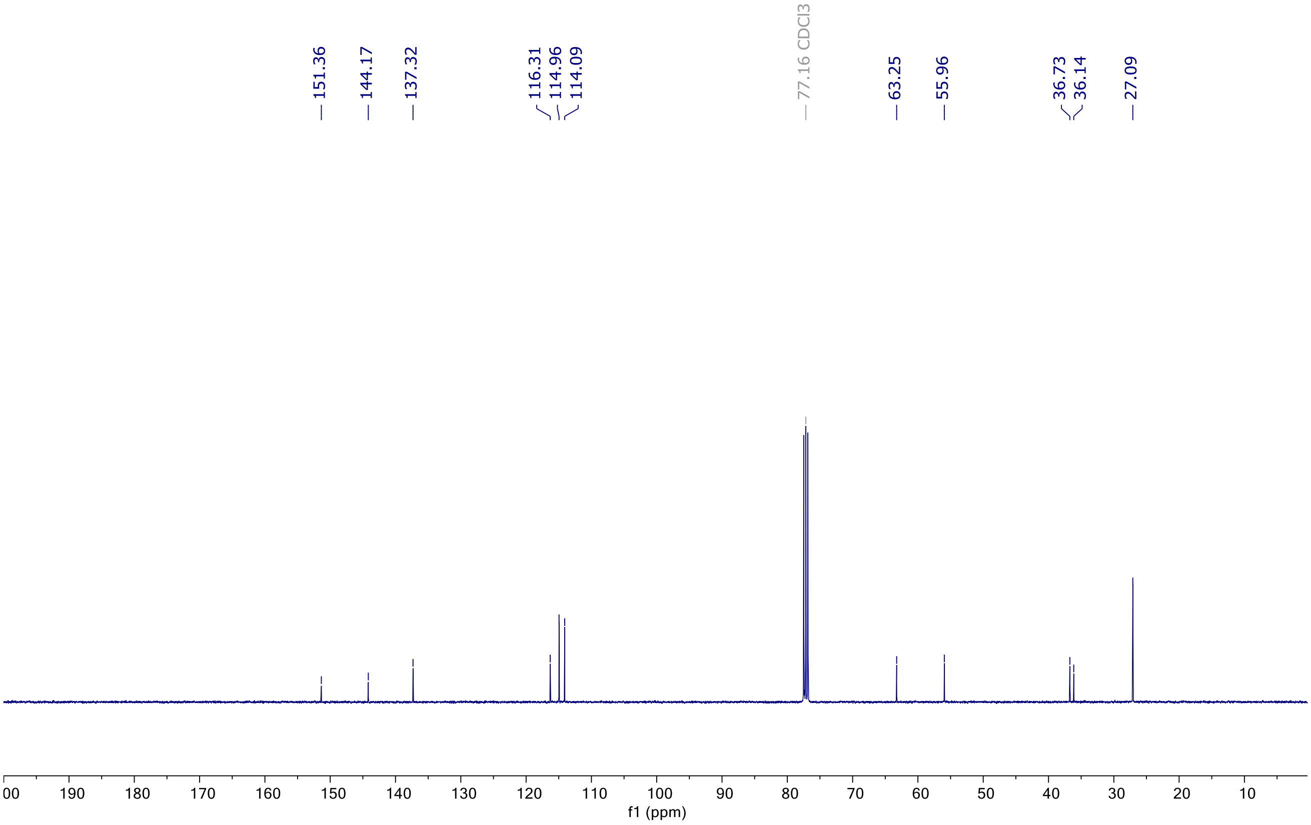

3p – ^{1}H-NMR (400 MHz, $CDCl_3$)

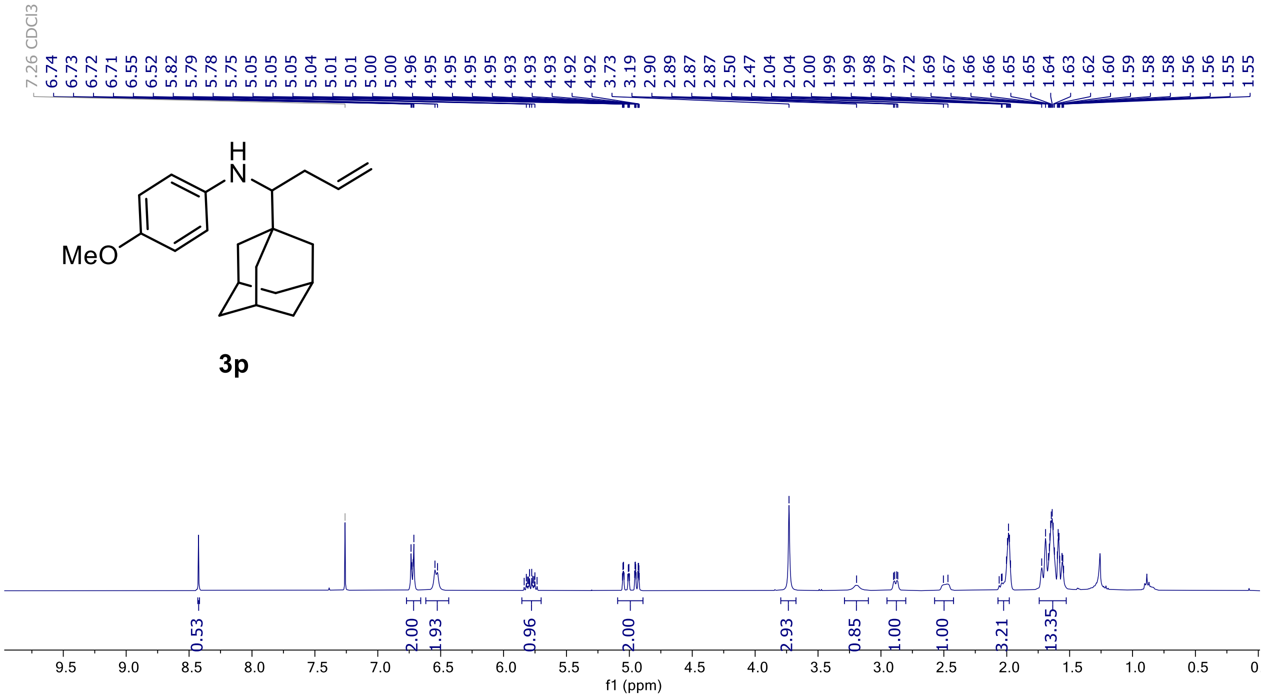

3p – ^{13}C-NMR (101 MHz, $CDCl_3$)

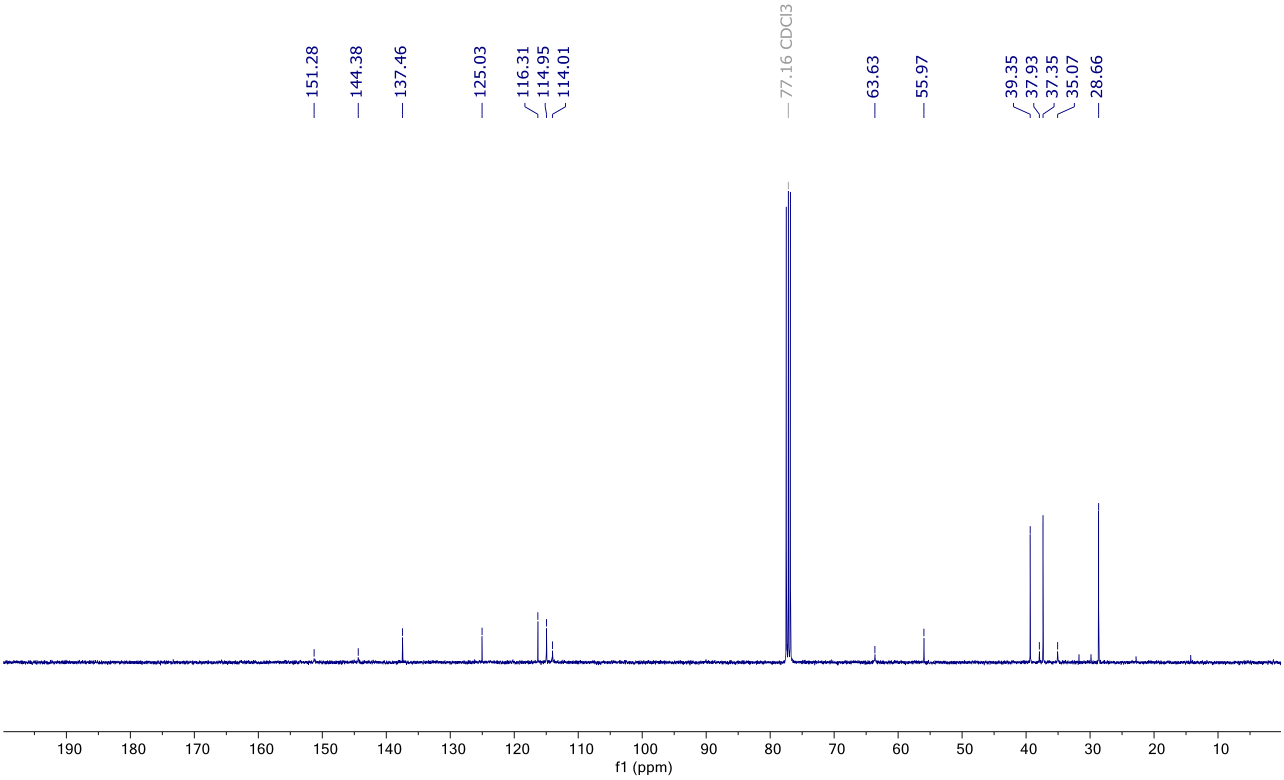

3q – ^{1}H-NMR (600 MHz, $CDCl_3$)

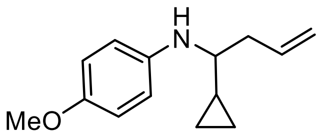

3q

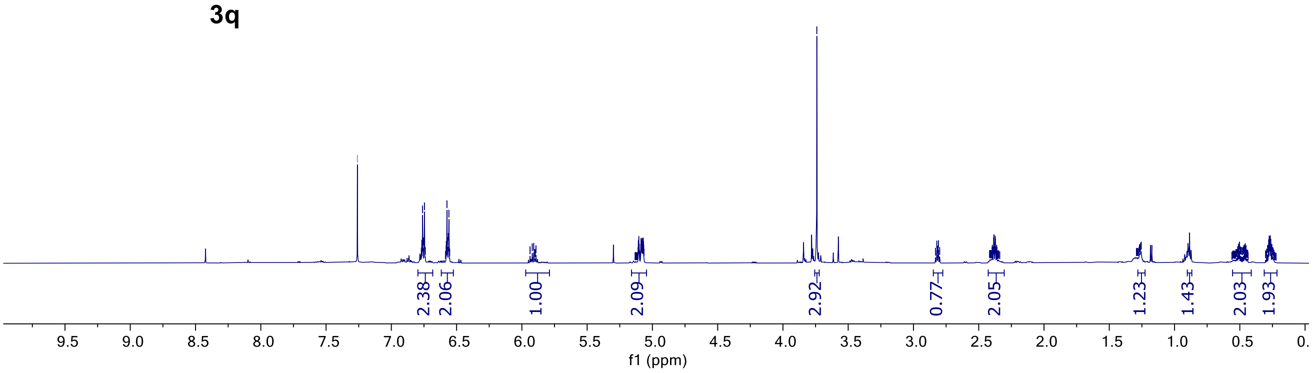

3q – ^{13}C-NMR (151 MHz, $CDCl_3$)

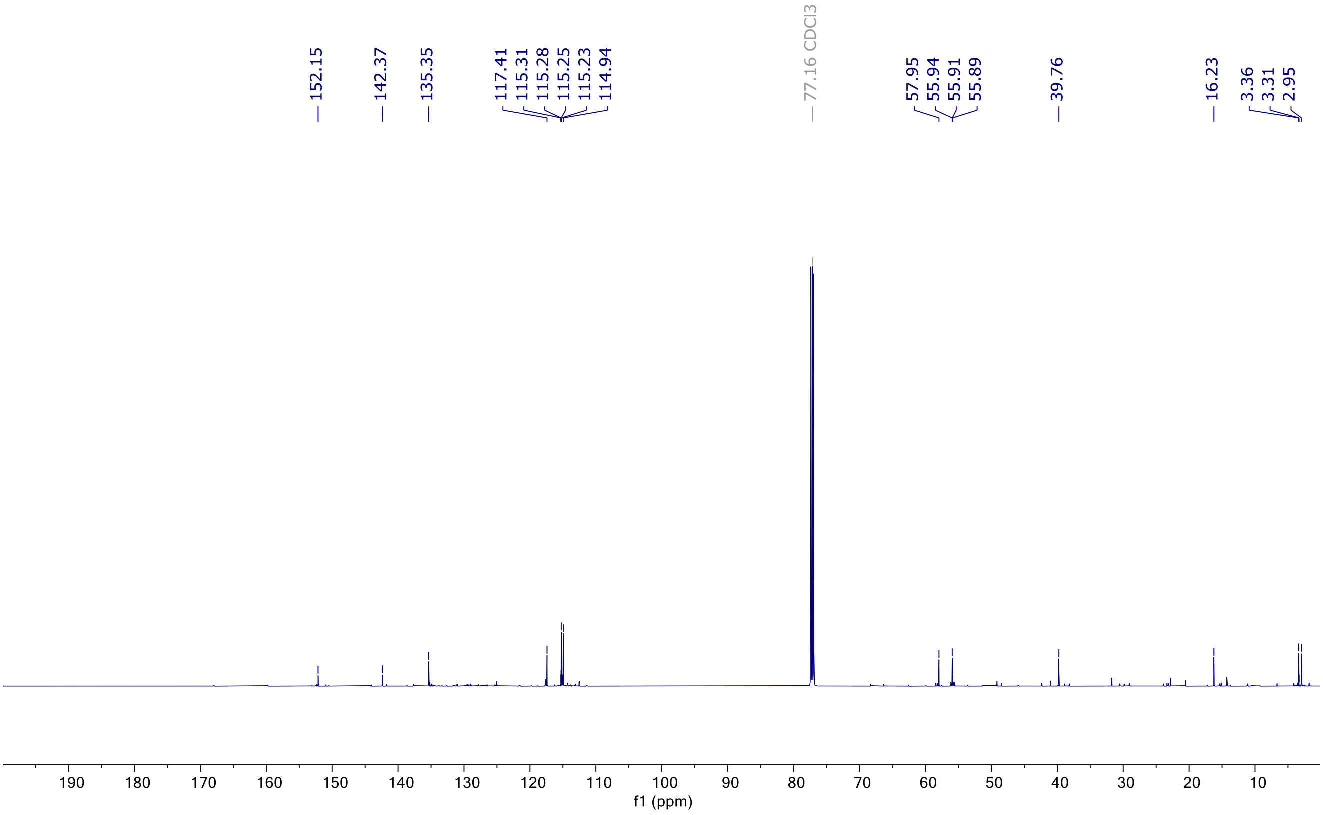

3r – ^{1}H-NMR (400 MHz, $CDCl_3$)

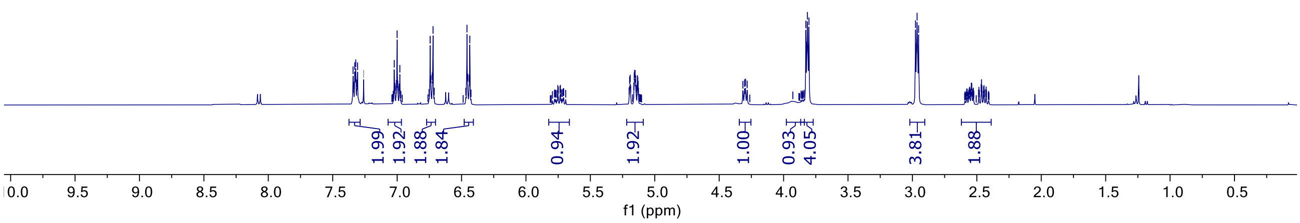

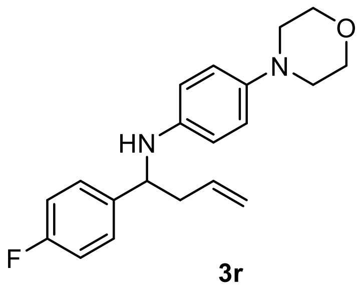

3r

3r – ^{13}C-NMR (101 MHz, $CDCl_3$)

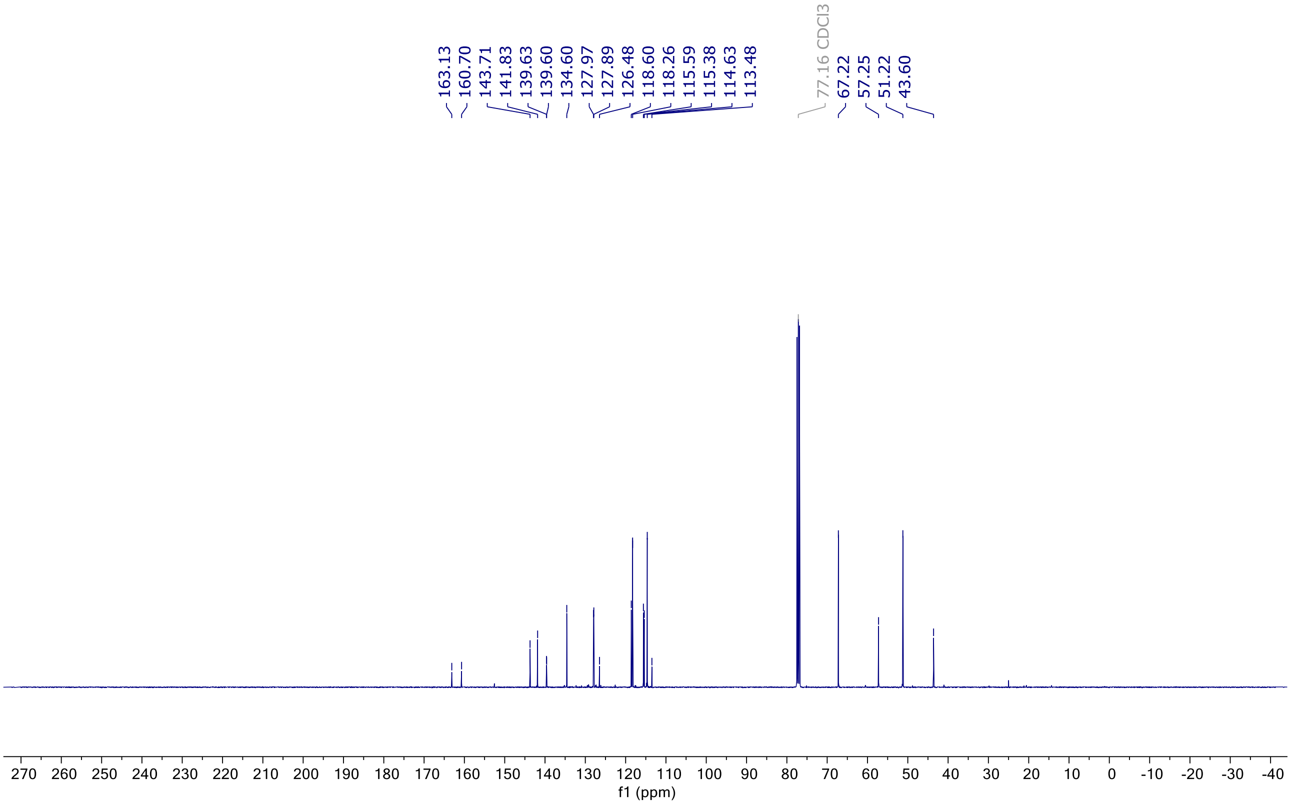

3r – ^{19}F NMR (471 MHz, $CDCl_3$)

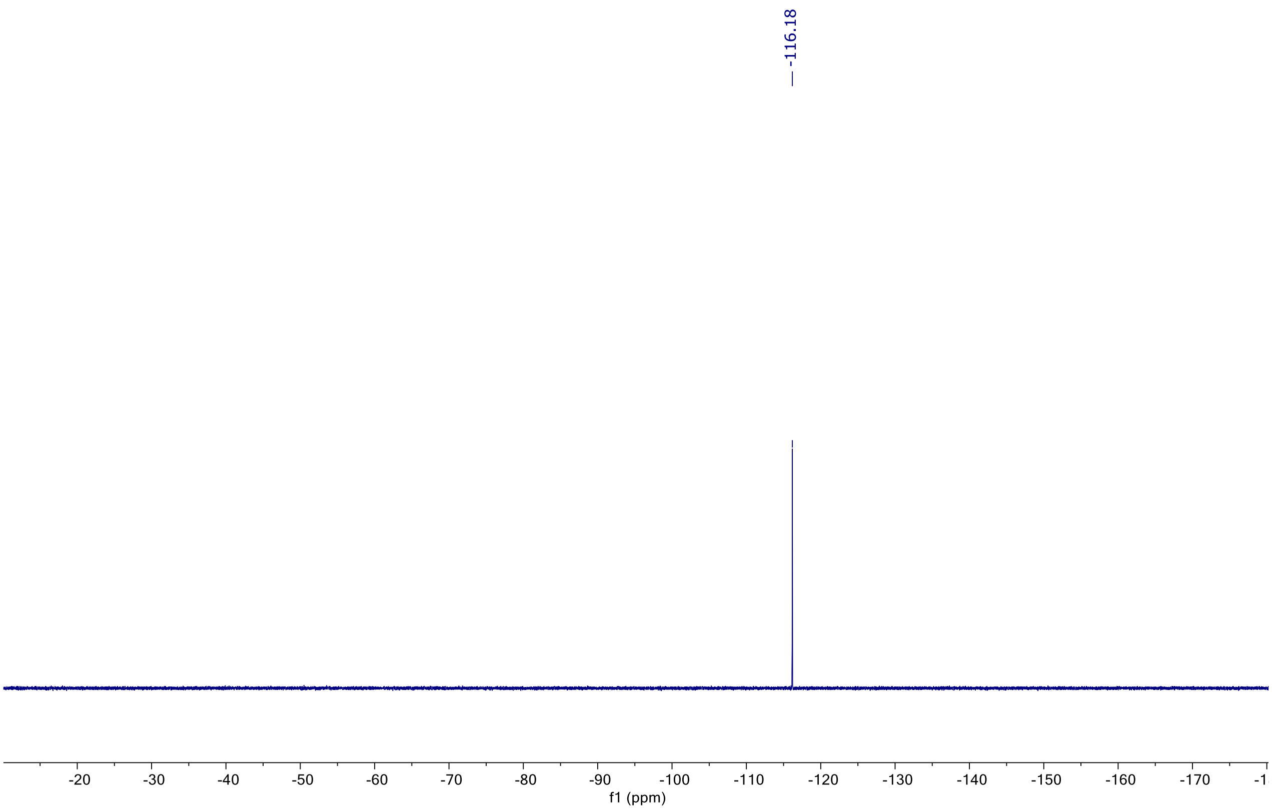

3s – ^{1}H-NMR (400 MHz, $CDCl_3$)

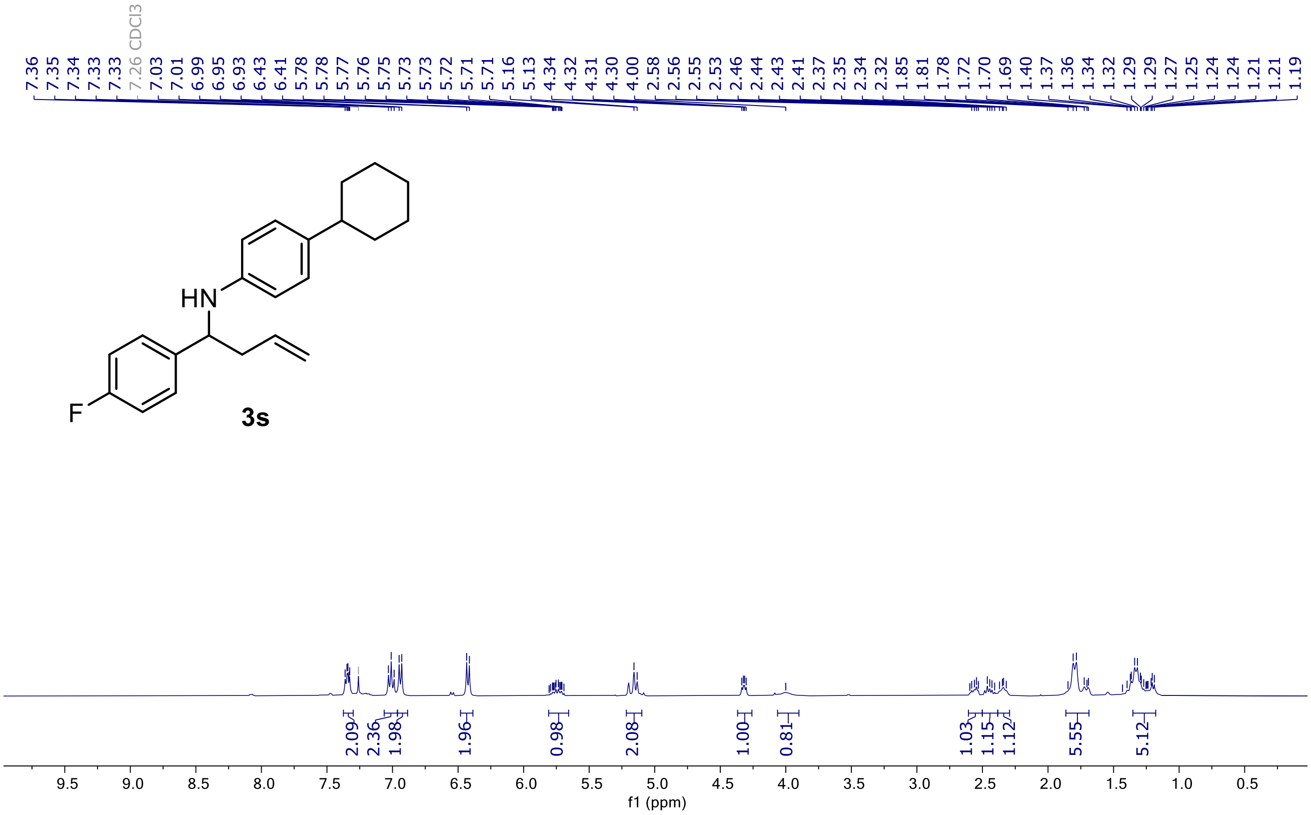

3s – ^{13}C-NMR (101 MHz, $CDCl_3$)

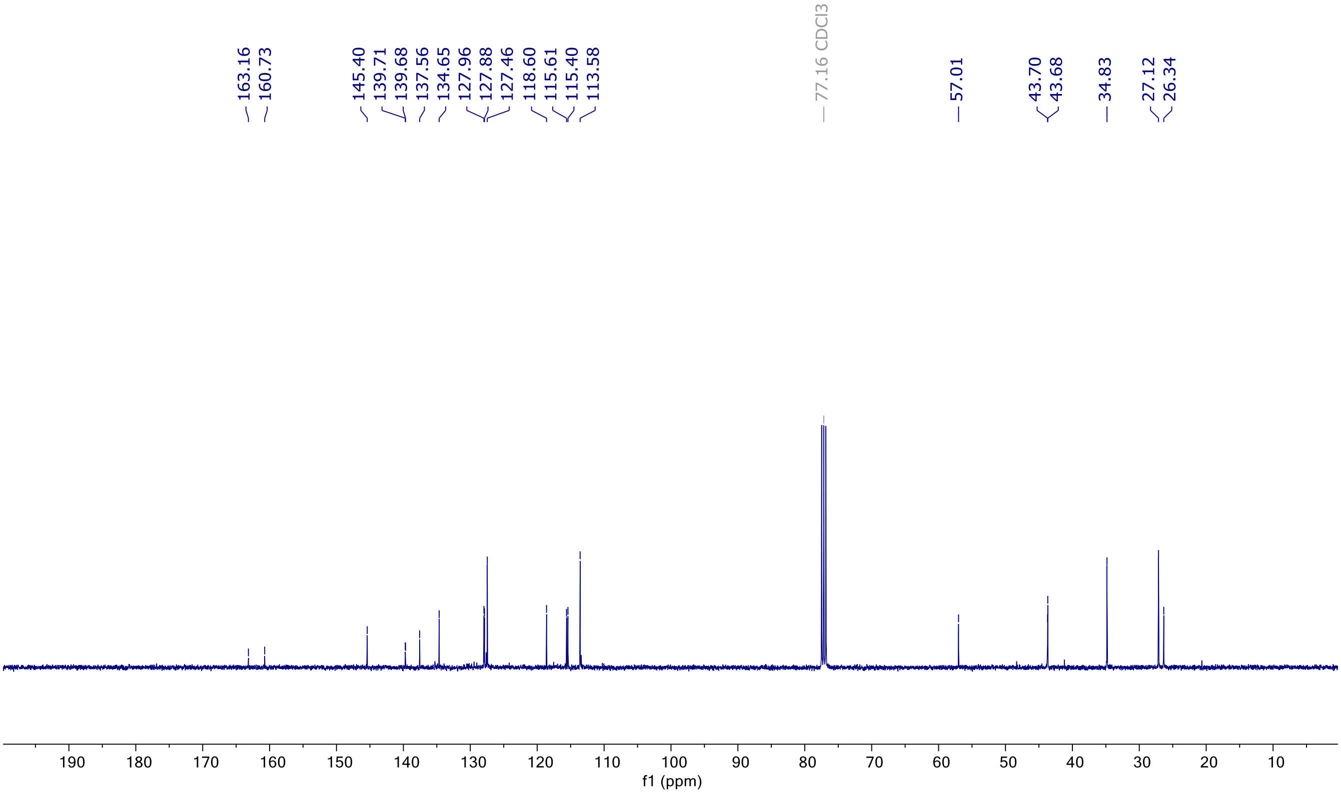

3s – ^{19}F NMR (471 MHz, $CDCl_3$)

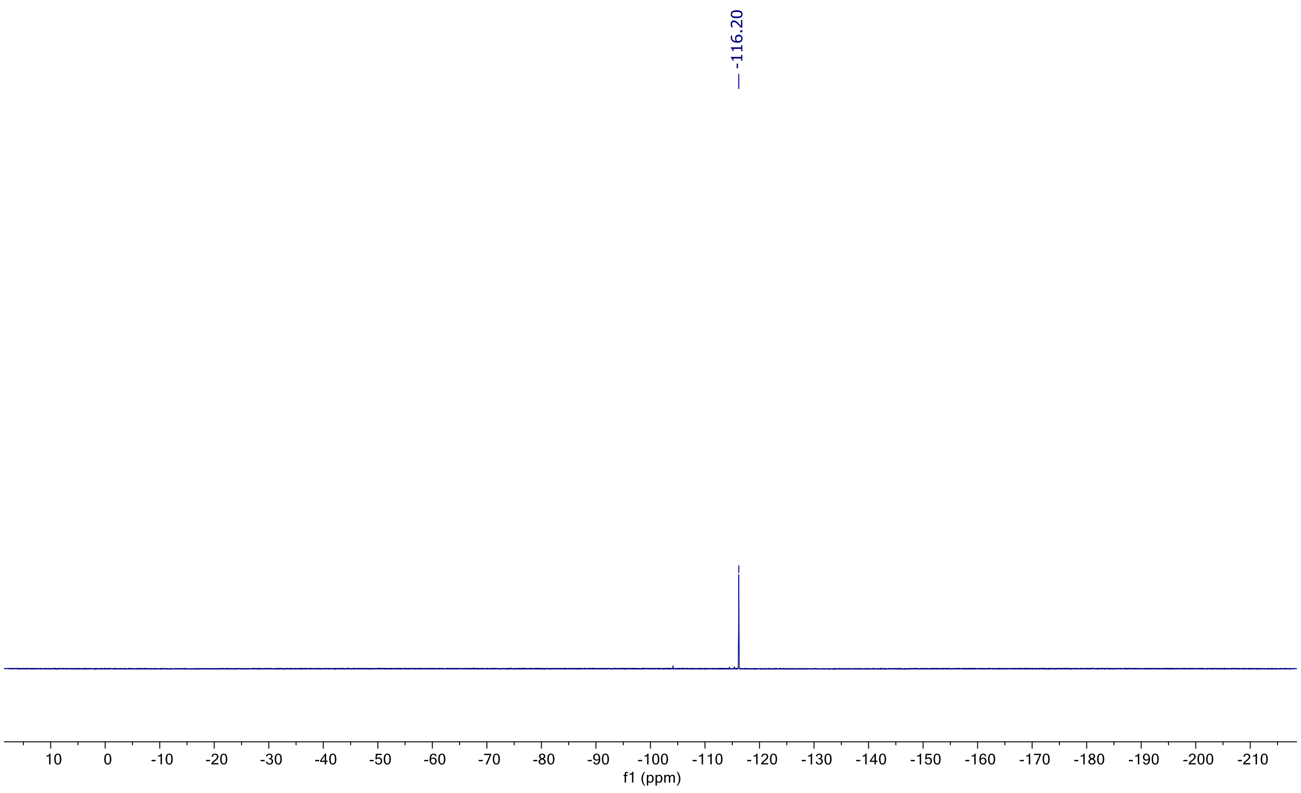

3t – ^{1}H-NMR (400 MHz, $CDCl_3$)

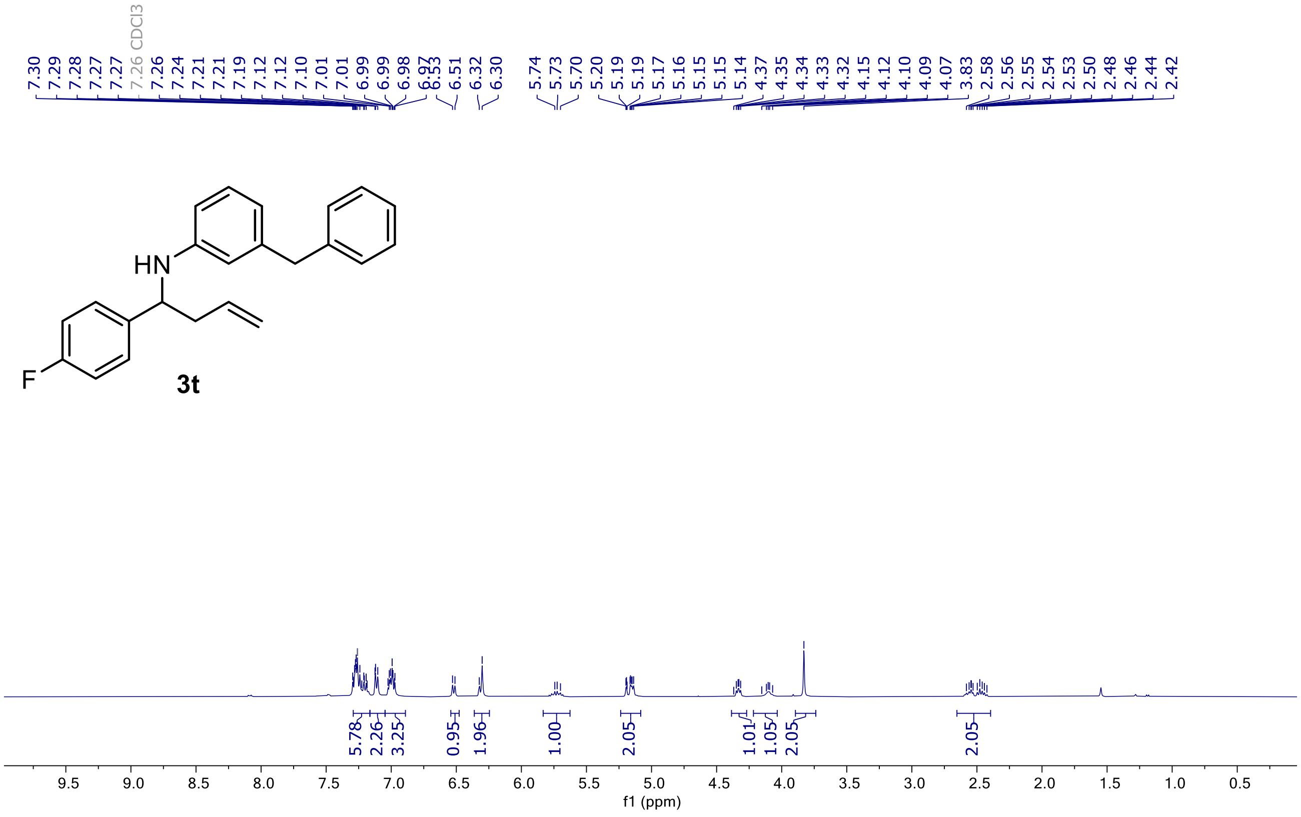

3t – ^{13}C-NMR (101 MHz, $CDCl_3$)

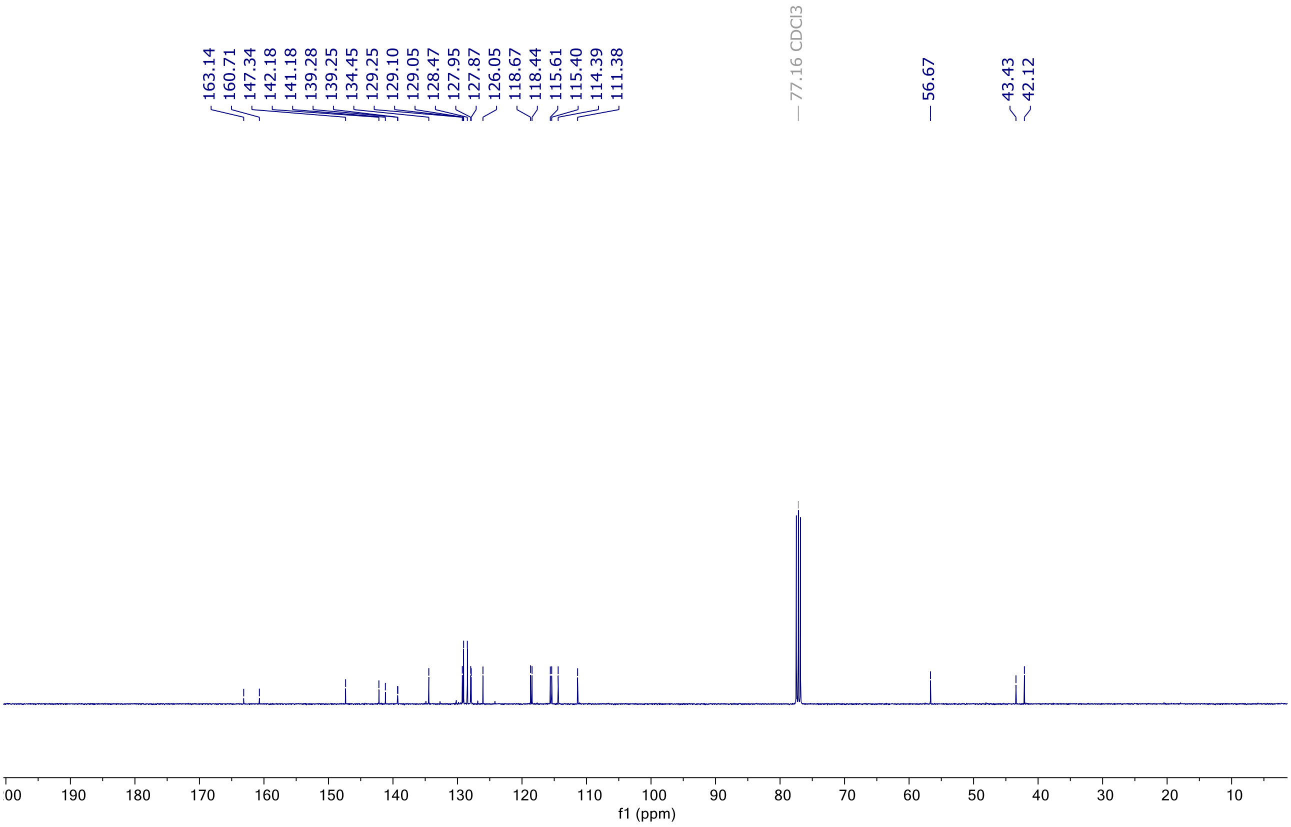

3t – ^{19}F NMR (471 MHz, $CDCl_3$)

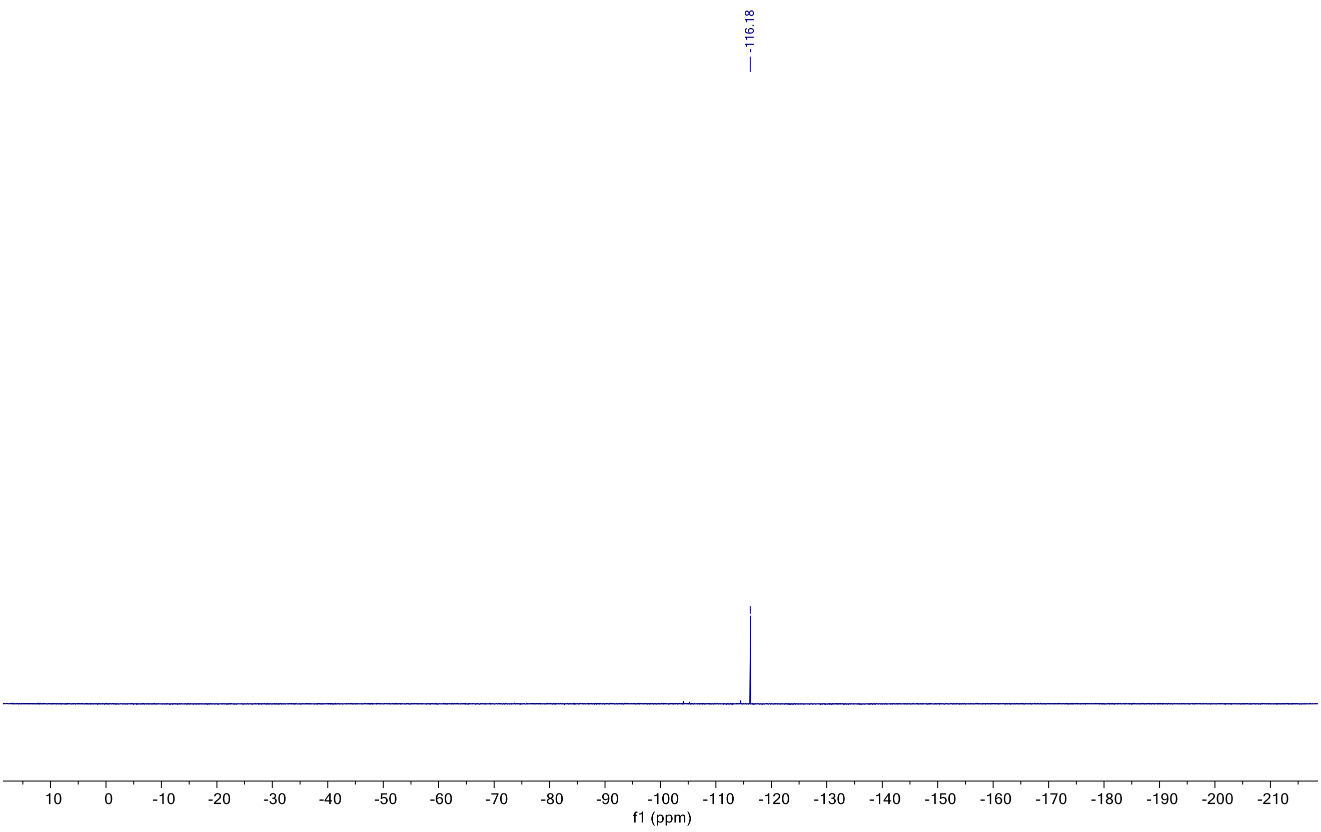

3u – ^{1}H-NMR (400 MHz, $CDCl_3$)

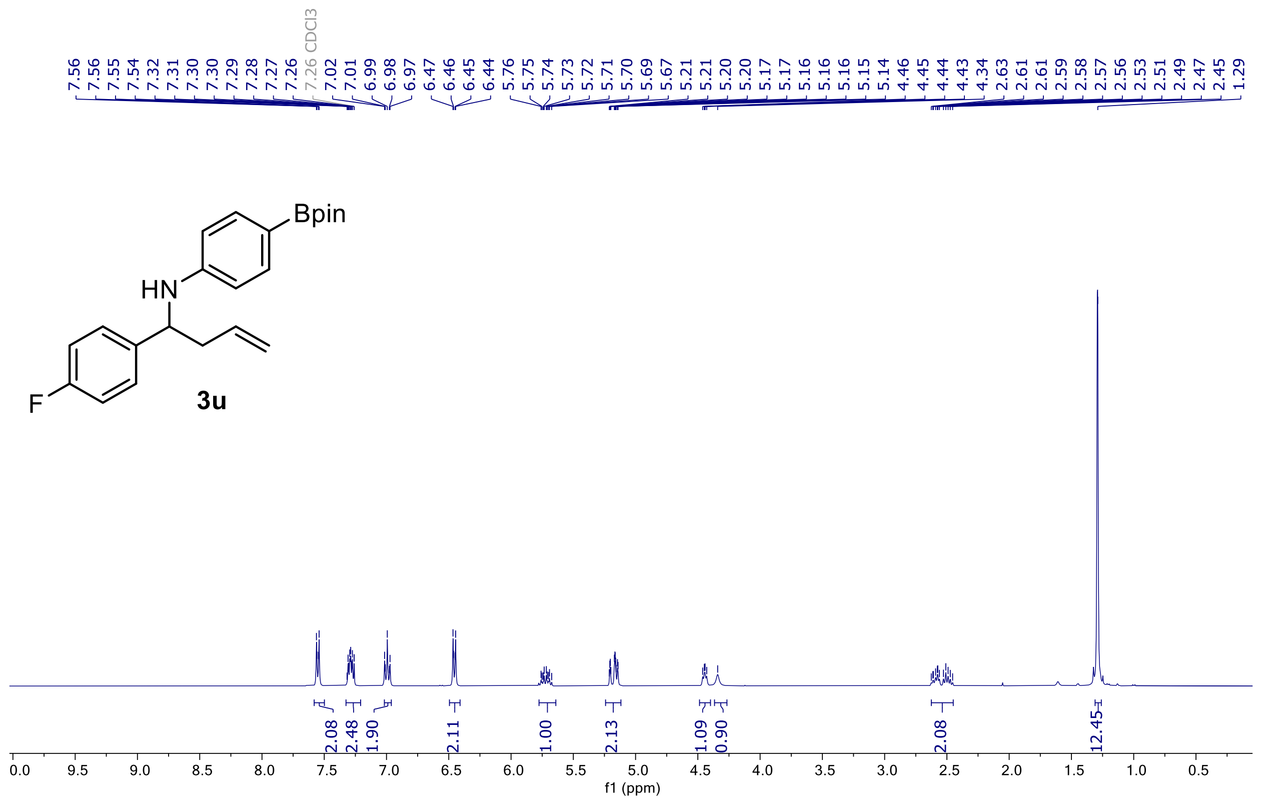

3u – ^{13}C-NMR (101 MHz, $CDCl_3$)

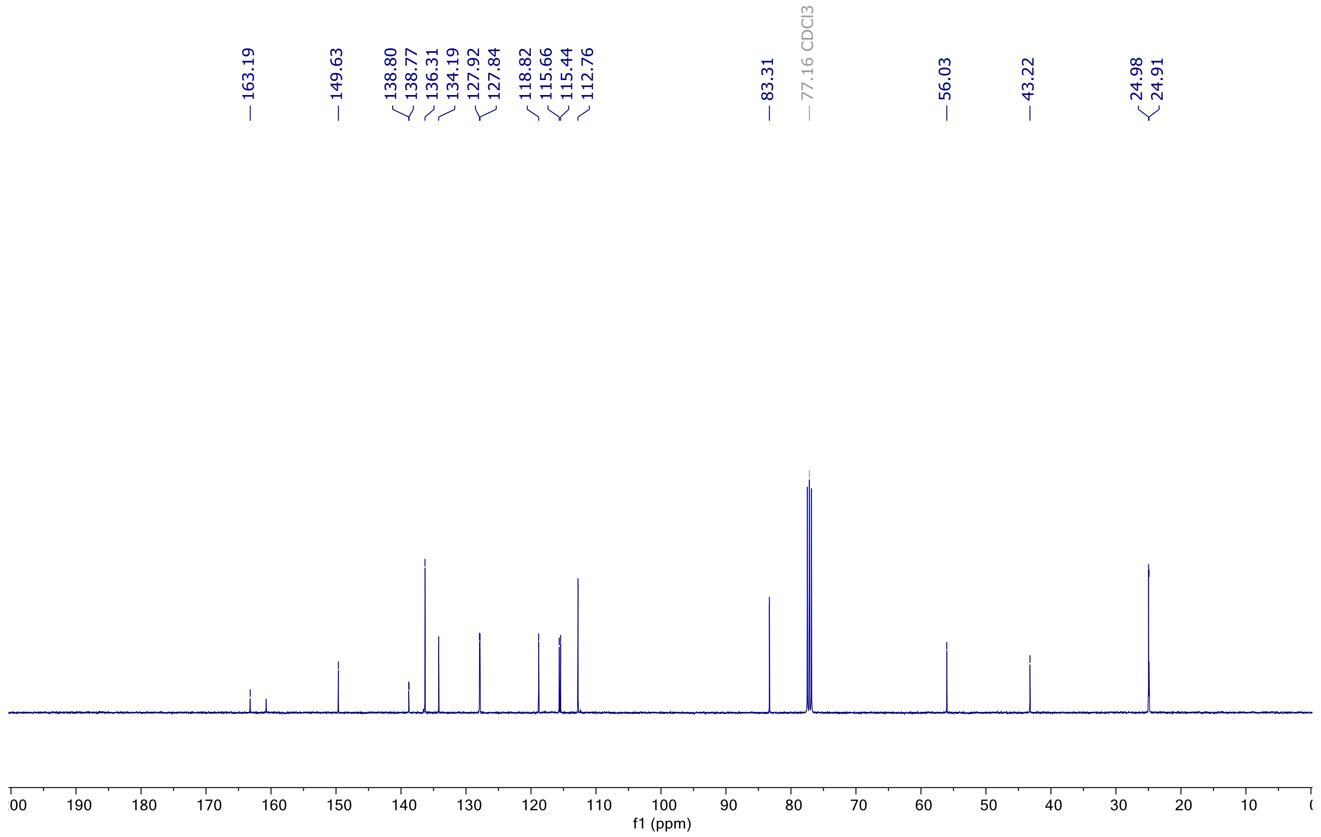

3u – ^{19}F NMR (471 MHz, $CDCl_3$)

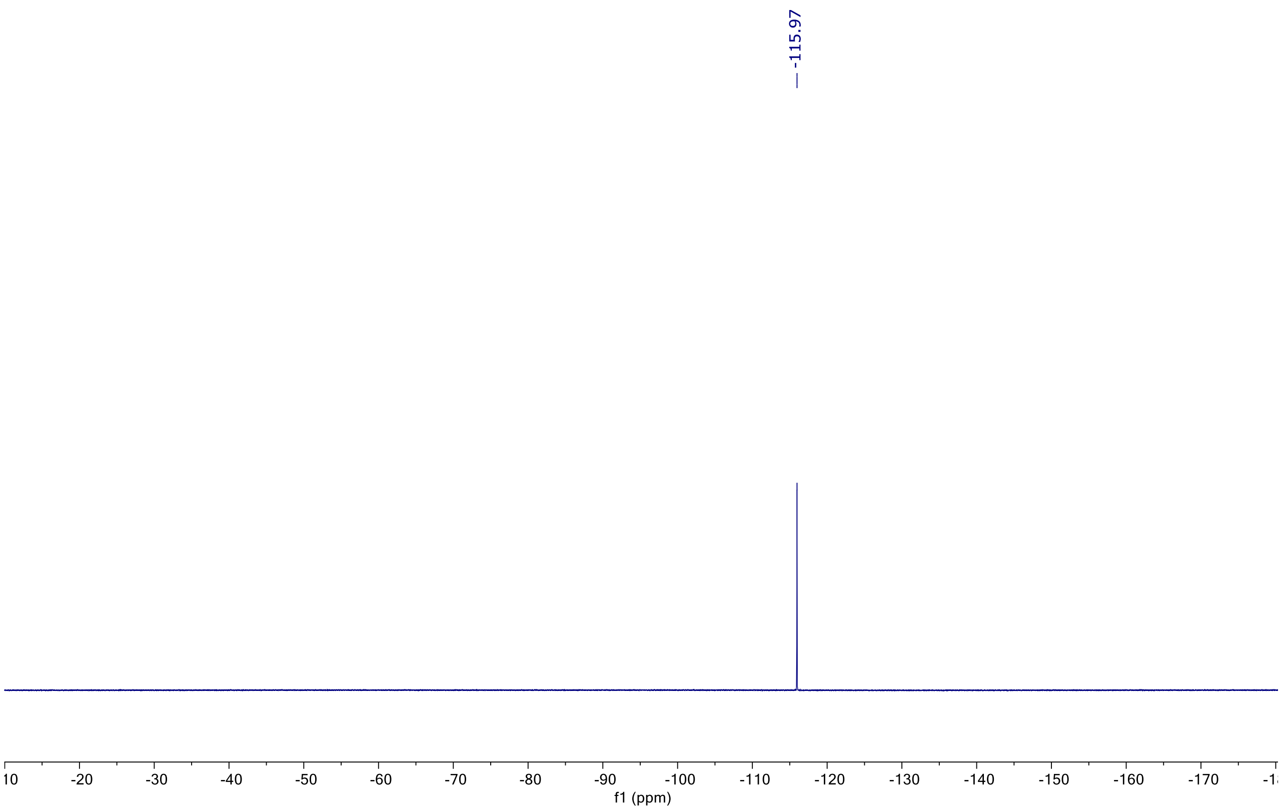

3u – ^{11}B NMR (128 MHz, $CDCl_3$)

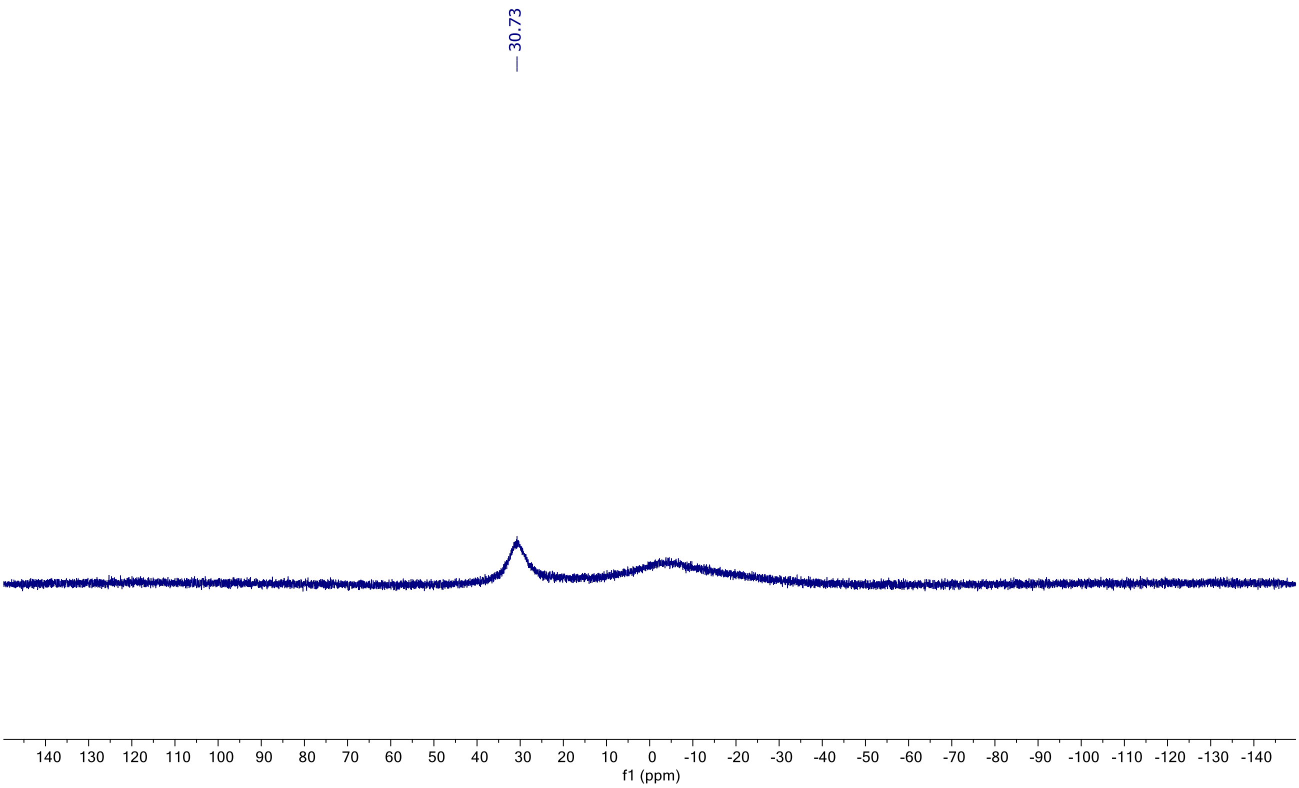

7.3 NMR Spectra of Olefination Products

4a – ^{1}H-NMR (400 MHz, $CDCl_3$)

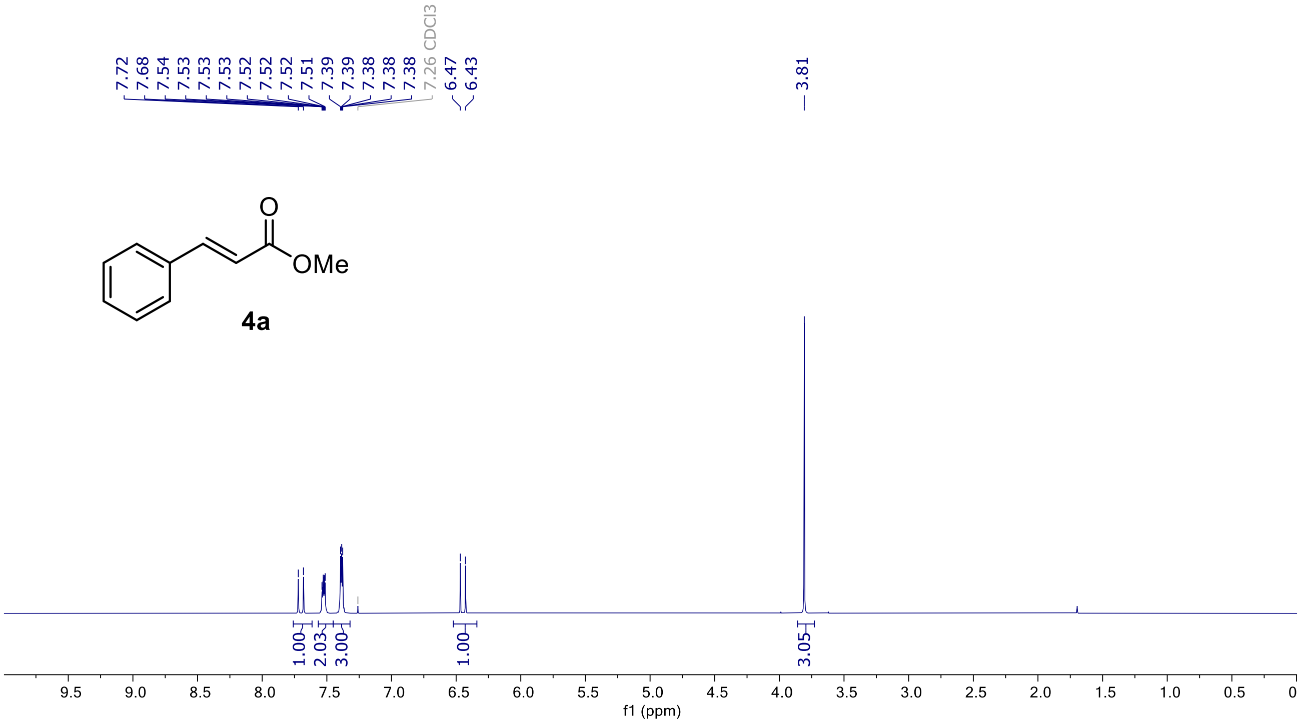

4a

4a – ^{13}C-NMR (101 MHz, $CDCl_3$)

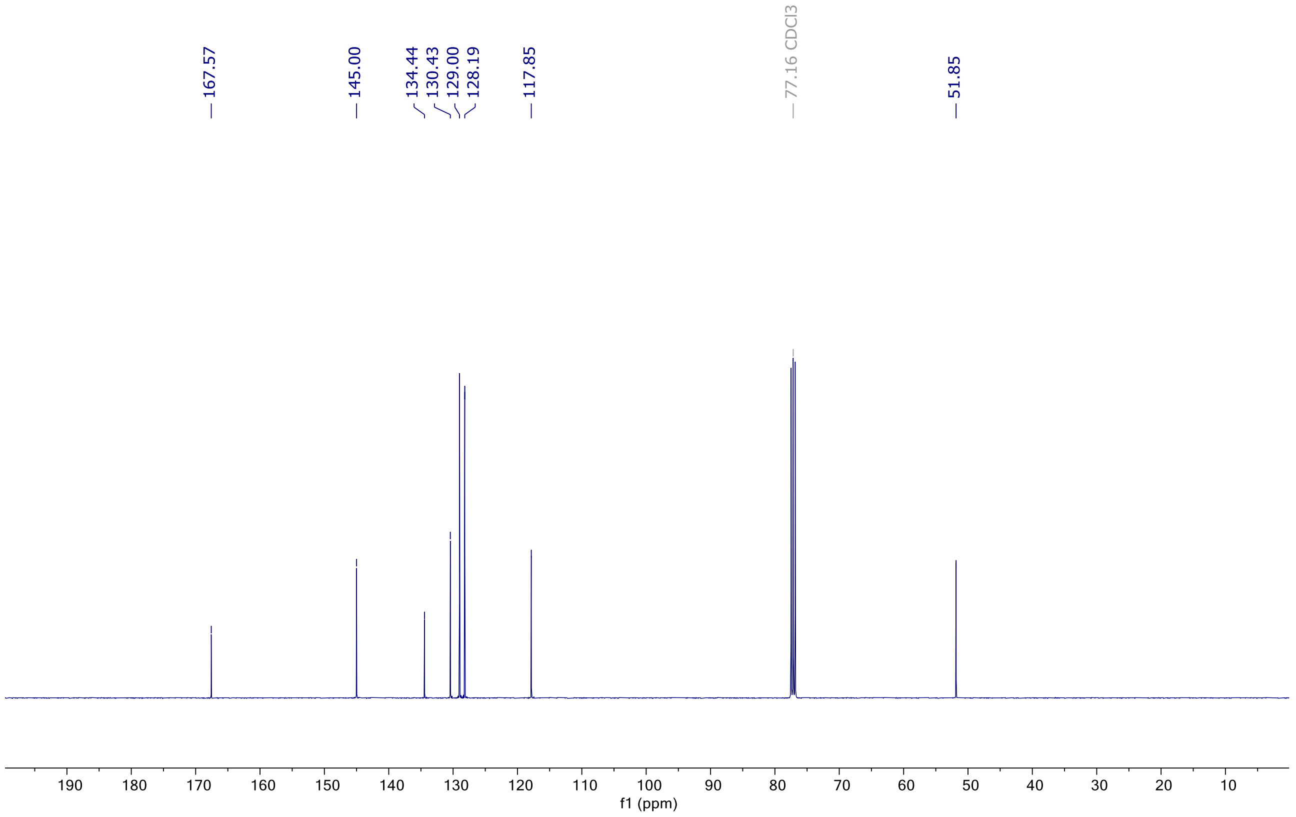

4v – ^{1}H-NMR (400 MHz, CDCl3)

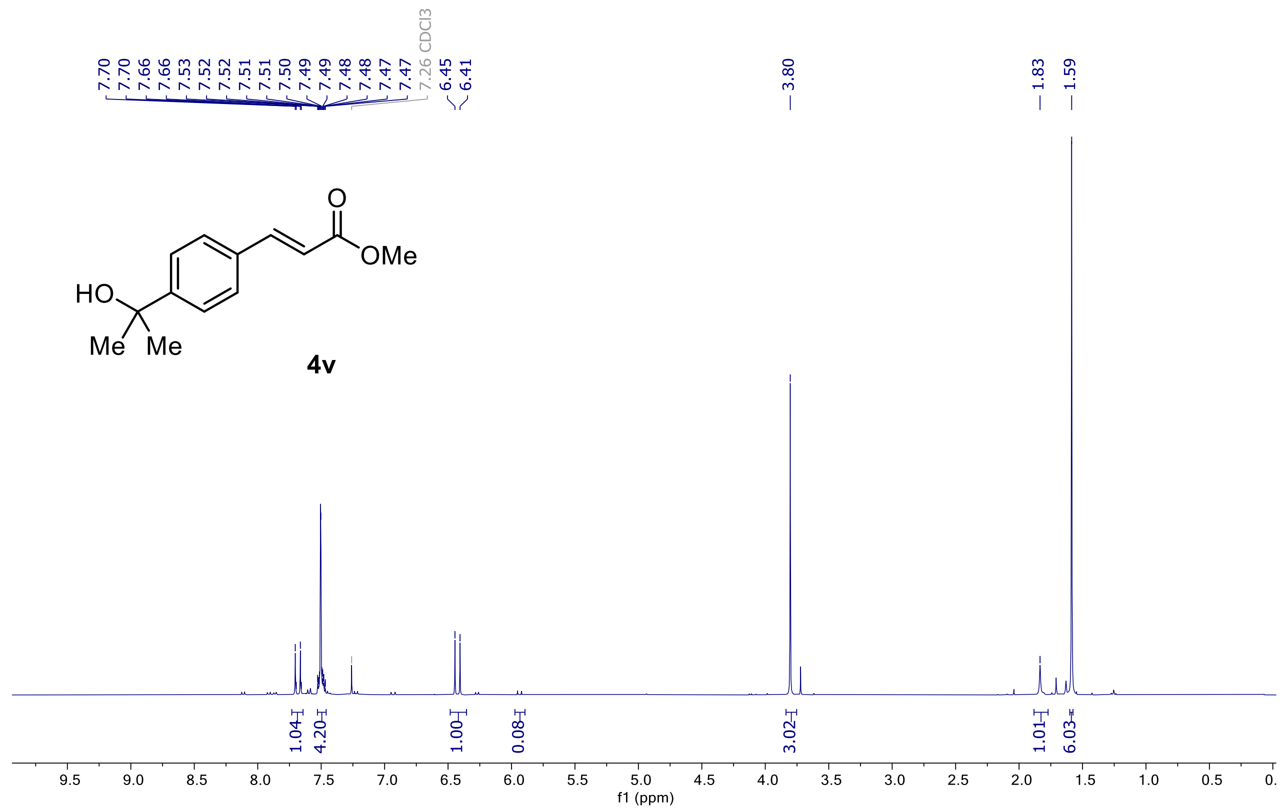

4v – ^{13}C-NMR (101 MHz, CDCl3)

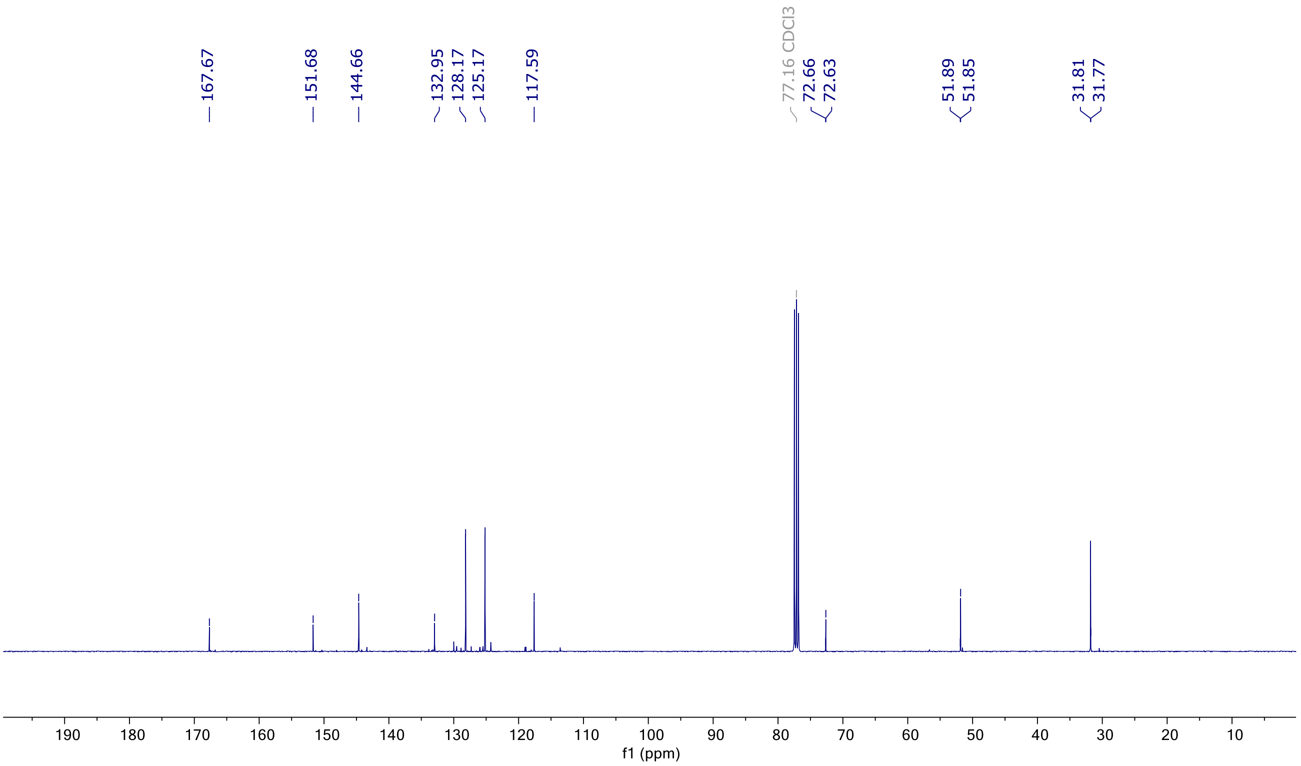

4w – ^{1}H-NMR (400 MHz, $CDCl_3$)

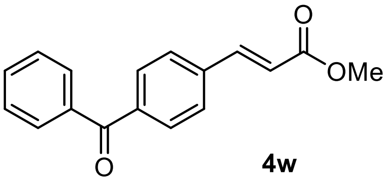

4w

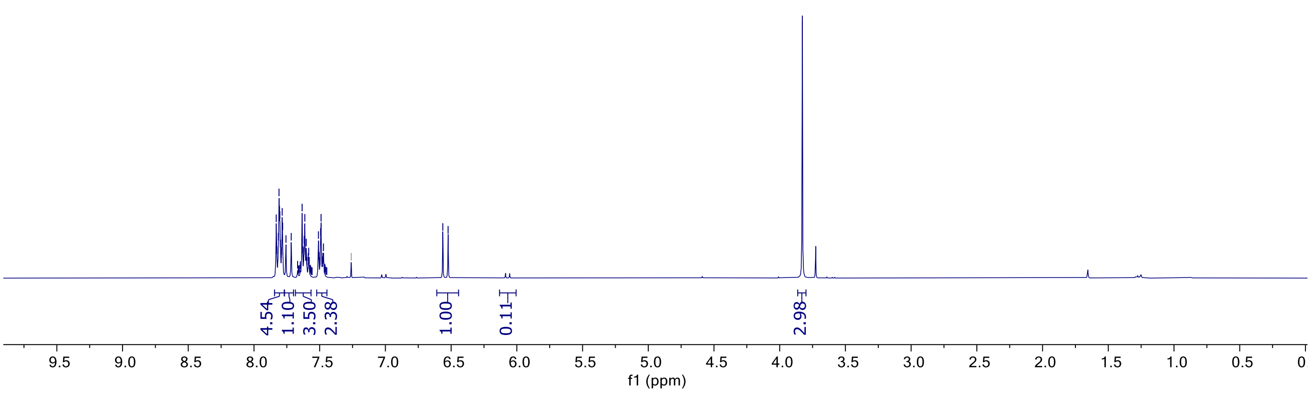

4w – ^{13}C-NMR (101 MHz, $CDCl_3$)

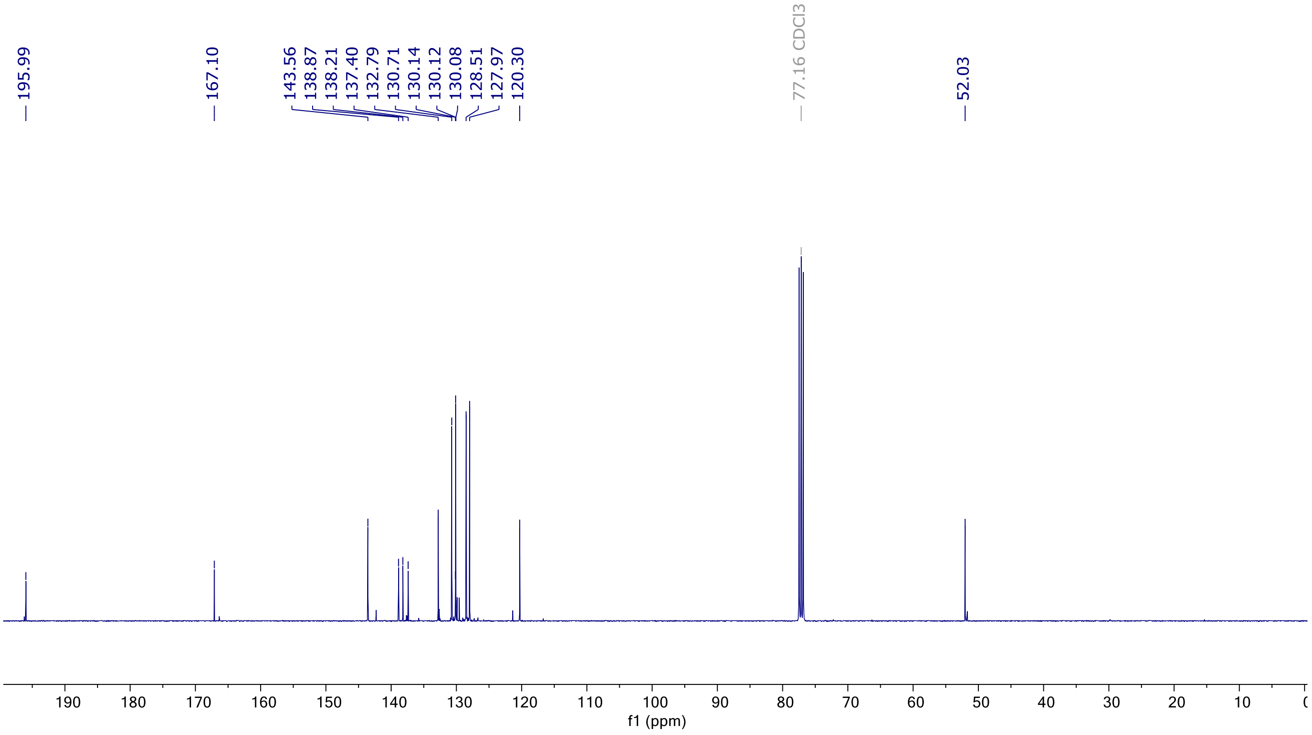

4x – ¹H-NMR (400 MHz, CDCl₃)

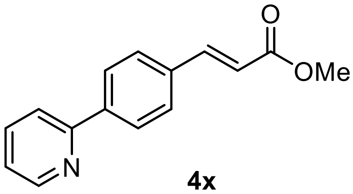

4x

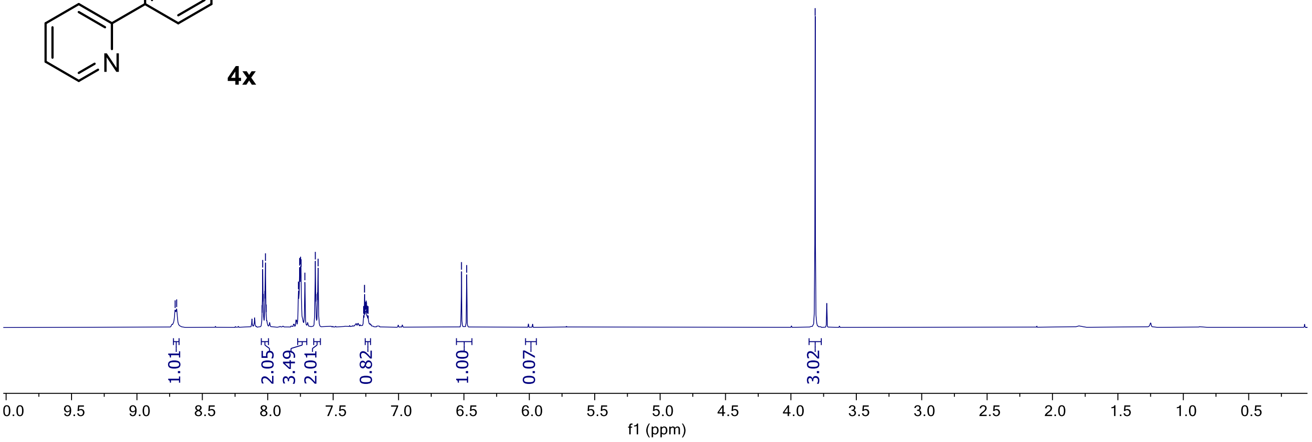

4x – ¹³C-NMR (101 MHz, CDCl₃)

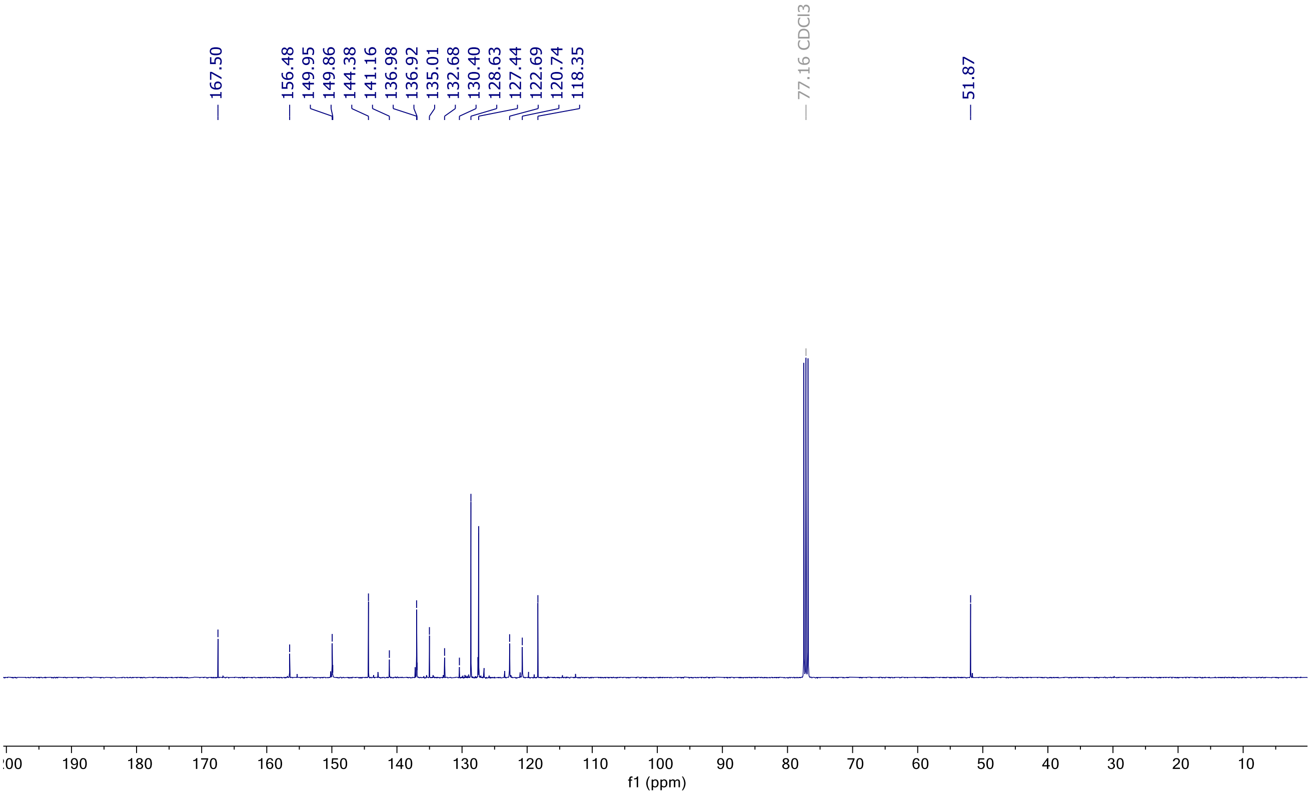

4y – ¹H-NMR (500 MHz, $CDCl_3$)

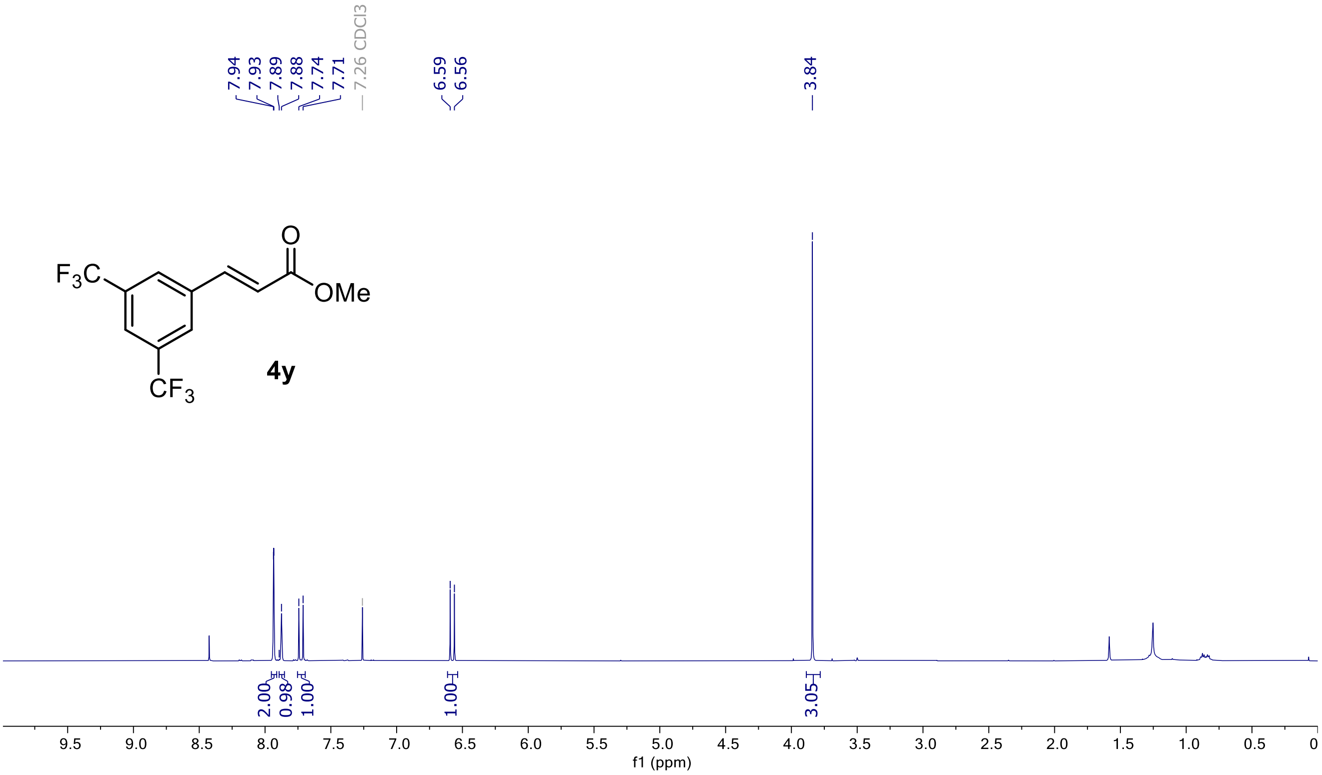

4y – ¹³C-NMR (126 MHz, $CDCl_3$)

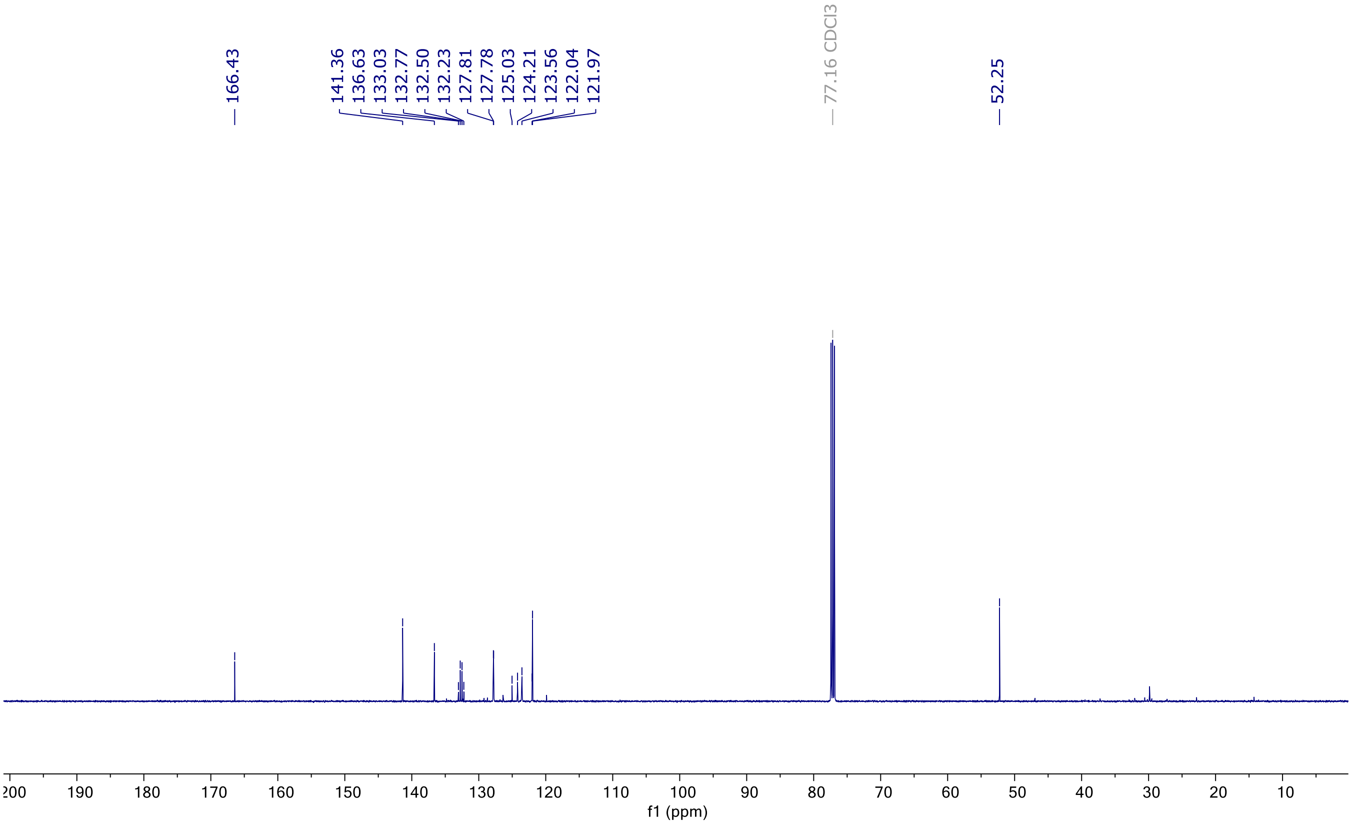

4z – ^{1}H-NMR (400 MHz, CDCl$_3$)

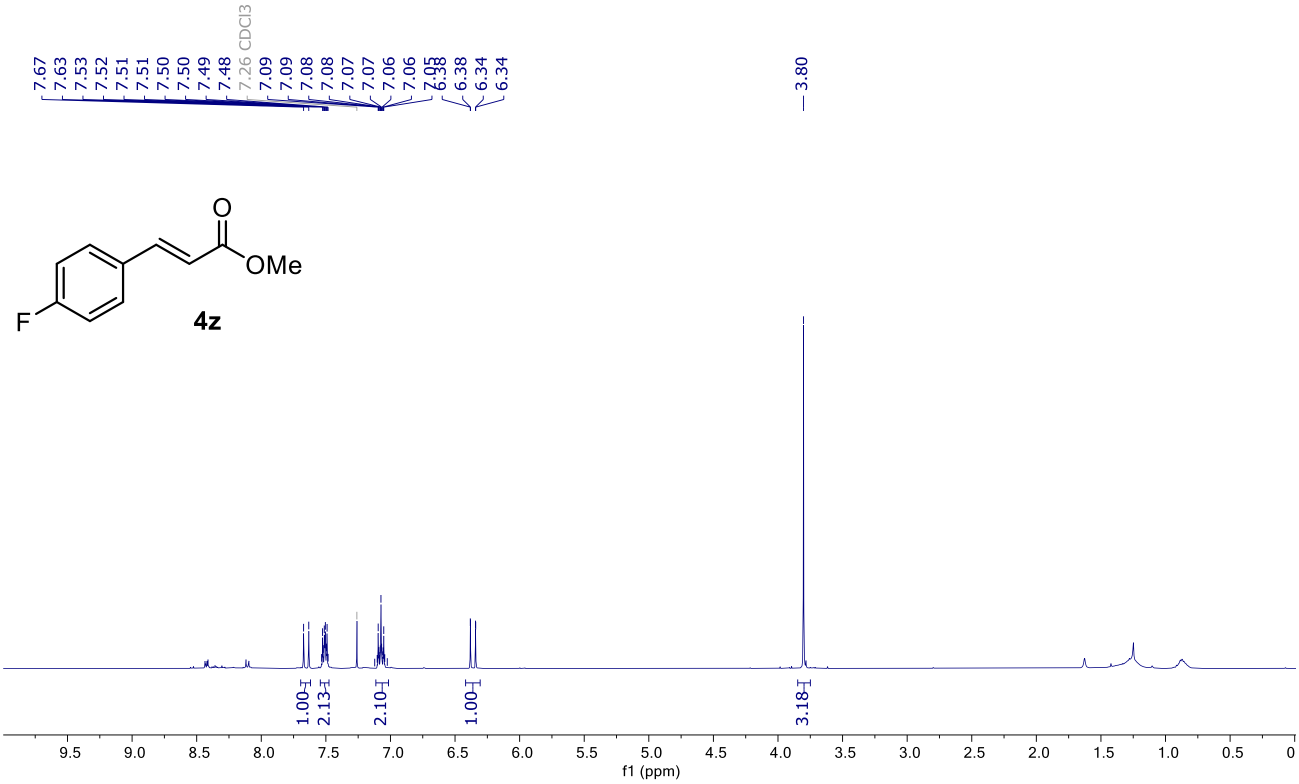

4z – ^{13}C-NMR (101 MHz, CDCl$_3$)

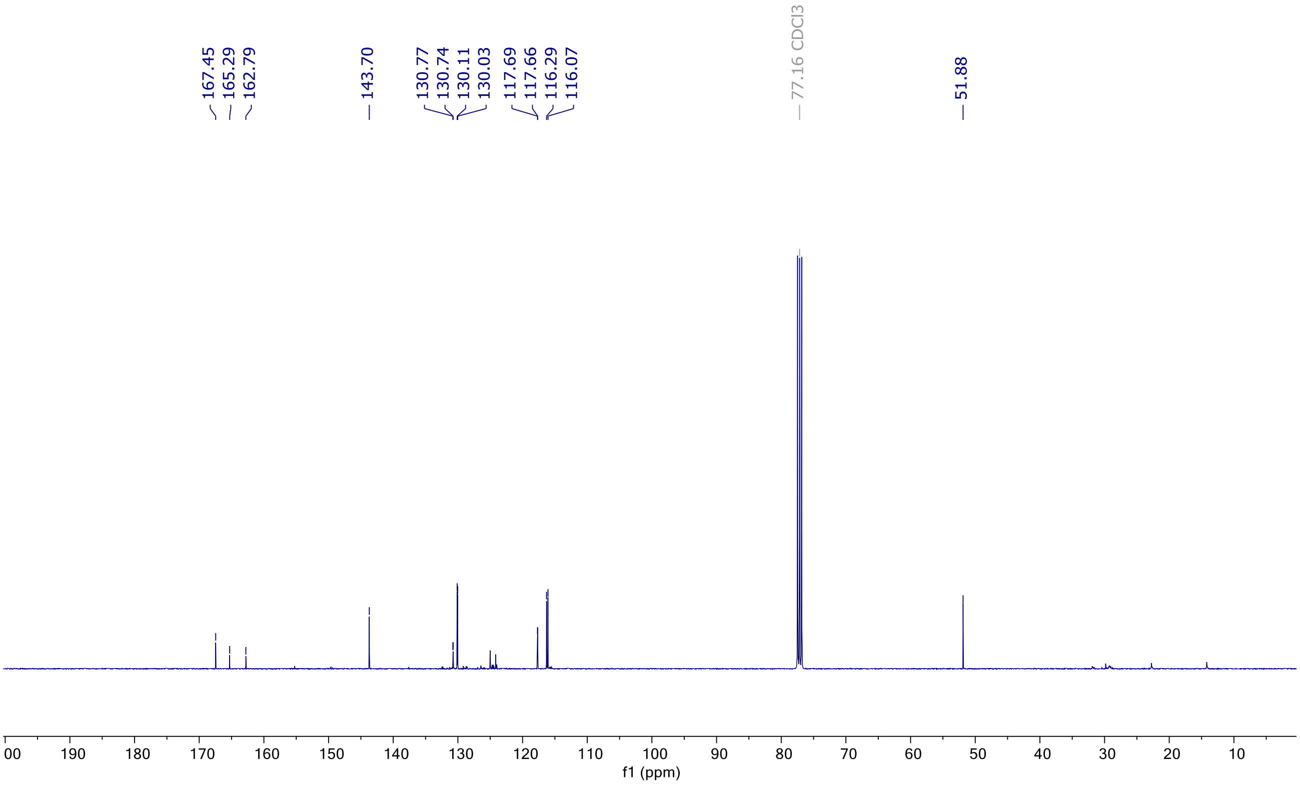

7.4 NMR Spectra of Alcohol Products

5aa – ^{1}H-NMR (400 MHz, $CDCl_3$)

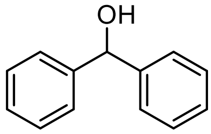

5aa

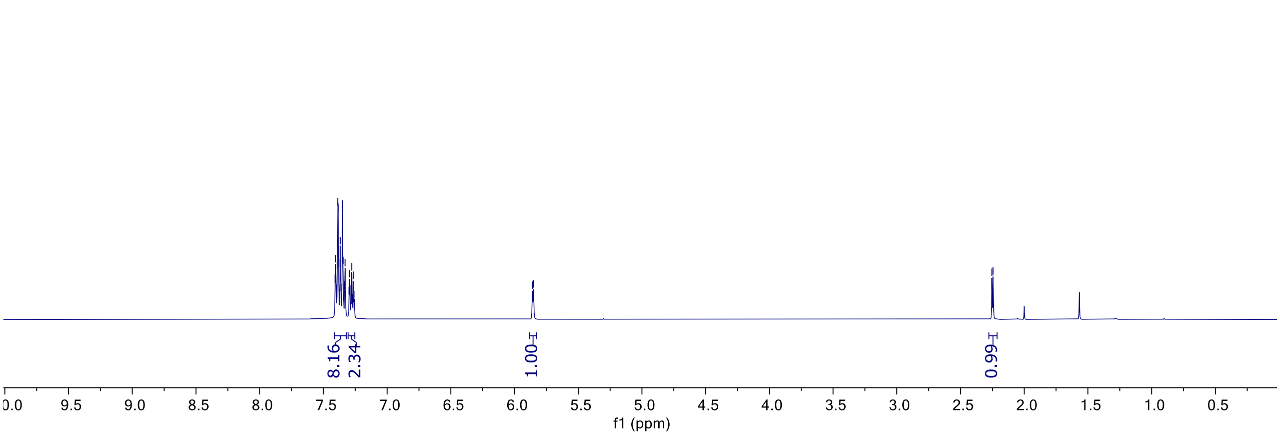

5aa – ^{13}C-NMR (101 MHz, $CDCl_3$)

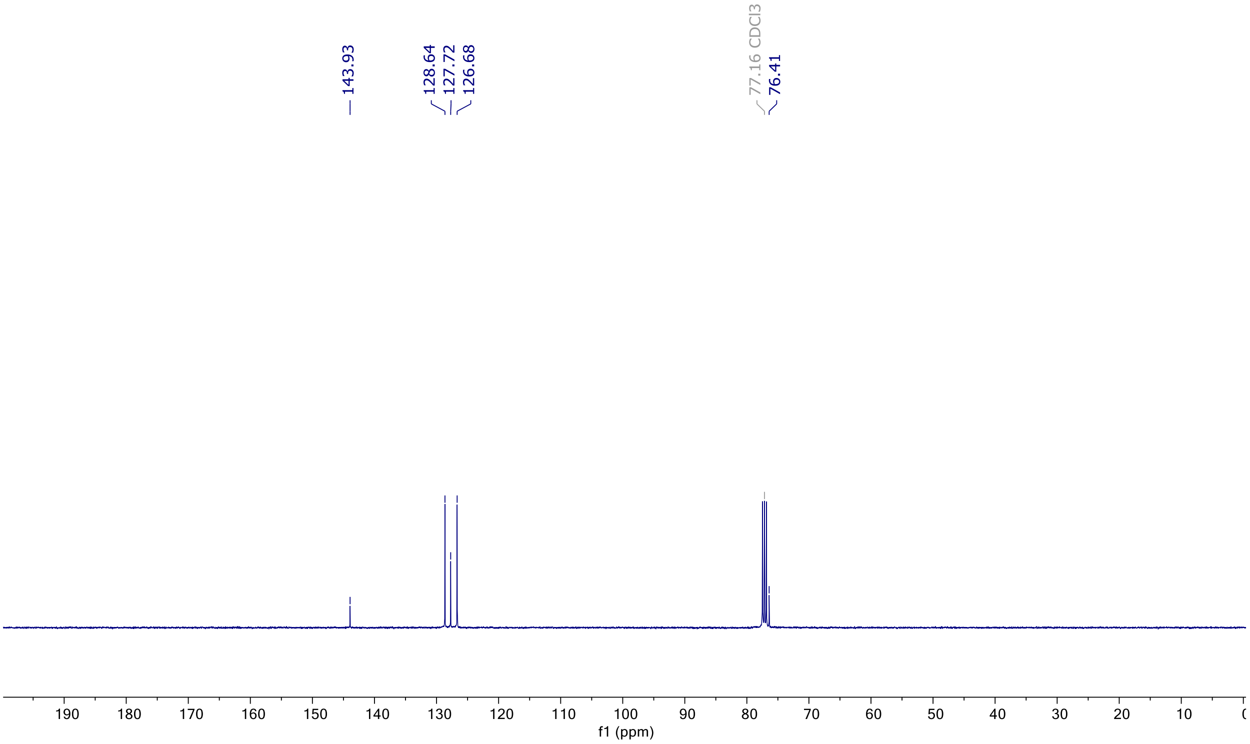

5ab – ^{1}H-NMR (400 MHz, $CDCl_3$)

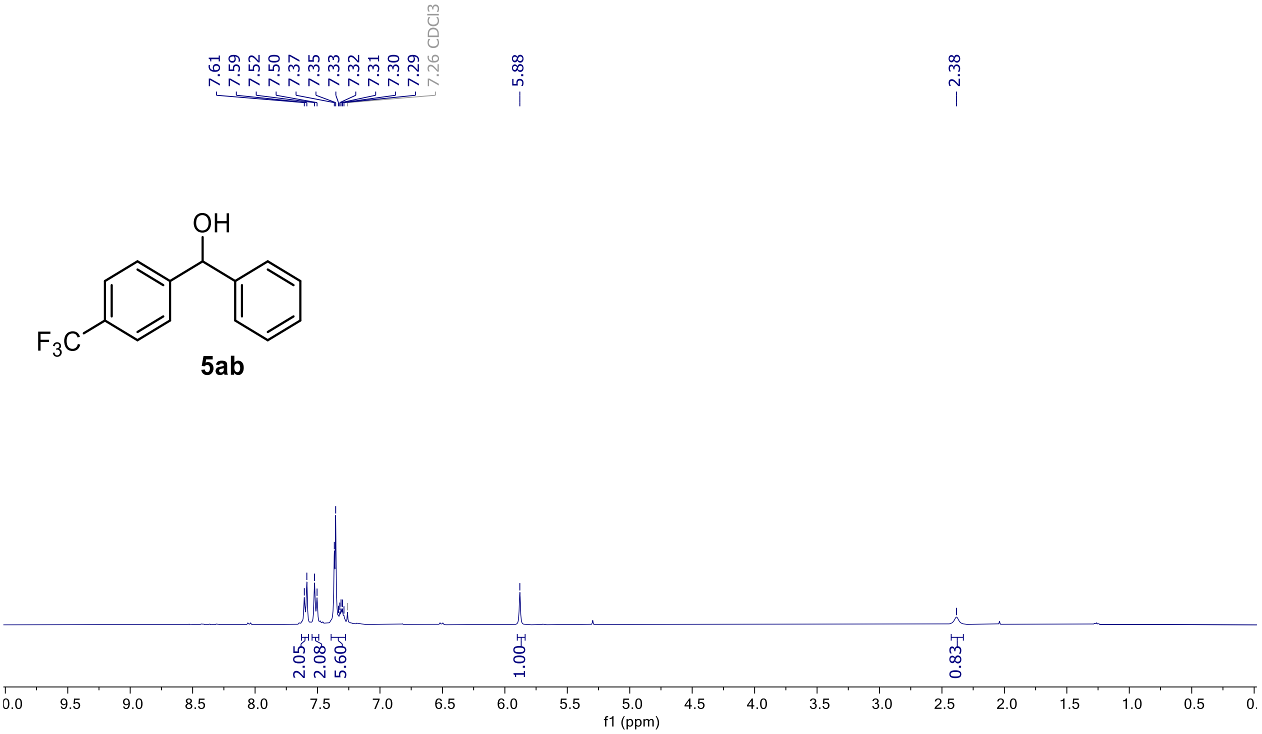

5ab – ^{13}C-NMR (101 MHz, $CDCl_3$)

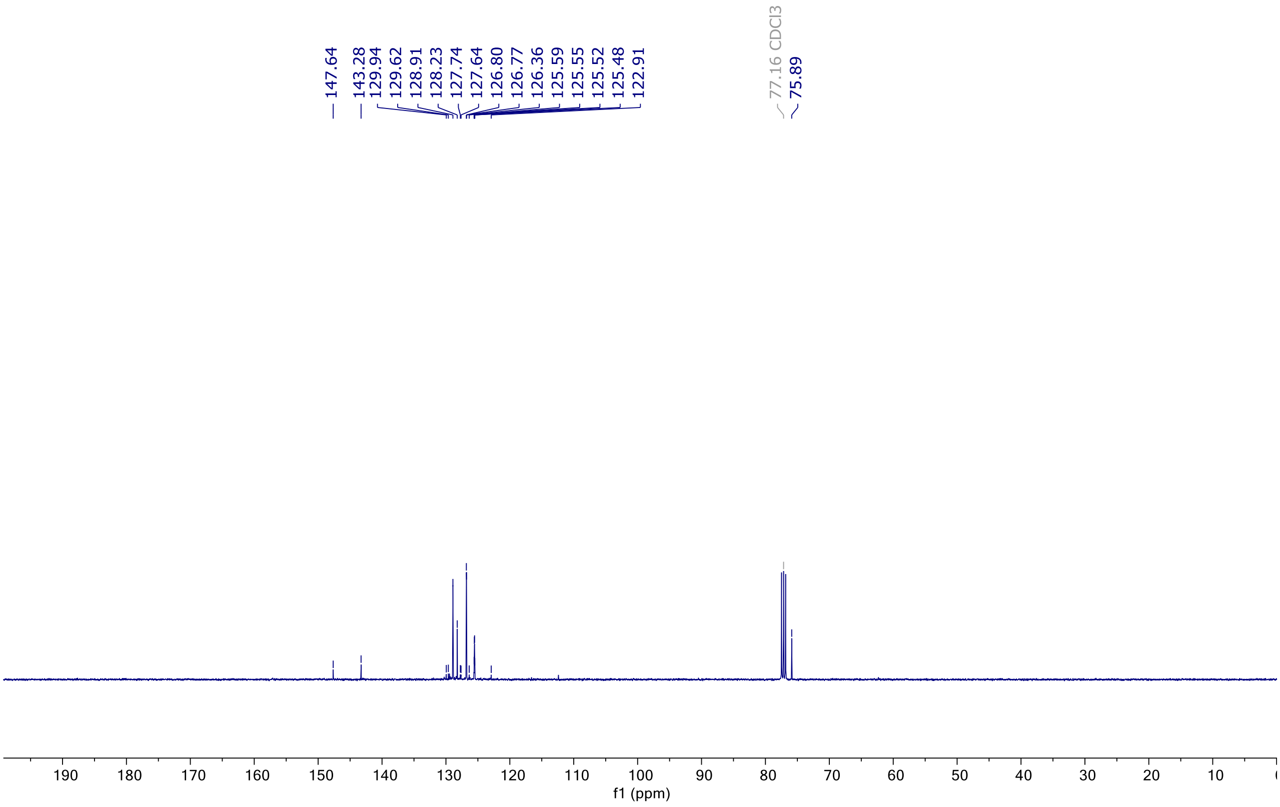

5ab – ^{19}F-NMR (376 MHz, $CDCl_3$)

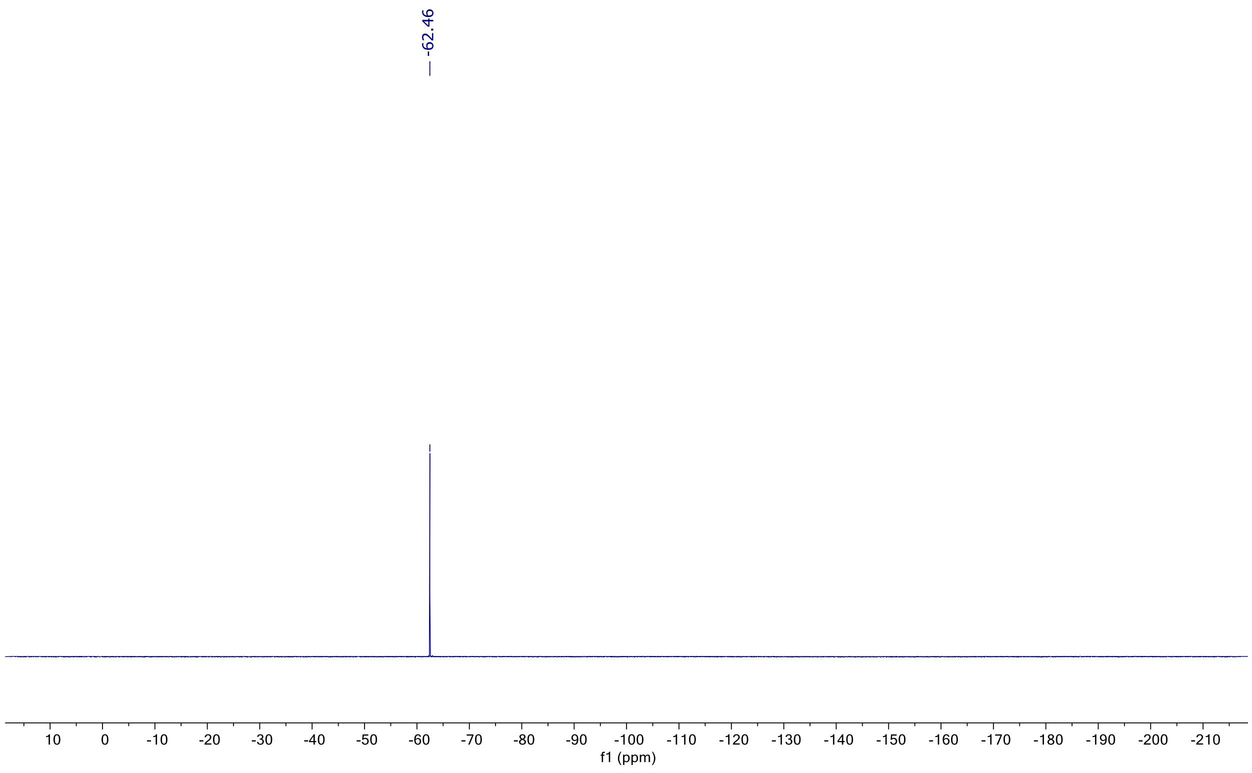

5ac – ^{1}H-NMR (400 MHz, $CDCl_3$)

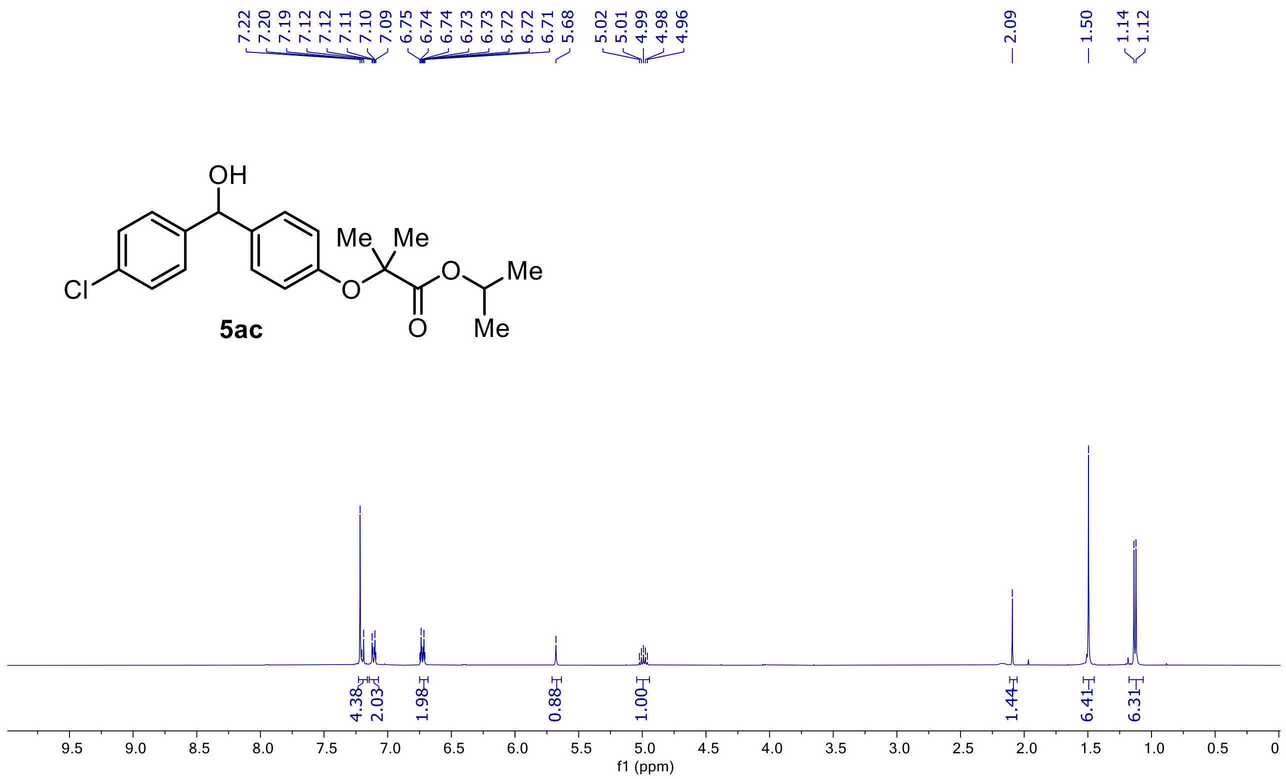

5ac – ^{13}C-NMR (101 MHz, $CDCl_3$)

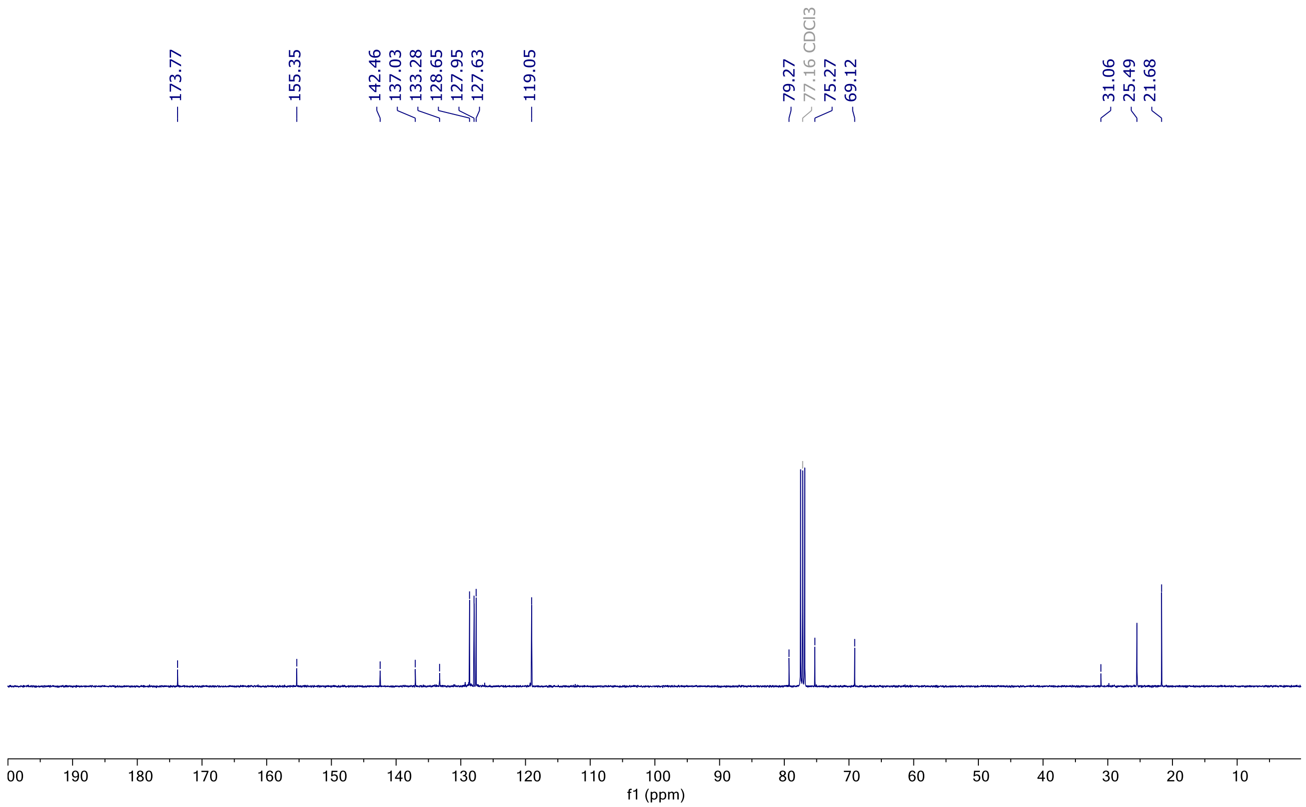

5ad – ^{1}H-NMR (400 MHz, $CDCl_3$) – mixture of diastereoisomers

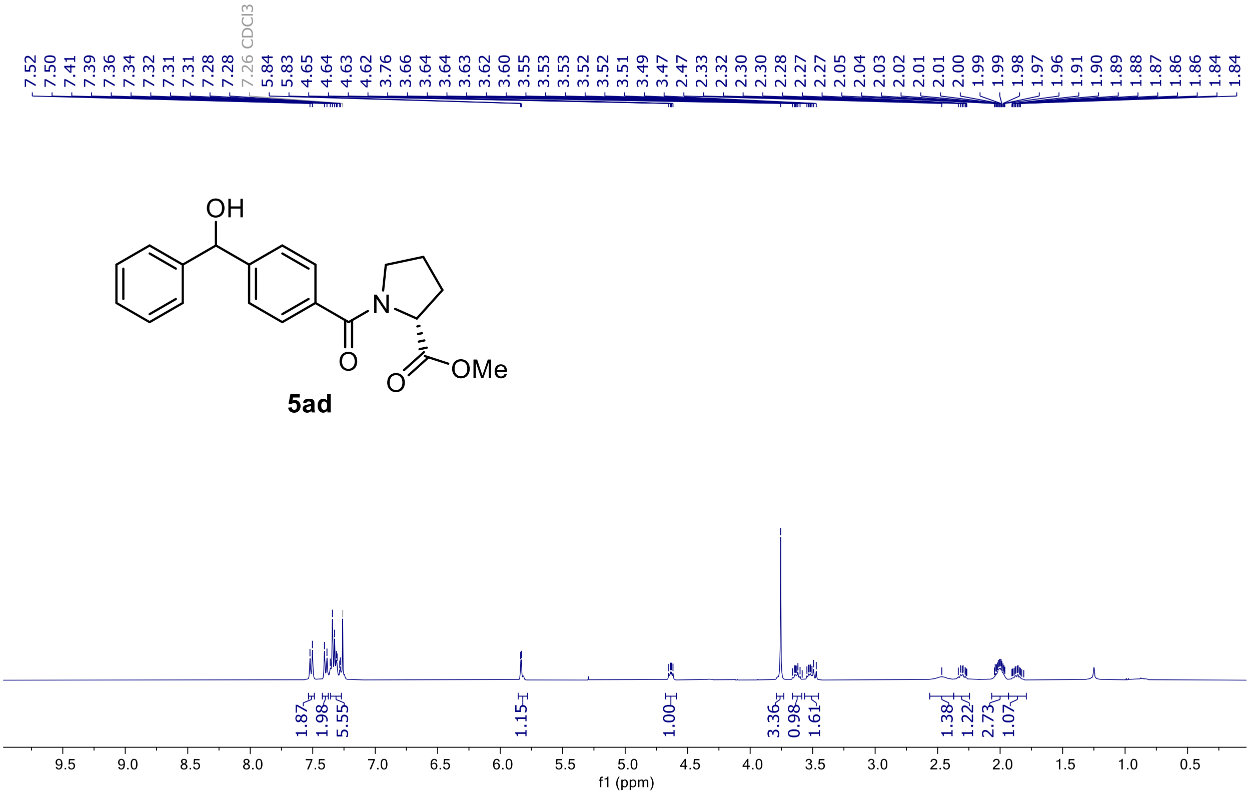

5ad – ^{13}C-NMR (101 MHz, $CDCl_3$) – mixture of diastereoisomers

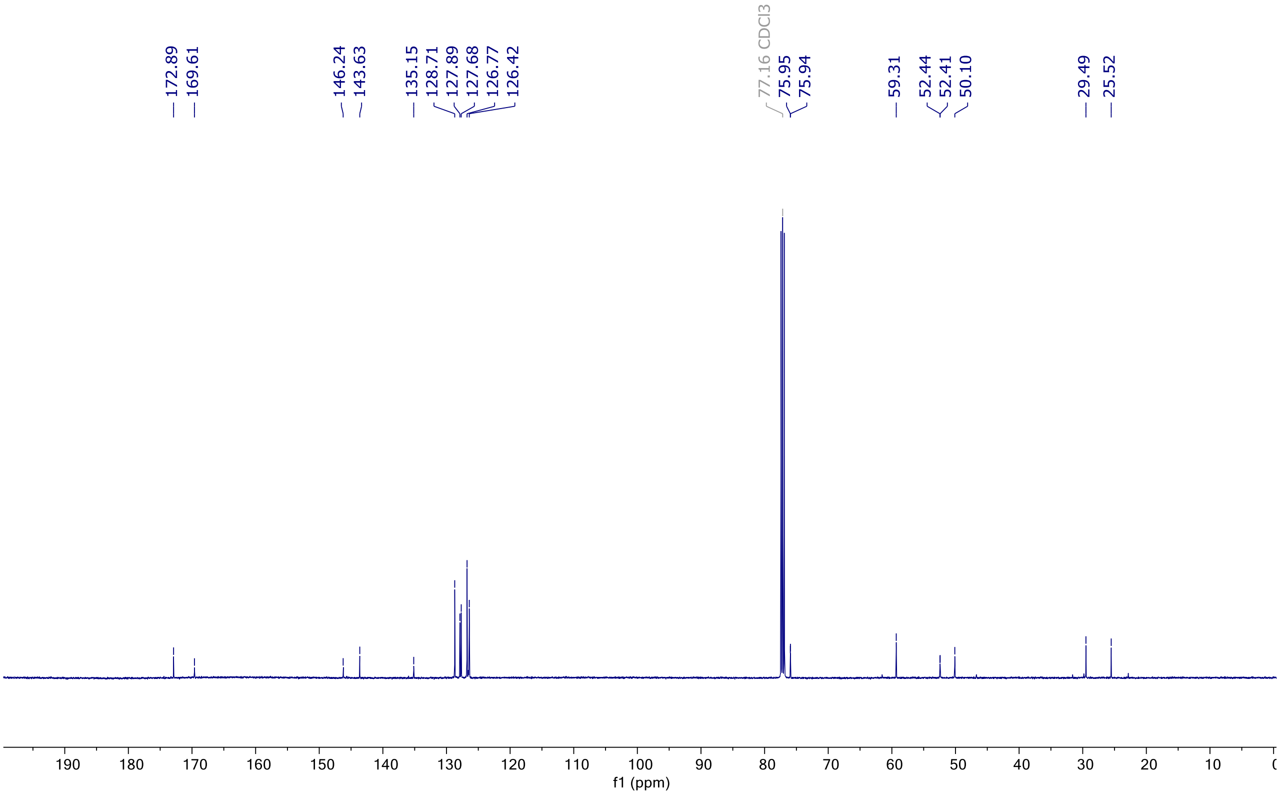

5ae – ^{1}H-NMR (700 MHz, $CDCl_3$) – mixture of diastereoisomers

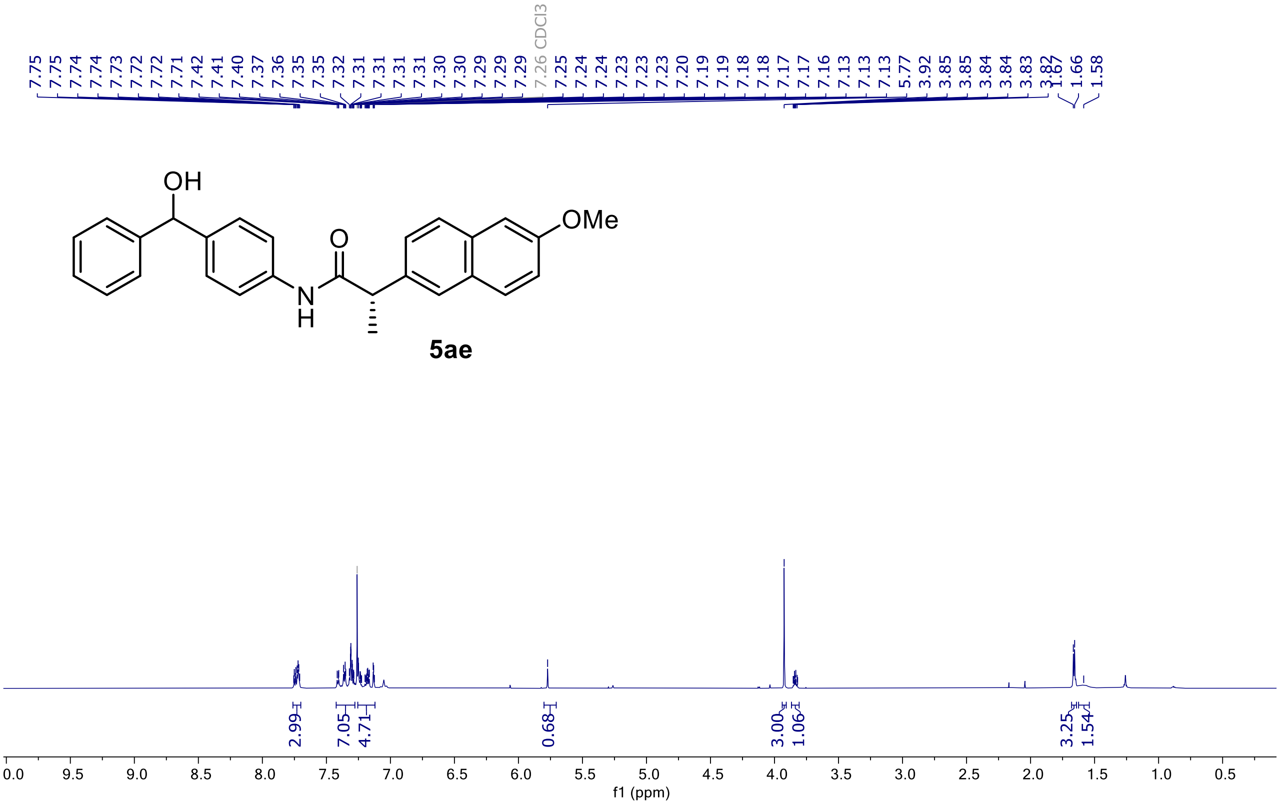

5ae

5ae – ^{13}C-NMR (176 MHz, $CDCl_3$) – mixture of diastereoisomers

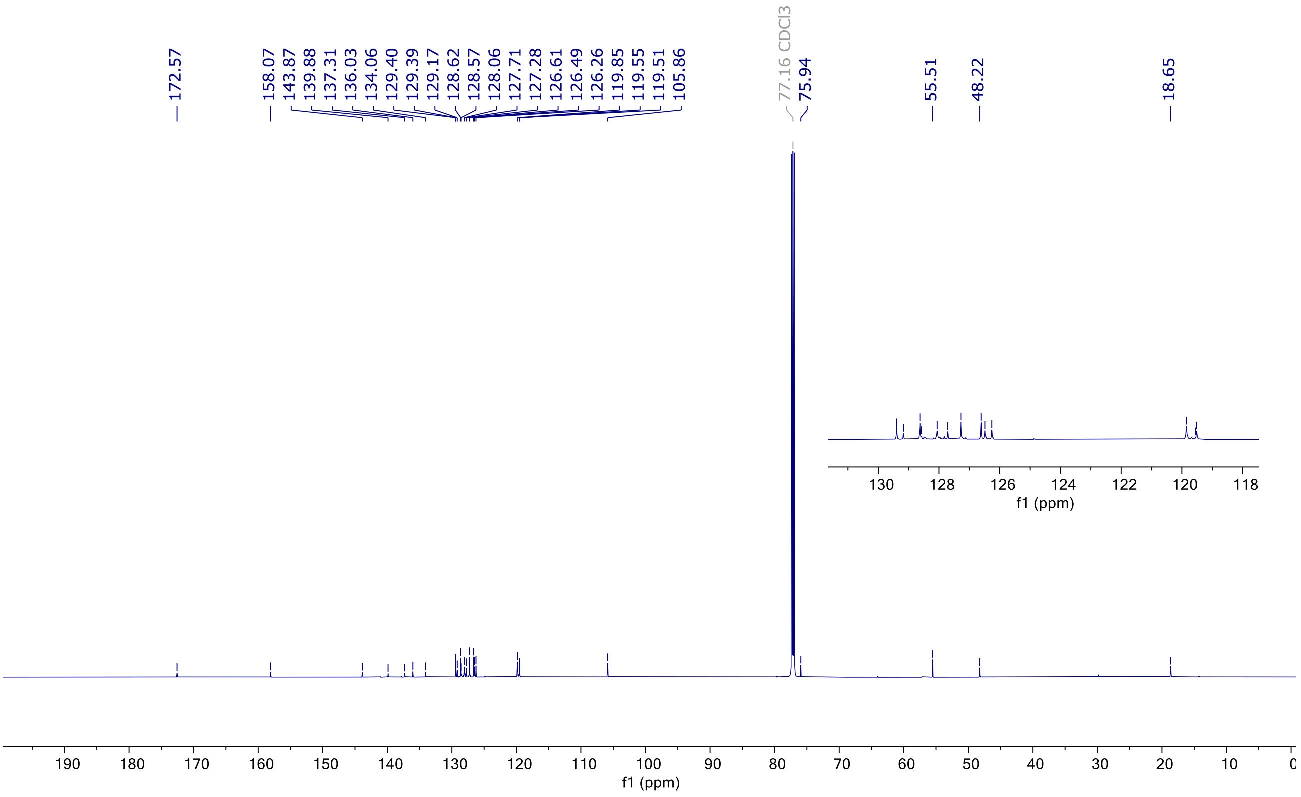

5af – ^{1}H-NMR (400 MHz, $CDCl_3$)

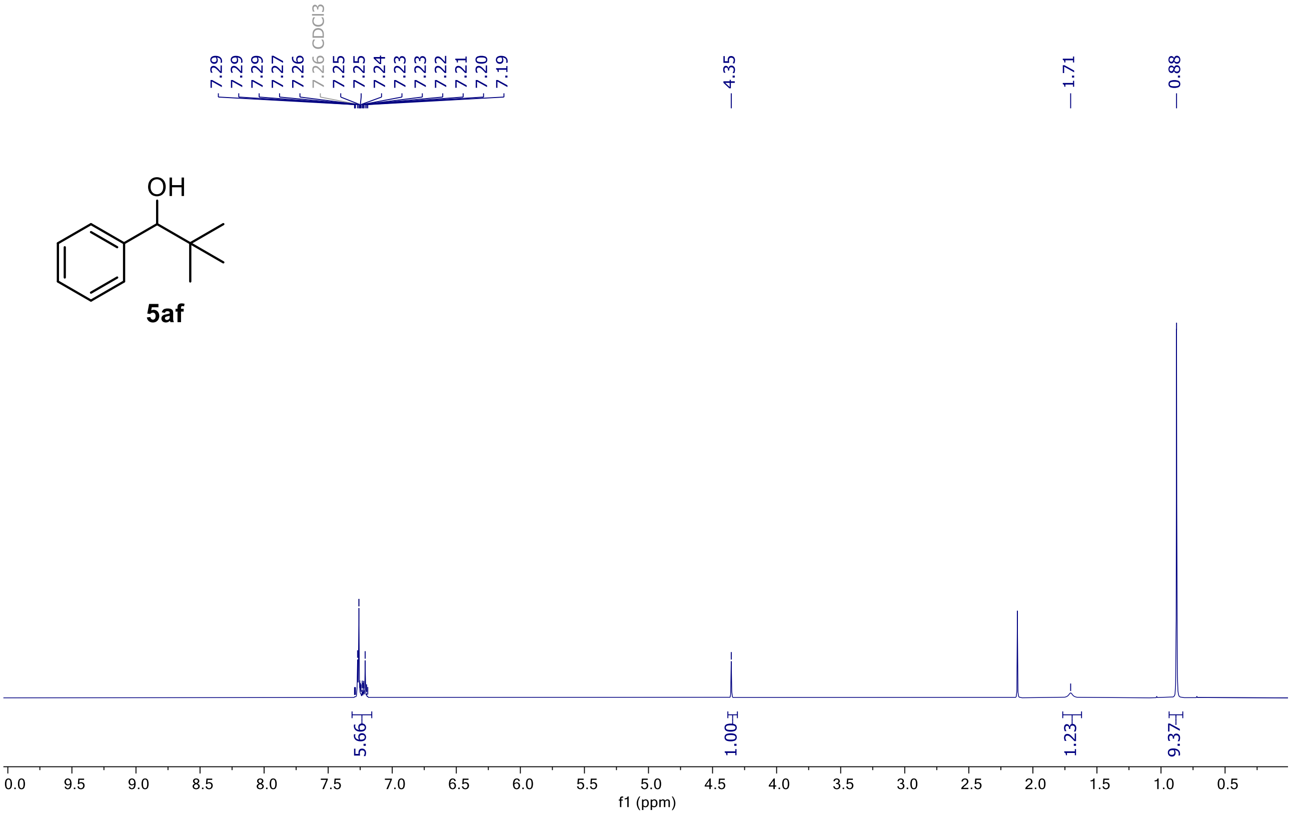

5af – ^{13}C-NMR (101 MHz, $CDCl_3$)

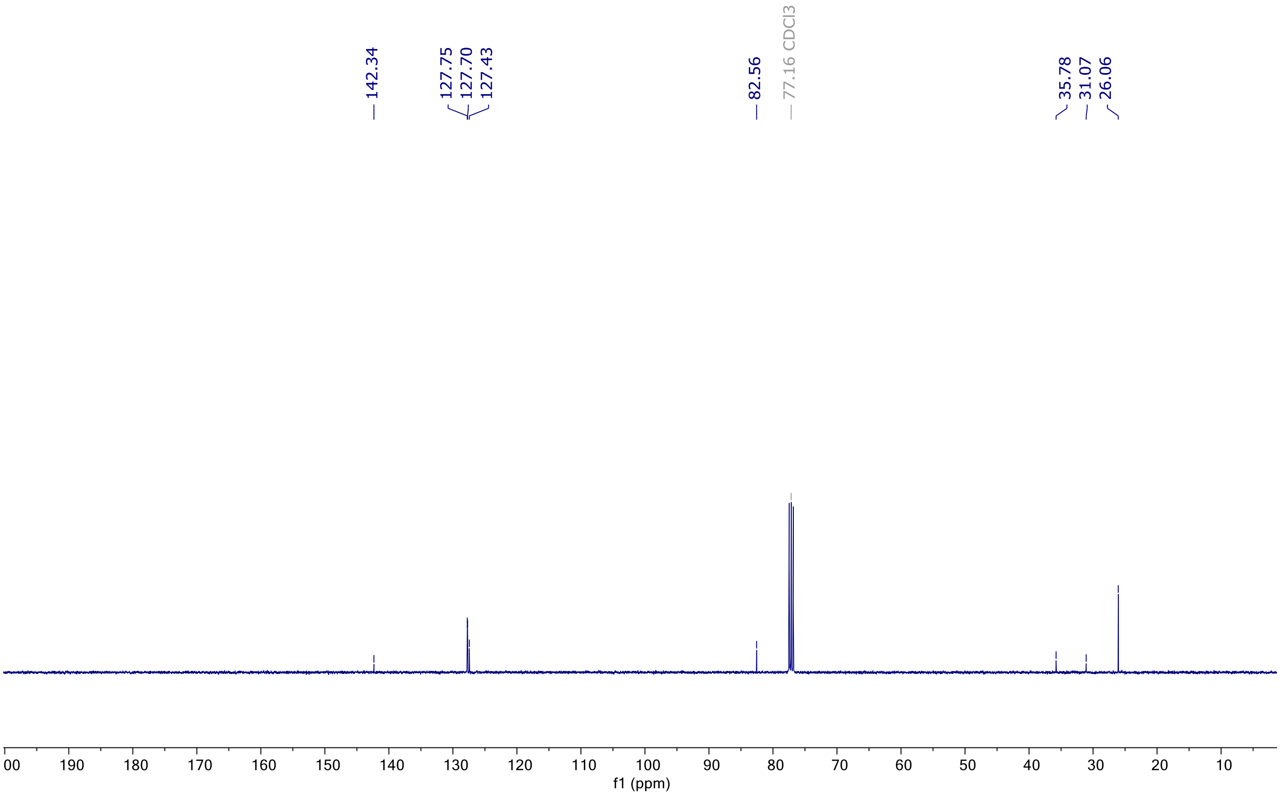

5ag – ^{1}H-NMR (400 MHz, $CDCl_3$) – mixture of diastereoisomers

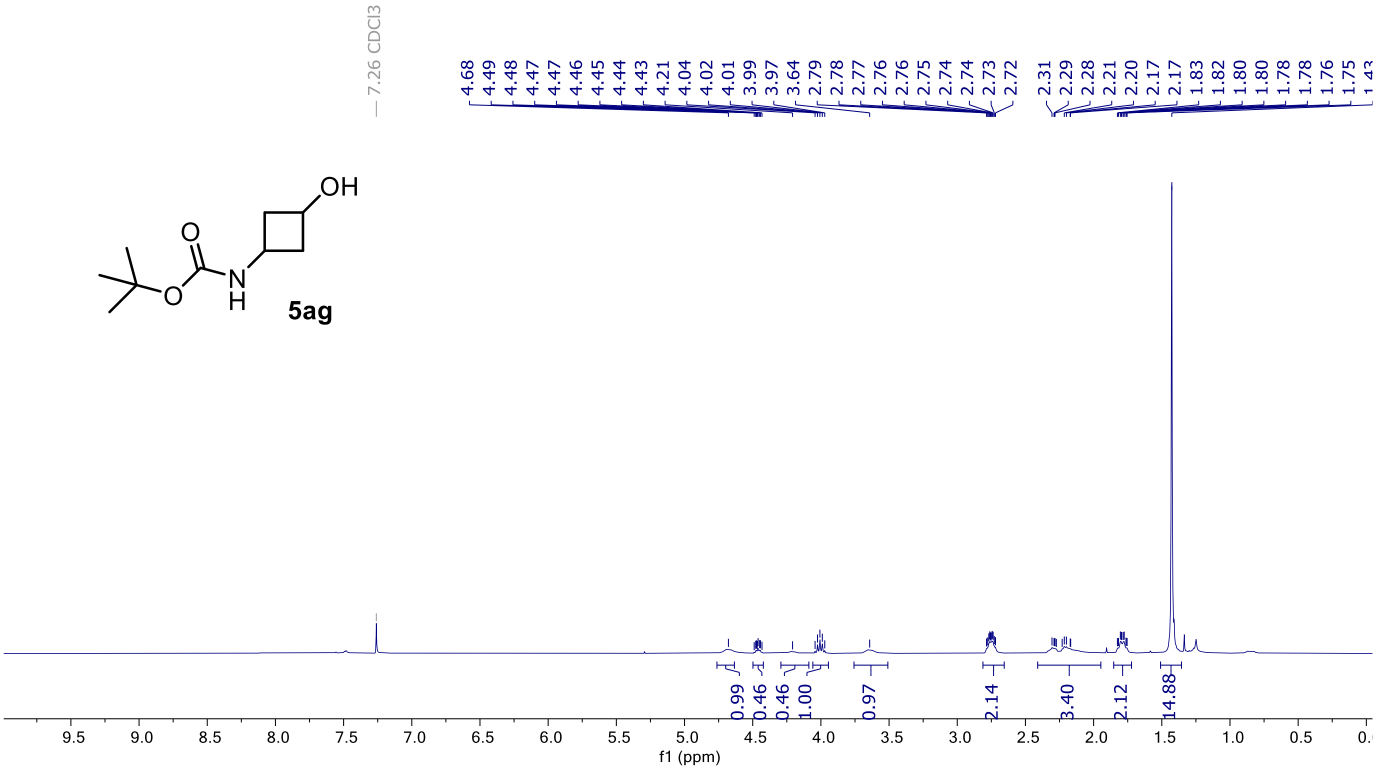

5ag – ^{13}C-NMR (101 MHz, $CDCl_3$) – mixture of diastereoisomers

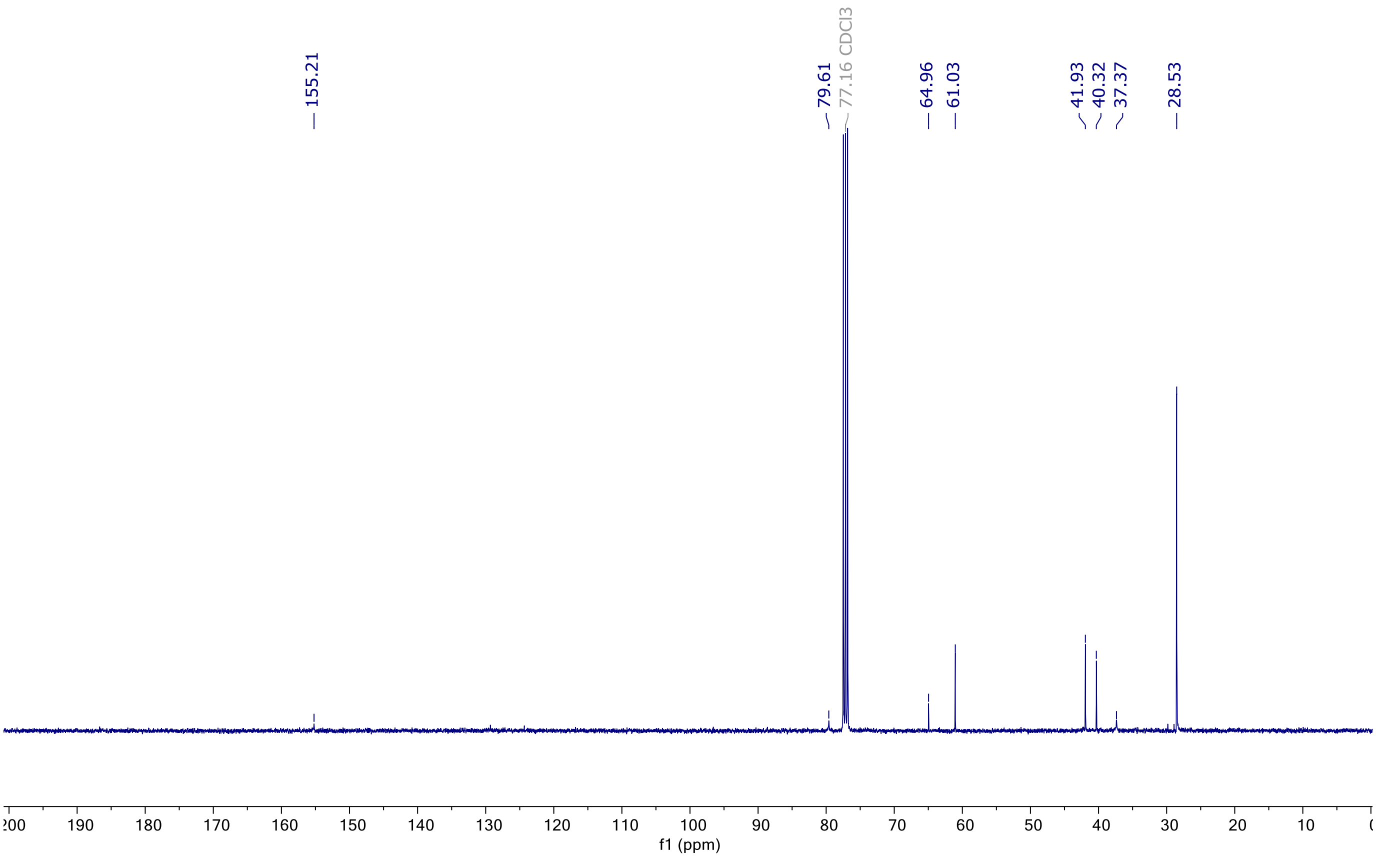

5ah – ^{1}H-NMR (400 MHz, CDCl$_3$) – single diastereoisomer

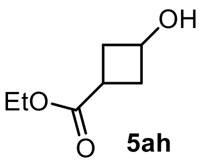

5ah

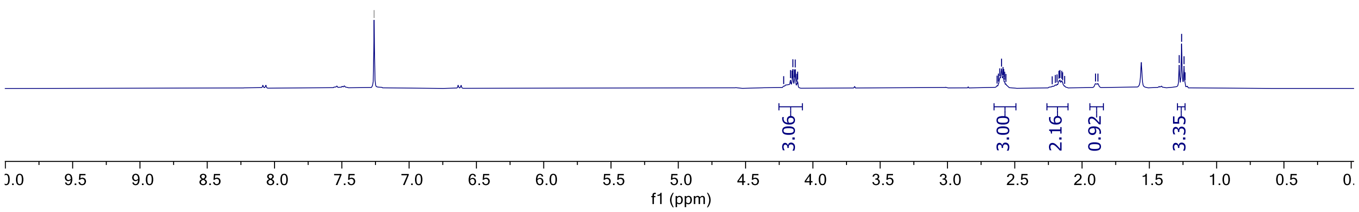

5ah – ^{13}C-NMR (101 MHz, CDCl$_3$) – single diastereoisomer

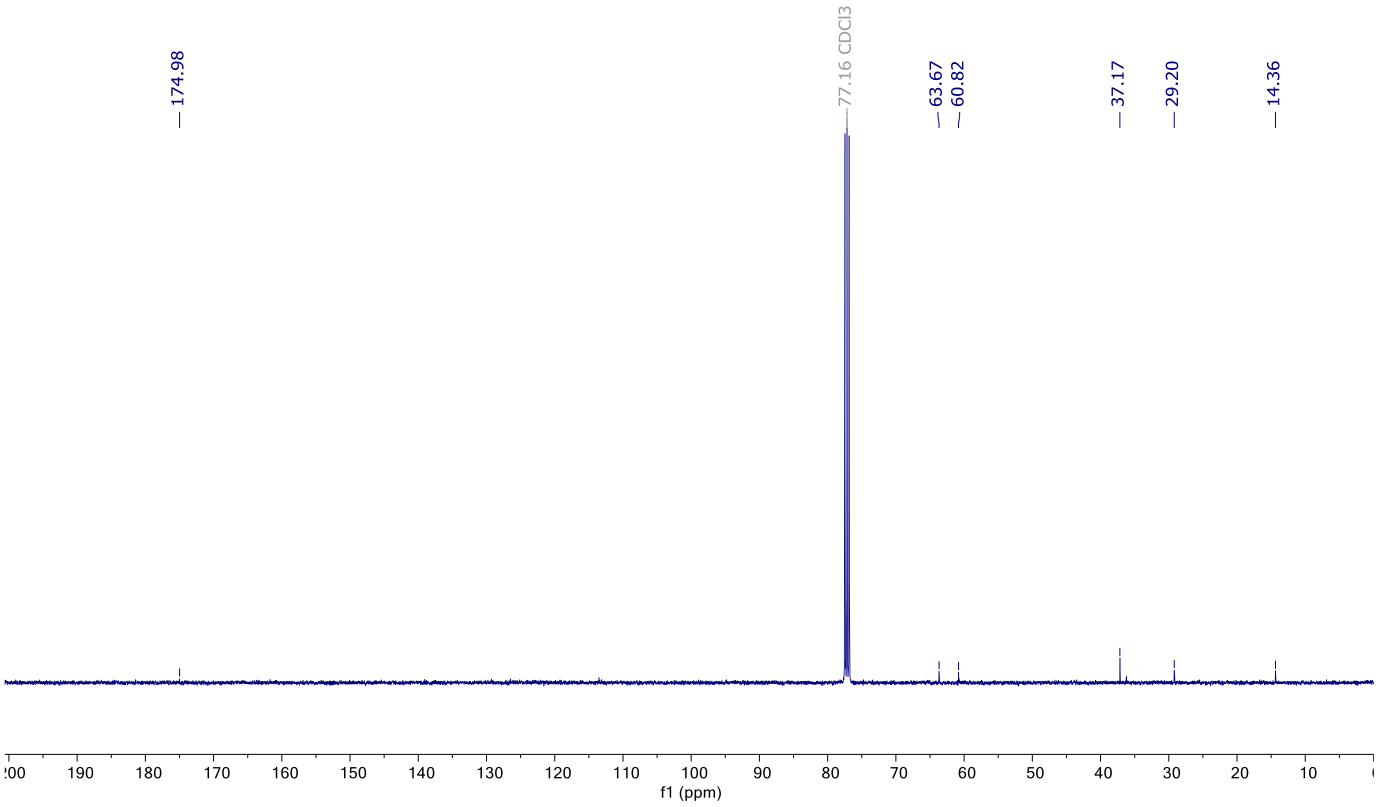

8 References

[1] A. K. Chakraborti, S. V. Chankeshwara, *Org. Biomol. Chem.* **2006**, *4*, 2769-2771.

[2] J. Kuzmin, J. Röckl, N. Schwarz, J. Djossou, G. Ahumada, M. Ahlquist, H. Lundberg, *Angew. Chem. Int. Ed.* **2023**, *62,* e202304272.

[3] G. Sun, X. Liu, J. Li, J.-X. Yang, J.-K. Xie, X. Wen, H. Sun, Q.-L. Xu, *New J. Chem.* **2023**, *47*, 4746-4751.

[4] Q. Zhu, J. Long, X. Song, K. Wang, J. Zeng, Y. Fan, *J. Org. Chem.* **2024**, *89*, 3726-3731.

[5] K. Sun, T. Sun, Y. Jiang, J. Shi, W. Sun, Y. Zheng, Z. Wang, Z. Li, X. Lv, X. Zhang, F. Luo, S. Liu, *Chem. Comm.* **2024**, *60*, 5755-5758.

[6] G. Hazra, A. Masarwa, *Org. Lett.* **2023**, *25*, 6396-6400.

[7] R. Pilli, K. Selvam, B. S. S. Balamurugan, V. Jose, R. Rasappan, *Org. Lett.* **2024**, *26*, 2993-2998.

[8] X.-H. Shan, M.-M. Wang, L. Tie, J.-P. Qu, Y.-B. Kang, *Org. Lett.* **2020**, *22*, 357-360.

[9] M. Obieta, G. Urgoitia, M. T. Herrero, R. Sanmartin, *Cat. Sci. Techn.* **2024**, *14*, 478-488.

[10] F. H. Cui, J. Chen, S. X. Su, Y. L. Xu, H. S. Wang, Y. M. Pan, *Adv. Syn. Cat.* **2017**, *359*, 3950-3961.

[11] A. O. Yoshihiro Nishimoto, Makoto Yasuda, Akio Baba, *Org. Lett.* **2012**, *14*, 1846-1849.

[12] D. Nawrot, M. Kolenič, J. Kuneš, F. Kostelansky, M. Miletin, V. Novakova, P. Zimcik, *Tetrahedron* **2018**, *74*, 594-599.

[13] K. Colas, R. Martín-Montero, A. Mendoza, *Angew. Chem. Int. Ed.* **2017**, *56*, 16042-16046.

[14] N. Barry, N. Brondel, S. E. Lawrence, A. R. Maguire, *Tetrahedron* **2009**, *65*, 10660-10670.

[15] D. D. Tanner, S. Koppula, P. Kandanarachchi, *J. Org. Chem.* **1997**, *62*, 4210-4215.

[16] A. R. Jadhao, S. S. Gaikwad, *J. Org. Chem.* **2023**, *88*, 14078-14087.

[17] A. Vulpetti, N. Ostermann, S. Randl, T. Yoon, A. Mac Sweeney, F. Cumin, E. Lorthiois, S. Rüdisser, P. Erbel, J. Maibaum, *ACS Med. Chem. Lett.* **2018**, *9*, 490-495.

[18] Y.-Z. Yang, Y. Li, G.-F. Lv, D.-L. He, J.-H. Li, *Org. Lett.* **2022**, *24*, 5115-5119.

[19] D. Raveenthrarajan, T. Satkunarajah, B. A. Kostiuk, M. J. Adler, *Chem. Eur. J.* **2023**, *29,* e202301063.

[20] R. E. Grote, E. R. Jarvo, *Org. Lett.* **2009**, *11*, 485-488.

[21] Y. Jiang, S. E. Schaus, *Angew. Chem. Int. Ed.* **2017**, *56*, 1544-1548.

[22] A. Mercadante, V. Campisciano, A. Morena, L. Valentino, V. La Parola, C. Aprile, M. Gruttadauria, F. Giacalone, *Eur. J. Org. Chem.* **2022**, *2022,* e202200497.

[23] X. Jia, Y. Wen, C. He, X. Huang, *Chin. J. Chem.* **2024**, *42*, 294-300.

[24] M.-L. Wang, H. Xu, H.-Y. Li, B. Ma, Z.-Y. Wang, X. Wang, H.-X. Dai, *Org. Lett.* **2021**, *23*, 2147-2152.

[25] H. Sahoo, L. Zhang, J. Cheng, M. Nishiura, Z. Hou, *J. Am. Chem. Soc.* **2022**, *144*, 23585-23594.

[26] S. Manzini, C. A. U. Blanco, S. P. Nolan, *Adv. Synth. Cat.* **2012**, *354*, 3036-3044.

[27] G. Dilauro, C. Luccarelli, A. F. Quivelli, P. Vitale, F. M. Perna, V. Capriati, *Angew. Chem. Int. Ed.* **2023**, *62,* e202304720.

[28] Q. Li, Y. Sun, M.-X. Fu, J.-H. Lin, J.-C. Xiao, *J. Org. Chem.* **2024**, *89*, 16022-16027.

[29] S. Hamada, M. Sumida, R. Yamazaki, Y. Kobayashi, T. Furuta, *J. Org. Chem.* **2023**, *88*, 12464-12473.

[30] J. Y. Wu, S. W. Schneller, K. L. Seley, E. De Clercq, *Heterocycles* **1998**, *47*, 757-763.
